# Revision of the Middle American clade of the ant genus *Stenamma* Westwood (Hymenoptera, Formicidae, Myrmicinae)

**DOI:** 10.3897/zookeys.295.4905

**Published:** 2013-04-24

**Authors:** Michael G. Branstetter

**Affiliations:** 1 Department of Entomology, University of California, Davis, CA 95616, USA; 2 Department of Entomology, National Museum of Natural History, Smithsonian Institution, Washington, DC 20560, USA

**Keywords:** Stenammini, systematics, taxonomy, cryptic ants, Neotropics, Central America, cloud forest

## Abstract

*Stenamma* is a cryptic “leaf-litter” ant genus that occurs in mesic forest habitats throughout the Holarctic region, Central America, and part of northwestern South America (Colombia and Ecuador). The genus was thought to be restricted primarily to the temperate zone, but recent collecting efforts have uncovered a large radiation of Neotropical forms, which rival the Holarctic species in terms of morphological and behavioral diversity. By inferring a broad-scale molecular phylogeny of *Stenamma*, Branstetter (2012) showed that all Neotropical species belong to a diverse Middle American clade (MAC), and that this clade is sister to an almost completely geographically separated Holarctic clade (HOC). Here, the Middle American clade of *Stenamma* is revised to recognize 40 species, of which 33 are described as new. Included in the revision are a key to species based on the worker caste, and for each species where possible, descriptions and images of workers and queens, images of males, information on geographic distribution, descriptions of intraspecific variation, and notes on natural history. Several species groups are defined, but the majority of species remain unassigned due to a lack of diagnostic morphological character states for most molecular clades. The following species are redescribed: *Stenamma alas* Longino, *Stenamma diversum* Mann, *Stenamma expolitum* Smith, *Stenamma felixi* Mann, *Stenamma huachucanum* Smith, *Stenamma manni* Wheeler, and *Stenamma schmidti* Menozzi. The following are described as new: *Stenamma andersoni* **sp. n.**, *Stenamma atribellum* **sp. n.**, *Stenamma brujita* **sp. n.**, *Stenamma callipygium* **sp. n.**, *Stenamma catracho* **sp. n.**, *Stenamma connectum* **sp. n.**, *Stenamma crypticum* **sp. n.**, *Stenamma cusuco* **sp. n.**, *Stenamma excisum* **sp. n.**, *Stenamma expolitico* **sp. n.**, *Stenamma hojarasca* **sp. n.**, *Stenamma ignotum* **sp. n.**, *Stenamma lagunum* **sp. n.**, *Stenamma llama* **sp. n.**, *Stenamma leptospinum* **sp. n.**, *Stenamma lobinodus* **sp. n.**, *Stenamma longinoi* **sp. n.**, *Stenamma maximon* **sp. n.**, *Stenamma megamanni* **sp. n.**, *Stenamma monstrosum* **sp. n.**, *Stenamma muralla* **sp. n.**, *Stenamma nanozoi* **sp. n.**, *Stenamma nonotch* **sp. n.**, *Stenamma ochrocnemis* **sp. n.**, *Stenamma pelophilum* **sp. n.**, *Stenamma picopicucha* **sp. n.**, *Stenamma saenzae* **sp. n.**, *Stenamma sandinista* **sp. n.**, *Stenamma stictosomum* **sp. n.**, *Stenamma tiburon* **sp. n.**, *Stenamma tico* **sp. n.**, *Stenamma vexator* **sp. n.**, and *Stenamma zelum* **sp. n.** Although many of the newly defined species consist of challenging species complexes, this study establishes a robust baseline that will guide future work on the systematics of MAC *Stenamma*. The total global diversity of *Stenamma* now includes 84 extant species.

## Introduction

The genus *Stenamma* Westwood comprises a taxonomically challenging but intriguing lineage of myrmicine ants, which occupy mesic forest habitats throughout most of the northern temperate zone, Middle America, and northwestern South America (Branstetter 2009, 2012, Bolton 2013). The genus is generally characterized by its species having cryptic, unobtrusive habits, and an exceptional tolerance of cool, wet environments, with many being active and abundant in high-elevation cloud forests, or during the winter months in snow-free temperate woodlands and scrublands. *Stenamma* also has been branded a “leaf-litter ant genus,” because it is encountered most easily by sampling moist detritus from the forest floor. Beyond these generalizations, it is slowly becoming apparent that the life history strategies of *Stenamma* species are quite diverse, and in some cases unique among ants (see Smith 1957, Longino 2005, Branstetter 2012, this study).

Understanding of the diversity and distribution of *Stenamma* has progressed relatively slowly compared to more conspicuous ant genera. This is because of a general lack of material, poor representation of colony series and reproductive castes in collections, and the tendency for worker morphology to be both conservative and convergent among species. Despite these obstacles, the genus has received considerable attention in the Holarctic region, with the publication of many species descriptions, as well as several large regional revisions (Smith 1957, Snelling 1973, DuBois 1998, Rigato 2011). The Neotropical fauna, in contrast, has been largely overlooked, with only a few species being described over the last century (Wheeler 1914, Mann 1922, Menozzi 1931a, Smith 1962, Longino 2005). Because of this disparity, *Stenamma* is perceived as a predominately Holarctic group, with a few aberrant species penetrating into the Neotropics. This view, however, is no longer tenable as increased collecting efforts in the region over the last fifty years, combined with greater use of leaf-litter extraction techniques (e.g. Winkler bags, Berlese funnels), have revealed a trove of new species in Middle America. Together, these new forms rival the Holarctic fauna in terms of morphological, ecological and species-level diversity.

On a global scale, understanding of *Stenamma* has been greatly improved by several recent studies that incorporate molecular data. First, two investigations looking at higher-level relationships among ants found that *Stenamma* is closely related to the genera *Aphaenogaster* Mayr, *Messor* Forel, *Goniomma* Emery, and *Oxyopomyrmex* André (Brady et al. 2006, Moreau et al. 2006). Although not formally described, this clade of genera will likely form a revised version of the tribe Stenammini, which as currently defined is not monophyletic. Next, Branstetter (2009) used molecular and morphological data to redefine *Stenamma*,providing a new diagnosis of the worker caste, as well as evidence that *Stenamma* is likely monophyletic. Lastly, and most pertinent to this study, Branstetter (2012) inferred a densely sampled broad-scale phylogeny of *Stenamma*, revealing that the genus is composed of two, well-supported clades: a “Holarctic clade” (HOC) and a “Middle American clade” (MAC).

**Figure 1.**
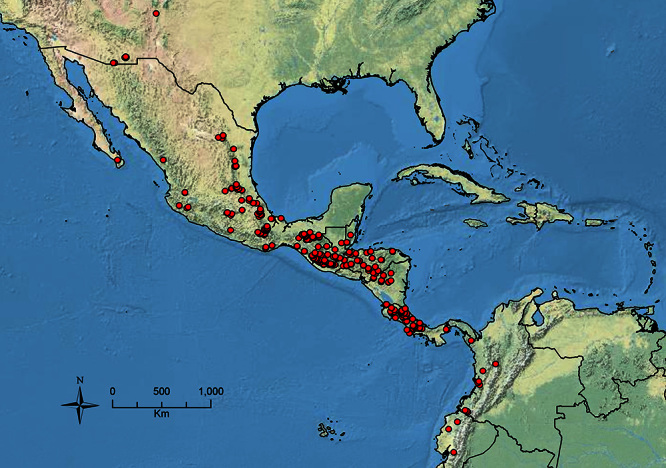
Map showing the global distribution of the Middle American clade of *Stenamma*.

The HOC currently consists of 44 extant species distributed across the Nearctic and Palearctic regions, with most Nearctic forms occurring north of Mexico (only a few records from northern Baja California). Species in the MAC occur from the southwestern U.S.A. to northwestern South America (Colombia, Ecuador), with most of the diversity occurring in the wet forests of eastern Mexico and Central America (Figure 1). The only MAC species to reach the U.S.A. and to co-occur with HOC species is *Stenamma huachucanum* Smith, which is known from the sky islands of Arizona, New Mexico, and Texas, as well as from many sites in Mexico (Texas record in Van Pelt 1983). Therefore, the two clades are almost completely geographically isolated from one another. Within the MAC, Branstetter (2012) further identified two main clades, a depauperate northern clade, which includes *Stenamma excisum* sp. n. and *Stenamma lagunum* sp. n., and the “MAC core,” which contains the majority of Neotropical diversity.

I present here the first comprehensive revision of the Middle American clade of *Stenamma*, recognizing 40 species, of which 33 are described as new. This work represents the first all-in-one treatment of Neotropical taxa since Smith’s (1962) review, which included only five species. It is also the first clade-based, rather than region-based *Stenamma* revision, taking advantage of the strong phylogenetic framework described above. The focus of this study is on the worker caste, but descriptions of the queen caste, and images of both the queen and male castes are provided when possible. Many of the species described here represent difficult complexes, and in most cases, these are treated as widespread, polytypic species. Although additional data are needed to refine species boundaries within complexes, this study establishes a robust baseline for future work on the systematics of MAC *Stenamma*.

The first realization that Middle America contained a diverse radiation of previously unknown *Stenamma* species came from collections made in Costa Rica by J. T. Longino and the Arthropods of La Selva project (ALAS; http://viceroy.eeb.uconn.edu/ALAS/ALAS.html), which he and R. Colwell organized. Later, it was discovered that several coleopterists (R. S. Anderson, A. F. Newton, S. B. Peck), who used Berlese funnels extensively, had collected many *Stenamma* specimens as bycatch over the last 50 years from throughout Mexico and Central America. Much of this material was processed at various institutions, but was aggregated at the Los Angeles County Museum (LACM) by the late R. R. Snelling who began to reviseNeotropical *Stenamma* (Snelling pers. comm.). Additional material from the coleopterists was stored in the Field Museum of Natural History (FMNH) bulk sample collection and reviewed by myself. Over the last six years, a very large amount of material has come from the Leaf Litter Arthropods of MesoAmerica project (LLAMA; https://sites.google.com/site/longinollama/), organized by J. T. Longino and R. S. Anderson. This project has intensively sampled leaf litter insects from tropical wet forests in Chiapas, Guatemala, Honduras, and Nicaragua. Many new species were collected by LLAMA, but just as importantly, the project has provided fresh specimens for the molecular studies described above and on-going work. A significant amount of material has come also from personal collections made during LLAMA expeditions.

## Overview of natural history

Biological information specific to individual species is provided below in the species accounts. Here I present a summary of what is known about the natural history of MAC *Stenamma* species. The biology of Nearctic and Palearctic species is provided in Smith (1957) and DuBois (1998), respectively.

Neotropical *Stenamma* species are found mainly in mesic forest habitats from sea level to around 3000 m elevation, with the highest record reported at 3700 m at Pico Orizaba in Mexico (*Stenamma manni* Wheeler). *Stenamma* specieshave been collected mostly in lowland rainforest, montane wet forest, cloud forest, and dwarf forest. A few species (*Stenamma huachucanum*, *Stenamma lagunum*, *Stenamma manni*) also have been found in seasonal woodland habitats in drier areas of Mexico and the southwestern United States. *Stenamma* has never been collected in tropical dry forest or scrub environments. One hypothesis for the nearly complete geographic separation of the HOC and MAC is the presence of extensive xeric environments in northern Mexico and the southwestern United States (Branstetter 2012).

An exceptional characteristic of *Stenamma* is that many species seem to be well adapted to cool, wet environments. Although present in the lowlands, the diversity and abundance of *Stenamma* species peak at mid-elevations between 800–1600 m (Branstetter unpublished data). Also, it has been found that *Stenamma* can be the most common ant genus in leaf-litter samples collected from very wet and cool, cloud forest localities. These ecological traits are in contrast to the pattern seen in ants generally, in which diversity and abundance decrease with elevation. Biogeographic results indicate that *Stenamma* originated in the Nearctic, potentially preadapting it to thrive in cool montane forest environments (Branstetter 2012).

Most *Stenamma* species have very cryptic habits. Nests are usually small, and workers are slow moving, often becoming immobile upon disturbance. Consequently, *Stenamma* is rarely found by the casual observer and most collections are made by sifting leaf-litter from the forest floor. This fact has given *Stenamma* its stereotype as a “leaf-litter ant genus.” Although nests of many species do occur in the leaf litter, and foragers are common there, recent collecting efforts have revealed that MAC *Stenamma* species nest in a variety of microhabitats. I have found nests in large logs, in small rotting branches, in and under bark, in steep clay or mud banks, in and under epiphytes, under rocks, in the ground, and under leaves in leaf litter. At least a few *Stenamma* species nest arboreally. For example, several variants of *Stenamma schmidti* Menozzi have been found reliably underneath epiphyte clumps in the canopy and by canopy fogging, and *Stenamma longinoi* sp. n. is known only from one collection under epiphytes in a treefall. Based on circumstantial evidence, I surmise that a few other species have arboreal nests or at least forage arboreally. *Stenamma stictosomum* sp. n. and *Stenamma felixi* Mann have been found in epiphytic orchids inspected at quarantine in the U.S., and *Stenamma callipygium* sp. n. has been collected most often by beating vegetation.

One of the most intriguing recent discoveries has been the observation that some *Stenamma* species nest in clay banks. Longino (2005) documented in detail the complex nesting behaviors of *Stenamma alas* Longino and *Stenamma expolitum* Smith. Both of these species nest in clay banks in wet forest habitats often along streams, and they exhibit a unique set of behaviors that are thought to be used in evading predation by other ants (possibly army ants specifically). These species construct multiple nests, but only occupy one with brood and a queen; they build each nest with a vertical (*Stenamma expolitum*) or horizontal (*Stenamma alas*) turret sunk into a shallow alcove; and they maintain a small clay “door-pebble,” which is used to block the nest entrance upon encounter with the appropriate disturbance. Reported here for the first time, several other *Stenamma* species are now known to nest in clay banks. I have found *Stenamma diversum* Mann, *Stenamma megamanni* sp. n., *Stenamma muralla* sp. n., and *Stenamma pelophilum* sp. n. all nesting in clay banks along streams or on steep clay slopes. *Stenamma diversum* is particularly interesting, because it has convergently evolved the same nest architecture as *Stenamma alas* (Branstetter pers. obs.). It nests exclusively in steep clay substrate, and builds a nest entrance with an ear-like turret sunk into the clay. It does not, however, appear to build multiple nests per colony or maintain a door-pebble. Based on where I have collected workers and how the workers are sculptured, I believe other *Stenamma* species nest in clay banks as well (e.g. *Stenamma llama* sp. n., *Stenamma lobinodus* sp. n.). Why does *Stenamma* nest in clay banks? I hypothesize that like cloud forests, the clay bank habitat is less hospitable to the average ant, and thus provides *Stenamma* species with a more protected and less competitive environment in which to nest and forage.

*Stenamma* colonies tend to be small and simple, but there is considerable variation in size and structure. *Stenamma diversum*, for example, has very small colonies. Nests consist of a single chamber and have at most a dozen workers, a few alates, brood, and a single dealate queen. In contrast, colonies of *Stenamma manni* and *Stenamma megamanni* are very large. I have found mature colonies of both species in logs and in the ground (usually underneath a log or rock). They tend to have many chambers containing several hundred to perhaps a thousand workers, along with brood and alates (a thorough census has not been performed). *Stenamma alas* and *Stenamma expolitum* have colonies of intermediate size, with up to 200 workers, 50 alates, brood, and a single dealate queen (Longino 2005). Data from nest collections suggest so far that all *Stenamma* species are monogynous, and that most produce winged queens and males. It is unknown how often alates are released and how far they are able to disperse.

## Materials and methods

### Analysis of morphology

Morphological observations were performed at up to 63x magnification using a Leica MZ12.5 stereomicroscope. Measurements were made with Syncroscopy Auto-Montage software by using the measuring tool (calibrated with a stage micrometer) on single images taken at 70× magnification with a JVC KY-F75U digital camera attached to a Leica MZ16A stereomicroscope. Color montage images were created using the same equipment setup used for measurements, except stacks of images were combined with the program Zerene Stacker (http://zerenesystems.com/cms/home) rather than Auto-Montage. Adobe Photoshop and Illustrator, both CS5, were used to enhance images and create Figures.

### Data management

Collection and specimen data for all material examined in this study, along with all color images, have been uploaded to AntWeb (http://www.antweb.org), a site hosted by the California Academy of Sciences. AntWeb subsequently provides all specimen-level data, images, and natural history content to the Global Biodiversity Information Facility (http://www.GBIF.org), the Encylopedia of Life (http://www.EOL.org), and Wikipedia (http://www.Wikipedia.org). The most important linking fields for specimen data are the collection and specimen codes.

Collection codes link specimens to collection and locality information. They are essentially “lot numbers,” meaning they apply to many specimens, and should not be confused with specimen codes. When collections are from individual collectors these codes usually are formed by the collector’s initials followed by a number, e.g. MGB1471 for a personal collection. Collections from the ALAS or LLAMA projects have longer codes, in which each part of the code contains information specific to the project, e.g. Wa-B-03-1-32 for a LLAMA collection. All data from the ALAS and LLAMA projects are available from their respective websites (addresses given above). If labels lacked a collection code, then a generic ANTC# code was added to the pin.

Specimen codes, also referred to as “unique specimen identifiers,” were attached to all pins examined in this study. It is usually intended that these codes refer to single specimens only, but some pins studied here included multiple mounted specimens. In these cases, rather than add multiple specimen codes to a single pin, or remove specimens from a pin, I treat the specimen code as a pin code and add text following the code to unambiguously identify which specimen is being referenced (e.g. top specimen).

Specimen and collection data were transcribed primarily from an electronic database and therefore may not exactly match label information. All distances and elevations are provided in metric units, converted from feet or miles when necessary. All latitude and longitude coordinates are provided in decimal degrees, with up to five decimal places, depending on the precision of the measurement. In cases where coordinates were not present on a label, these were estimated using Google Earth (http://www.google.com/earth/index.html), the GEOnet Names Server (http://earth-info.nga.mil/gns/html/index.html), the FMNH bulk sample database (http://emuweb.fieldmuseum.org/arthropod/bulksamples.php), and/or data already available on AntWeb. Estimated coordinates are indicated with brackets and often include an error estimate on AntWeb. Data for type material follow the format: [Country], [First Administrative District], [Locality], [GPS Coordinates ±Error], [Elevation], [Collection Date], [Habitat], [Microhabitat], [Collector and Collection Code], [Repository and Specimen Code].

The material examined section of each species account presents an overview of a species distribution (i.e. map data). It is not an exhaustive list of every specimen examined for a particular species. In general it includes one collection from every site where a species has been collected, with each site separated by at least 2km from other sites. In some cases a series of very close records were included to capture elevational range information at a particular site. The format for this section is the same as for type material, except without the [Error], [Habitat], [Microhabitat], [Collection Code], [Repository], or [Specimen Code] fields. Complete data for all examined specimens are available on AntWeb.

Maps for each species were generated using the software ArcGIS v10.1 (Esri, Redlands, CA). The mountainous basemap used in all maps was accessed within ArcMap, but is attributed to the U.S. National Park Service.

### Species delimitation

The underlying philosophy driving the separation of species in this study is that of the biological species concept, in which good species represent reproductively isolated entities consisting of one to many populations connected by gene flow (Coyne and Orr 2004). Evidence for reproductive isolation came from finding morphological and/or genetic discontinuities that are maintained in sympatry among closely related forms. The genetic data used here come from Branstetter (2012), as well as from a growing unpublished dataset that includes samples from multiple populations for many of the variable species.

**Figure 2.**
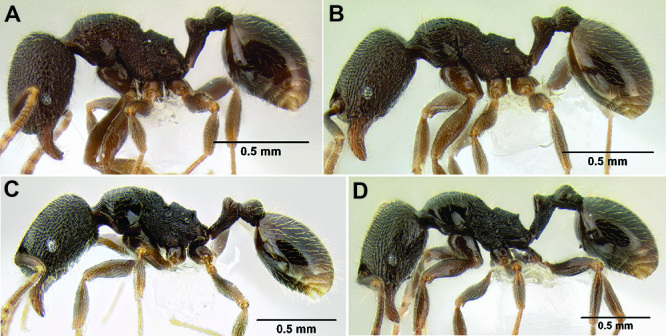
Morphological variation in *Stenamma connectum* along an elevational gradient **A** 990 m (CASENT0605586) **B** 1370 m (CASENT0605496) **C** 1770 m (CASENT06005461) **D** 1990 m (CASENT0605552).

*Stenamma* taxonomy is complicated by several phenomena. First, species are almost always represented by morphologically distinct, allopatric populations, in which intrapopulation variation is less than interpopulation variation. If one were to split all of these variants into species, there would be two to three times more MAC species than included here. Second, many species show exceptional variation along elevational gradients (Figure 2). Usually, populations at lower elevations are smaller and more heavily sculptured, while populations at higher elevations increase in size, become darker in color, and have smooth shiny sculpture. If one compares only specimens from low and high elevations they often appear exceptionally divergent, but I have found that it is common for specimens from intervening elevations to have intermediate phenotypes. Consequently, without having thorough geographic coverage, delimiting *Stenamma* species is a rather arbitrary task. In general, I lump morphological variants into widespread polytypic species if intermediate forms are present and the genetic data are ambiguous, and I split variants into species if they seem “sufficiently” divergent and occur at multiple sites. All species described here should be treated as hypotheses to be tested once more data become available. My reasoning for species delimitation decisions is provided in the comments section of each species account.

### Species names

All new species names in this paper should be treated as nouns in apposition and thus invariant. This holds true even if the derivation of a name suggests otherwise.

### Measurements and indices

**Figure 3.**
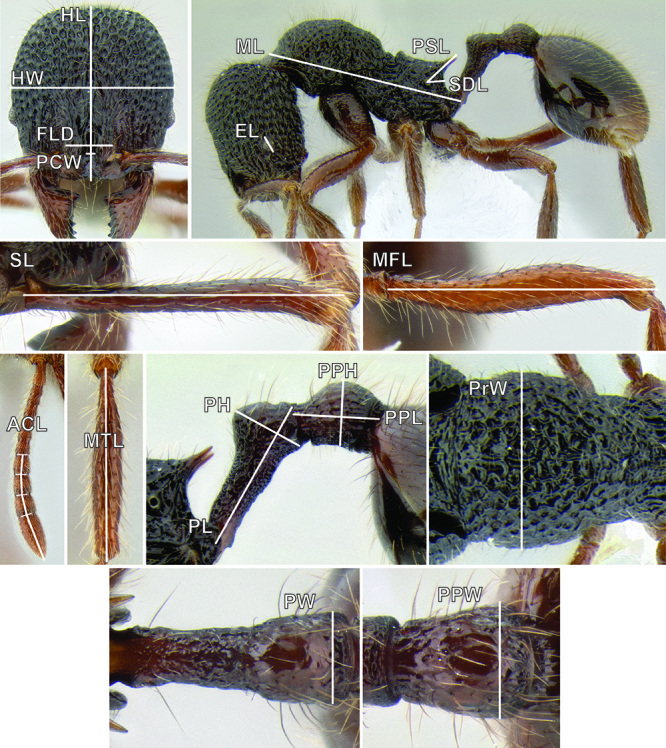
Standard measurements used in this study.

All measurements presented as a range of values in mm, with the holotype specimen's measurements in parentheses unless otherwise stated (see Figure 3).

**HL** Head length (full-face view): maximum length of the head, measured from the posterior margin of the head to the anterior-most extremity of the clypeus.

**HW** Head width (full-face view): maximum width of the head, eyes excluded.

**FLD** Frontal lobes distance (full-face view): the maximum distance separating the outer margins of the frontal lobes.

**PCW** Posterior clypeus width (full-face view): width of the posterior extension of the clypeus (depressed area between the frontal lobes), measured at the midpoint of the antennal sockets.

**SL** Scape length (most suitable view): length of the first antennal segment, exclusive of the basal condyle.

**EL** Eye length (most suitable view): the maximum diameter of the compound eye, including the outer ring of ommatidia, which are often black.

**ACL** Antennal club length (most suitable view): the combined length of antennal segments 9-12, each measured individually and then summed together.

**ML** Mesosoma length (profile view): greatest distance from the approximate inflection point, where the pronotum curves into the cervical shield, to the posterior apex of the propodeal lobes.

**PrW** Pronotum width (dorsal view): maximum width of the pronotum.

**PSL** Propodeal spine length (profile view): distance from the center of the propodeal spiracle to the tip of the propodeal spine.

**SDL** Spiracle to declivity length (profile view): minimum distance from the center of the propodeal spiracle to the propodeal declivity.

**PL** Petiole length (profile view): maximum length of the petiole, measured from the narrowest point of the anterior constriction, to the posterior margin; because the propodeal lobes usually obscure the anterior constriction in lateral view, this point must be approximated.

**PH** Petiole height (profile view): maximum height of the petiole, measured at a right angle to PL and taken from the dorsal surface of the petiolar node to the ventral surface of the postpetiolar helcium, where it inserts into the petiole.

**PW** Petiole width (dorsal view): maximum width of the petiole.

**PPL** Postpetiole length (profile view): maximum length of the postpetiole, measured from the posterior margin of the enlarged portion of the helcium, to the posterior margin of the postpetiole.

**PPH** Postpetiole height (profile view): height of the postpetiole, measured in a line perpendicular to PPL. If the ventral surface is concave upward, the measurement should be taken from the uppermost portion of the curve.

**PPW** Postpetiole width (dorsal view): maximum width of the postpetiole.

**MFL** Metafemur length (most suitable view): maximum length of the metafemur, measured from the distal margin of the trochanter to the metafemur apex. In most cases this measurement was taken from the anterior side of the metafemur.

**MTL** Metatibia length (most suitable view): maximum distance of the metatibia, measured from the proximal constriction, just before the inserting condyle, to the apex. This measurement is always performed on the dorsal surface of the metatibia so that the proximal condyle is not obscured by the metafemur.

**CI** Cephalic index: HW/HL × 100.

**SI** Scape index: SL/HW × 100.

**REL** Relative eye length: EL/HW × 100.

**FLI** Frontal lobes index: FLD/HW × 100.

**PSI** Propodeal spine index: PSL/SDL.

**MFI** Metafemur index: HW/MFL × 100.

**ACI1** Antennal club index: segments 11+12/ACL × 100.

**ACI2** Antennal club index: ACL/SL × 100.

### Specimen repositories

**CAS** California Academy of Sciences, San Francisco, CA, USA.

**EAPZ** Escuela Agricola Panamericana Zamorano, Tegucigalpa, Honduras.

**ECOSCE** Colección Entomológica de El Colegio de la Frontera Sur, Unidad San Cristóbal, Chiapas, Mexico.

**FMNH** Field Museum of Natural History, Chicago, IL, USA.

**ICN** Insect Collection, Instituto de Ciencias Naturales, Universidad Nacional de Colombia, Bogotá D.C., Colombia.

**INBio** Instituto Nacional de Biodiversidad, Costa Rica.

**JTLC** John T. Longino, personal collection, University of Utah, Salt Lake City, Utah, USA.

**LACM** Los Angeles County Museum of Natural History, Los Angeles, CA, USA.

**MCZ** Museum of Comparative Zoology, Harvard University, Cambridge, MA, USA.

**MGBPC** Michael G. Branstetter, personal collection.

**MZSP** Museu de Zoologia da Universidade de São Paulo, São Paulo, Brazil.

**NHMB** Naturhistorisches Museum, Basel, Switzerland.

**PSWC** Philip S. Ward, personal collection, University of California, Davis, CA, USA.

**UCD** Bohart Museum of Entomology, University of California, Davis, CA, USA.

**UNAM** Universidad Nacional Autonoma de Mexico, Mexico D.F., Mexico.

**USNM** National Museum of Natural History, Washington, D.C., USA.

**UVGC** Colección de Artrópodos, Universidad del Valle de Guatemala, Guatemala City, Guatemala.

### Characters

Several characters useful in distinguishing *Stenamma* species are discussed below. It should be noted that almost every character type is variable within species and prone to some level of homoplasy among species. Thus, it is difficult to assess which characters are “better” than others for separating species. In MAC *Stenamma* any variable character can be useful in some cases, but misleading in others. All characters used previously to separate HOC *Stenamma* species tend to be more variable among MAC species. New characters for distinguishing species include the structure of the basal margin of the mandible, configuration of gastral pilosity, frontal lobe width, promesonotum shape, and propodeal spine length.

*Body size*. Species of *Stenamma* are relatively small compared to most other ant genera, but within the genus there is exceptional variability among species, within species, and, in some cases, within single colonies. Even though within-species and within-colony size variation can be substantial, size is still useful as a character in species separation. Using HL, ML, and PrW as proxies for overall size I assign species to the qualitative categories small (HL ≤ 0.73, ML ≤ 0.92, PrW ≤ 0.45), medium (HL 0.74–0.96, ML 0.93–1.27, PrW 0.46–0.62), and large (HL ≥ 0.97, ML ≥ 1.28, PrW ≥ 0.62), with intermediate states possible. One phenomenon that has not been reported previously in *Stenamma* is the existence of substantial within-colony size variation. For medium- to large-sized species, I have noticed substantial size variation from the smallest nanitics to the largest workers. In some cases, the largest workers have allometrically enlarged heads, making them appear almost soldier-like. In all cases the size variation appears to be continuous rather than discrete.

**Figure 4.**
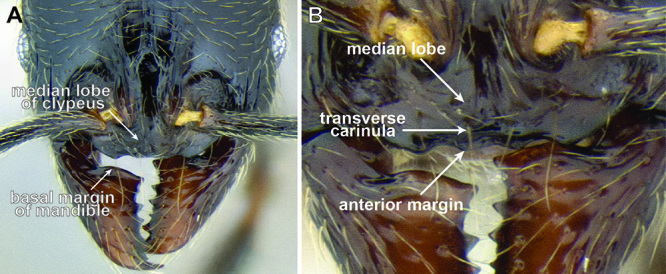
Clypeus structure in *Stenamma maximon* (CASENT0604875) **A** Clypeus in full-face view **B** Clypeus in anterodorsal view.

*Clypeus & mandible structure*. Snelling’s (1973) revision of western U.S. *Stenamma* species described the utility of clypeus shape for distinguishing species. As suggested in Branstetter (2009), this character, along with the shape of the basal margin of the mandible, is also very useful in separating Neotropical species. The clypeus has two variable features, the median lobe and the anterior margin (Figure 4). The median lobe can be flattened and nearly absent, or it can be more prominent, often projecting anteriorly over the anterior clypeal margin in full-face view. In the latter case, it is necessary to view the clypeus from an anterodorsal angle in order to see the underlying clypeal margin. The median lobe can be completely smooth or have a pair of faint to somewhat distinct longitudinal carinulae. There is usually a short transverse carinula at the apex of the lobe.

The anterior clypeal margin varies greatly (see Figure 19). It can be entire; have a shallow median emargination; have a deep median excision; have a slight undulation; have two to four sharp to blunt teeth; or have a deep, irregular median depression. It also varies in orientation, usually projecting anteriorly, but sometimes projecting more ventrally, making it difficult to view.

The structure of the basal margin of the mandible usually correlates with the shape of the anterior clypeal margin (see Figure 19). This is because clypeal teeth, if present, usually merge with the basal margin of the mandible when the mandibles are closed. When the anterior clypeal margin is entire or only has a median emargination, the basal margin of the mandible is straight to slightly sinuous. When the anterior clypeal margin is undulating or forming teeth, the basal margin of the mandible is sinuous and usually has a distinct, shallow to deep basal depression. In some species, the basal depression is accompanied by a small tooth that projects inward from the proximal edge of the depression.

The clypeus and basal margin of the mandible are very useful in species identification. Consequently, it is highly recommended that specimens be mounted with mandibles open. If all specimens from a series have the mandibles closed, then the mandibles should be carefully pried open (before mounting) by pushing a pin between the mandibles from the ventral side of the head.

*Color*. Overall body and appendage color and the contrast between the two can be very useful in separating some species. Body color ranges from jet black to pale yellow, with red-black, brown-black, orange-brown, or brown as common intermediate colors. Sometimes species have a mottled appearance, with areas of dark or light cuticle occurring irregularly. Appendages are almost always lighter in color, usually becoming very light toward the extremities. It is important to note that older museum specimens lose their color, often having a faded red-brown color (see Figures 73, 82, 85, 150).

**Figure 5.**
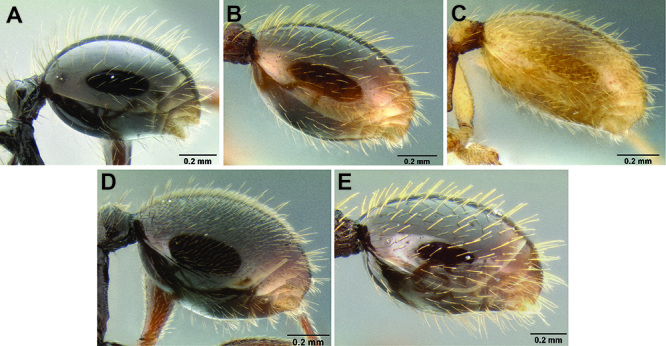
Comparison of gastral pilosity among several *Stenamma* species **A** *Stenamma diversum* (CASENT0606723) **B** *Stenamma monstrosum* (CASENT0621327) **C** *Stenamma lagunum* (CASENT0622371) **D** *Stenamma schmidti* (INB0003665417) **E** *Stenamma longinoi* (JTLC000007475).

*Gastral pilosity*. On a given Stenamma specimen, pilosity on the body dorsum is generally similar in form, but it is usually best viewed on the gastral tergites. Among species, pilosity varies in length, density, thickness, and whether or not it is separated into two distinct layers (Figure 5). There is a continuum from being long, sparse, and clearly single layered, to being short, dense, and clearly bilayered, with the bilayered pilosity consisting of a longer layer of suberect to subdecumbent setae and a shorter layer of subdecumbent to appressed setae. The suberect, upper layer of setae can sometimes be distinctly thickened (i.e. stout), and the lower layer can be either very sparse or very dense and thin, becoming pubescent. Using this character is complicated by the fact that some species have intermediate character states and some species show substantial intraspecific variation.

**Figure 6.**
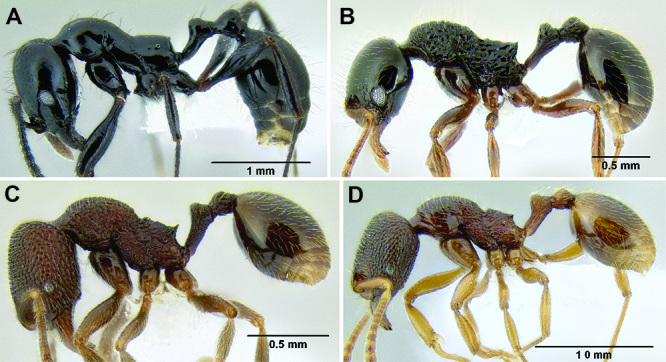
Comparison of several different sculpture patterns in *Stenamma* **A** *Stenamma atribellum* (CASENT0622351) **B** *Stenamma tico* (CASENT0622571) **C** *Stenamma catracho* (CASENT0621306) **D** *Stenamma ochrocnemis* (CASENT0603793).

*Sculpture*. In principal I adhere to the terminology and philosophy of Harris (1979). Surface sculpture varies greatly among MAC *Stenamma* species and is a very good separatory character. There are three main patterns of sculpturing: entire body smooth and shiny; face smooth and shiny, but mesosoma mostly sculptured; and head and mesosoma completely sculptured. It is also common for the pronotum to become smooth and shiny, whereas the remainder of the body is sculptured (Figure 6). The gaster is usually always smooth and shiny except for a few species that have variably developed basal punctae. Sculpture types include carinae, rugae, rugoreticulae, costae, punctae, and foveae, as well as the diminutive forms of all types. I often use the term punctae in a broad sense to refer to what might also be described as punctulae or foveolae. Species that occur over a broad elevational range usually have variable sculpturing, with specimens from higher elevations becoming smoother and shinier. Species that vary substantially in this way are more difficult to characterize.

**Figure 7.**
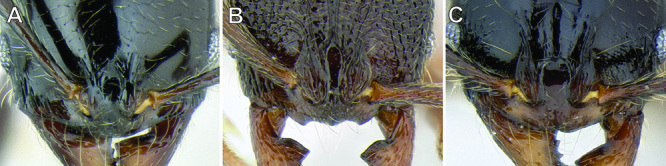
Comparison of the frontal lobes and posterior extension of the clypeus in several *Stenamma* species **A** *Stenamma llama* (CASENT0605479) **B** *Stenamma cusuco* (CASENT0622137) **C** *Stenamma diversum* (CASENT0606723).

*Frontal lobes and posterior extension of the clypeus*. There is significant variation in the width of the frontal lobes and the width of the depressed area in between the lobes (Figure 7). In some species the frontal lobes are strongly expanded laterally or dorsolaterally. This variation is characterized quantitatively with the measurement FLD and the index FLI. It is also described qualitatively by whether or not the underlying torular lobes are covered by the frontal lobes in full-face view. The area in between the frontal lobes, referred to here as the posterior extension of the clypeus, can be very narrow to quite wide, with the sides varying in shape from subparallel to strongly hour-glass shaped. An attempt to quantify this variation is made with the measurement PCW. Both of these characters are useful, but most species display intermediate values.

**Figure 8.**
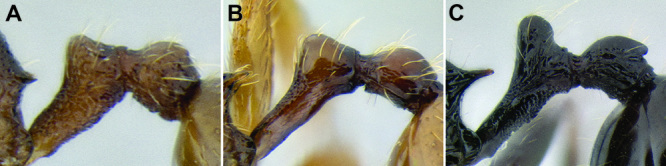
Comparison of petiole and postpetiole shape in several *Stenamma* species **A** *Stenamma crypticum* (CASENT0603755) **B** *Stenamma hojarasca* (CASENT0622132) **C** *Stenamma lobinodus* (CASENT0605658).

*Petiole and postpetiole shape*. Many *Stenamma* species have very distinctive petiole and postpetiole shapes (Figure 8). Categories of qualitative descriptors include overall robustness, symmetry of the nodes (in profile), and sharpness and angle of node apices. I also attempt to capture variation quantitatively with length, width, and height measurements of each segment. Often these are most useful as ratios. For example, if the postpetiole is very wide in relation to the petiolar node, I include an index such as PPW/PW. The actual index used depends on the distinctive feature for a particular species.

**Figure 9.**
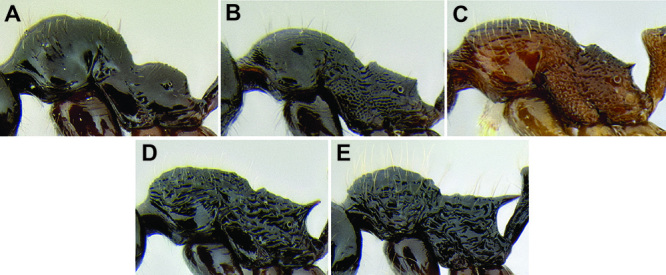
Comparison of promesonotum and propodeal spines in several *Stenamma* species **A** *Stenamma alas* (CASENT0606832) **B** *Stenamma muralla* (CASENT0621311) **C** *Stenamma crypticum* (CASENT0603755) **D** *Stenamma lobinodus* (CASENT0605658) **E** *Stenamma diversum* (CASENT0606723).

*Promesonotum shape*. The shape of the promesonotum in profile is variable and can be very distinctive in some species (Figure 9). The average form is for it to be low-domed and roughly symmetrical from front to back, but it can become distinctly asymmetrical, with the apex shifted away from the midpoint, or with the anterior or posterior slope much longer than the other. It can also be distinctly bulging, becoming high-domed.

*Propodeal spines*. The propodeal spines in *Stenamma* can be absent to quite long and robust (Figure 9). To quantify this character I use the measurement PSL and the index PSI. I also describe them as absent, tuberculate, short, medium, or long. When absent, the juncture of the propodeal dorsum and declivity can be smoothly rounded or it can form a sharp angle.

### Discussion of Middle American clade (MAC)

The MAC is distributed from the southwestern U.S.A. (Arizona, New Mexico, Texas) to northwestern South America (Colombia, Ecuador) (Figure 1). It is known to co-occur with the Holarctic clade only in the southwestern U.S.A, where *Stenamma huachucanum* reaches the northern limit of its range. In this area of sympatry it is easy to separate the two clades based on the structure of the anterior clypeal margin and basal margin of the mandible (Branstetter 2012). In *Stenamma huachucanum* the anterior clypeal margin forms four teeth, and the basal margin of the mandible is sinuous and has a slight basal depression. In contrast, the HOC species have the anterior clypeal margin with a simple median emargination (can be difficult to see in *impar* group species) and the basal margin of the mandible straight. The HOC species also tend to have very conspicuous longitudinal clypeal carinulae, whereas these are reduced in *Stenamma huachucanum* and most MAC species.

Using just the worker caste it has not been possible to find diagnostic features that adequately distinguish the MAC from the HOC. In general, MAC species exhibit a much greater diversity of phenotypes, resulting in an abundance of autapomorphies. The following features help define the MAC: clypeal carinae usually faint or absent (usually more distinct in HOC); structure of the anterior clypeal margin more variable than in HOC, often forming distinct teeth (usually a simple median emargination in HOC); structure of the basal margin of the mandible more variable, often sinuous, with a distinct basal depression or notch (usually straight in HOC); surface sculpture often reduced, sometimes completely smooth and shiny (HOC species usually completely sculptured); form of pilosity on gastral dorsum more diverse (HOC species usually with pilosity short, bilayered, and not noticeably thickened); gastral tergites and sternites usually smooth and shiny (only sometimes punctate in *Stenamma huachucanum* and *Stenamma manni*, and with short basal carinulae in *Stenamma atribellum* sp. n. and *Stenamma callipygium*; HOC species commonly with basal carinulae and punctae).

A global diagnosis of the worker caste of *Stenamma* is presented in Branstetter (2009), with some additional notes comparing MAC and HOC *Stenamma* species. Within the Neotropics, *Stenamma* is likely to be confused only with the myrmicine genera *Adelomyrmex* Emery, *Aphaenogaster* Mayr, *Lachnomyrmex* Wheeler, *Megalomyrmex* Forel, *Pheidole* Westwood, or *Rogeria* Emery. *Stenamma* can be separated from these genera by having the following combination of characters: antenna 12-segmented; flagellum with a distinct to indistinct 4-segmented club; propodeum in profile depressed compared to promesonotum; posterior extension of the clypeus between antennal insertions relatively narrow and long (only broad in a few species).

### Discussion of species groups

Using worker morphology and results from molecular phylogenetic analysis (Branstetter 2012, unpublished data), an attempt was made to create morphologically diagnosable, monophyletic species groups within MAC *Stenamma*. However, for most well-supported clades within the MAC, this was not possible due to significant morphological variability and homoplasy among species. The few exceptions to this are the following: (I) *atribellum* group (*Stenamma atribellum*, *Stenamma callipygium*), (II) *diversum* group (*Stenamma diversum*, *Stenamma tico* sp. n.), (III) *expolitum* group (*Stenamma alas*, *Stenamma expolitico* sp. n., *Stenamma expolitum*), and (IV) *lobinodus* group (*Stenamma llama*, *Stenamma lobinodus*, *Stenamma tiburon* sp. n.). If species complexes become better resolved and the reproductive castes are studied in more detail, additional species groups may be identified. To simplify the organization of this revision, the species accounts below are ordered alphabetically by species, rather than by species group. Diagnostic character states for each group can be found in the key and in the individual species accounts.

### Synopsis of MAC *Stenamma* species

List includes 40 species, 33 of which are new. See Supplementary file for a table that links the species names presented here with the code names used in Branstetter (2012).

*Stenamma alas* Longino 2005. Costa Rica to Ecuador.

*Stenamma andersoni* Branstetter sp. n. Southern Mexico.

*Stenamma atribellum* Branstetter sp. n. Honduras.

*Stenamma brujita* Branstetter sp. n. Mexico to Honduras.

*Stenamma callipygium* Branstetter sp. n. Guatemala.

*Stenamma catracho* Branstetter sp. n. Honduras.

*Stenamma connectum* Branstetter sp. n. Southern Mexico.

*Stenamma crypticum* Branstetter sp. n. Southern Mexico to Nicaragua.

*Stenamma cusuco* Branstetter sp. n. Honduras.

*Stenamma diversum* Mann 1922. Southern Mexico to Nicaragua.

*Stenamma excisum* Branstetter sp. n. Mexico to Honduras.

*Stenamma expolitico* Branstetter sp. n. Costa Rica.

*Stenamma expolitum* Smith 1962. Nicaragua to Costa Rica.

*Stenamma felixi* Mann 1922. Mexico to Ecuador.

*Stenamma hojarasca* Branstetter sp. n. Southern Mexico to Honduras.

*Stenamma huachucanum* Smith 1957. Southwestern U.S.A. to southern Mexico.

*Stenamma ignotum* Branstetter sp. n. Southern Mexico to Guatemala.

*Stenamma lagunum* Branstetter sp. n. Northern Mexico.

*Stenamma leptospinum* Branstetter sp. n. Southern Mexico.

*Stenamma llama* Branstetter sp. n. Southern Mexico to Guatemala.

*Stenamma lobinodus* Branstetter sp. n. Mexico.

*Stenamma longinoi* Branstetter sp. n. Southern Mexico.

*Stenamma manni* Wheeler 1914. Mexico to Nicaragua.

*Stenamma maximon* Branstetter sp. n. Southern Mexico to Honduras.

*Stenamma megamanni* Branstetter sp. n. Southern Mexico to Nicaragua.

*Stenamma monstrosum* Branstetter sp. n. Honduras to Nicaragua.

*Stenamma muralla* Branstetter sp. n. Honduras to Nicaragua.

*Stenamma nanozoi* sp. n. Honduras.

*Stenamma nonotch* Branstetter sp. n. Southern Mexico to Guatemala.

*Stenamma ochrocnemis* Branstetter sp. n. Southern Mexico to Honduras.

*Stenamma pelophilum* Branstetter sp. n. Mexico to Honduras.

*Stenamma picopicucha* Branstetter sp. n. Honduras to Nicaragua.

*Stenamma saenzae* Branstetter sp. n. Southern Mexico to Honduras.

*Stenamma sandinista* sp. n. Nicaragua.

*Stenamma schmidti* Menozzi 1931a. Nicaragua to Ecuador.

*Stenamma stictosomum* Branstetter sp. n. Mexico to Honduras.

*Stenamma tiburon* Branstetter sp. n. Northern Mexico.

*Stenamma tico* Branstetter sp. n. Nicaragua to Panama.

*Stenamma vexator* Branstetter sp. n. Mexico.

*Stenamma zelum* Branstetter sp. n. Honduras to Panama.

### Key to species based on worker caste

**Table:** 

1	Anterior constriction of gaster, along with basal striae, distinctly elongate (Figure 10A, B)	2
–	Anterior constriction of gaster and basal striae not elongate (Figure 10C)	3
2 (1)	Surface sculpture almost entirely smooth and shiny (Figure 11A); anterior clypeal margin in full-face view with a shallow median emargination (Figure 11A) (Honduras)	*Stenamma atribellum* sp. n.
–	Face and much of mesosoma with conspicuous carinulae (Figure 11B); median lobe of clypeus projecting over anterior clypeal margin, forming a well-defined median apex (Figure 11B) (Guatemala)	*Stenamma callipygium* sp. n.
3 (1)	Anterior clypeal margin in full-face view forming three well-defined teeth (visible even if mandibles closed), with middle tooth formed by median lobe, which projects over true clypeal margin (Figure 12A); frontal lobes markedly expanded outward, completely obscuring torular lobes in full-face view (Figure 12A) (FLD 0.24–0.26, FLI 36–39); head and mesosoma densely sculptured with rugoreticulae and punctae; propodeal spines well-developed (Figure 12B) (SSL 0.16–0.19, PSI 1.8–2.2) (Honduras)	*Stenamma cusuco* sp. n.
–	Anterior clypeal margin in full-face view variable, not as above (Figure 19A–I); frontal lobes, body sculpture, and propodeal spines variable	4
4 (3)	Face and promesonotal dorsum foveate to coarsely rugoreticulate (Figure 13A, B); pilosity on gastral tergites long, dense, and mostly suberect (Figure 13C); eyes relatively small (EL 0.09–0.13, REL 10–16), with 5–8 ommatidia at greatest diameter; 4-segmented antennal club indistinct; larger species (HL 0.87–1.20, HW 0.78–1.15, ML 1.15–1.62)	5
–	Sculpture variable, but not distinctly foveate; other characters variable, not as above	6
5 (4)	Anterior clypeal margin forming four relatively sharp teeth (middle teeth only visible if mandibles open) (Figure 14A); propodeal spines reduced to sharp angles or small tubercles (Figure 14D) (SSL 0.12–0.16, PSI 1.0–1.3); somewhat smaller species (HL 0.87–1.03, HW 0.78–0.92, ML 1.15–1.38) (eastern Honduras to Panama)	*Stenamma zelum* sp. n.
–	Anterior clypeal margin with a median emargination that has four well-defined to completely effaced blunt teeth (Figure 14B, C); propodeal spines usually present, ranging from short tubercles to long robust spines (Figure 14E–G) (SSL 0.15–0.37, PSI 1.3–2.9); somewhat larger species (HL 0.90–1.20, HW 0.77–1.15, ML 1.15–1.62) (Mexico to western Honduras)	*Stenamma brujita* sp. n.
6 (4)	Lateral apex of hypostomal bridge projecting ventrally as a subquadrate (Figure 15A, B) to broadly rounded (Figure 15C) lobe, visible behind base of mandible in profile view	7
–	Lateral margin of hypostomal bridge tapering to a narrow point, not visible in profile view (Figure 15D–F)	11
7 (6)	Propodeal spines long and slender (SSL 0.17–0.22, PSI 1.9–2.2) (Figure 16A); face with a dense fan of carinulae extending out from frontal lobes toward posterior and lateral margins of head (Figure 16B); postpetiole appearing somewhat anteroposteriorly compressed (Figure 16A) (southern Mexico)	*Stenamma leptospinum* sp. n. (part)
–	Propodeal spines tuberculate or short; face sculpture and petiole variable, not as above	8
8 (7)	Pronotum conspicuously punctate, with longitudinal rugulae faintly present among punctae on dorsum (Figure 17A, B); suberect setae on gastral tergites very stout, somewhat sparse, short (Figure 17C); propodeal spines present, short (SSL 0.16–0.19, PSI 1.4–1.6) (southern Mexico)	*Stenamma longinoi* sp. n.
–	Lacking one or more of the above character states	9
9 (8)	Petiole and postpetiole more slender, with postpetiole in profile appearing particularly small and elongate (Figure 18A) (PPH/PH 0.74–0.84, PW/PPW 0.76–0.89); pronotum usually mostly smooth and shiny, with only some vestigial rugulae (Figure 18A); smaller species (HL 0.72–0.83, HW 0.63–0.75, ML 0.89–1.08) (Honduras to Nicaragua)	*Stenamma muralla* sp. n.
–	Waist segments more robust, with postpetiole in profile sometimes bulging and usually more circular (Figure 18B–D) (PPH/PH ≥ 0.79, PW/PPW ≥ 0.87); pronotum sculpture variable, but usually more dense; larger species (HL ≥ 0.81, HW ≥ 0.70, ML ≥ 1.05) (Mexico to Nicaragua)	10
10 (9)	Eye usually smaller (EL 0.10–0.16), with 5–8 ommatidia at greatest diameter; body color usually lighter, dark red-brown to brown, but sometimes orange-brown or yellow-brown (Figure 18B, C) (Mexico to Nicaragua)	*Stenamma manni* (part)
–	Eye usually larger (EL 0.14–0.19), with 8–11 ommatidia at greatest diameter; body color mostly black (Figure 18D) (southern Mexico to Nicaragua)	*Stenamma megamanni* sp. n.
11 (6)	Basal margin of mandible straight to slightly sinuous, without a distinct basal notch or depression (Figure 19A–C); anterior clypeal margin entire (Figure 19A), or with a rounded median emargination (Figure 19B), or with a deep median excision (Figure 19C) (rarely with mandible completely straight and anterior clypeal margin with 2 blunt teeth)	12
–	Basal margin of mandible sinuous, with a shallow to deep basal depression or notch, but without a basal tooth (Figure 19D–F); anterior clypeal margin undulating, forming 2–4 variably developed teeth (Figure 19D–F) (sometimes teeth very reduced, so clypeal margin is nearly flat)	30
–	Basal margin of mandible sinuous, with a distinct basal depression or notch, and an accompanying small tooth (Figure 19G–I); anterior clypeal margin usually undulating, forming 2–4 variably developed teeth (Figure 19D–I)	41
12 (11)	Propodeal spines absent; petiole and postpetiole almost completely smooth and shiny, with only faint vestigial punctae sometimes present (Figure 20A–D); postpetiole in profile bulging, globular, appearing more voluminous than petiolar node (Figure 20A–D)	(*expolitum* group) 13
–	Propodeal spines present or absent; petiole and postpetiole not as above, either with more conspicuous sculpturing, or with the postpetiole not distinctly larger than petiolar node	15
13 (12)	Face almost completely smooth and shiny, with only some vestigial carinulae near frontal lobes and anterolateral margins of head (Figure 21A); carinulae around frontal lobes extending to about midpoint of head or less; dorsum of promesonotum smooth and shiny (Nicaragua to Costa Rica)	*Stenamma expolitum*
–	Face with more extensive sculpture, consisting of a fan of carinulae that extend from the area around antennal insertions to at least midpoint of head, but sometimes reaching to posterior margin (Figure 21B–D); dorsum of promesonotum smooth and shiny, or with variably developed transverse furrows or striae (Costa Rica to Ecuador)	14
14 (13)	Dorsal and declivitous faces of propodeum in profile flat, and forming a blunt 90° angle (Figure 20D); dorsum of promesonotum with distinctive transverse furrows, sometimes reticulate posteriad (Figure 22C) (Costa Rica)	*Stenamma expolitico* sp. n.
–	Propodeum in profile more rounded, with transition between dorsal and declivitous faces less abrupt (Figure 20B, C); dorsum of promesonotum either smooth and shiny, or with few to many transverse striae (Figure 22B, C) (Costa Rica to Ecuador)	*Stenamma alas*
15 (12)	Face mostly smooth and shiny, except for scattered piligerous punctae and at most a few carinulae, rugulae, and/or punctae around frontal lobes and anterolateral margins of head (Figure 23A, B)	16
–	Face sculpture more extensive, usually completely sculptured; type of sculpture variable (Figure 23C)	21
16 (15)	Dorsum of promesonotum mostly smooth and shiny, except for scattered piligerous punctae, and at most a few longitudinal rugulae (Figure 24A, B)	17
–	Dorsum of promesonotum with more extensive sculpturing, either reticulately costate or rugoreticulate (Figure 24C)	19
17 (16)	Mesosoma compact, with promesonotum somewhat bulging (Figure 25A); propodeal spines present as short robust triangles (Figure 25A) (SSL 0.09–0.14, PSI 1.7–2.5); petiolar node robust, relatively tall (PH/PL 0.66–0.80), and distinctly angled posteriad (Figure 25A); scape and metafemur relatively short (SI 80–85, MFI 101–113) (southern Mexico to Honduras)	*Stenamma llama* sp. n.
–	Lacking one or more of the above character states (Figure 25B–D)	18
18 (17)	Median lobe of clypeus bicarinate, projecting, and with area between carinae distinctly depressed (Figure 26A); promesonotum in profile asymmetrical, with anterior slope long and gently curving, dorsum nearly flat, and posterior slope short and forming a sharp transition with dorsum (Figure 25B); propodeal spines absent (SSL 0.06–0.07, PSI 1.0–1.3) (northern Mexico)	*Stenamma tiburon* sp. n.
–	Median lobe of clypeus smooth and with a simple median emargination (Figure 26B); promesonotum in profile usually asymmetrical (Figure 25C), with posterior slope distinctly longer than anterior slope, but sometimes promesonotum more evenly domed (Figure 25D); propodeal spines tuberculate to short (SSL 0.06–0.10, PSI 1.1–1.7) (Mexico to Honduras)	*Stenamma pelophilum* sp. n.
19 (16)	Promesonotum in profile distinctly asymmetrical, with anterior slope long and gently curving, dorsum nearly flat and at a slight downward angle, and posterior slope short and forming a sharp transition with dorsum (Figure 27A, B); postpetiolar node in profile with a longitudinal dorsal lobe that projects posteriorly over postpetiole (Figure 27A, B) (Mexico)	*Stenamma lobinodus* sp. n.
–	Promesonotum in profile low domed and roughly symmetrical (Figure 27C, D); postpetiole in profile more circular and without a distinct dorsal lobe projecting posteriorly (Figure 27B, C)	20
20 (19)	Propodeal spines present, long (Figure 27C) (SSL 0.28–0.34, PSI 3.0–3.7); frontal lobes expanded dorsolaterally, usually completely covering torular lobes in full-face view (Figure 28A) (FLD 0.25–0.29, FLI 35–38); eye smaller (EL 0.11–0.15, REL 16–20) (southern Mexico to Nicaragua)	*Stenamma diversum*
–	Propodeal spines reduced to sharp right angles, or small upward projecting tubercles (Figure 27D) (SSL 0.14–0.18, PSI 1.4–1.9); frontal lobes narrower, with torular lobes partly visible in full-face view (Figure 28B, C) (FLD 0.22-0.27, FLI 33-36); eye larger and somewhat bulging (EL 0.15-0.18, REL 23–24) (Nicaragua to Panama)	*Stenamma tico* sp. n. (part)
21 (15)	Propodeal spines absent, with the dorsal and declivitous faces of the propodeum forming a relatively shallow, blunt angle in profile view (Figure 29A, B) (SSL 0.08–0.11, PSI 0.8–1.1); eyes large (EL ≥ 0.15, REL ≥ 18)	22
–	Propodeal spines varying from tuberculate to long, or with the dorsal and declivitous faces of the propodeum forming a sharp, steep angle (Figure 29C–E) (SSL ≥ 0.07, PSI ≥ 1.0); eyes variable	23
22 (21)	Head and mesosoma strongly sculptured (Figure 29A, 30A, B); face mostly rugoreticulate or carinate; scape relatively shorter (SI 84–101); eye relatively smaller (REL 18–22); larger, more robust species (HL 0.90–1.19, HW 0.81–1.04, ML 1.25–1.62) (Mexico to Ecuador)	*Stenamma felixi*
–	Head and mesosoma more faintly sculptured (Figure 29B, 30C, D); face with variable amount of carinulae, rugulae, and punctae; scape relatively longer (SI 107–121); smaller, usually somewhat gracile species (HL 0.79–0.88, HW 0.65–0.73, ML 1.04–1.16) (Nicaragua to Ecuador)	*Stenamma schmidti* (part)
23 (21)	Facial sculpture light, largely effaced, usually not extending all the way to posterior margin of head (Figure 31A); mesosoma mostly reticulately costate (Figure 31B); propodeal spines reduced to sharp right angles, or small upward projecting tubercles (Figure 31B) (PSL 0.14–0.18, PSI 1.4–1.9); eye large, somewhat bulging (EL 0.15-0.18, REL 23–24) (Nicaragua to Panama)	*Stenamma tico* sp. n. (part)
–	Lacking one or more of the above character states	24
24 (23)	Smaller species (HL ≤ 0.76, HW ≤ 0.68, ML ≤ 1.00); eye usually smaller (EL ≤ 0.12), with 2–7 ommatidia at greatest diameter (Figure 32A, B); lateral margin of hypostomal bridge tapering to a point, never visible in profile view (Figure 15D–F)	25
–	Larger species (HL ≥ 0.80, HW ≥ 0.70, ML ≥ 1.02); eye usually larger (EL ≥ 0.11), with 5–11 ommatidia at greatest diameter (Figure 32C); lateral margin of hypostomal bridge usually broadly rounded and somewhat projecting, often visible in profile view (Figure 15C)	29
25 (24)	Eye small (EL 0.04–0.08, REL 8–14), subcircular, with 2–5 ommatidia at greatest diameter (Figure 32A)	26
–	Eye larger (EL 0.09–0.12, REL 15–21), more oval-shaped, with 4–7 ommatidia at greatest diameter (Figure 32B)	27
26 (25)	Sculpture on face (and much of mesosoma) consisting of an even distribution of short longitudinal rugulae (Figure 33A); body color a mottled yellow-brown (often pale yellow), with patches of darker brown; median clypeal lobe in full-face view visible and well developed (Figure 33A); anterior clypeal margin with a shallow median emargination (northern Mexico)	*Stenamma lagunum* sp. n.
–	Face densely sculptured and mostly rugoreticulate (Figure 33B, C); mesosoma densely sculptured with punctae, rugae, and/or rugoreticulae; body color usually a darker orange-brown; clypeus in full-face view appearing very short, with median lobe nearly invisible due its dorsoventral orientation (Figure 33B, C); anterior clypeal margin often with a deep median excision (Figure 33B), but sometimes only with a very weak median depression (Figure 33C) (Mexico to Honduras)	*Stenamma excisum* sp. n.
27 (25)	Anterior clypeal margin either entire, or with a nearly imperceptible median notch (Figures 19A, 34A); dorsum of promesonotum either rugoreticulate, or with many irregular rugulae (Figure 34B); petiolar node often broadly rounded and pointing distinctly posteriad (Figure 34C) (southern Mexico to Guatemala)	*Stenamma nonotch* sp. n.
–	Anterior clypeal margin not as above (Figure 25D, G); dorsum of promesonotum with relatively dense longitudinal rugulae/carinulae (Figure 25E, H); petiolar node not as above (Figure 25F, I)	28
28 (27)	Anterior clypeal margin with a shallow, but distinct median emargination (Figure 34D); petiole usually appearing more elongate (Figure 34F); petiolar node usually sharper and pointing more vertically; pilosity on gastral tergites longer and mostly forming a relatively sparse layer of suberect setae, only sometimes with decumbent setae (Figure 34F); metafemur relatively longer (MFI 96–104) (southern Mexico to Honduras)	*Stenamma ignotum* sp. n.
–	Anterior clypeal margin forming 2 small blunt teeth, which straddle the midline (Figure 34G); petiole more compact, with the node less sharp and pointing more strongly posteriad (Figure 34I); pilosity on gastral tergites somewhat shorter and usually distinctly bilayered, with a layer of suberect setae, and a layer of decumbent setae (Figure 34G); metafemur relatively shorter (MFI 104–110) (Honduras to Nicaragua)	*Stenamma picopicucha* sp. n.
29 (24)	Propodeal spines long and slender (Figure 29E) (SSL 0.17–0.22, PSI 1.9–2.2); face with a dense fan of carinulae extending out from frontal lobes toward posterior and lateral margins of head (Figure 35A); postpetiole appearing somewhat anteroposteriorly compressed (Figure 29E) (southern Mexico)	*Stenamma leptospinum* sp. n. (part)
–	Propodeal spines tuberculate to short (Figure 29D) (SSL 0.09–0.19, PSI 1.0–1.6); face sculpture variable, but usually mostly rugoreticulate, and never densely carinulate (Figure 35B, C); postpetiole usually more circular, not anteroposteriorly compressed (Figure 29D) (Mexico to Nicaragua)	*Stenamma manni* (part)
30 (11)	Posterior ¼ or more of face smooth and shiny (Figure 36A, B)	31
–	Face completely sculptured, mostly rugoreticulate (Figure 36C, D)	33
31 (30)	Promesonotum in profile with relatively sharp transitions between anterior and dorsal faces, and between pronotum and mesonotum (Figure 37B); pilosity on gastral tergites forming a layer of stout suberect setae, and a sparse layer of decumbent setae (Figure 37C); face and pronotum almost completely smooth and shiny (Figures 37A, B) (southern Mexico)	*Stenamma andersoni* sp. n.
–	Promesonotum in profile more smoothly rounded; pilosity on gastral tergites variable, but without stout setae; face and pronotum sculpture variable	32
32 (31)	Postpetiole in profile bulging and distinctly larger than petiolar node (Figure 38A) (PPH 0.20–0.25, PPW 0.22–0.27, PPH/PH 0.96–1.14); eye larger (EL 0.11–0.16); face sculpture variable, but usually mostly smooth and shiny, with some carinulae around frontal lobes and anterolateral margins of head (Figure 36A); larger species (HL 0.67–0.86, HW 0.57–0.76, ML 0.84–1.09) (southern Mexico to Honduras)	*Stenamma maximon* sp. n. (part)
–	Postpetiole smaller, about same size as petiolar node (Figure 38B, C) (PPH 0.14–0.19, PPW 0.15–0.20, PPH/PH 0.79–0.97); eye smaller (EL 0.07–0.12); face sculpture usually more developed, with carinulae, punctae, and occasional rugoreticulae (Figure 36B); smaller species (HL 0.55–0.72, HW 0.46–0.64, ML 0.66–0.85) (southern U.S.A. to Mexico)	*Stenamma huachucanum* (part)
33 (30)	Pilosity on gastral tergites predominately suberect and relatively sparse (Figure 39A, B); decumbent setae if present very sparse	34
–	Pilosity on gastral tergites shorter, denser, and usually distinctly bilayered, with a layer of suberect setae and an equally dense layer of decumbent setae (Figure 39C–E)	35
34 (33)	Entire face and most of mesosoma densely punctate, or densely carinulate (longitudinal orientation on face), or intermediate, with carinulae emerging from borders of punctae (Figure 40A–C); eye larger (EL 0.15–0.18, REL 20–25), with 8–9 ommatidia at greatest diameter; propodeal spines absent to tuberculate, shorter (SSL 0.09–0.11, PSI 0.9–1.2) (Mexico to Honduras)	*Stenamma stictosomum* sp. n.
–	Face less densely sculptured, usually mostly rugoreticulate, but sometimes sculpture more polished, with reticulae indistinct (Figure 40D, E); eye smaller (EL 0.10–0.15, REL 18–22), with 5–8 ommatidia at greatest diameter; propodeal spines tuberculate to short, longer (SSL 0.08–0.16, PSI 1.1–1.8) (Mexico)	*Stenamma vexator* sp. n.
35 (33)	Propodeal declivity in profile forming a broadly sinuous connection between propodeal spine and propodeal lobe (Figure 41A) (southern Mexico [Oaxaca, Veracruz])	*Stenamma connectum* sp. n.
–	Propodeal declivity in profile straighter, leaving propodeal spine and lobe separated as distinct features (Figures 41B, 42A–F)	36
36 (35)	Petiole in profile appearing longer and more gracile, with node either dome-like and almost completely smooth and shiny (Figure 42A), or small and somewhat compressed dorsoventrally (Figure 42B); pronotum mostly sculptured, with punctae on side (Figure 42A, B), and rugae or rugoreticulae on dorsum; frontal lobes often (but not always) distinctly expanded, covering the torular lobes in full-face view (FLD 0.15–0.25, FLI 28–46); propodeal spines present, short to medium length (SSL 0.11–0.17, PSI 1.6–2.1)	37
–	Petiole in profile usually appearing shorter, more compact, and sometimes more robust, with node only shiny on anterior face and usually of moderate size (Figure 42C–F); side of pronotum variably sculptured, often mostly smooth, and usually not punctate (Figure 42C–F); frontal lobes almost always of average width, and never completely covering torular lobes in full-face view (FLD 0.11–0.20, FLI 23–31); propodeal spines variable, usually shorter (SSL 0.06–0.14, PSI 1.0–1.8)	38
37 (36)	Petiole relatively longer (PL/HW 0.60–0.68); petiolar and postpetiolar nodes mostly smooth and shiny (Figure 42A); postpetiole relatively smaller and appearing more shield-like (PPH/PH 0.75–0.84); scape and metafemur relatively shorter (SI 82–89, MFI 102–109); larger species (HL 0.65–0.76, HW 0.54–0.66, ML 0.81–0.99) (southern Mexico to Honduras)	*Stenamma hojarasca* sp. n.
–	Petiole relatively shorter (PL/HW 0.53–0.59); petiolar and postpetiolar nodes smooth only on anterior faces; postpetiole relatively larger (Figure 42B) (PPH/PH 0.85–0.91); scape and metafemur relatively longer (SI 92–104, MFI 84–95); smaller species (HL 0.61–0.68, HW 0.51–0.59, ML 0.75–0.82) (Honduras)	*Stenamma catracho* sp. n.
38 (36)	Postpetiole in profile bulging and distinctly larger than petiolar node (Figures 38A, 42C) (PPH 0.20–0.25, PPW 0.22–0.27, PPH/PH 0.96–1.14); eye larger (EL 0.11–0.16), with 6–8 ommatidia at greatest diameter (southern Mexico to Honduras)	*Stenamma maximon* sp. n. (part)
–	Postpetiole in profile smaller, about same size as petiolar node or smaller (Figure 42D–F) (PPH 0.12–0.20, PPW 0.13–0.22, PPH/PH 0.79–1.04); eye smaller (EL 0.07–0.12), with 4–8 ommatidia at greatest diameter (usually ≤ 6)	39
39 (38)	Head and mesosoma dark red-brown to orange-brown, with appendages a distinctly lighter orange- to yellow-brown (Figure 43A); basal margin of mandible with basal depression, shallow or deep (Figure 11F); eye often relatively smaller (REL 12–17); larger species (HL 0.63–0.83, HW 0.54–0.73, ML 0.76–1.09 PrW 0.37–0.50) (southern Mexico to Honduras)	*Stenamma ochrocnemis* sp. n.
–	Body and appendage color less contrasting, generally dark to light brown (Figure 43B); basal margin of mandible with basal depression shallow, never deep; eye often relatively larger (REL 14–21); smaller species (HL ≤ 0.72, HW ≤ 0.64, ML ≤ 0.85, PrW ≤ 0.41)	40
40 (39)	Pronotum usually longitudinally rugose on most of dorsum and upper half of side, with small patches of smooth cuticle on middle of dorsum and lower half of side, but sometimes dorsum completely rugose or mostly smooth; propodeal spines tuberculate to short, often relatively longer (PSI 1.2–1.8); petiole compact, with a relatively small node that points slightly posteriad (Figures 42E, 43B) (note geography is easiest way to separate species in this couplet) (southern Mexico [Chiapas] to Nicaragua)	*Stenamma crypticum* sp. n.
–	Pronotum sculpture variable, but usually not as above; propodeal spines absent or tuberculate, often relatively shorter (PSI 1.0–1.4); petiole variable, but often more elongate, or with the petiolar node distinctly enlarged and pointing vertically (Figure 42F) (southern U.S.A. to southern Mexico [Oaxaca])	*Stenamma huachucanum* (part)
41 (11)	Mesosoma in profile somewhat elongate, with metanotal groove wide, shallow, and indistinct, and propodeal dorsum markedly long and flat (Figure 44A); anterior clypeal margin in full-face view with a deep uneven median emargination (Figure 44B); basal tooth of mandible very robust and well-defined; eye relatively small (EL 0.09–0.11, REL 13–15), subcircular, with 5–6 ommatidia at greatest diameter (Honduras to Nicaragua)	*Stenamma monstrosum* sp. n.
–	Lacking one or more of the above characters states	42
42 (41)	Eye usually larger (EL 0.09–0.18, REL 18–29), with 6 or more ommatidia at greatest diameter (note other characterisctics of this species highly variable) (Nicaragua to Ecuador)	*Stenamma schmidti* (part)
–	Eye usually smaller (EL 0.05–0.14, REL 10–20), with 5 or fewer ommatidia at greatest diameter (southern Mexico to Nicaragua)	43
43 (42)	Anterior 4/5 of face sculptured with short evenly spaced carinulae or rugulae (longitudinal in orientation), remaining posterior surface smooth and shiny (Figure 45A); pilosity on gastral tergites forming a layer of longer suberect setae and a sparse layer of shorter decumbent setae, all setae of moderate thickness (Figure 46A); larger species (HL 0.62–0.73, HW 0.56–0.70, ML 0.77–0.89) (Nicaragua)	*Stenamma sandinista* sp. n.
–	Face completely sculptured, mostly rugoreticulate (Figure 45B, C); gastral pilosity variable, but not as above (Figure 46B, C); usually smaller species (HL ≤0.69, HW ≤0.59, ML ≤0.82) (southern Mexico to Honduras)	44
44 (43)	Pilosity on gastral tergites forming a short layer of dense decumbent to appressed setae and a sparse layer of short suberect setae (Figure 46B); propodeal spines tuberculate to short, longer (SSL 0.07–0.14, PSI 1.5–2.3); eye slightly smaller (EL 0.05–0.09, REL 10–16), subcircular, with 3–5 ommatidia at greatest diameter; scape and metafemur relatively shorter (SI 81–92, MFI 107–119); frontal lobes, not expanded outward (FLD 0.11–0.14, FLI 22–27) (southern Mexico to Honduras)	*Stenamma saenzae* sp. n.
–	Pilosity on gastral tergites forming a layer of stout suberect setae and a very sparse layer of appressed setae (Figure 46C); propodeal spines reduced to sharp angles or small tubercles, shorter (SSL 0.07–0.09, PSI 1.2–1.4); eye larger (EL 0.08–0.10, REL 15–18), more oval-shaped, with 4–5 ommatidia at greatest diameter; scape and metafemur longer (SI 90–99, MFI 97–104); frontal lobes slightly expanded outward (FLD 0.14–0.15, FLI 28–31) (Honduras)	*Stenamma nanozoi* sp. n.

**Figure 10.**
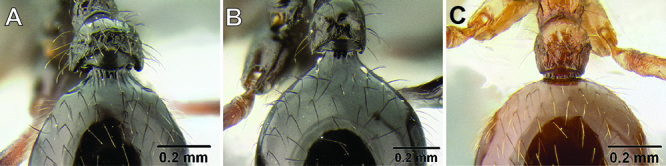
Anterior constriction of gaster in dorsal view **A** *Stenamma callipygium* (CASENT0606207) **B** *Stenamma atribellum* (CASENT0622351) **C** *Stenamma manni* (CASENT0605527).

**Figure 11.**
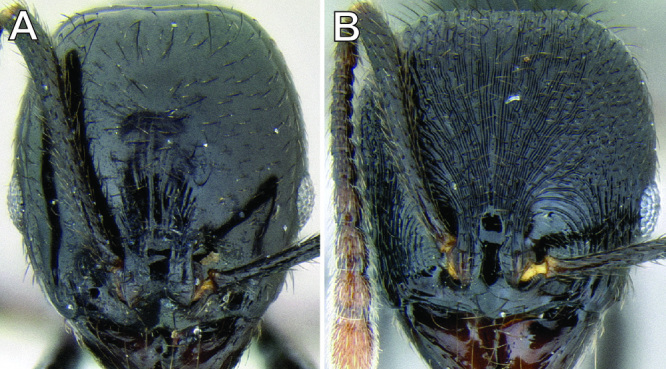
Face and anterior clypeal margin in full-face view **A** *Stenamma atribellum* (CASENT0622351) **B** *Stenamma callipygium* (CASENT0606207).

**Figure 12.**
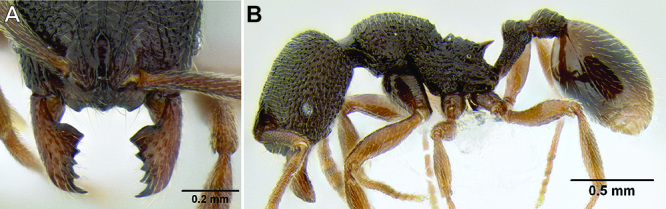
*Stenamma cusuco* (CASENT0622137) **A** Anterior clypeal margin in full-face view **B** Body in profile.

**Figure 13.**
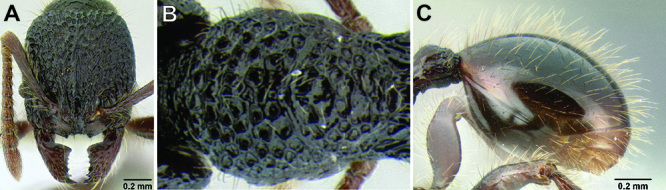
*Stenamma zelum* (CASENT0622535) **A** Face **B** Dorsum of promesonotum **C** Gastral pilosity.

**Figure 14.**
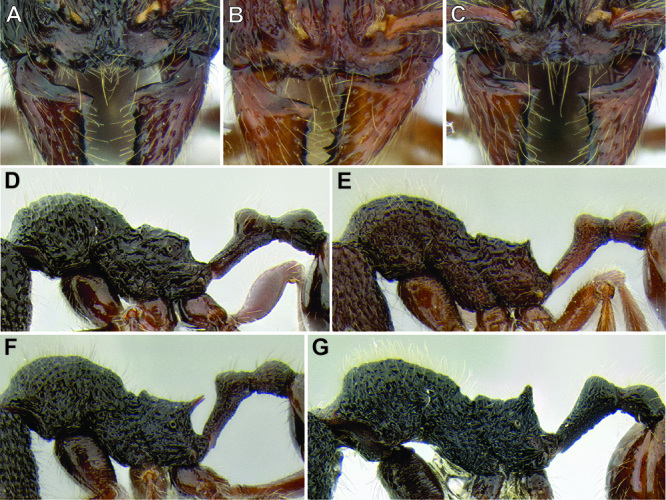
Comparison of clypeal structure (anterodorsal view) and mesosoma (profile) between **A, D*** Stenamma zelum* (CASENT0622535) **B** *Stenamma brujita* (CASENT0126254) **C** *Stenamma brujita* (CASENT0604945) **E** *Stenamma brujita* (CASENT0126254) **F** *Stenamma brujita* (CASENT0604945) **G** *Stenamma brujita* (CASENT0604607).

**Figure 15.**
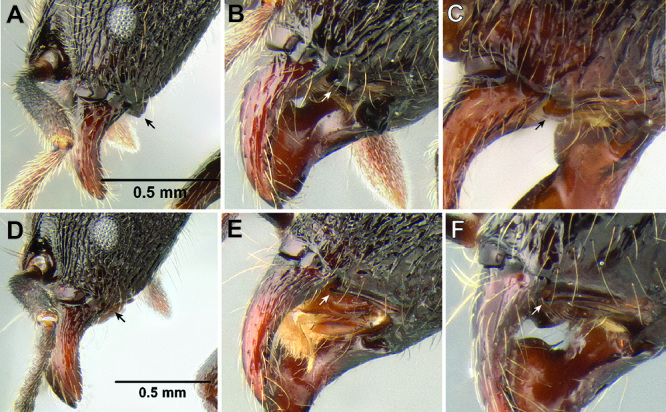
Lateral apex of hypostomal bridge **A** *Stenamma megamanni* (CASENT0622853) head in profile view **B** *Stenamma megamanni* (CASENT0622853) head in lateroventral view **C** *Stenamma manni* (CASENT0604893) in lateroventral view **D** *Stenamma felixi* (CASENT0620969) head in profile view **E** *Stenamma felixi* (CASENT0620969) head in lateroventral view **F** *Stenamma maximon* (CASENT0604675) head in lateroventral view.

**Figure 16.**
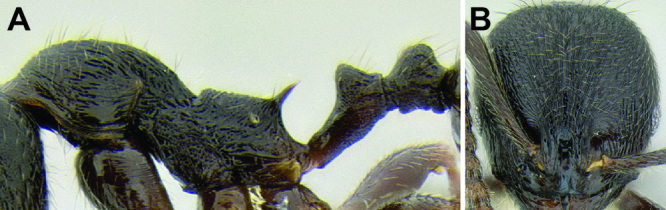
*Stenamma leptospinum* (CASENT0605530) **A** Mesosoma and waist in profile **B** Face.

**Figure 17.**
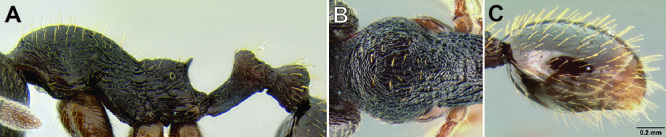
*Stenamma longinoi* (JTLC000007475) **A** Mesosoma in profile **B** Dorsum of promesonotum **C** Gastral pilosity.

**Figure 18.**
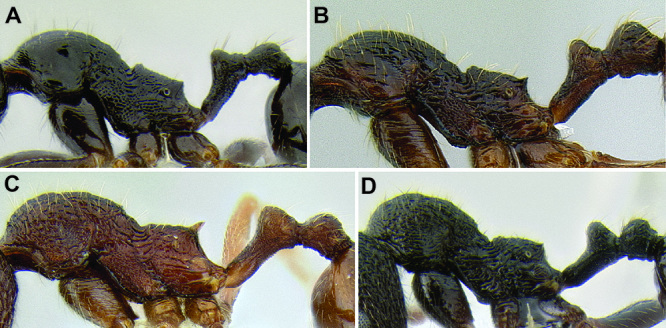
Mesosoma and waist in profile. **A** *Stenamma muralla* (CASENT0621311) **B** *Stenamma manni* (CASENT0621574) **C** *Stenamma manni* (CASENT0605592), **D** *Stenamma megamanni* (CASENT0604730).

**Figure 19.**
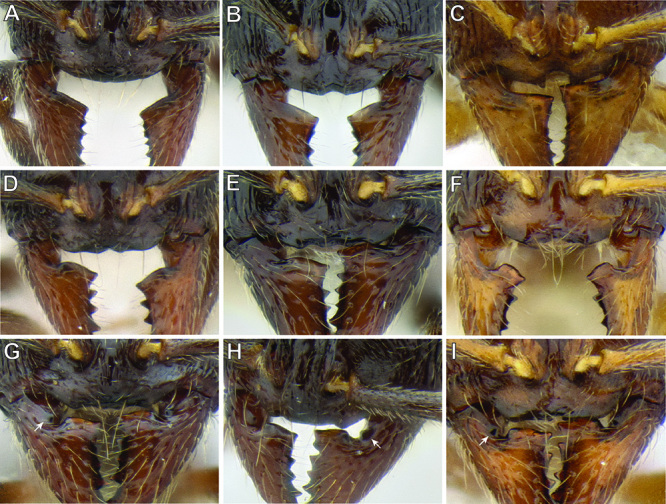
Variation in the structure of the anterior clypeal margin and the basal margin of the mandible **A** *Stenamma nonotch* (CASENT0604711) **B** *Stenamma muralla* (CASENT0621311) **C** *Stenamma excisum* (CASENT0605563) **D** *Stenamma crypticum* (CASENT0603821) **E** *Stenamma maximon* (CASENT0604875) **F** *Stenamma ochrocnemis* (CASENT0621468) **G, H** *Stenamma schmidti* (INB0003665417) **I** *Stenamma saenzae* (CASENT0604912).

**Figure 20.**
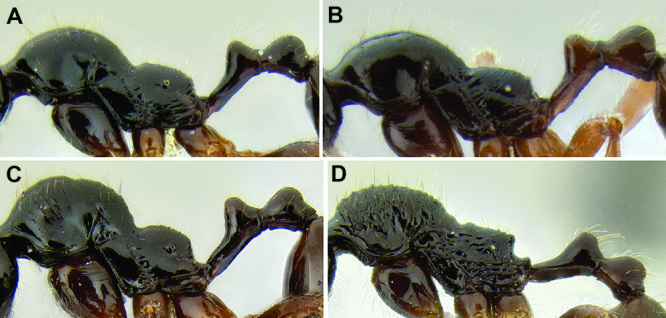
Mesosoma and waist in profile **A** *Stenamma expolitum* (CASENT0600043) **B** *Stenamma alas* (JTLC000005880) **C** *Stenamma alas* (CASENT0606832) **D** *Stenamma expolitico* (INBIO282473).

**Figure 21.**
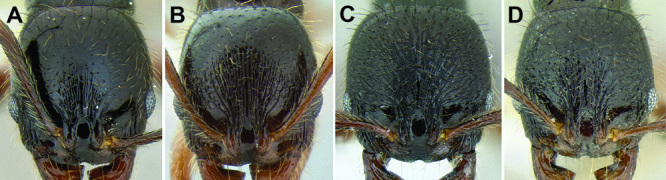
Face sculpture **A** *Stenamma expolitum* (CASENT0600043) **B** *Stenamma alas* (JTLC000005880) **C ***Stenamma alas* (CASENT0606832) **D** *Stenamma expolitico* (INBIO282473).

**Figure 22.**
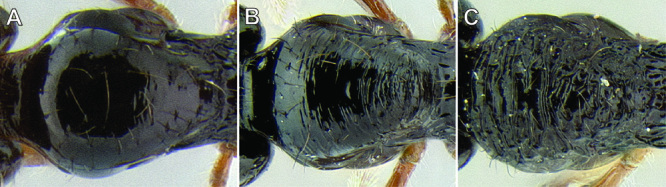
Dorsum of promesonotum **A** *Stenamma alas* (JTLC000005880) **B** *Stenamma alas* (CASENT0606832) **C** *Stenamma expolitico* (INBIO282473).

**Figure 23.**
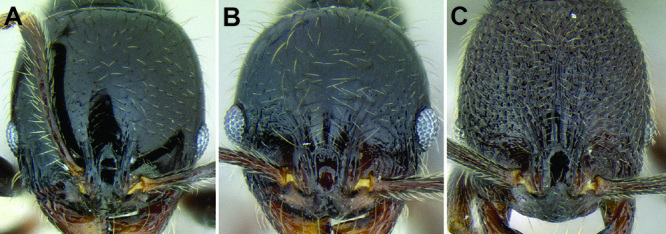
Face sculpture **A** *Stenamma lobinodus* (CASENT0622422) **B** *Stenamma tico* (CASENT0622416) **C ***Stenamma nonotch* (CASENT0604711).

**Figure 24.**
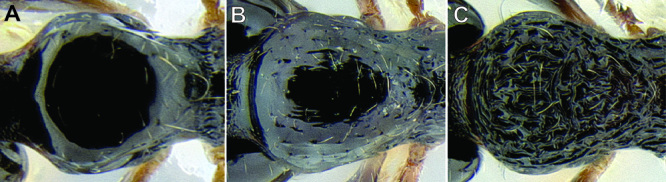
Sculpture on dorsum of promesonotum **A** *Stenamma pelophilum* (CASENT0605613) **B** *Stenamma llama* (CASENT0604952) **C** *Stenamma lobinodus* (CASENT0605658).

**Figure 25.**
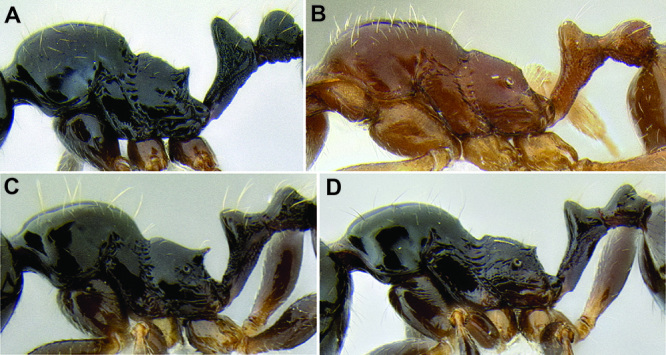
Mesosoma and waist in profile view **A** *Stenamma llama* (CASENT0605236) **B** *Stenamma tiburon* (CASENT0620965) **C** *Stenamma pelophilum* (CASENT0605613) **D** *Stenamma pelophilum* (CASENT0605428).

**Figure 26.**
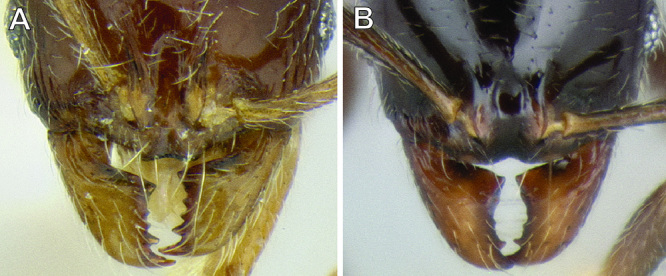
Clypeus in full-face view **A** *Stenamma tiburon* (CASENT0620965) **B** *Stenamma pelophilum* (CASENT0606223).

**Figure 27.**
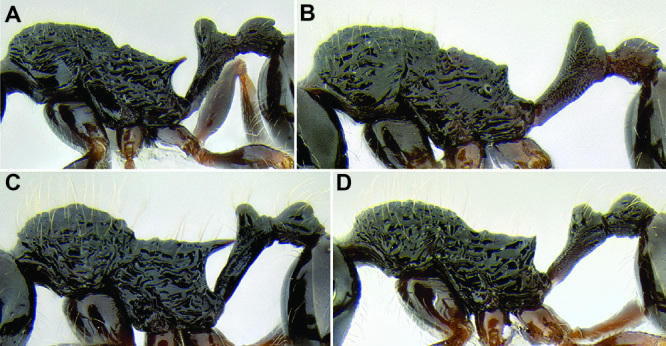
Mesosoma and waist in profile **A** *Stenamma lobinodus* (CASENT0605658) **B** *Stenamma lobinodus* (CASENT0605814) **C** *Stenamma diversu* m (CASENT0606723*) D S. tico (CASENT0622571)*.

**Figure 28.**
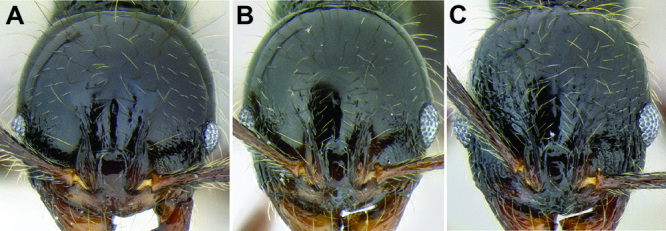
Face **A** *Stenamma diversum* (CASENT0606723) **B** *Stenamma tico* (CASENT0622416) **C** *Stenamma tico* (CASENT0600104).

**Figure 29.**
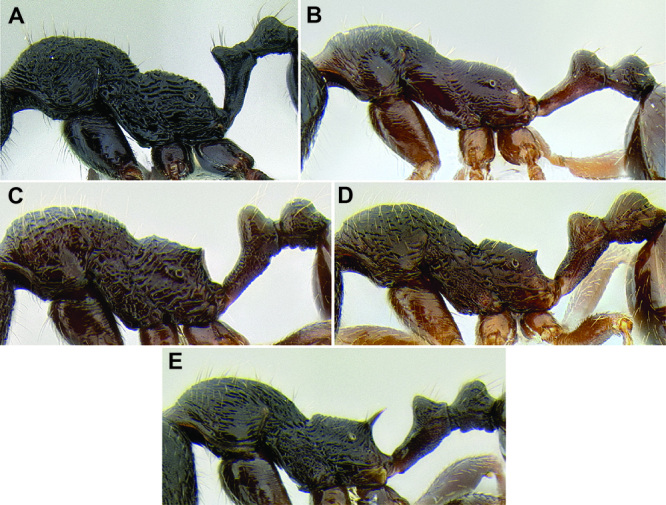
Mesosoma in profile **A** *Stenamma felixi* (CASENT0620969) **B** *Stenamma schmidti* (INB0003210597) **C** *Stenamma nonotch* (CASENT0605789)**D** *Stenamma manni* (CASENT0604893) **E** *Stenamma leptospinum* (CASENT0605530).

**Figure 30.**
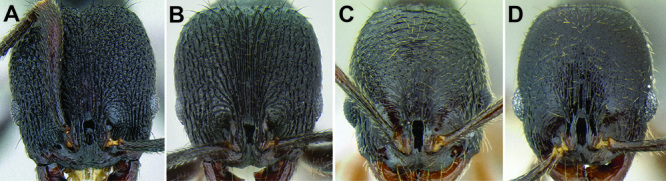
Face sculpture **A** *Stenamma felixi* (CASENT0620969) **B** *Stenamma felixi* (CASENT0622555) **C** *Stenamma schmidti* (INB0003210597) **D** *Stenamma schmidti* (INB0002659320).

**Figure 31.**
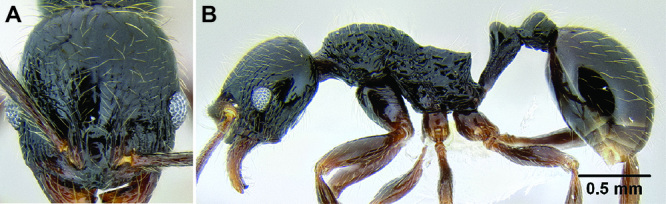
*Stenamma tico* (CASENT0600104) **A** Face **B** Body in profile.

**Figure 32.**
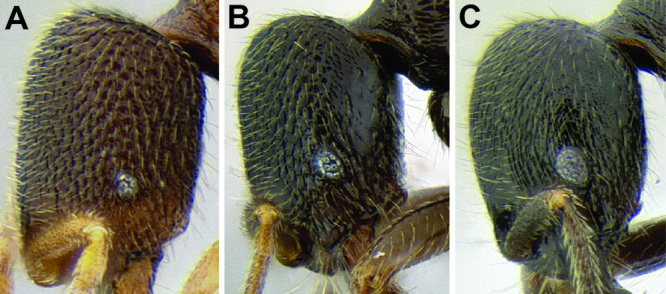
Head and eye in profile view **A** *Stenamma excisum* (CASENT0605563) **B** *Stenamma ignotum* (CASENT0603762) **C** *Stenamma leptospinum* (CASENT0605530).

**Figure 33.**
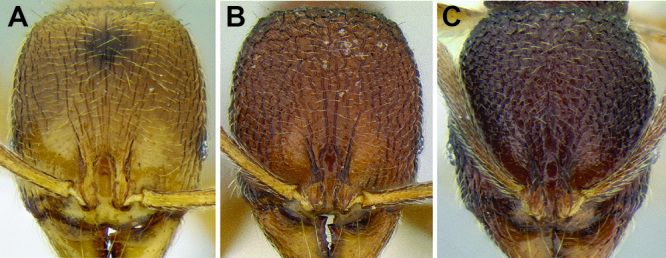
Faceand clypeus in full-face view **A** *Stenamma lagunum* (CASENT0622371) **B** *Stenamma excisum* (CASENT0621834) **C** *Stenamma excisum* (CASENT0605441).

**Figure 34.**
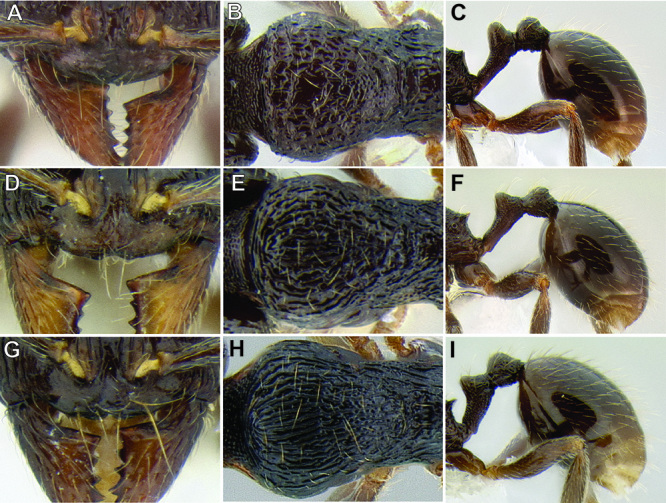
Comparison of the anterior clypeal margin, pronotal dorsum, waist, and gaster **A–C ***Stenamma nonotch* (CASENT0605789) **D–F** *Stenamma ignotum* (CASENT0603762) **G–I** *Stenamma picopicucha* (CASENT0606709).

**Figure 35.**
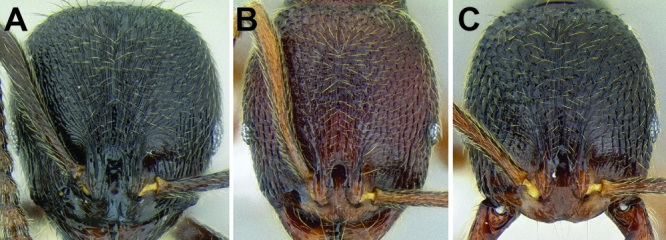
Face sculpture **A** *Stenamma leptospinum* (CASENT0605530) **B** *Stenamma manni* (CASENT0605592) **C** *Stenamma manni* (CASENT0604893).

**Figure 36.**
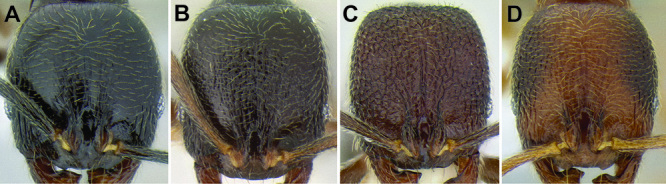
Face sculpture **A** *Stenamma maximon* (CASENT0603886) **B** *Stenamma huachucanum* (CASENT0605616) **C** *Stenamma catracho* (CASENT0621306) **D** *Stenamma ochrocnemis* (CASENT0605129).

**Figure 37.**
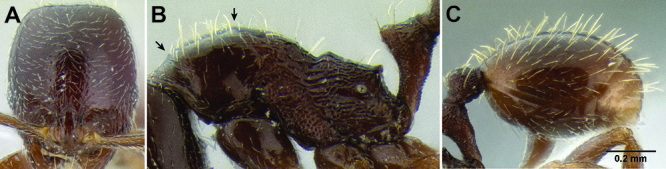
*Stenamma andersoni* (CASENT0604603) **A** Head **B** Mesosoma in profile **C** Gastral pilosity.

**Figure 38.**
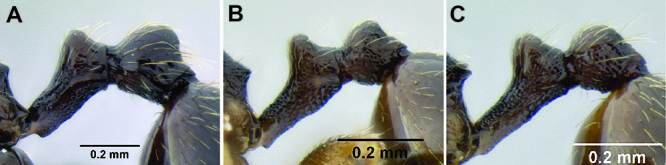
Petiole and postpetiole in profile view **A** *Stenamma maximon* (CASENT0603886) **B** *Stenamma huachucanum* (CASENT0605616) **C** *Stenamma huachucanum* (CASENT0605647).

**Figure 39.**
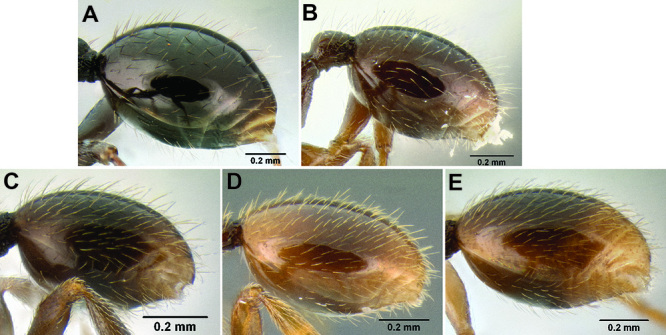
Gastral pilosity **A** *Stenamma stictosomum* (CASENT0605499) **B** *Stenamma vexator* (CASENT0126485) **C** *Stenamma crypticum* (CASENT0605185) **D** *Stenamma hojarasca* (CASENT0622132) **E** *Stenamma ochrocnemis* (CASENT0621468).

**Figure 40.**
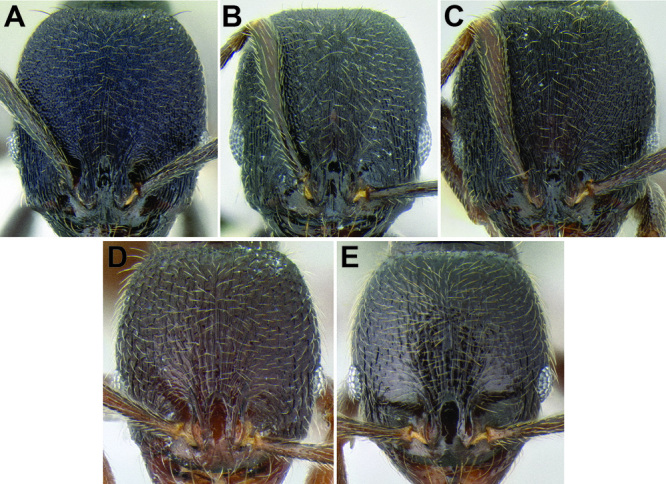
Face sculpture **A** *Stenamma stictosomum* (CASENT0605499) **B** *Stenamma stictosomum* (CASENT0606221) **C** *Stenamma stictosomum* (CASENT012624) **D** *Stenamma vexator* (CASENT0604641) **E** *Stenamma vexator* (CASENT0605506).

**Figure 41.**
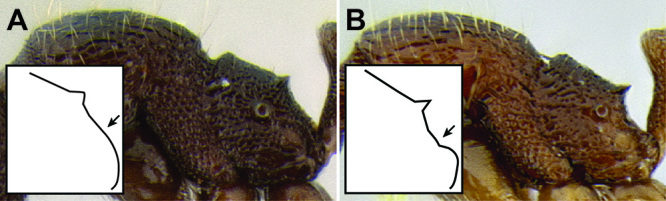
Mesosoma in profile **A** *Stenamma huachucanum* (CASENT0605586) **B** *Stenamma crypticum* (CASENT0603755).

**Figure 42.**
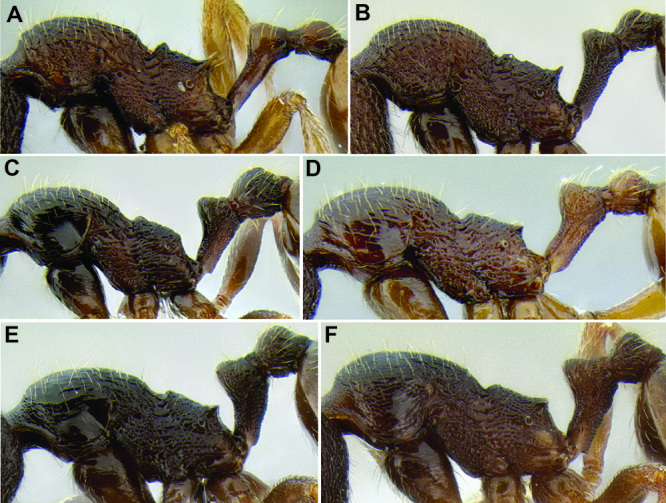
Mesosoma and waist in profile **A** *Stenamma hojarasca* (CASENT0622132) **B** *Stenamma catracho* (CASENT0621306)**C** *Stenamma maximon* (CASENT0605063)**D** *Stenamma ochrocnemis* (CASENT0603793) **E** *Stenamma crypticum* (CASENT0603821) **F** *Stenamma huachucanum* (CASENT0126556).

**Figure 43.**
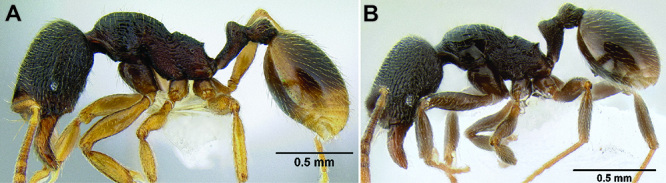
Body and appendages in profile view **A** *Stenamma ochrocnemis* (CASENT0621468) **B** *Stenamma crypticum* (CASENT0603821).

**Figure 44.**
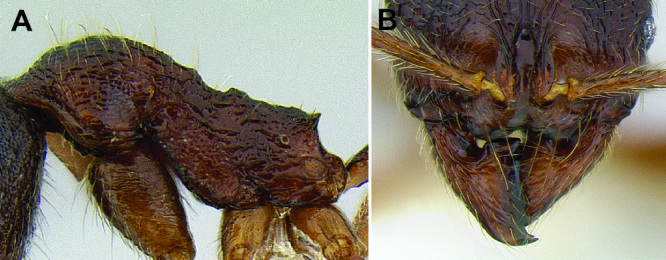
*Stenamma monstrosum* (CASENT0621327) **A** Mesosoma in profile **B** Clypeus and mandibles in full-face view.

**Figure 45.**
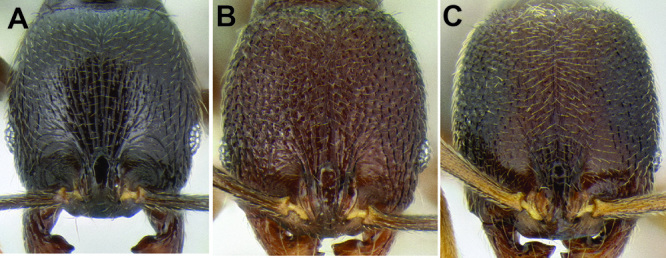
Face sculpture **A** *Stenamma sandinista* (CASENT0622578)**B** *Stenamma nanozoi* (CASENT0621828) **C** *Stenamma saenzae* (CASENT0604912).

**Figure 46.**
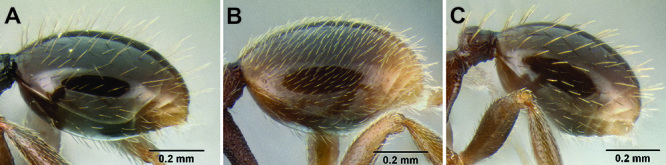
Gastral pilosity **A** *Stenamma sandinista* (CASENT0622578) **B** *Stenamma saenzae* (CASENT0603860) **C** *Stenamma nanozoi* (CASENT0621828).

## Species accounts

### Stenamma alas

Longino

http://species-id.net/wiki/Stenamma_alas

**Figure 47.**
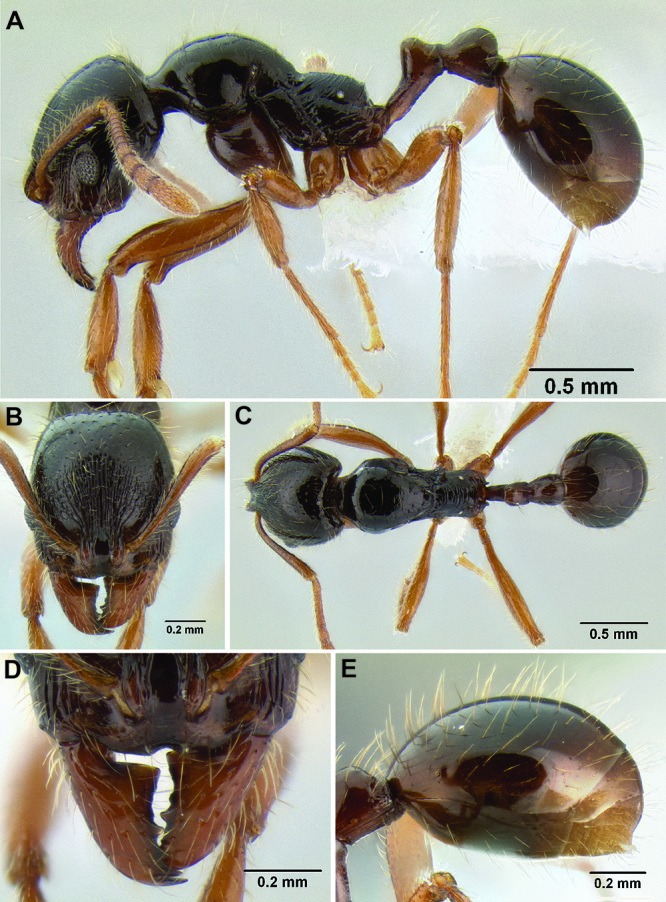
*Stenamma alas* paratype worker (JTLC000005880) **A** Profile **B** Face **C** Dorsum **D** Anterior clypeal margin in anterodorsal view **E** Gaster.

**Figure 48.**
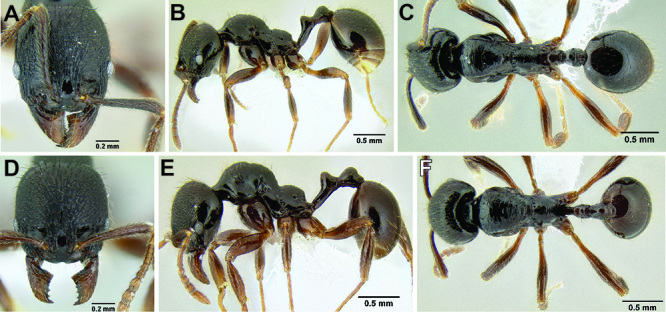
*Stenamma alas* worker variants. Head, profile, and dorsal views **A–C** Variant 1 (CASENT0600114) **D–F** Variant 2 (CASENT0606832).

**Figure 49.**
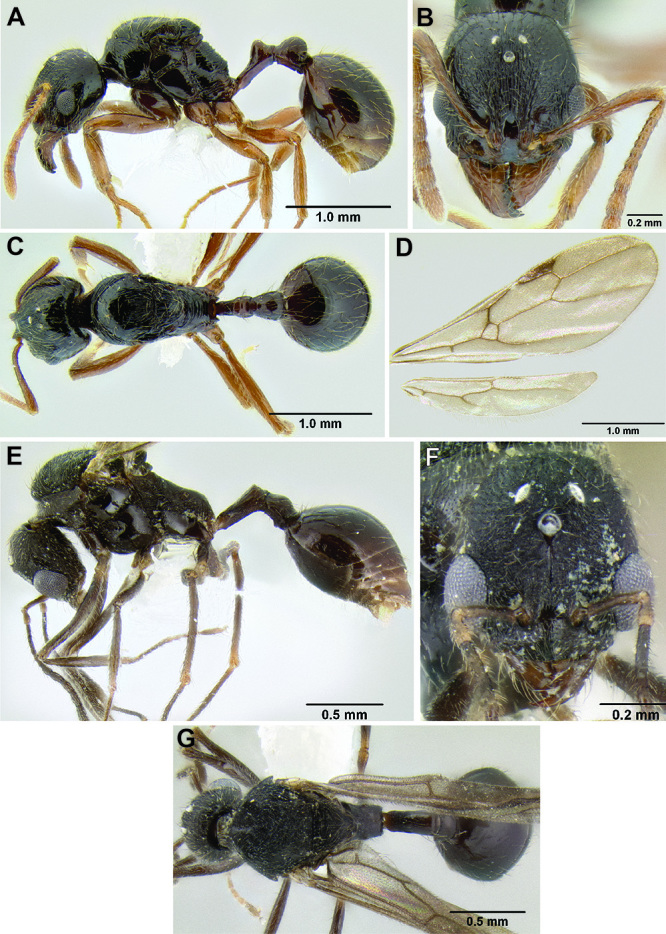
*Stenamma alas* **A** Queen (CASENT000005886), profile **B** Same, face **C** Same, dorsum **D **Queen (CASENT0623104), wings **E** Male (CASENT0623103), profile **F** Same, face **G **Same, dorsum.

**Figure 50.**
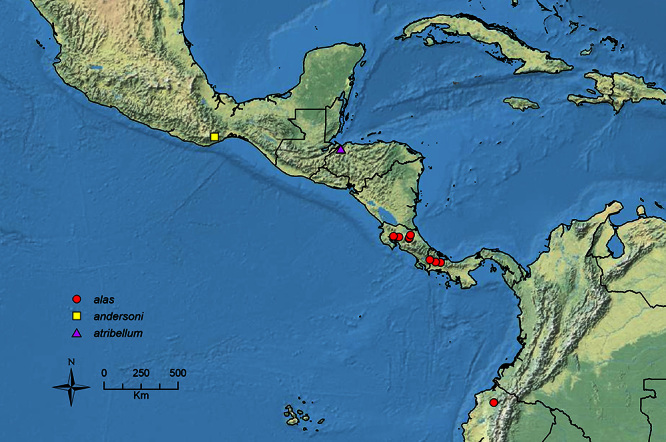
Distribution map of *Stenamma alas* (circles), *Stenamma andersoni* (squares), and *Stenamma atribellum* (triangles).

Worker: Figures 47 , 48 ; Queen: Figure 49A–D ; Male: Figure 49E–G ; Map: Figure 50

Stenamma alas Longino, 2005: 672, figs 1, 2. Holotype worker: COSTA RICA, Prov. Heredia: 11km ESE La Virgen, 10°21'N, 84°03'W [10.350°N, 84.050°W], 300m, 15 April 2004, (J. Longino, collection JTL5338) (INBio, specimen JTLC000005588) [examined]. Branstetter, 2012: phylogeny.

#### Worker diagnosis.

Integument mostly black to dark red-brown, with appendages uniformly orange-brown, or mostly dark brown changing to orange-brown at extremities; medium- to large-sized species (see HL, ML, PrW below); anterior clypeal margin with a median emargination; basal margin of mandible straight; propodeal spines absent (PSL 0.10–0.15, PSI 0.7–1.0); petiole and postpetiole almost completely smooth and shiny, with only faint vestigial punctae sometimes present; postpetiole in profile bulging, globular, appearing more voluminous than petiolar node; face with a fan of carinulae extending from frontal lobes to just past midpoint of head or further, sometimes reaching posterior and lateral margins, carinulae when completely covering face, often very dense (almost striate); promesonotum completely smooth and shiny, or with a variable number of transverse striae on dorsal surface; promesonotum in profile domed, symmetrical, and moderately to strongly bulging; eye relatively large (EL 0.14–0.18, REL 18–23), oval-shaped, with 8–10 ommatidia at greatest diameter; setae on gastral dorsum sparse, long, and mostly suberect; frontal lobes of moderate width (FLD 0.20–0.29, FLI 29–32), not obscuring torular lobes in full-face view. *Similar species*: *Stenamma expolitico*, *Stenamma expolitum*.

#### Geographic range.

Costa Rica to Ecuador.

#### Worker description.

(17 measured, paratype JTLC000005880 in parentheses) HL 0.77–0.98 (0.85), HW 0.66–0.88 (0.76), FLD 0.20–0.29 (0.23), PCW 0.05–0.09 (0.07), SL 0.65–0.77 (0.72), EL 0.14–0.18 (0.17), ACL 0.64–0.75 (0.70), ML 1.02–1.30 (1.16), PrW 0.51–0.66 (0.57), PSL 0.10–0.15 (0.13), SDL 0.11–0.15 (0.13), PL 0.37–0.51 (0.41), PH 0.22–0.28 (0.25), PW 0.16–0.22 (0.18), PPL 0.24–0.32 (0.29), PPH 0.23–0.28 (0.25), PPW 0.20–0.28 (0.24), MFL 0.78–1.00 (0.88), MTL 0.64–0.78 (0.70), CI 86–93 (90), SI 83–101 (95), REL 18–23 (22), FLI 29–32 (30), PSI 0.7–1.0 (1.0), MFI 84–94 (87), ACI1 61–65 (62), ACI2 94–101 (98).

Medium- to large-sized species; general body color black to dark red-brown, with appendages uniformly orange-brown (type population), or mostly dark brown changing to orange-brown at joints and extremities; setae golden brown; mandible with 5–7 teeth, consisting of 4 distinct apical teeth, a basal tooth, and 1–2 worn teeth/denticles in between; basal margin of mandible straight, without a basal notch or depression; mandible surface mostly smooth, with scattered piligerous punctae and a few striations on base and lateral surface; anterior clypeal margin with a median emargination; median lobe of clypeus obliquely flattened, mostly smooth and shiny, with a short transverse carinula near anterior margin, remainder of clyepeus mostly smooth and shiny; posterior extension of clypeus between antennal insertions somewhat wide (PCW 0.05–0.09), with sides subparallel to slightly diverging anteriorly; frontal lobes of moderate width (FLD 0.20–0.29, FLI 29–32), not greatly obscuring torular lobes in full-face view; head roughly oval-shaped (CI 86–93), with posterior margin flat, to genetly convex, not depressed medially; eye relatively large (EL 0.14–0.18, REL 18–23), oval-shaped, with 8–10 ommatidia at greatest diameter; face with a fan of carinulae extending from frontal lobes to just past midpoint of head (type population) or further, sometimes reaching posterior and lateral margins, carinulae when completely covering face, often very dense (almost striate); gena with some carinulae; posterolateral and ventral surfaces of head smooth and shiny; scape of moderate length (SI 83–101), reaching, but not distinctly surpassing posterior margin of head in full-face view; scape surface mostly smooth, with scattered piligerous punctae; flagellum with distinct 4-segmented antennal club; promesonotum completely smooth and shiny (type population), or with a variable number of transverse striae on dorsal surface, remainder of mesosoma mostly smooth, except for transverse carinulae on propodeal dorsum, and a few rugulae on side of propodeum and mesopleuron; promesonotum in profile domed, symmetrical, and moderately to strongly bulging; metanotal groove distinct, but often shallow; propodeal spines absent (PSL 0.10–0.15, PSI 0.7–1.0); dorsum of propodeum in profile slightly to strongly convex, usually not flat; petiole and postpetiole almost completely smooth and shiny, with only faint vestigial punctae sometimes present, mostly on venters; postpetiole in profile bulging, globular, appearing more voluminous than petiolar node (PPH/PH 0.89–1.06, PW/PPW 0.73–0.84); petiole in profile appearing of moderate length (PL/HW 0.50–0.58); petiolar node in profile nearly symmetrical, dorsum of node broadly rounded, and pointed vertically to slightly posteriad; gaster smooth and shiny, with scattered piligerous punctae; face with short suberect to decumbent pilosity; setae on remainder of body dorsum sparse, long, and mostly suberect; setae on scapes subdecumbent; setae on legs mostly subdecumbent, with longer suberect setae on femoral venters and coxae.

#### Queen description.

(5 measured) HL 0.82–0.94 (0.82), HW 0.73–0.87 (0.73), FLD 0.23–0.27 (0.23), PCW 0.07–0.09 (0.07), SL 0.70–0.92 (0.70), EL 0.22–0.23 (0.22), ACL 0.71–0.78 (0.72), ML 1.18–1.42 (1.18), PrW 0.66–0.79 (0.66), PSL 0.13–0.15 (0.14), SDL 0.12–0.15 (0.13), PL 0.44–0.52 (0.44), PH 0.26–0.30, PW 0.19–0.23 (0.19), PPL 0.29–0.35 (0.29), PPH 0.27–0.31 (0.27), PPW 0.25–0.30 (0.25), MFL 0.86–0.99 (0.86), MTL 0.68–0.79 (0.68), CI 89–93 (89), SI 88–108 (96), REL 25–30 (30), FLI 30–32 (31), PSI 0.9–1.1 (1.0), MFI 85–91 (85), ACI1 62–63 (63), ACI2 79–103 (103).

Same as worker except for standard queen modifications and as follows: face sculpture usually slightly longer, and denser; pronotum with faint transverse striations; posterior half of mesoscutum with median patch of longitudinal carinulae; scutellum longitudinally carinulate; wing venation as in Figure 49D.

#### Male.

See Figure 49E–G.

#### Biology.

The nesting biology of *Stenamma alas* is described in detail in Longino (2005) and was reviewed in the overview of natural history section above, but is summarized again here. *Stenamma alas* is a specialized inhabitant of clay bank environments. Nests are found in nearly vertical clay banks along streams or vertical cuts along trails. *Stenamma alas* occurs in relatively pristine wet forest habitats from 50 to approximately 1800 m (note the holotype form of *Stenamma alas* only reaches about 1600 m). I have noticed that *Stenamma alas* is most abundant at mid-elevations between 300–800 m elevation. At lower elevations, *Stenamma expolitum*, which is a closely related and often sympatric species, seems to be more dominant.

Colonies include one to five closely spaced nests, but the queen, brood, and most of the workers only occupy one of them. Colonies seem to be continually building new nests and occasionally migrating from one to another. Each nest consists of a horizontally oriented ear-like turret that is sunk into a small alcove. The nest entrance is in the middle of the turret. Next to the entrance the workers always maintain a small clay “door pebble” and when the proper stimulus is applied to the nest entrance, such as an army ant or other predaceous ant, an *Stenamma alas* worker quickly emerges from the nest and closes the entrance with the pebble. It is hypothesized that all of these complex nesting behaviors evolved to avoid predation by army ants.

Each *Stenamma alas* nest contains a single small chamber. Colonies are fairly large for *Stenamma*, with up to 250 individuals. All excavated colonies have contained only one egg-laying queen. Foragers are solitary, slow moving and freeze when disturbed. It is unknown what *Stenamma alas* forages on primarily, but I have observed workers returning to the nest with cookie baits and small pieces of unidentified organic matter, suggesting that the species might be a generalist scavenger.

#### Comments.

*Stenamma alas*, along with *Stenamma expolitico* and *Stenamma expolitum*, belongs to the *expolitum* species group (a diagnosis of this group is given under *Stenamma expolitum* below). *Stenamma alas* is easily separated from *Stenamma expolitico* and *Stenamma expolitum* by comparing the sculpturing on the face and promesonotal dorsum. In the field, the holotype form of *Stenamma alas* (discussed below) can be separated from *Stenamma expolitum* by the structure of the nest entrance. *Stenamma alas* nests always have a horizontal turret, whereas *Stenamma expolitum* nests have vertical turrets.

As I have circumscribed it here, *Stenamma alas* represents a complex of species, whose boundaries are not clear. The type form of *Stenamma alas* (Figure 47) occurs only in Costa Rica. It is characterized by the following: facial carinulae extending to about midpoint of head, but not further; pronotum completely smooth and shiny; legs uniformly orange-brown. Variant 1 (Figure 48A–C) differs from the holotype form as follows: facial carinulae more extensive, sometimes very dense and extending all the way to the posterior margin of the head; legs dark brown to brown; dorsum of promesonotum with variably developed transverse striations. Variant 2 (Figure 48D–F) is the same as variant 1 except that the promesonotum in profile is strongly bulging upward, appearing high-domed. Variant 2 is known only from a few localities in the Bocas del Toro and Chiriquí provinces of Panama. It does not occur in sympatry with the other forms and some specimens appear intermediate, with the promesonotum less bulging. There is also variation in how dense and long the facial carinulae appear.

Variant 1 includes specimens from Costa Rica to Ecuador, but it does not appear to be a monophyletic entity. The specimens in Costa Rica occur at high elevation, above 1500 m and Longino (pers. comm.) reported finding nests in the ground, rather than in clay banks. One nest was found in a small clay hummock in the middle of a trail in forest. The other was in the ground under leaf litter in forest. There is some variation in how dense the facial carinulae are among sites, with a specimen from Las Alturas having very dense carinulae, similar to variant 2. The variant 1 specimens in Ecuador look nearly identical to those in Costa Rica, with some variation in facial sculpture. They are from lower elevation (800–900 m) and have nests in clay banks like the type form of *Stenamma alas* (Donoso, pers. comm.).

Molecular phylogenetic data show that variant 1 specimens from Costa Rica form a clade sister to *Stenamma expolitum* and *Stenamma expolitico* (Branstetter unpublished data). Specimens from Ecuador form a clade sister to *Stenamma alas*. No specimens from Panama have been sampled yet. This result suggests that the variant 1 specimens in Costa Rica are distinct from the type form of *Stenamma alas* and the specimens in Ecuador, but I cannot tell them apart based on worker or queen morphology. Thus, I treat *Stenamma alas* as a paraphyletic species, but acknowledge that it could include multiple cryptic taxa. More morphological and molecular data will be needed to resolve this problematic species.

#### Material examined:

**COSTA RICA: *Alajuela***: 3km E Monteverde, 10.300°N, 84.7833°W, 1400m, 26 Apr 1990 (J. Longino); Río Peñas Blancas, 10.3167°N, 84.7167°W, 800m, 4 Mar 2004 (J. Longino); ***Heredia***: La Selva Biological Station, 10.43047°N, 84.00675°W, 100m, 5 Jun 2007 (M. G. Branstetter); 8km ENE Vara Blanca, 10.20°N, 84.10°W, 1800m, 16 Apr 2002 (ALAS); 9km NE Vara Blanca, 10.233°N, 84.083°W, 1500m, 8 Mar 2005 (J. Longino); 10km NE Vara Blanca, 10.233°N, 84.083°W, 1500m, 12 Feb 2005 (ALAS); 13km NE Vara Blanca, 10.2667°N, 84.0833°W, 1100m, 16 Apr 2001 (ALAS); 11km ESE La Virgen, 10.35°N, 85.05°W, 300m, 15 Apr 2004 (J. Longino); 12km N Vol. Barba, 10.250°N, 84.083°W, 1420m, 10 Jul 1986 (J. Longino); 13km N Vol. Barba, 10.250°N, 84.083°W, 1320m, 10 Jul 1986 (J. Longino); ***Puntarenas***: Las Alturas Biological Station, 8.94997°N, 82.83375°W, 1800m, 26 May 2007 (M. G. Branstetter); Monteverde, 10.30°N, 84.80°W, 1400m, Apr–May 1987 (S. Little); **ECUADOR:** ***Pichincha***: Otongachi, 0.313°N, 78.950°W, 850m, 6 Aug 2009 (G. Ramón); Río Toachi, 4km W La Palma, 0.3183°N, 78.9533°W, 870m, 25 Jan 2006 (D. A. Donoso); **PANAMA: *Bocas del Toro***: Fortuna-Chiriquí Grande Rd., 8.78333°N, 82.1833°W, 800m, 16 Jul 1987 (D. M. Olson); Sendero Divisa, 15km SSW Chiriquí Grande, 8.783°N, 82.200°W, 1250m, 9 Jul 1987 (D. M. Olson); ***Chiriquí***: El Mirador, Finca Collins, nr Boquete, [ca. 8.813°N, 82.484°W], 1830m, 26 Jun 1976 (A. F. Newton).

### Stenamma andersoni sp. n.

urn:lsid:zoobank.org:act:6EC8C7EC-60F7-47A9-8099-89D349DC73C1

http://species-id.net/wiki/Stenamma_andersoni

**Figure 51.**
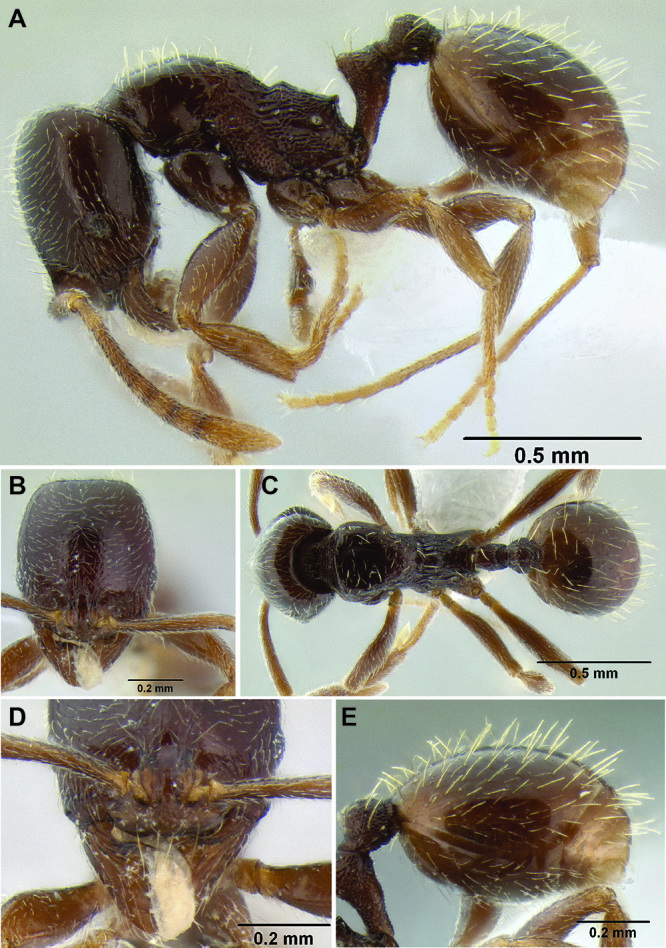
*Stenamma andersoni* holotype worker (CASENT060460) **A** Profile **B** Face **C** Dorsum **D **Anterior clypeal margin in anterodorsal view **E** Gaster.

Worker: Figure 51 ; Map: Figure 50

#### Type material.

*Holotype worker*. MÉXICO, Oaxaca: 10.9km N. Candelaria, [ca. 16.220°N, 95.891°W], 990m, 12 Jul 1987, cloud forest, ex sifted leaf litter (R. S. Anderson, collection RSA87-15) [USNM, specimen CASENT0604603]. *Paratype*: same data as holotype [1w, UNAM, CASENT0604602].

**Worker diagnosis.** Integument brown to red-brown (note that observed specimens are old, fresh specimens probably darker); small-sized species (see HL, ML, PrW below); anterior clypeal margin undulating, forming 2–4 small blunt teeth; basal margin of mandible sinuous, with a shallow basal depression, but without a basal tooth; head and pronotum mostly smooth and shiny; promesonotum in profile with relatively sharp transitions between anterior and dorsal faces, and between pronotum and mesonotum; pilosity on gastral tergites forming a layer of stout suberect setae, and a sparse layer of decumbent setae; eye of moderate size (EL 0.08–0.09, PSL 0.08–0.10), oval-shaped, with 5 ommatidia at greatest diameter; propodeal spines tuberculate (PSL 0.08–0.10, PSI 1.2–1.3); frontal lobes of moderate width (FLD 0.12–0.13, FLI 23–24), not obscuring torular lobes in full-face view. *Similar species*: *Stenamma connectum*, *Stenamma crypticum*, *Stenamma huachucanum*, *Stenamma maximon*.

#### Geographic range.

Southern Mexico.

#### Worker description.

(2 measured) HL 0.60–0.63 (0.63), HW 0.50–0.55 (0.55), FLD 0.12–0.13 (0.13), PCW 0.03 (0.03), SL 0.49–0.52 (0.52), EL 0.08–0.09 (0.09), ACL 0.47–0.49 (0.49), ML 0.76–0.81 (0.81), PrW 0.36–0.39 (0.39), PSL 0.08–0.10 (0.10), SDL 0.06–0.08 (0.08), PL 0.27–0.29 (0.29), PH 0.16–0.19 (0.19), PW 0.12–0.13 (0.13), PPL 0.15–0.16 (0.16), PPH 0.14–0.16 (0.16), PPW 0.16–0.17 (0.17), MFL 0.54–0.57 (0.57), MTL 0.42–0.45 (0.45), CI 85–88 (85), SI 95–98 (95), REL 16–17 (17), FLI 23–24 (23), PSI 1.2–1.3 (1.2), PI 53–54 (53), MFI 93–97 (97), ACI1 68 (68), ACI2 94–96 (94).

Small-sized species; general body color brown to red-brown (note observed specimens are older, fresh material almost certainly darker), with appendages lighter, brown to yellow-brown at extremities; setae golden; mandible with 6 teeth; basal margin of mandible sinuous, with a shallow basal depression, but no basal tooth; mandible mostly smooth, except for some conspicuous basal striae, and scattered piligerous punctae; anterior clypeal margin undulating, forming 2–4 small blunt teeth; median lobe of clypeus smooth, without noticeable carinulae, apex of lobe with a short transverse carinulae, remainder of clypeus smooth and shiny; posterior extension of clypeus between antennal insertions somewhat narrow (PCW 0.03), with sides subparallel; frontal lobes of moderate width (FLD 0.12–0.13, FLI 23–24), not obscuring torular lobes in full-face view; head roughly oval-shaped (CI 85–88), posterior margin with a slight median depression; eye of moderate size (EL 0.08–0.09, REL 16–17), oval-shaped, with 5 ommatidia at greatest diameter; face almost completely smooth and shiny, except for a few carinulae around frontal lobes and on genae, and scattered piligerous punctae; scape of moderate length (SI 95–98), not quite reaching posterior margin when laid back; scape surface mostly smooth, with faint striations, and scattered piligerous punctae; flagellum with a distinct 4–segmented antennal club; all of pronotum and most of mesonotal dorsum smooth and shiny, anepisternum rugose, katepisternum punctate, side of propodeum mostly punctate, with a few rugulae, dorsum and declivity of propodeum with transverse carinulae; promesonotum in profile low-domed, roughly symmetrical, with relatively sharp (and distinctive) transitions between anterior and dorsal faces, and between pronotum and mesonotum; metanotal groove of well-demarcated, of moderate width and depth; propodeal spines tuberculate (PSL 0.08–0.10, PSI 1.2–1.3); petiole appearing of moderate length (PL/HW 0.53–0.54); petiolar node in profile of moderate height (PH/PL 0.60–0.65), roughly symmetrical, with anterior and posterior faces almost equal in length, node dorsum rounded, but somewhat narrow, pointing vertically; postpetiole in profile subspherical, slightly smaller than petiolar node (PPH/PH 0.84–0.86); petiole and postpetiole mostly lightly punctate, with anterior faces of nodes smooth; gaster mostly smooth and shiny, with scattered piligerous punctae; most of body dorsum with thickened standing pilosity; pilosity on gastral tergites forming a layer of stout suberect setae, and a sparse layer of decumbent setae; setae on scapes dense, decumbent to appressed; setae on legs mostly appressed, with a few suberect to subdecumbent setae on femoral venters and coxae.

#### Queen.

Unknown.

#### Male.

Unknown.

#### Biology.

This species is recorded from a single Berlese sample of sifted leaf litter collected in cloud forest at 990 m elevation.

#### Comments.

*Stenamma andersoni* should be easy to separate from similar species by its smooth head and pronotum, unique pronotum shape, and thickened gastral setae.

*Stenamma andersoni* is known from only two specimens collected in 1987. As a result the specimens are somewhat faded in color and were not useable for molecular phylogenetic work. With morphology alone, it is not clear to which species *Stenamma andersoni* is most closely related, but I hypothesize that it is probably near *Stenamma crypticum* or *Stenamma huachucanum*.

#### Material examined.

Known only from the type locality.

### Stenamma atribellum sp. n.

urn:lsid:zoobank.org:act:D989706A-4AFF-47FB-AC6A-6F6C0718E78B

http://species-id.net/wiki/Stenamma_atribellum

**Figure 52.**
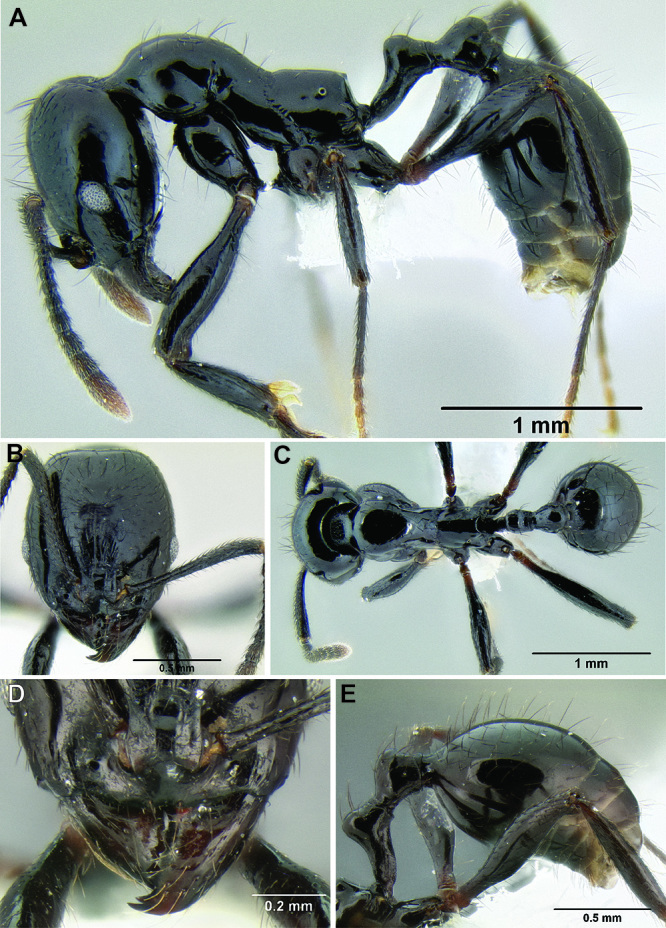
*Stenamma atribellum* holotype worker (CASENT0622351) **A** Profile **B** Face **C** Dorsum **D **Anterior clypeal margin in anterodorsal view **E** Gaster.

**Figure 53.**
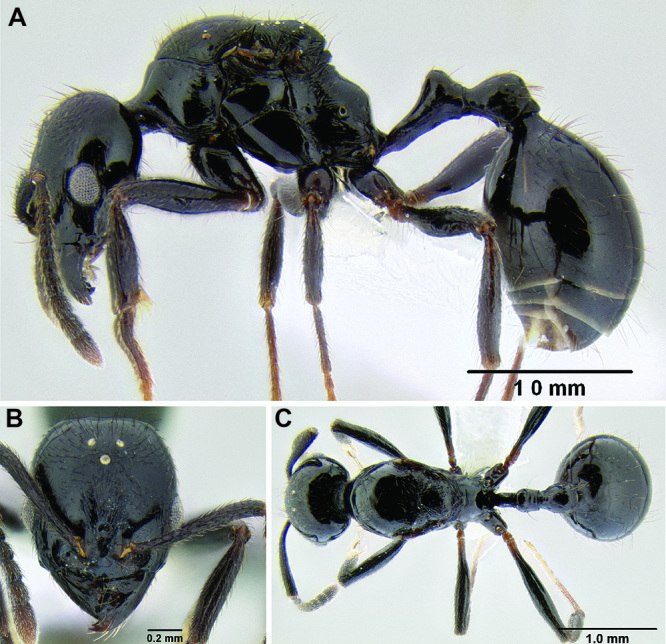
*Stenamma atribellum* paratype queen (CASENT0622349) **A** Profile **B** Face **C** Dorsum.

Worker: Figure 52 ; Queen: Figure 53 ; Map: Figure 50

#### Type material.

*Holotype worker*. HONDURAS: Cortés, Parque Nacional Cusuco, 15.50739°N, 88.23373°W ±20m, 2030m, 3 Jun 2010, cloud forest, nest under bark of log (M. G. Branstetter, collection MGB1606) [USNM, specimen CASENT0622351]. *Paratypes*: same data as holotype [1w, CAS, CASENT0623237], [1w, EAPZ, CASENT0623238], [1w, ECOSCE, CASENT0623239], [1w, FMNH, CASENT0623240], [1w, ICN, CASENT0623241], [1w, INBio, CASENT0623242], [1w, JTLC, CASENT0623525], [1w, LACM, CASENT0623243], [1w, MGBPC, CASENT0623525], [1w, MCZ, CASENT0623244], [1w, MZSP, CASENT0623245], [1w, UCD, CASENT0623246], [1w, UNAM, CASENT0623247], [1dq, 1w, USNM, CASENT0622349], [1w, UVGC, CASENT0623077].

#### Worker diagnosis.

Integument mostly black; medium- to large-sized species (see HL, ML, PrW below); gaster with elongate anterior constriction; entire body almost completely smooth and shiny, with only some faint carinulae and punctae; anterior clypeal margin with a median emargination; basal margin of mandible straight, without a basal notch or deep depression; eye relatively large (EL 0.16–0.20, REL 21–24), oval-shaped, with 9–10 ommatidia at greatest diameter; propodeal spines reduced to very small tubercles (PSL 0.10–0.13, PSI 0.9–1.0); gastral pilosity forming a layer of somewhat stout suberect setae, and a very sparse layer of short decumbent setae; frontal lobes of moderate width (FLD 0.19–0.22, FLI 24–26), not completely obscuring torular lobes in full-face view. *Similar species*: *Stenamma alas*, *Stenamma callipygium*, *Stenamma expolitum*.

#### Geographic range.

Honduras.

#### Worker description.

(10 measured) HL 0.88–0.99 (0.94), HW 0.77–0.88 (0.86), FLD 0.19–0.22 (0.22), PCW 0.04–0.06 (0.04), SL 0.77–0.85 (0.83), EL 0.16–0.20 (0.18), ACL 0.66–0.71 (0.69), ML 1.19–1.32 (1.26), PrW 0.52–0.58 (0.55), PSL 0.10–0.13 (0.12), SDL 0.11–0.13 (0.11), PL 0.43–0.47 (0.45), PH 0.25–0.28 (0.27), PW 0.19–0.21 (0.20), PPL 0.27–0.31 (0.30), PPH 0.25–0.27 (0.26), PPW 0.25–0.28 (0.26), MFL 1.00–1.13 (1.08), MTL 0.77–0.87 (0.82), CI 87–92 (92), SI 94–101 (96), REL 21–24 (21), FLI 24–26 (25), PSI 0.9–1.0 (1.0), MFI 71–84 (80), ACI1 62–66 (63), ACI2 82–91 (83).

Medium- to large-sized species; general body color mostly black, with patches of dark brown; appendages black to orange-brown, lighter at extremities; setae dark brown; mandible with 6 teeth, but two teeth nearest basal tooth usually worn and indistinct; basal margin of mandible straight, without basal notch or deep depression; mandible mostly smooth and shining, with scattered piligerous punctae; anterior clypeal margin with a median emargination; median lobe of clypeus smooth and shiny, lacking carinae, remainder of clypeus smooth and shiny; posterior extension of clypeus between antennal insertions of moderate to wide width (PCW 0.04–0.06), sides slightly hourglass-shaped; frontal lobes of moderate width (FLD 0.19–022, FLI 24–26), not obscuring torular lobes in full-face view; head roughly oval-shaped, distinctly longer than broad (CI 87–92), with posterior margin slightly depressed medially; eye relatively large (EL 0.16–0.20, REL 21–24), roughly oval-shaped, with 9–10 ommatidia at greatest diameter; head almost completely smooth and shiny, with short faint longitudinal carinulae around midline of face near antennal lobes; scape relatively long (SI 94–101), just reaching posterior margin of head when laid back; scape surface smooth and shiny, except for scattered piligerous punctae; funiculus with a somewhat distinct antennal club; mesosoma almost completely smooth and shining, except for shallow furrows along metanotal grove, and scattered piligerous punctae; promesonotum in profile low-domed and asymmetrical, with the apex slightly anterior of midpoint; metanotal grove shallow, but distinct; propodeal spines reduced to small tubercles (PSL 0.10–0.13, PSI 0.9–1.0); petiole of moderate length (PL 0.43–0.47, PL/HW 0.52–0.57); petiolar node in profile nearly symmetrical, and of moderate height (PH/PL 0.57–0.60), dorsum smoothly rounded; postpetiole bulging and distinctly wider than petiole (PW/PPW 0.72–0.78), anterior face long and shield-like, posterior face short and truncate; petiole and postpetiole almost completely smooth and shining, with some faint punctae confined mostly to the ventral surfaces; gaster with an elongate anterior constriction and with faint dorsal striae, remainder of gaster smooth and shiny, except for piligerous punctae; most of body dorsum with a layer of moderately long and stout standing pilosity; scape with suberect to decumbent setae; gaster with a layer of suberect setae, and a very sparse layer of short decumbent setae; legs with mostly appressed setae, but some suberect setae on coxae and femoral venters.

#### Queen description.

(1 measured) HL 0.99, HW 0.89, FLD 0.24, PCW 0.06, SL 0.85, EL 0.26, ACL 0.72, ML 1.53, PrW 0.83, PSL 0.16, SDL 0.15, PL 0.58, PH 0.34, PW 0.25, PPL 0.35, PPH 0.32, PPW 0.33, MFL 1.16, MTL 0.89, CI 90, SI 95, REL 29, FLI 27, PSI 1.1, MFI 77, ACI1 62, ACI2 85.

Same as worker except for standard queen modifications and as follows: face with a few distinct carinulae extending from frontal lobes to ocelli; mesoscutum, near posterior margin, and scutellum, with some longitudinal carinulae/rugulae.

#### Male.

Unknown.

#### Biology.

This species is a cloud forest specialist ranging from 1550–2030 m elevation, and is known from one leaf litter sample and one nest collection. The nest was collected underneath the bark of a large log in cloud forest near the edge of dwarf forest. The entire nest was not censused, but it was relatively large, with at least 100 workers, a single queen and brood.

#### Comments.

*Stenamma atribellum* is a distinctive species that should not be confused with any other MAC species. Molecular phylogenetic data show that it is sister to *Stenamma callipygium*, which is the only other *Stenamma* species to have an elongate anterior gastral constriction (Branstetter unpublished data). This character joins *Stenamma atribellum* and *Stenamma callipygium* in the *atribellum* species group. *Stenamma atribellum* can be separated from *Stenamma callipygium* by its completely smooth and shiny sculpture and emarginate anterior clypeal margin. Furthermore, these species have not been collected in sympatry and both appear to be narrow endemics.

*Stenamma atribellum* might be confused with the superficially similar *Stenamma alas* and *Stenamma expolitum*, which are both mostly smooth and shiny. However, these latter species do not have the anterior constriction of the gaster elongate and they tend to have more facial sculpture (especially in *Stenamma alas*). These species are also geographically separated from each another.

#### Material examined.

**HONDURAS:** ***Cortés***: Parque Nacional Cusuco, 15.50739°N, 88.23373°W, 2030m, 3 Jun 2010 (M. G. Branstetter); 25km N Cofradia, PN Cusuco, [ca. 15.497°N, 88.227°W], 1550m, 26 Aug 1994 (S. & J. Peck).

### Stenamma brujita sp. n.

urn:lsid:zoobank.org:act:746AA110-6E0F-4B6B-8718-46F8F6ABF231

http://species-id.net/wiki/Stenamma_brujita

**Figure 54.**
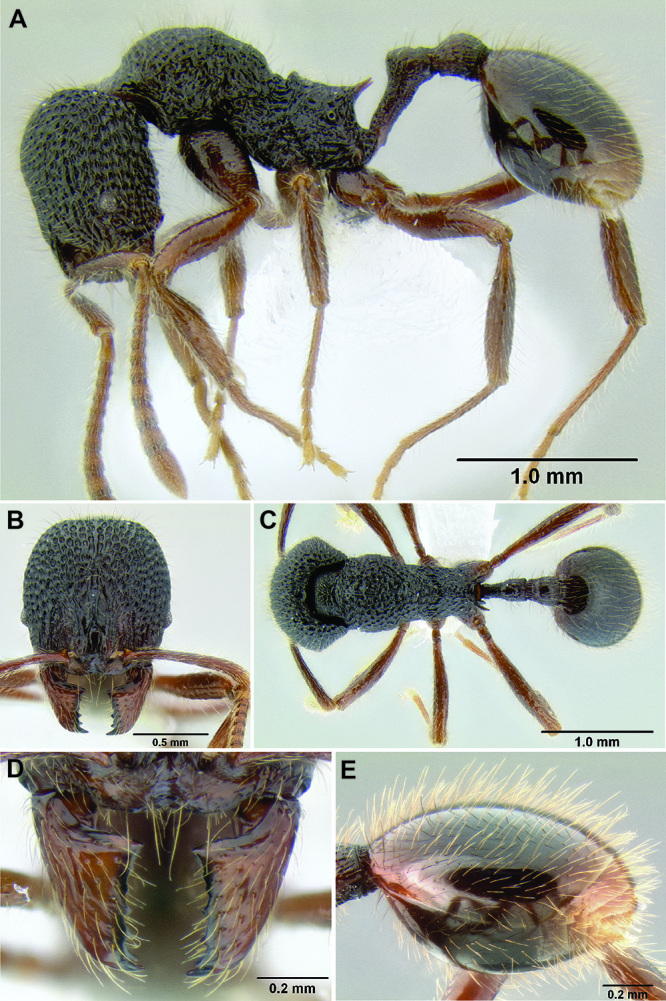
*Stenamma brujita* holotype worker (CASENT0604945) **A** Profile **B** Face **C** Dorsum **D **Anterior clypeal margin in anterodorsal view **E** Gaster.

**Figure 55.**
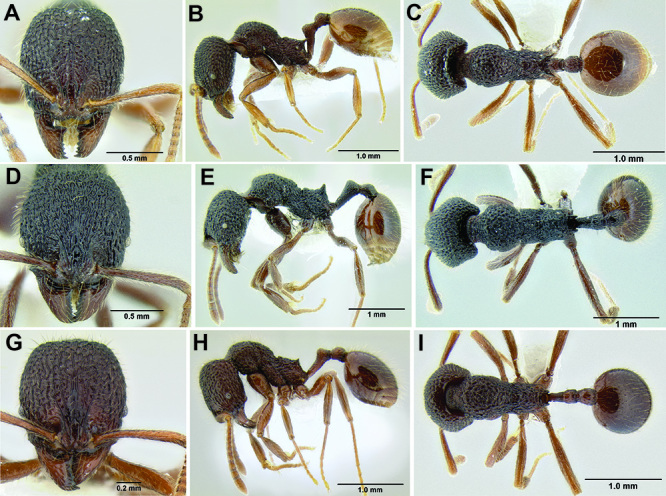
*Stenamma brujita* worker variants. Face, profile, and dorsal views **A–C** Variant 1 (CASENT0603918) **D–F** Variant 2 (CASENT0604607) **G–I** Variant 3 (CASENT0604578).

**Figure 56.**
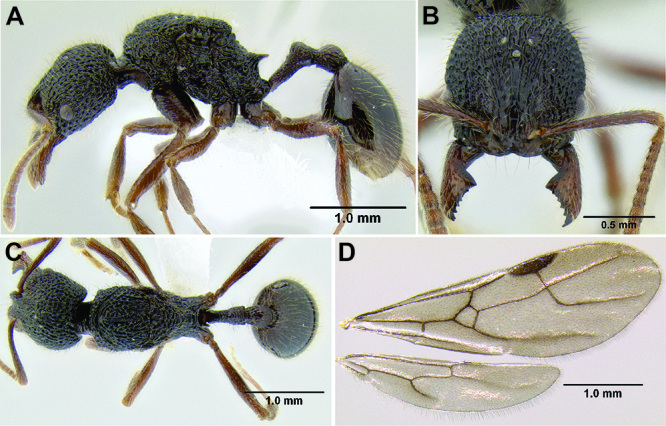
*Stenamma brujita* **A** Queen (CASENT0604991), profile **B** Same, face **C** Same, dorsum **D **Queen (CASENT0621749), wings.

**Figure 57.**
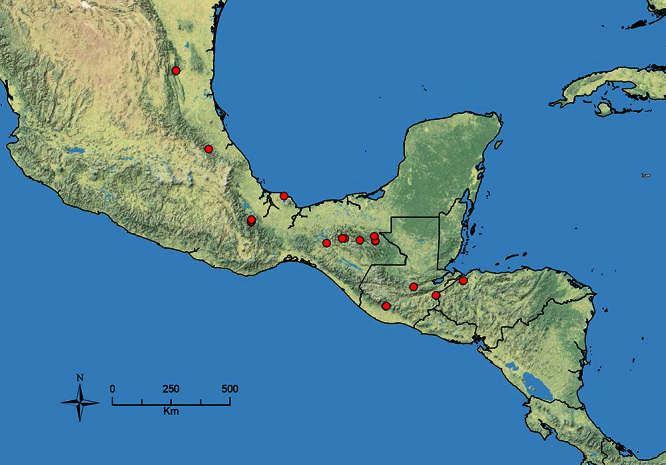
Distribution map of *Stenamma brujita*.

Worker: Figures 54 , 55 ; Queen: Figure 56 ; Map: Figure 57

Stenamma mgb05 [variant 3 below] Branstetter, 2012: phylogeny.

#### Type material.

*Holotype worker*. GUATEMALA, Zacapa: 2km SE La Unión, 14.94706°N, 89.27660°W ±50m, 1550m, 12 May 2009, cloud forest, ex sifted leaf litter (LLAMA, collection Wa-B-03-1-32) [USNM, specimen CASENT0604945]. *Paratypes*: same data as holotype but 14.94460°N, 89.27726°W ±57m, 1550m, 12 May 2009 (LLAMA, Wm-B-03-1-04), [1w, CAS, CASENT0623248], [1w, EAPZ, CASENT0623249], [1w, ECOSCE, CASENT0623250], [1w, FMNH, CASENT0623251], [1w, ICN, CASENT0623252, [1w, INBio, CASENT0623253], [1w, JTLC, CASENT0623527], [1w, LACM, CASENT0623254], [2w, MGBPC, CASENT0623528, CASENT0623529], [1w, MCZ, CASENT0623255], [1w, MZSP, CASENT0623256, [1w, UCD, CASENT0623257], [1w, UNAM, CASENT0623258], [1dq, 1w, USNM, CASENT0606239, CASENT0606656], [1w, UVGC, CASENT0623259].

#### Worker diagnosis.

Integument mostly black, red-black, or brown; medium to large-sized species (see HL, ML, PrW below); head and mesosoma foveate to coarsely rugoreticulate; eye relatively small (EL 0.09–0.13, REL 10–14), circular, and slightly bulging, with 5–7 ommatidia at greatest diameter; pilosity on gastral dorsum long, dense and mostly suberect; propodeal spines tuberculate to long and robust, usually of moderate length (PSL 0.15–0.37, PSI 1.3–2.9); anterior clypeal margin forming a single shallow to deep median emargination, or rarely, 4 blunt teeth; basal margin of mandible straight to sinuous, sometimes with a broad basal depression, but without a distinct notch or tooth; 4-segmented antennal club indistinct. *Similar species*: *Stenamma zelum*.

#### Geographic range.

Mexico (Atlantic slope) to Honduras.

#### Worker description.

(21 measured) HL 0.90–1.20 (1.05), HW 0.77–1.15 (1.00), FLD 0.21–0.32 (0.29), PCW 0.04–0.07 (0.06), SL 0.75–1.01 (0.93), EL 0.09–0.13 (0.12), ACL 0.63–0.78 (0.72), ML 1.15–1.62 (1.45), PrW 0.52–0.78 (0.70), PSL 0.15–0.37, SDL 0.08–0.16, PL 0.45–0.64 (0.56), PH 0.21–0.35 (0.27), PW 0.15–0.24 (0.21), PPL 0.21–0.31 (0.26), PPH 0.18–0.28 (0.24), PPW 0.20–0.30 (0.27), MFL 0.81–1.25 (1.10), MTL 0.66–0.95 (0.88), CI 85–96 (96), SI 85–99 (88), REL 10–14 (12), FLI 25–31 (29), PSI 1.3–2.9 (2.3), MFI 86–105 (91), ACI1 62–65 (64), ACI2 78–89 (82).

Medium to large-sized species; general body color usually red-black (type population) to black, with patches of brown, but some populations more uniformly brown; mandibles and appendages always lighter than body, brown to orange-brown; setae golden brown; mandible with 4–8 teeth (usually 7), consisting of 3–4 distinct apical teeth, a basal tooth, and a variable number of inner teeth, which are often worn and indistinct; basal tooth usually of moderate size (type population), but sometimes more robust and projecting; basal margin of mandible straight to slightly sinuous, sometimes with a shallow, broad basal depression, but without a distinct notch or tooth; mandible mostly smooth and shining, with scattered piligerous punctae and a few lateral striae; median lobe of clypeus usually slightly produced and clearly visible in full-face view (type population), but sometimes becoming obliquely flattened and angled more dorsoventrally, making it less visible; anterior clypeal margin varying from having a shallow to deep median emargination (type population), to forming 4 distinct blunt teeth; median lobe usually with a pair of faint longitudinal carinulae that diverge toward anterior margin, apex of lobe with a faint to strong transverse carinula; posterior extension of clypeus between frontal lobes of relatively moderate width (PCW 0.04–0.07), with sides subparallel to hour-glass-shaped; frontal lobes average to slightly expanded outward (FLD 0.21–0.32, FLI 25–31), with underlying torular lobes always visible in full-face view; head in full-face view roughly oval shaped to subcircular (CI 85–97), with posterior margin slightly to distinctly depressed medially; eyes relatively small (EL 0.09–0.13, REL 10–14), circular, and somewhat bulging, with 5–7 ommatidia at greatest diameter; head foveate to coarsely rugoreticulate, shiny, often with a few short costae extending back from frontal lobes, interstices with piligerous punctae; scape relatively short, not reaching posterior margin of head when laid back (SI 85–99); scape shiny, usually with only scattered piligerous punctae (type population), but sometimes more robust, with punctae deeper and broader, becoming foveolae; flagellum with indistinct 4-segmented antennal club; mesosoma robust, foveate to coarsely rugoreticulate, with foveae most prominent on promesonotal dorsum; propodeal spines varying from short tubercles to long robust spines (PSL 0.15–0.37, PSI 1.3–2.9), which are usually spiniform and project dorsoposteriorly (type population), but sometimes form robust vertical pointing triangles; promesonotum in profile varying from being domed and nearly symmetrical (type population), to domed and asymmetrical, with apex occurring anterior of midpoint, to high-domed and asymmetrical; humeral angles rounded and indistinct, to becoming produced and angulate (type population), the latter occurring when the promesonotal side is scalloped slightly inward; metanotal grove present, but variable in depth and degree of distinctness; anterodorsal margin of propodeum in profile flat to distinctly raised into a welt (type population); propodeal declivity with a variable number of transverse carinae, often mostly smooth and shiny; petiole shape in profile usually appearing relatively long and somewhat gracile (PL/HW 0.53–0.63), with a small distinct node (PH/PL 0.45–0.55) (type population), but sometimes petiole is more robust and strongly wedge-shaped, without a clear distinction between the node and peduncle; postpetiole in profile usually low-domed, nearly symmetrical, and appearing as high or slightly smaller than petiolar node (type population), but sometimes postpetiole distinctly larger than petiolar node; postpetiole in dorsal view elongate, and reaching its widest point near posterior margin; waist sculpture variable, nodes usually mostly smooth and shiny, but sometimes more punctate and/or with longitudinal costae or rugulae, ventral surface punctate, dorsal surface of peduncle punctate and with a variable number of rugulae; gaster mostly smooth and shiny, with scattered piligerous punctae, and short furrows on anterior constriction where gaster inserts into postpetiole; most of body with relatively long standing pilosity; scape either with a single layer of mostly decumbent setae, or bilayered with a sparse layer of longer suberect setae over a denser decumbent layer (type population); gastral pilosity relatively long and somewhat dense, with most setae suberect to subdecumbent; setae on legs suberect to decumbent, with some populations having predominately suberect setae (type population) and others mainly decumbent setae, longer suberect setae always present on femoral venters and coxae.

#### Queen description.

(5 measured) HL 0.96–1.03 (1.03), HW 0.91–0.99 (0.99), FLD 0.26–0.29 (0.29), PCW 0.05–0.07 (0.06), SL 0.84–0.89 (0.89), EL 0.17–0.19 (0.17) ACL 0.68–0.72 (0.71), ML 1.47–1.57 (1.57), PrW 0.76–0.82 (0.82), PSL 0.18–0.28 (0.28), SDL 0.13–0.16 (0.13), PL 0.59–0.61 (0.60), PH 0.28–0.32 (0.31), PW 0.22–0.25 (0.24), PPL 0.27–0.33 (0.32), PPH 0.23–0.28 (0.28), PPW 0.25–0.31 (0.29), MFL 1.01–1.06 (1.06), MTL 0.79–0.85 (0.85), CI 93–97 (97), SI 86–94 (90), REL 17–20 (17), FLI 28–31 (29), PSI 1.1–2.1 (2.1), MFI 87–95 (93), ACI1 63–64 (63), ACI2 80–85 (80).

Same as worker except for standard queen modifications and the following: Propodeal spines less variable (PSL 0.18–0.28, PSI 1.1–2.1), usually present, of moderate length, and thick at base (only Nahá population with spines tuberculate); setae on scape less variable, usually with a sparse layer of longer suberect setae and a layer of denser decumbent setae (only Nahá population with setae uniformly subdecumbent); wing venation as in Figure 56D.

#### Male.

Unknown.

#### Biology.

*Stenamma brujita* is known only from Winkler and Berlese samples of leaf litter collected from the floor of wet forest habitats (e.g. lowland rainforest, montane wet forest, cloud forest, pine cloud forest, oak-pine forest). The species has a broad elevational range, occurring from 200–1800 m, but it is most common at mid-elevations (1000–1500 m).

#### Comments.

The combination of large size, small eyes, and foveate sculpture make *Stenamma brujita* a very distinctive species, unlikely to be confused with any other MAC species. However, *Stenamma zelum*, which is not closely related to *Stenamma brujita* (Branstetter 2012), has converged on a similar phenotype and may cause problems with identification. Fortunately, these two species are geographically isolated from one another, with *Stenamma brujita* reaching only as far south as northwestern Honduras, and *Stenamma zelum* extending only as far north as northeastern Honduras. Using morphology, *Stenamma brujita* can be distinguished from *Stenamma zelum* by its more rounded head (rectangular in *Stenamma zelum*), longer propodeal spines (PSI 1.3–2.9 vs. 1.0–1.3), and lower FLI (27–31 vs. 31–34). In addition, the anterior clypeal margin forms four sharp teeth in *Stenamma zelum*, with the outer teeth usually strongly projecting. In contrast, most populations of *Stenamma brujita* have the anterior clypeal margin forming a single median emargination (all Honduras populations like this), and in those specimens that do have clypeal teeth, the teeth are all blunt.

*Stenamma brujita* is quite variable throughout its range and may comprise a complex of several species. I choose to identify a single species here, because every population exhibits some amount of variation, none of the variants occur in sympatry, and some populations have intermediate phenotypes. I do however, identify three main variants that differ significantly from the holotype population.

Variant 1 (Figure 54A–C) includes all collections from Tamaulipas and Hidalgo, Mexico. It has the following features: body sculpture more rugoreticulate than foveate; propodeal spines long, straight and more slender; petiole with a distinct concavity below node.

Variant 2 (Figure 55D–F), the most distinctive variant, is known from a few collections taken on the wet Atlantic slope of the Sierra Juarez, between Oaxaca and Valle Nacional in Mexico. It has the following features: body very large; general body color very dark, mostly black; petiole wedge-shaped, usually without a distinct node; propodeal spines forming robust, blunt-tipped triangles, which point almost vertically.

Variant 3 (Figure 56G–I) occurs at several localities in Chiapas, México, mainly Nahá and Lago Metzabok. It is characterized by the following: general body color brown; promesonotum in profile high-domed, and asymmetrical; propodeal spines tuberculate; petiole in profile appearing more gracile with node reduced in size; anterior clypeal margin forming 4 blunt teeth.

Some additional populations in Chiapas, Mexico, and all of the populations in Guatemala and Honduras, are most similar to the holotype population (La Unión, Guatemala). However, there is considerable variation among populations and some have character states that are intermediate between the holotype form and the different variants just described. Specimens from higher elevations tend to be larger, darker and more robust. The specimens from Purulhá, Guatemala appear especially robust-looking, with very long, sinuous propodeal spines and coarser sculpturing. Interestingly, these specimens have the petiolar node more wedge-shaped, similar to variant 2. Key character states of the holotype population are indicated in the worker description.

#### Material examined.

**GUATEMALA:** ***Baja Verapaz***: 7.3km E Purulhá, [ca. 15.267°N, 90.132°W], 1700m, 19 May 1991 (R. S. Anderson); ***Suchitepéquez***: Finca Sn. Jerónimo, 14.55914°N, 91.16705°W, 1790m, 11 Dec 2010 (L. Sáenz); Volcán Atitlán, 10km SE Santiago Atitlán, 14.552°N, 91.193°W, 1690m, 10 Sep 2008 (M. G. Branstetter); 4km S Vol. Atitlán, 14.55195°N, 91.192333°W, 1750m, 15 Jun 2009 (LLAMA); ***Zacapa***: 2km SE La Unión, 14.94706°N, 89.27660°W, 1550m, 12 May 2009 (LLAMA); 2km SE La Unión, 14.95463°N, 89.27721°W, 1430m, 12 May 2009 (LLAMA); 3.5km SE La Union, 14.95000°N, 89.26667°W, 1500m, 6 Jun 1991 (R. S. Anderson); **HONDURAS:** ***Cortés***: Parque Nacional Cusuco, 15.48710°N, 88.23469°W, 1330m, 30 May 2010 (LLAMA); Parque Nacional Cusuco, 15.48940°N, 88.23584°W, 1290m, 30 May 2010 (LLAMA); 25km N Cofradia, PN Cusuco, [ca. 15.497°N, 88.227°W], 1550m, 26 Aug 1994 (S. & J. Peck); **MÉXICO: *Chiapas***: 10km W El Bosque, [ca. 17.0440°N, 92.8612°W], 1475m, 15 Sep 1992 (R. S. Anderson); 10.6km W El Bosque, [ca. 17.043°N, 92.762°W], 1460m, 25–29 Aug 1973 (A. F. Newton); Lago Metzabok, 17.12562°N, 91.63086°W, 570m, 6 Jun 2008 (LLAMA); Nahá, 16.96358°N, 91.59332°W, 985m, 8 Jun 2008 (LLAMA); Nahá, 16.94864°N, 91.59383°W, 930m, 8 Jun 2008 (LLAMA); 12.5km NW Ocosingo, [ca. 16.983°N, 92.183°W], 1400m, 16 Sep 1992 (R. S. Anderson); 19km NW Ocozocoautla, [ca. 16.877°N, 93.458°W], 975m, 4-5 Sep 1973 (A. F. Newton); ***Hidalgo***: 11km SW Chapulhuacán, [ca. 21.147°N, 98.966°W], 1200m, 5 Jul 1976 (A. F. Newton; ***Oaxaca***: 10km S Valle Nacional, [ca. 17.724°N, 96.324°W], 650m, 19 May 1971 (S. B. Peck); 13.2km SW Valle Nacional, 17.65934°N, 96.33426°W, 1360m, 11 Aug 2009 (M. G. Branstetter); 25km S Valle Nacional, [ca. 17.670°N, 96.330°W], 1200m, 21 May 1971 (S. B. Peck); 26km S Valle Nacional, km 71, [ca. 17.645°N, 96.336°W], 1220m, 25 Jun 1983 (S. & J. Peck); ***Puebla***: 24km N Xicotepec de Juarez, [ca. 20.282°N, 97.963°W], 1070m, 17 Jun 1983 (R. S. Anderson); ***Tamaulipas***: El Cielo, nr Alta Cima, 23.06518°N, 99.20433°W, 980m, 21 Aug 2009 (L. Sáenz); 1.8km W Alta Cima, 23.06110°N, 99.21564°W, 1340m, 23 Aug 2009 (M. G. Branstetter); nr Gomez Farias Rancho del Cielo, [ca. 23.063°N, 99.205°W], 1000m, 7 Aug 1983 (S. & J. Peck) ***Veracruz***: Los Tuxtlas, 10km NNW Sontecomapan, 18.583°N, 95.083°W, 200m, 20 Mar 1985 (P. S. Ward); Los Tuxtlas, 10km NNW Sontecomapan, 18.583°N, 95.083°W, 500m, 21 Mar 1985 (P. S. Ward).

### Stenamma callipygium sp. n.

urn:lsid:zoobank.org:act:A948932E-F687-4A9B-8772-DC26DEE8603A

http://species-id.net/wiki/Stenamma_callipygium

**Figure 58.**
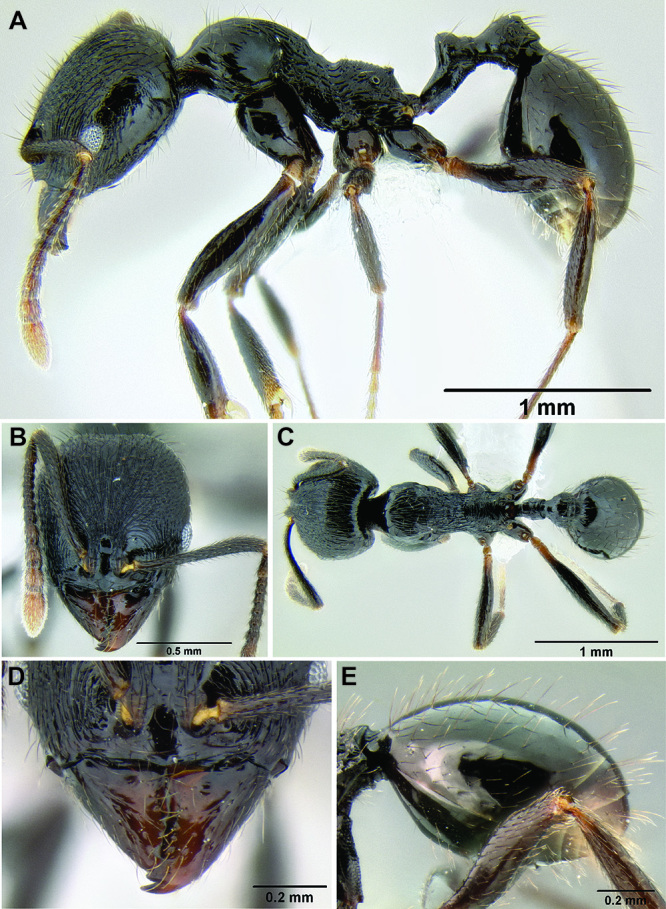
*Stenamma callipygium* holotype worker (CASENT0606207) **A** Profile **B** Face **C** Dorsum **D **Anterior clypeal margin in anterodorsal view **E** Gaster.

**Figure 59.**
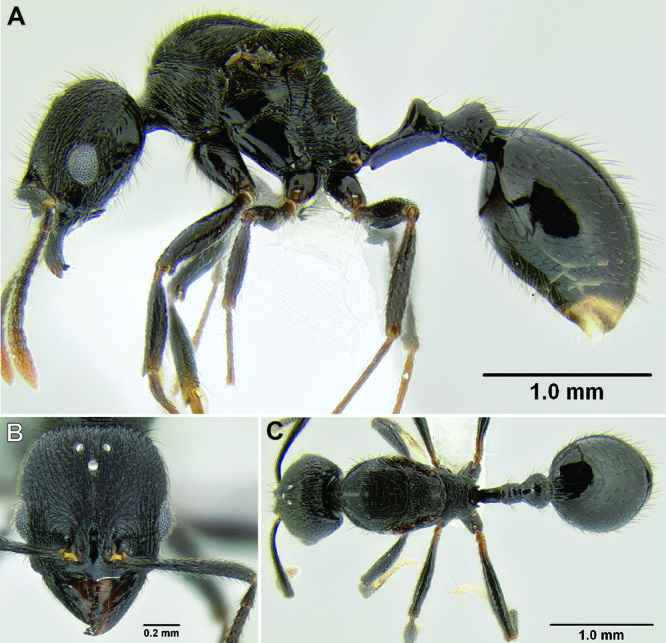
*Stenamma callipygium* paratype queen (CASENT0603928) **A** Profile **B** Face **C** Dorsum.

**Figure 60.**
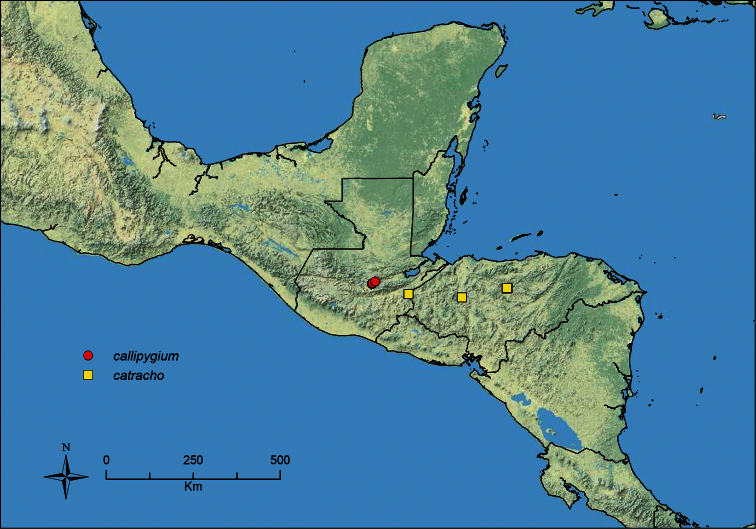
Distribution map of *Stenamma callipygium* (circles) and *Stenamma catracho* (squares).

Worker: Figure 58 ; Queen: Figure 59 ; Map: Figure 60

Stenamma mgb24 Branstetter, 2012: phylogeny.

#### Type material.

*Holotype worker*. GUATEMALA: Baja Verapaz, Biotopo Quetzal, 15.21329°N, 90.21516°W ±110m, 1715m, 8 May 2009, cloud forest, beating vegetation (LLAMA, collection Go-B-02-1-02) [USNM, specimen CASENT0606207]. *Paratypes*: same data as holotype [1w, CAS, CASENT0606206]; same data but (LLAMA, Go-B-02-1-01) [1w, MCZ, CASENT0606203]; 15.21227°N, 90.21430°W ±50m, 1750m, 7 May 2008 (LLAMA, Wa-B-02-1-37) [1w, MGBPC, CASENT0604923]; 15.21278°N, 90.21552°W ±10m, 1720m, 7–10 May 2009 (LLAMA, Ft-B-02-1-01) [1w, UCD, CASENT0606227]; 15.21202°N, 90.21653°W ±207m, 1735m, 8 May 2009 (LLAMA, Ba-B-02-1-07-05) [1dq, USNM, CASENT0603928]; GUATEMALA: Baja Verapaz, Ranchito El Quetzal, 15.21508°N, 90.22003°W, 1700m, 20 Sep 2008 (R. S. Anderson, RSA2008-139) [1w, UVGC, CASENT0606095].

#### Worker diagnosis.

Integument mostly black; medium-sized species (see HL, ML, PrW below); gaster with an elongate anterior constriction, with anterolateral margins of gaster forming hard shoulder-like angles (best viewed dorsally); median lobe of the clypeus projecting out over mandibles, forming a well-defined, blunt apex; basal margin of mandible sinuous, but without a basal notch or deep depression; basal third of mandible distinctly attenuated (dorsoventrally thinned); face with dense fan of carinulae extending to posterior and lateral margins of head; eye large (EL 0.14–0.19, REL 20–24), oval-shaped, with 9–10 ommatidia at greatest diameter; propodeal spines tuberculate (PSL 0.08–0.10, PSI 1.0–1.4); setae on gastral tergites moderately long and sparse, mostly suberect; frontal lobes of moderate width (FLD 0.16–0.21, FLI 22–27), not covering torular lobes in full-face view. *Similar species*: *Stenamma atribellum*.

#### Geographic range.

Guatemala.

#### Worker description.

(10 measured) HL 0.69–0.93 (0.85), HW 0.60–0.90 (0.82), FLD 0.16–0.21 (0.20), PCW 0.03–0.06 (0.05), SL 0.59–0.78 (0.75), EL 0.14–0.19 (0.18), ACL 0.55–0.66 (0.65), ML 0.93–1.23 (1.13), PrW 0.41–0.56 (0.51), PSL 0.08–0.10 (0.10), SDL 0.06–0.10 (0.10), PL 0.30–0.42 (0.38), PH 0.19–0.26 (0.24), PW 0.41–0.56 (0.51), PPL 0.20–0.27 (0.27), PPH 0.19–0.26 (0.24), PPW 0.21–0.32 (0.28), MFL 0.71–0.98 (0.93), MTL 0.55–0.77 (0.73), CI 86–97 (96), SI 86–98 (91), REL 20–24 (21), FLI 22–27 (24), PSI 1.0–1.4 (1.0), MFI 84–92 (89), ACI1 62–68 (63), ACI2 84–94 (87).

Medium-sized species; general body color black to dark brown, with appendages brown to orange-brown; setae dark brown; mandible with 6–7 teeth, consisting of 3–4 distinct apical teeth, an indistinct basal tooth, and 2–3 inner denticles; basal third of mandible distinctly attenuated (dorsoventrally thinned), with masticatory and basal margins somewhat elongated, attenuated section bordered by an oblique carina; basal margin of mandible sinuous, but without a basal notch or distinct depression; mandible surface mostly smooth and shiny, with scattered piligerous punctae; anterior clypeal margin reduced and mostly hidden underneath the median lobe; median lobe of clypeus projecting out over mandibles, forming a well-defined, blunt apex (almost tooth-like), clypeal carinae absent, remainder of clypeus mostly smooth and shiny; posterior extension of clypeus between antennal insertions of moderate to wide width (PCW 0.03–0.06), sides subparallel; frontal lobes of moderate width (FLD 0.16–0.21, FLI 22–27), but not completely obscuring torular lobes in full-face view; head usually robust and somewhat heart-shaped (CI 86–97), with posterior margin broadly depressed medially; eye large (EL 0.14–0.19, REL20–24), oval-shaped, with 9–10 ommatidia at greatest diameter; face densely sculptured with a fan of longitudinal carinulae that extend to the posterior and lateral margins, area between eye and antennal insertion with shorter irregular rugulae, interstices near lateral margins faintly punctate; scape of moderate length (SI 86–98), just reaching posterior margin of head when laid back; scape surface mostly smooth and shiny, with scattered piligerous punctae and a few striations; flagellum with somewhat distinct 4-segmented antennal club; side and anterior half of pronotum mostly smooth and shiny, with a few scattered rugulae; remainder of promesonotal dorsum longitudinally carinate; mesopleuron and side of propodeum with scattered rugulae and faint punctae; propodeal dorsum and declivity with transverse carinae; promesonotum in profile low-domed and somewhat asymmetrical, with apex occurring anterior of midpoint and the anterior slope longer and steeper than posterior slope; metanotal grove present, somewhat shallow; propodeal spines forming small, but sharp tubercles (PSL 0.08–0.10, PSI 1.0–1.4); petiole of moderate length and form (PL/HW 0.44–0.51); petiolar node in profile somewhat small (PH/PL 0.61–0.67), gently rounded to subquadrate, and slightly angled posteriad, anterior slope distinctly longer than posterior slope; postpetiole bulging, distinctly wider than petiole (PW/PPW 0.60–0.71), and anterior gastral constriction (most noticeable in dorsal view), anterior face of node in profile long and shield-like, posterior face truncate; anterior faces of petiolar and postpetiolar nodes smooth and shiny, posterior faces with a few rugulae and punctae; ventral surface of waist segments faintly punctate; anterior constriction of gaster distinctly elongate and with elongate dorsal striae; gaster in dorsal view with shoulder-like anterolateral corners where anterior constriction begins; remainder of gaster mostly smooth and shiny, with scattered piligerous punctae; most of body dorsum with long standing pilosity; setae on scape subdecumbent to appressed, of roughly uniform length; gastral pilosity somewhat stout and mostly forming a sparse layer of suberect to subdecumbent setae; setae on legs mostly decumbent to appressed, with suberect setae on coxae and femoral venters.

#### Queen description.

(1 measured) HL 0.88, HW 0.81, FLD 0.21, PCW 0.05, SL 0.77, EL 0.25, ACL 0.68, ML 1.38, PrW 0.76, PSL 0.14, SDL 0.12, PL 0.50, PH 0.29, PW 0.21, PPL 0.27, PPH 0.29, PPW 0.35, MFL 0.98, MTL 0.80, CI 92, SI 95, REL 30, FLI 26, PSI 1.2, MFI 83, ACI1 64, ACI2 88.

Same as worker except for standard queen modifications and as follows: mesosoma almost completely carinulate, only mesopleuron mostly smooth; pronotum with transverse carinulae; pronotum with transverse carinulae that wrap around entire surface; mesoscutum and scutellum with longitudinal carinae; anterior of mesoscutum with a short narrow strip of smooth surface from which carinulae arise; gastral setae slightly more dense.

#### Male.

Unknown.

#### Biology.

*Stenamma callipygium* has been collected by sifting leaf litter, beating vegetation and baiting with cookie bait. In addition, a single worker was found in a flight intercept trap placed underneath a Malaise trap. This species is a cloud forest specialist known from 1630–1750 m elevation. Very few *Stenamma* species are known from beating samples, suggesting that this species may nest or at least forage arboreally. The diversity of collecting methods that have retrieved this species suggests that it is an active forager. It is surprising, however, that out of the over 100 leaf litter samples collected at the Biotopo Quetzal by the LLAMA project, only one sample had *Stenamma callipygium*.

The oddly shaped clypeus and mandible of this species is unique within *Stenamma* and begs for explanation. Most likely the projecting clypeal tooth is used for the capture or maceration of a specific prey type.

#### Comments.

*Stenamma callipygium* and *Stenamma atribellum* are sister species and make up the *atribellum* species group. The diagnostic character state of this group is the elongate gastral constriction. *Stenamma callipygium* is easy to distinguish from *Stenamma atribellum* by its median clypeal tooth and carinulate sculpture.

#### Material examined.

**GUATEMALA:** ***Baja Verapaz***: Biotopo Quetzal, 15.21329°N, 90.21516°W, 1715m, 8 May 2009 (LLAMA); Biotopo Quetzal, 15.21227°N, 90.21430°W, 1750m, 7 May 2009 (LLAMA); Ranchito El Quetzal, 15.21508°N, 90.22003°W, 1700m, 20 Sep 2008 (R. S. Anderson); 4.5km E Purulhá, [ca. 15.226°N, 90.200°W], 1630m, 21 May 1991 (R. S. Anderson); 7.3km E Purulhá, [ca. 15.2667°N, 90.132°W], 1700m, 19 May 1991 (R. S. Anderson).

### Stenamma catracho sp. n.

urn:lsid:zoobank.org:act:6D8C5D59-F8F2-4C8A-8D0B-1A543DF13706

http://species-id.net/wiki/Stenamma_catracho

**Figure 61.**
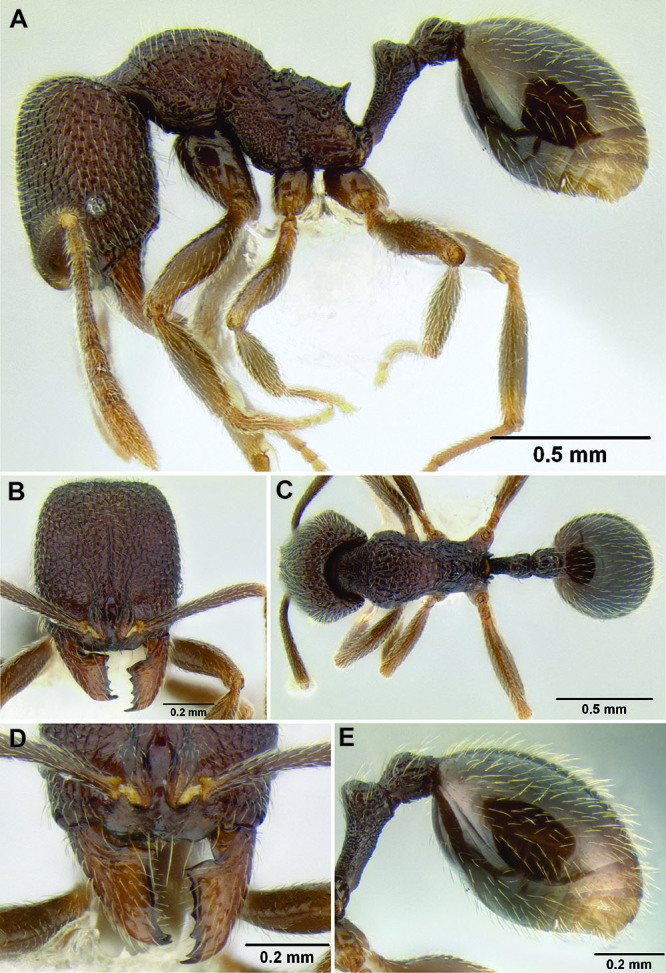
*Stenamma catracho* holotype worker (CASENT0621306) **A** Profile **B** Face **C** Dorsum **D **Anterior clypeal margin in anterodorsal view **E** Gaster.

**Figure 62.**
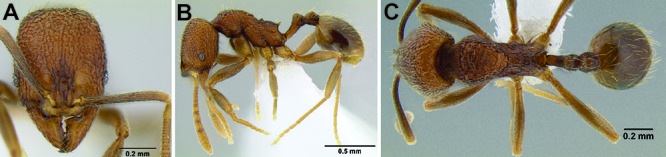
*Stenamma catracho* worker variant (CASENT0621563) **A** Face **B** Profile **C** Dorsum.

**Figure 63.**
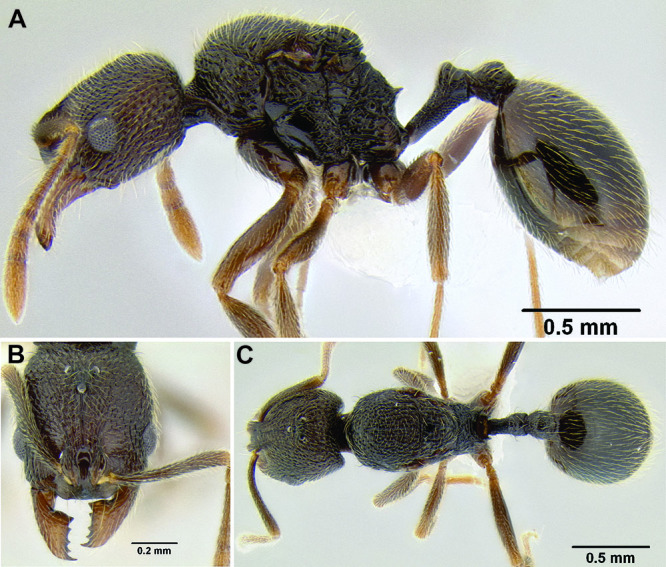
*Stenamma catracho* paratype queen (CASENT0621305) **A** Profile **B** Face **C** Dorsum.

Worker: Figures 61 , 62 ; Queen: Figure 63 ; Map: Figure 60

#### Type material.

*Holotype worker*. HONDURAS: Olancho, Parque Nacional La Muralla, 15.09965°N, 86.74072°W ±20m, 1530m, 2 May 2010, cloud forest, ex sifted leaf litter (LLAMA, collection Wa-C-01-1-36) [USNM, specimen CASENT0621306]. *Paratypes*: same data as holotype [1dq, 1w, USNM, CASENT0621305, CASENT0621307], [1w, CAS, CASENT0621308]; same data as holotype but 15.09925°N, 86.74063°W ±20m, 1530m, 2 May 2010 (LLAMA, Wa-C-01-1-27) [1w, EAPZ, CASENT0621298], [1w, ECOSCE, CASENT0621299], [1w, FMNH, CASENT0621297], [1w, ICN, CASENT0621300]; 15.09859°N, 86.74216°W ±10m, 1510m, 2 May 2010 (LLAMA, Wm-C-01-1-03) [1w, INBio, CASENT0622022], [1w, JTLC, CASENT0622023], [1w, LACM, CASENT0622024], [1w, MGBPC, CASENT0623530], [1w, MCZ, CASENT0623531], [1w, MZSP, CASENT0623532], [1w, UCD, CASENT0623533]; 15.09694°N, 86.74533°W ±10m, 1460m, 5 May 2010 (LLAMA, Wm-C-01-1-09) [1w, UNAM, CASENT0622048], [1w, UVGC, CASENT0623534].

#### Worker diagnosis.

Integument mostly dark red-brown to orange-brown; small-sized species (see HL, ML, PrW below); anterior clypeal margin viewed from anterodorsal angle undulating (straight in full-face view), appearing as 2–4 blunt teeth; basal margin of mandible sinuous, with a shallow, but distinct basal depression; head and mesosoma densely sculptured, mostly rugoreticulae; gastral pilosity short, relatively dense, and usually appearing uniformly suberect to subdecumbent, but sometimes more clearly bilayered; petiole somewhat long and gracile, with node relatively small; petiolar and postpetiolar nodes smooth only on anterior faces; eye relatively small (EL 0.08–0.09, REL 14–16), subcircular to oval-shaped, with 4–5 ommatidia in greatest diameter; frontal lobes moderately expanded (FLD 0.15–0.18, FLI 28–33), mostly to completely covering torular lobes in full-face view; propodeal spines short to medium length (PSL 0.11–0.18, PSI 1.6–2.1). *Similar species*: *Stenamma crypticum*, *Stenamma cusuco*, *Stenamma hojarasca*.

#### Geographic range.

Guatemala to Honduras.

#### Worker description.

(9 measured) HL 0.61–0.68 (0.68), HW 0.51–0.59 (0.58), FLD 0.15–0.18 (0.18), PCW 0.01–0.03 (0.03), SL 0.52–0.55 (0.55), EL 0.08–0.09 (0.08), ACL 0.51–0.54 (0.53), ML 0.75–0.82 (0.82), PrW 0.36–0.41 (0.41), PSL 0.11–0.12 (0.12), SDL 0.05–0.07 (0.07), PL 0.28–0.32 (0.32), PH 0.15–0.17 (0.17), PW 0.12–0.13 (0.13) PPL 0.13–0.15 (0.15), PPH 0.13–0.15 (0.15), PPW 0.15–0.16 (0.16), MFL 0.57–0.63 (0.62), MTL 0.47–0.51 (0.51), CI 82–88 (86), SI 92–104 (94), REL 14–16 (14), FLI 28–33 (32), PSI 1.6–2.1 (1.8); MFI 84–95 (93), ACI1 68–71 (68), ACI2 96–102 (96).

Small species; general body color dark red-brown to orange-brown, with patches of dark brown to brown on gaster; appendages lighter, brown to yellow-brown; setae golden brown; mandible with 6 teeth, consisting of 3 distinct apical teeth, a basal tooth, and 2 middle teeth, which are often worn and indistinct; basal margin of mandible sinuous, with a shallow, but distinct basal depression, accompanied by attenuation of cuticle where mandible fits underneath clypeus; mandible mostly smooth and shining, with scattered piligerous punctae and basal striae; anterior clypeal margin viewed at anterodorsal angle undulating (straight in full-face view), appearing as 2–4 blunt teeth; median lobe of clypeus somewhat flattened, longitudinal carinulae absent or very faint, apex with a short transverse carinula; remainder of clypeus mostly smooth and shiny; posterior extension of clypeus between antennal insertions narrow (PCW 0.01–0.03), sides diverging posteriad; frontal lobes moderately expanded (FLD 0.15–0.18, FLI 28–33), with lateral apices shifted slightly posteriad of torular lobes, which are mostly to completely covered in full-face view; head subrectangular to oval-shaped (CI 82–88), posterior margin slightly depressed medially; eye relatively small (EL 0.08–0.09, REL 14–16), subcircular to oval-shaped, with 4–5 ommatidia in greatest diameter; head mostly rugoreticulate, with a few longitudinal rugae along midline, interstices faintly punctate; scape relatively long (SI 94–104), but variable, either reaching or not quite reaching posterior margin when laid back; scape surface shiny, but somewhat rough, with punctae and faint striae; flagellum with distinct 4-segmented antennal club; mesosoma densely sculptured, except for propodeal declivity, which only has a few faint transverse carinulae; promesonotal dorsum rugose to rugoreticulae, interstices faintly punctate; mesosomal side mostly punctate, with a few rugulae; propodeal dorsum with a few transverse carinae; promesonotum in profile, low-domed, roughly symmetrical; metantoal groove distinct, but somewhat shallow; anterodorsal margin of propodeum in profile with a small welt; propodeal spines present, short to medium length (PSL 0.11–0.18, PSI 1.6–2.1); petiole somewhat long and gracile (PL/HW 0.53–0.59); petiolar node in profile relatively small (PH/PL 0.49–0.59), asymmetrical, with anterior face long and sloping and posterior face short and nearly vertical, dorsum of node rounded, apex pointing posteriad; postpetiole relatively small (PPH/PH 0.85–0.91), somewhat dorsoventrally compressed; anterior faces of petiolar and postpetiolar nodes smooth and shiny, remainder of waist mostly punctate, with a few small rugulae around postpetiolar node; gaster mostly smooth and shiny, with scattered piligerous punctae; most of body dorsum with short to moderately long standing pilosity; scape with suberect to decumbent setae; gastral pilosity variable, usually short, relatively dense, and uniformly suberect to subdecumbent, but sometimes clearly bilayered, with a longer suberect layer, and a shorter decumbent layer; setae on legs mostly decumbent to appressed, with some suberect setae on coxae and venter of profemur.

#### Queen description.

(3 measured) HL 0.67–0.69 (0.69), HW 0.59–0.62 (0.61), FLD 0.16–0.21 (0.21), PCW 0.02–0.04 (0.02), SL 0.56–0.58 (0.56), EL 0.16–0.18 (0.17), ACL 0.54–0.57 (054), ML 0.92–1.00 (0.95), PrW 0.50–0.55 (0.53), PSL 0.15–0.16 (0.15), SDL 0.08–0.09 (0.09), PL 0.36–0.38 (0.38), PH 0.18–0.20 (0.19), PW 0.15–0.16 (0.15), PPL 0.15–0.17 (0.15), PPH 0.18–0.19 (0.18), PPW 0.19–0.20 (0.19), MFL 0.63–0.69 (0.63), MTL 0.53–0.58 (0.53), CI 88–90 (88), SI 92–96 (92), REL 27–30 (27), FLI 25–34 (34), PSI 1.6–2.1 (1.6), MFI 88–97 (97), ACI1 67–69 (69), ACI2 96–98 (69).

Same as worker except for standard queen modifications and as follows: mesoscutum mostly longitudinally rugose, but with a central longitudinal carina that is thick at anterior margin; most of katepisternum and part of anepisternum smooth and shiny; gastral pilosity denser, and more distinctly bilayered.

#### Male.

Unknown.

#### Biology.

*Stenamma catracho* is known only from sifted leaf litter collected from the forest floor. It occurs from approximately 1100–1900 m elevation in montane wet forest habitats.

#### Comments.

*Stenamma catracho* is most likely to be confused with *Stenamma cusuco* or *Stenamma hojarasca*, both of which have expanded frontal lobes and somewhat elongate petioles. *Stenamma cusuco* can be distinguished easily by its distinctive tridentate clypeal margin, and *Stenamma hojarasca* can be separated by its longer petiole and completely shiny petiolar and postpetiolar nodes.

There are some morphological differences unique to the Meambar population (Figure 62) of *Stenamma catracho*. Meambar specimens have most pilosity longer, with the gastral pilosity clearly bilayered; they have lighter integument color; and they have narrower frontal lobes. The other populations have the pilosity shorter overall and the gastral pilosity denser and not as clearly bilayered. I treat these differences as intraspecific variation until more material is collected.

Molecular phylogenetic data show that *Stenamma catracho* most likely belongs to a clade that includes *Stenamma crypticum*, *Stenamma monstrosum*, and *Stenamma saenzae*, with *Stenamma monstrosum* being its sister species (Branstetter unpublished data).

#### Material examined.

**GUATEMALA:** ***Zacapa***: 2km SE La Unión, 14.95344°N, 89.27587°W, 1430m, 12 May 2009 (LLAMA); **HONDURAS: *Comayagua***: PN Cerro Azul Meambar, 14.86960°N, 87.89843°W, 1140m, 20 May 2010 (LLAMA); ***Olancho***: PN La Muralla, 15.09511°N, 86.73925°W, 1420m, 2 May 2010 (LLAMA); PN La Muralla, 15.09965°N, 86.74072°W, 1530m, 2 May 2010 (LLAMA); PN La Muralla, 15.10257°N, 86.73642°W, 1650m, 2 May 2010 (R. S. Anderson); PN La Muralla, 15.09807°N, 86.72047°W, 1860m, 3 May 2010 (LLAMA).

### Stenamma connectum sp. n.

urn:lsid:zoobank.org:act:E7037873-2998-4A7F-A239-250CBEA55373

http://species-id.net/wiki/Stenamma_connectum

**Figure 64.**
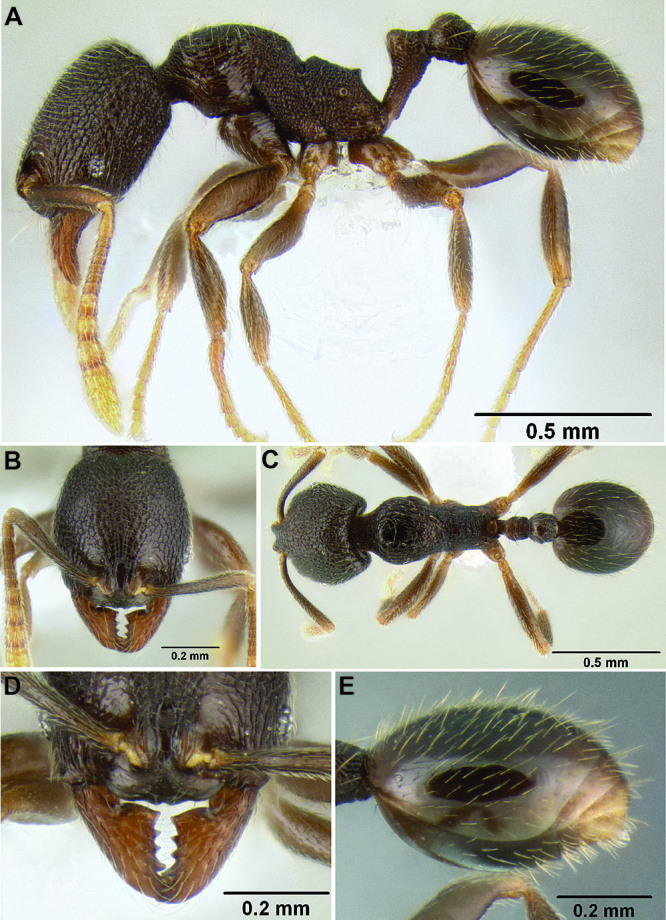
*Stenamma connectum* holotype worker (CASENT0622438) **A** Profile **B** Face **C** Dorsum **D **Anterior clypeal margin in anterodorsal view **E** Gaster.

**Figure 65.**
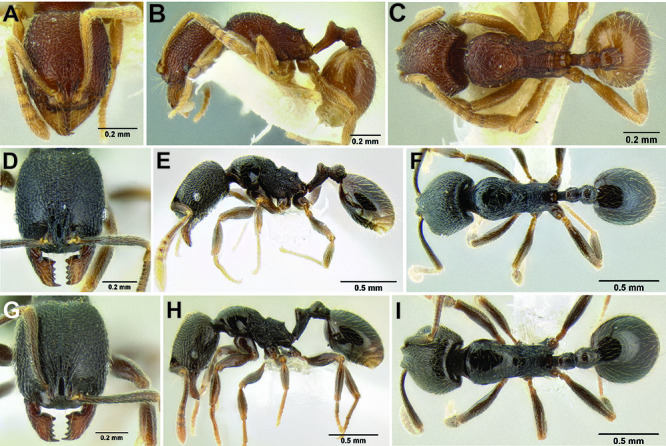
*Stenamma connectum* worker variants. Face, profile, and dorsal views **A–C** Variant 1 (CASENT012648) **D–F** Variant 2 (CASENT06005461) **G–I** Variant 3 (CASENT0605552).

**Figure 66.**
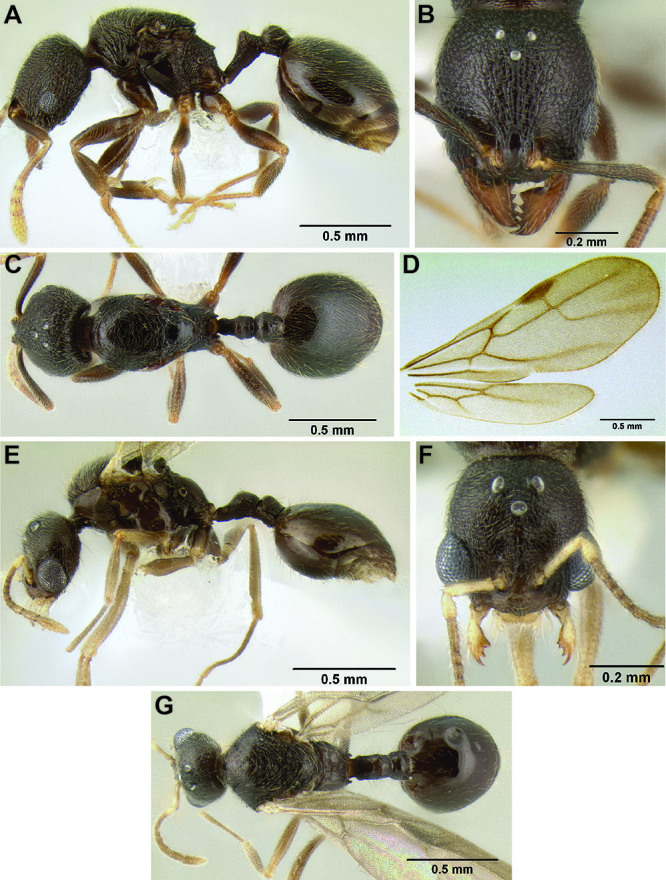
*Stenamma connectum* **A** Paratype queen (CASENT0622439), profile **B** Same, face **C** Same, dorsum **D** Queen (CASENT0126417), wings **E** Male (CASENT0605585), profile **F** Same, face **G** Same, dorsum.

**Figure 67.**
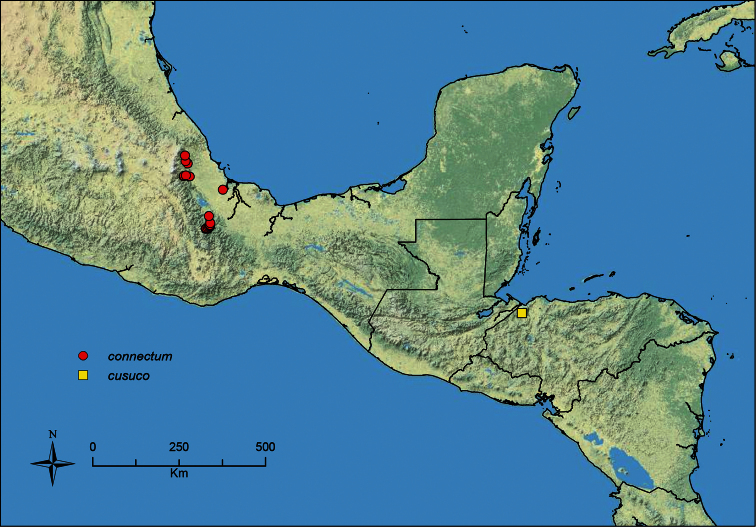
Distribution map of *Stenamma connectum* (circles) and *Stenamma cusuco* (squares).

Worker: Figures 64 , 65 ; Queen: Figure 66 ; Male: Figure 66E–G ; Map: Figure 67

#### Type material.

*Holotype worker.* MÉXICO, Oaxaca: 10.8km SW Valle Nacional, 17.68102°N, 96.33026°W ±66m, 1120m, 13 Aug 2009, disturbed mesophyll forest, ex sifted leaf litter (M. G. Branstetter, collection MGB1387) [USNM, specimen CASENT0622438]. *Paratypes*: same data as holotype [1dq, 1w, CAS, CASENT0623275, CASENT0623261], [1w, EAPZ, CASENT0623262], [1w, ECOSCE, CASENT0623263], [1w, FMNH, CASENT0623264], [1w, ICN, CASENT0623265], [1w, INBio, CASENT0623266], [1w, JTLC, CASENT0623274], [1w, LACM, CASENT0623267], [1w, MGBPC, CASENT0623268], [1dq, 1w, MCZ, CASENT0623276, CASENT0623272], [1w, MZSP, CASENT0623269], [1w, UCD, CASENT0623270], [1w, UNAM, CASENT0623271], [1dq, 1w, USNM, CASENT0622439, CASENT0623260], [1w, UVGC, CASENT0623273].

#### Worker diagnosis.

Integument mostly dark brown to brown, rarely black; small-sized species (see HL, ML, PrW below); basal margin of mandible sinuous, with a distinct basal depression; anterior clypeal margin undulating, forming 2–4 blunt teeth; face completely sculptured, mostly rugoreticulate; mesosoma mostly sculptured, except for pronotum, which is usually lightly carinulate-punctate, with a small smooth patch on dorsum and side, but sometimes pronotum completely punctate or mostly smooth; remainder of mesosoma usually strongly punctate; propodeal declivity in profile usually with distinctive outline, in which propodeal lobe is broadly rounded and makes a smooth, sinuous connection with propodeal spine (degree of sinuosity variable); eye of small to moderate size (EL 0.07–0.11, REL 15–19), oval-shaped, with 4–5 ommatidia at greatest diameter; pilosity on gastral dorsum bilayered, with a layer of longer suberect setae, and a denser layer of decumbent setae; propodeal spines tuberculate, or reduced to sharp angles (PSL 0.07–0.09, PSI 1.1–1.4); geography useful in species determination. *Similar species*: *Stenamma crypticum*, *Stenamma huachucanum*.

#### Geographic range.

 Southern Mexico (Oaxaca, Veracruz).

#### Worker description.

(20 measured) HL 0.52–0.69 (0.54), HW 0.45–0.60 (0.48), FLD 0.11–0.16 (0.12), PCW 0.02–0.04 (0.02), SL 0.39–0.58 (0.42), EL 0.07–0.11 (0.08), ACL 0.40–0.54 (0.43), ML 0.61–0.87 (0.65), PrW 0.31–0.40 (0.32), PSL 0.07–0.09 (0.08), SDL 0.05–0.07 (0.06), PL 0.22–0.32 (0.23), PH 0.14–0.19 (0.14), PW 0.10–0.16 (0.11), PPL 0.12–0.20 (0.14), PPH 0.12–0.19 (0.13), PPW 0.13–0.19 (0.15), MFL 0.38–0.62 (0.44), MTL 0.31–0.52 (0.35), CI 84–92 (89), SI 85–99 (87), REL 15–19 (16), FLI 24–28 (25), PSI 1.1–1.4 (1.4), MFI 96–117 (109), ACI1 67–72 (70), ACI2 93–105 (102).

Small-sized species; general body color usually dark brown to brown, rarely almost black; appendages brown to yellow-brown, becoming lighter toward extremities; in specimens with dark brown to black body color, flagellum sometimes noticeably bright yellow; setae golden brown; mandible with 6 teeth, with basal tooth well defined; basal margin of mandible sinuous, with a distinct basal depression; mandible surface mostly smooth and shiny, with scattered piligerous punctae, and some striations on base and lateral surface; anterior clypeal margin undulating, forming 2–4 blunt teeth, inner teeth often projecting beyond lateral teeth (type population); median lobe of clypeus with a pair of longitudinal carinulae that diverge anteriorly, apex of lobe with a moderately long transverse carinula, area in between median lobe and anterior margin forming a distinct cavity where mandibles insert, remainder of clypeus mostly smooth and shiny; posterior extension of clypeus between antennal insertions of moderate to somewhat narrow width (PCW 0.02–0.04), with sides subparallel to slightly hour-glass shaped; frontal lobes of moderate width (FLD 0.11–0.16, FLI 24–28), not greatly obscuring torular lobes in full-face view; head roughly oval-shaped (CI 84–92), posterior margin with a slight to distinct median depression; eye of small to moderate size (EL 0.07–0.11, REL 15–19), oval-shaped, with 4–5 ommatidia at greatest diameter; face completely sculptured, mostly rugoreticulate, with some longitudinal carinulae extending back from frontal lobes, interstitial areas with light piligerous punctae; one high-elevation population with facial sculpture more polished, becoming smooth toward posterior margin; scape somewhat short (SI 85–99), not reaching posterior margin when laid back; scape surface mostly smooth, with scattered piligerous punctae; flagellum with a distinct 4-segmented antennal club; mesosoma mostly sculptured, except for pronotum, which ranges from completely punctate to almost completely smooth (both extremes rare); pronotum most often lightly punctate on side, and lightly carinulate-punctate on dorsum, with a small smooth patch in middle (type population); remainder of mesosoma mostly punctate, with some longitudinal carinulae, becoming rugoreticulae on mesonotum; propodeal dorsum and declivity sometimes with transverse carinulae; one high-elevation population with mesopleuron rugulose-punctate, and side of propodeum longitudinally carinulate; promesonotum in profile low-domed and roughly symmetrical; metanotal groove well demarcated, of moderate width and depth; propodeum in profile usually with a distinct profile, in which propodeal lobe has a broadly rounded margin that connects smoothly with propodeal spine; connection usually sinuous, but degree of sinuosity variable; propodeal spines usually tuberculate (type population), but sometimes reduced to sharp angles (PSL 0.07–0.09, PSI 1.1–1.4); petiole in profile of moderate length to slightly elongate (PL/HW 0.49–0.55); petiolar node in profile appearing small (PH/PL 0.52–0.64), and asymmetrical, with anterior face longer and more sloping than posterior face, node dorsum broadly rounded (type population) to more angulate, with apex pointing distinctly posteriad; postpetiole in profile subspherical, usually about same size as petiolar node, but sometimes slighly bulging (PPH/PH 0.79–1.01); petiole and postpetiole usually mostly punctate, with only anterior faces smooth and shiny, but sometimes nodes almost completely smooth; most of body dorsum with short standing pilosity; setae on face very short, mostly subdecumbent; pilosity on gastral dorsum bilayered, with a sparse layer of longer suberect setae, and a denser layer of decumbent setae, sometimes lower layer very similar in density to upper layer; setae on scape subdecumbent to appressed; setae on legs mostly decumbent to appressed, with some suberect setae on femoral venters and coxae.

#### Queen description.

(8 meausred) HL 0.55–0.68 (0.56), HW 0.50–0.62 (0.51), FLD 0.13–0.15 (0.13), PCW 0.03–0.05 (0.03), SL 0.42–0.56 (0.45), EL 0.14–0.17 (0.15), ACL 0.43–0.52 (0.45), ML 0.74–1.00 (0.78), PrW 0.44–0.55 (0.46), PSL 0.09–0.11 (0.11), SDL 0.07–0.09 (0.09), PL 0.28–0.36 (0.29), PH 0.16–0.21 (0.16), PW 0.12–0.17 (0.13), PPL 0.14–0.21 (0.15), PPH 0.16–0.21 (0.16), PPW 0.17–0.22 (0.17), MFL 0.44–0.62 (0.46), MTL 0.38–0.53 (0.39), CI 88–93 (91), SI 83–95 (88), REL 27–30 (29), FLI 25–26 (26), PSI 1.2–1.5 (1.3), MFI 96–115 (111), ACI1 66–69 (68), ACI2 93–106 (102).

Same as worker except for standard queen modifications and as follows (comparsion of type population only): pronotum with transverse carinulae/rugulae; mesoscutum mostly smooth, with piligerous punctae, and some longidutinal carinulae along lateral margin; scutellum smooth in middle, rugulose-punctate on lateral margins; mesopleuron mostly smooth; propodeum carinulate-punctate; lower layer of setae on gastral dorsum very dense, almost pubescent; dorsum of mesosoma and anterior faces of petiolar and postpetiolar nodes with a dense layer of short, decumbent setae; propodeal declivity in profile less sinuous, with propodeal lobe smaller and less evenly rounded; wing venation as in Figure 66D.

#### Male.

See Figure 66E–G.

#### Biology.

*Stenamma connectum* is found in montane wet forest habitats from 600–2160 m elevation, and is known almost exclusively from Winkler or Berlese samples of sifted leaf litter. Only once has a nest been found: a single dealate queen with brood underneath a moss mat.

#### Comments.

Distinguishing *Stenamma connectum* from *Stenamma crypticum* and *Stenamma huachucanum * can be difficult. This is because each species is composed of multiple allopatric populations with no clear evidence of sympatry among divergent forms. Using morphology alone, the best solution would probably be to delimit a single widespread species. However, molecular phylogenetic data strongly suggest the existence of multiple biological species (Branstetter unpublished data). As a result, I have decided to delimit several species guided by the combination of morphological and molecular data. Some phenotypically divergent populations which have not been included in the phylogeny may prove challenging to identify.

As currently defined, geography is very useful in separating *Stenamma connectum* from similar species. *Stenamma connectum* occurs in the Mexican states of Veracruz and Oaxaca only. Within Oaxaca, it is found along the Caribbean slope in wet forest habitats. *Stenamma huachucanum* is distributed from the southwestern U.S.A to Oaxaca. Within Oaxaca, it occurs only in the drier habitats in the central and western parts of the state. I consider specimens from Hidalgo and San Luis Potosí states to be *Stenamma huachucanum*, but some of these specimens are hard to place. *Stenamma crypticum* occurs mainly from Chiapas, Mexico to Nicaragua; however, a couple of specimens known from one sample collected in Veracruz at 1600 m (11km N San Andrés Tuxtla) appear most like *Stenamma crypticum*. These specimens lack the broadly rounded propodeal lobes (in profile) and have the promesonotum mostly smooth. Until more material is collected, I treat these as *Stenamma crypticum*, but I find it possible that they are actually abberant specimens of *Stenamma connectum. * One specimen that clearly has the characteristics of *Stenamma connectum* was collected at nearly the same locality, only slightly lower at 1400 m elevation.

Many populations of *Stenamma connectum*, including the type population, share a set of distinctive morphological character states that distinguish *Stenamma connectum* from similar species. Key worker characters for the type population are as follows: body color mostly brown; side of pronotum lightly punctate; dorsum of pronotum carinulate-punctate with a small patch of smooth cuticle in middle; mesopleuron and side of propodeum strongly punctate; propodeal declivity in profile forming a sinuous outline, in which the propodeal lobe is broadly rounded and makes a smooth connection with the propodeal spine; propodeal spines tuberculate. The sinuous outline of the propodeal declivity is the most distinctive feature of this species. It is variable among populations, with specimens from lower elevations having the sinuosity more pronounced. Higher elevation populations begin to lose the character state, making these populations more difficult to separate from similar species. Several variants are described below.

Variant 1 (Figure 65A–C) is known only from the locality Pueblo Nuevo in Veracruz. These specimens differ as follows: smaller overall size; mesosoma completely punctate, with only a few scattered rugulae/carinulae; sinuosity in outline of propodeal declivity pronounced; petiolar node in profile reaching a more angular apex.

Variant 2 (Figure 65D–F) and variant 3 (Figure 65G-I) occur along the same elevational transect as the type population, but at mid (~1770 m) and high (> 1900 m) elevations, respectively. Variant 2 differs from the type form in that it has dark brown body color, reduced pronotal sculpture, and a noticeably yellow flagellum. Variant 3 is a high elevation form of *Stenamma connectum*. Without the intermediate phenotype present in variant 2, it would be easy to describe variant 3 as a new species as it is very divergent from the type population. It is similar to variant 2 except as follows: larger size; body color almost black; pronotum almost completely smooth and shiny; mesopleuron rugulose-punctate; side of propodeum carinulate; outline of propodeal declivity not strongly sinuous, with the propodeal lobe often more angular and isolated from the propodeal spine; petiolar and postpetiolar nodes mostly smooth; postpetiole bulging.

#### Material examined.

** MÉXICO: *Oaxaca***: Mirador Grande, 17.89844°N, 96.36263°W, 990m, 14 Aug 2009 (M. G. Branstetter); 10km S Valle Nacional, [ca. 17.724°N, 96.324°W], 650m, 19 May 1971 (S. B. Peck); 10.8 km SW Valle Nacional, 17.68102°N, 96.33026°W, 1120m, 13 Aug 2009 (M. G. Branstetter); 13.2km SW Valle Nacional, 17.65934°N, 96.33426°W, 1360m, 11 Aug 2009 (M. G. Branstetter); 14.8km SSW Valle Nacional, 17.64483°N, 96.33637°W, 1370m, 13 Aug 2009 (M. G. Branstetter); 20.5km SW Valle Nacional, 17.60560°N, 96.38398°W, 1770m, 12 Aug 2009 (M. G. Branstetter); 20.6km SW Valle Nacional, 17.60404°N, 96.378786°W, 1733m, 13 Aug 2009 (M. G. Branstetter); 22.4km SW Valle Nacional, 17.59112°N, 96.39133°W, 1990m, 13 Aug 2009 (M. G. Branstetter); 25km S Valle Nacional, [ca. 17.670°N, 96.330°W], 1200m, 21 May 1971 (S. B. Peck); 26km SW Valle Nacional, 17.58667°N, 96.44948°W, 2160m, 11 Aug 2009 (M. G. Branstetter); 30.1km S Valle Nacional, [ca. 17.627°N, 96.354°W], 1580m, 11–18 Aug 1973 (A. F. Newton); 47.5km SW Valle Nacional, Km 100.5, [ca. 17.595°N, 96.424°W], 2125m, 26 Jul 1992 (R. S. Anderson); 52km S Valle Nacional, [ca. 17.587°N, 96.449°W], 2130m, 22 May 1971 (S. B. Peck); ***Veracruz***: Cordoba, [ca. 18.908°N, 96.958°W], 4 Aug 1969 (S. B. Peck); Cordoba, Paraje Nuevo, Nacimiento, [ca. 18.88°N, 96.86°W], 7 Aug 1969 (S. & J. Peck); park cañon, Hwy150, 3.2km W Fortín, [ca. 18.884°N, 97.019°W, 6–9 Aug 1969 (S. & J. Peck); Fortín, Canyon Río Metlac, [ca. 18.90°N, 97.00°W], 5 Aug 1969 (S. & J. Peck); 7km E Huatusco, [ca. 19.207°N, 96.914°W], 22 Jun 1983 (Peck & Anderson); 7.1km N Huatusco, [ca. 19.262°N, 96.964°W], 1280m, 29 Jul–3 Aug 1973 (A. F. Newton); Paraje Nuevo, Cueva de Ojo de Agua, [ca. 18.877°N, 96.862°W], 7 Aug 1969 (S. & J. Peck) Pueblo Nuevo, nr Tetzonapa, 1 Aug 1953 (E. O. Wilson); 11km N San Andrés Tuxtla, 18.55°N, 96.00°W, 1400m, 23 Mar 1985 (P. S. Ward); 2.7km N Teocelo, [ca. 19.40°N, 96.98°W], 1130m, 22–24 Jul 1934 (A. F. Newton).

### Stenamma crypticum sp. n.

urn:lsid:zoobank.org:act:94BE0671-5381-4F80-B10C-7055DF3BB8D6

http://species-id.net/wiki/Stenamma_crypticum

**Figure 68.**
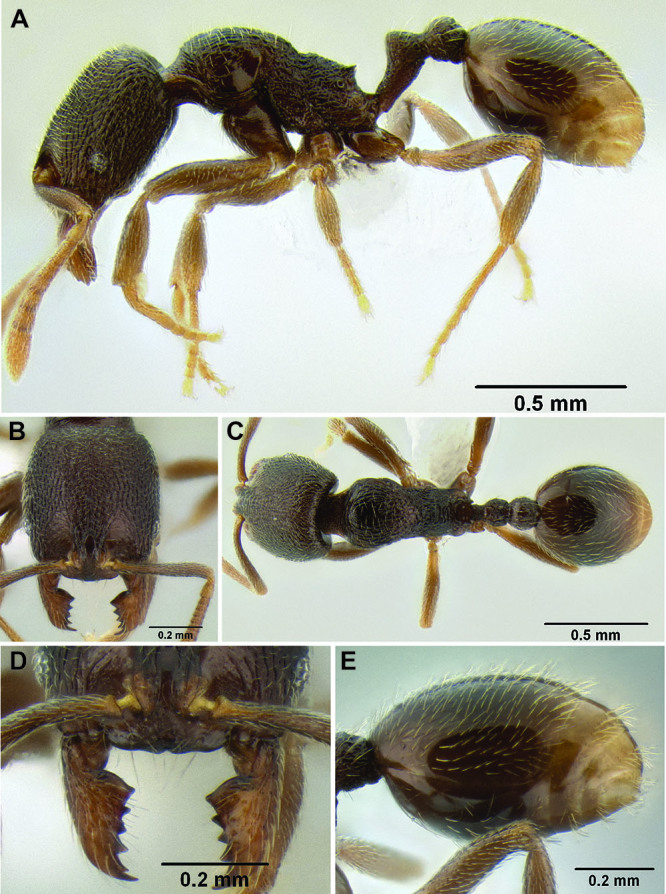
*Stenamma crypticum* holotype worker (CASENT0605185) **A** Profile **B** Face **C** Dorsum **D **Anterior clypeal margin in anterodorsal view **E** Gaster.

**Figure 69.**
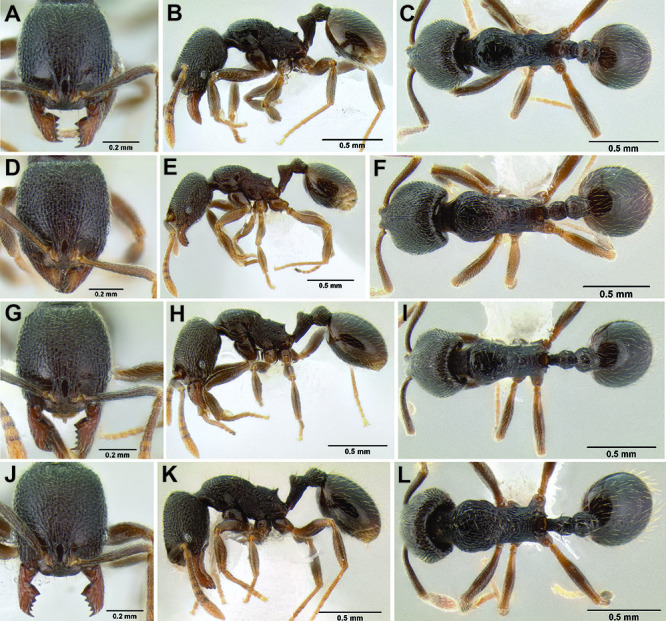
*Stenamma crypticum* worker variants. Face, profile, and dorsal views **A–C** Type population worker with less sculpture (CASENT0603821) **D–F** Type population worker with more sculpture (CASENT0604745) **G–I** Variant 1 (CASENT0604839) **J–L** Variant 2 (CASENT0604997).

**Figure 70.**
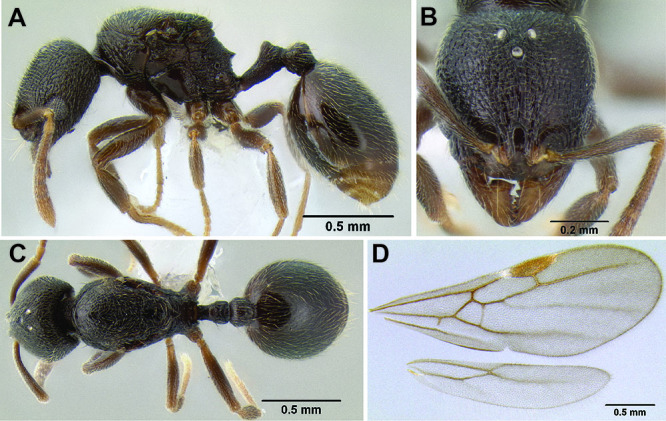
*Stenamma crypticum* **A** Queen (CASENT0604854), profile **B** Same, face **C** Same, dorsum **D** Queen (CASENT0605933), wings.

**Figure 71.**
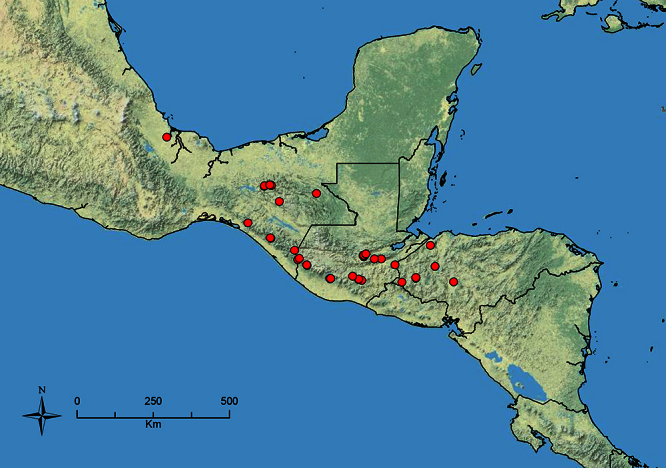
Distribution map of *Stenamma crypticum*.

Worker: Figures 68 , 69 ; Queen: Figure 70A–D ; Map: Figure 71

Stenamma mgb12 Branstetter, 2012: phylogeny.

#### Type material.

*Holotype worker.* MÉXICO, Chiapas: 2km SE Custepec, 15.72141°N, 92.93936°W, 1860m, 17 May 2008, oak-pine forest, ex sifted leaf litter (R. S. Anderson, collection RSA2008-013) [USNM, specimen CASENT0604771] *Paratypes*: MÉXICO, Chiapas: 3km SE Custepec, 15.71485°N, 92.93823°W ±50m, 1700m, 17 May 2008 (LLAMA, Wa-A-02-2-50) [1dq, 1w, CAS, CASENT0623277, CASENT0623280], [1w, EAPZ, CASENT0623281], [1w, ECOSCE, CASENT0623282], [1w, FMNH, CASENT0623283], [1w, ICN, CASENT0623284], [1w, INBio, CASENT0623286], [1w, JTLC, CASENT0623536], [1w, LACM, CASENT0623288], [1w, MGBPC, CASENT0623537], [1dq, 1w, MCZ, CASENT0623278, CASENT0623289], [1w, MZSP, CASENT0623290], [1w, UCD, CASENT0623291], [1w, UNAM, CASENT0623292], [1dq, 1w, USNM, CASENT0623279, CASENT0623293], [1w, UVGC, CASENT0623294].

#### Worker diagnosis.

Integument dark red-brown to brown; small-sized species (see HL, ML, PrW below); basal margin of mandible sinuous, usually with a small basal depression; anterior clypeal margin undulating, with two small blunt teeth bordering midline; eye of moderate size (EL 0.07–0.11, REL 15–20), oval-shaped, with 4–5 ommatidia at greatest diameter; face mostly rugoreticulate; mesosoma often mostly sculptured, but pronotum variable, usually rugose with smooth patches on dorsum and side, but sometimes mostly rugose or mostly smooth; propodeal spines tuberculate to short (PSL 0.07–0.10, PSI 1.2–1.8); gastral pilosity usually short, dense and clearly bilayered, with a layer of suberect setae and a denser underlying layer of subdecumbent setae, but sometimes setae more uniformly subdecumbent, or suberect setae thickened; geography useful in species determination. *Similar species*: *Stenamma connectum*, *Stenamma huachucanum*, *Stenamma ignotum*, *Stenamma picopicucha*.

#### Geographic range.

Southern Mexico (Chiapas, Veracruz?) to Honduras.

#### Worker description.

(21 measured) HL 0.54–0.65 (0.59), HW 0.45–0.57 (0.51), FLD 0.12–0.16 (0.14), PCW 0.02–0.04 (0.03), SL 0.39–0.51 (0.42), EL 0.07–0.11 (0.08), ACL 0.39–0.48 (0.42), ML 0.62–0.79 (0.70), PrW 0.31–0.40 (0.35), PSL 0.07–0.10 (0.09), SDL 0.05–0.06 (0.05), PL 0.22–0.29 (0.26), PH 0.13–0.18 (0.15), PW 0.11–0.16 (0.14), PPL 0.13–0.18 (0.16), PPH 0.12–0.18 (0.15), PPW 0.13–0.21 (0.16), MFL 0.40–0.54 (0.45), MTL 0.34–0.45 (0.37), CI 84–91 (87), SI 80–89 (81), REL 15–20 (16), FLI 25–29 (26), PSI 1.2–1.8 (1.6), MFI 103–117 (115), ACI1 69–72 (69), ACI2 93–103 (100).

Small-sized species; general body color dark brown to brown or orange-brown, with appendages brown or orange-brown to yellow-brown, becoming lighter toward extremities; setae golden brown; mandible with 6 teeth, inner teeth sometimes worn; basal margin of mandible sinuous, usually with a distinct basal depression, but without tooth; mandible mostly smooth and shining, with scattered piligerous punctae and some basal striae; anterior clypeal margin when viewed from anterodorsal angle undulating, usually forming 2 blunt teeth bordering midline; median lobe of clypeus with a pair of faint longitudinal carinulae that diverge anteriorly, apex of lobe with short transverse carinula; area in between median lobe and anterior clypeal margin forming a shallow concavity where mandibles insert; remaining surface of clypeus mostly smooth; posterior extension of clypeus between antennal insertions somewhat narrow (PCW 0.02–0.04), sides subparallel; frontal lobes of moderate width (FLD 0.12–0.16, FLI 25–29), not greatly obscuring torular lobes in full-face view; head subrectangular to oval-shaped (CI 84–91), posterior margin slightly depressed medially; eye somewhat small (EL 0.07–0.11, REL 15–20), oval-shaped, with 4–5 ommatidia at greatest diameter; head completely sculptured, mostly rugoreticulate, with a few longitudinal carinulae along midline; scape relatively short (SI 80–89), not reaching posterior margin of head when laid back; scape surface mostly smooth, with scattered piligerous punctae; flagellum with distinct 4-segmented antennal club, last segment noticeably bulging; pronotal sculpture variable, dorsum usually longitudinally rugose, with a small smooth patch mesad (type population), side usually rugose on upper half and smooth on lower half (type population), sometimes pronotum completely smooth, or completely rugose, with only a small smooth patch on side; dorsum of mesonotum rugulose punctate; katepisternum and side of propodeum punctate, sometimes with a few rugulae; propodeal dorsum punctate, with a few transverse carinulae; propodeal declivity mostly smooth, with a few transverse carinulae on upper half; promesonotum in profile low-domed, and roughly symmetrical; propodeal spines tuberculate to short (PSL 0.07–0.10, PSI 1.2–1.8); petiole usually somewhat short and stocky (PL/HW 0.47–0.54); petiolar node somewhat small (PH/PL 0.56–0.57), roughly symmetrical, dorsum reaching a defined apex, which points nearly vertical; postpetiole in profile variable, usually small and similar in size to petiolar node (type population), but sometimes bulging (PPH/PH 0.91–1.07); petiole and postpetiole usually mostly punctate, with anterior faces of nodes smooth, and posterior faces of nodes with a few rugulae; most of body dorsum with short standing pilosity; pilosity on gastral dorsum usually distinctly bilayered, with a layer of suberect setae, and a slightly more dense layer of decumbent setae (type population), but sometimes setae more uniformly suberect to subdecumbent and less clearly bilayered; suberect layer of setae sometimes slightly thickened; setae on scapes decumbent to appressed; setae on legs mostly subdecumbent to appressed, with longer suberect setae on femoral venters and coxae.

#### Queen description.

(7 measured) HL 0.59–0.67 (0.59), HW 0.52–0.61 (0.52), FLD 0.15–0.17 (0.15), PCW 0.04–0.05 (0.04), SL 0.42–0.50 (0.44), EL 0.14–0.17 (0.14), ACL 0.42–0.48 (0.44), ML 0.80–0.95 (0.80), PrW 0.46–0.55 (0.46), PSL 0.10–0.13 (0.11), SDL 0.07–0.08 (0.07), PL 0.28–0.34 (0.30), PH 0.17–0.20 (0.17), PW 0.15–0.17 (0.15), PPL 0.16–0.20 (0.18), PPH 0.17–0.22 (0.17), PPW 0.19–0.24 (0.19), MFL 0.47–0.57 (0.47), MTL 0.39–0.48 (0.41), CI 88–92 (88), SI 79–86 (84) REL 26–28 (28), FLI 26–30 (28), PSI 1.3–1.7 (1.6), MFI 102–113 (110), ACI1 68–70 (68), ACI2 90–100 (100).

Same as worker except for standard queen modifications and as follows: pronotum with transverse carinulae on humeri, becoming smooth mesad; mesoscutum mostly with longitudinal rugulae and foveolae, midline and mesolateral margin smooth; scutellum smooth along midline, and longitudinally carinulate laterad; propodeum with transverse carinulae/rugulae that wrap around surface; mesopleuron mostly smooth; lower layer of setae on gastral dorsum very dense, almost pubescent; wing venation as in Figure 70D.

#### Male.

Unknown.

#### Biology.

*Stenamma crypticum* is a rather common component of the leaf litter in mid- to high-elevation mesic forest habitats in Central America. It has been collected from 900–2800 m, but is most common from 1500–2500 m. Habitat types include cloud forest, mesophyll forest, oak forest, and mixed hardwood forest. Most collections come from samples of sifted leaf litter collected from the forest floor, but some specimens are also known from cookie baits. Nests have never been found, but dealate queens, as well as workers, are common in the leaf litter, suggesting that nests might be in this stratum.

#### Comments.

*Stenamma crypticum* should be distinguished easily from *Stenamma ignotum* and *Stenamma picopicucha* using the diagnostic characters given above. As noted under *Stenamma connectum* above, separating *Stenamma crypticum* from *Stenamma connectum* and *Stenamma huachucanum* is more challenging. This is because each species comprises a complex of multiple divergent populations, with no clear evidence of sympatry among distinct forms. Using morphology alone, it might be best to name a single species; however, molecular phylogenetic data strongly suggest the existence of at least three species (Branstetter unpublished data). Even though some populations/specimens may prove difficult to identify, I have chosen to delimit these species as best as possible. There are a few key morphological characters that separate the type populations of each species from one another, but when considering all populations, geography is useful.

*Stenamma crypticum* occurs mainly in Nuclear Central America from Chiapas, Mexico to Honduras, whereas *Stenamma connectum* is found north of *Stenamma crypticum* in Veracruz, Mexico and on the wet, Caribbean slope of Oaxaca. Complicating this picture, however, are a few specimens collected from Veracruz at 1600 m (11km N San Andrés Tuxtla). These specimens lack the broadly rounded propodeal lobes characteristic of *Stenamma connectum* and have a mostly smooth promesonotum. I tentatively identify these specimens as *Stenamma crypticum*, but I find it possible that they could actually be abberant members of *Stenamma connectum*, which has been collected close by at a slightly lower elevation (1400 m). The phylogenetic position of the putative *Stenamma crypticum* specimens has not been assessed. *Stenamma* *huachucanum* is distributed from the southwestern U.S.A. to Oaxaca, where it occurs only on the drier western side of the state. A wet forest version of *Stenamma huachucanum* occurs in eastern Mexico from Tamaulipas to Puebla.

*Stenamma picopichuca* can be separated from *Stenamma crypticum* using the diagnostic characters listed above, but I am somewhat uncertain about the phylogenetic placement and status of this species. *Stenamma picopichucha* has the basal margin of the mandible like *Stenamma ignotum* and the anterior clypeal margin like *Stenamma crypticum*. Because of several other similarities, I was originally going to include *Stenamma picopicucha* in *Stenamma crypticum*, but I later discovered that the two species occur in sympatry at Cusuco in Honduras. At this site, specimens of *Stenamma crypticum* clearly have a sinuous basal margin of the mandible and specimens of *Stenamma picopicucha* have a straight margin. The phylogenetic position of *Stenamma picopicucha* is yet to be tested, and it will be interesting to see if the two species are closely related.

Within the range of *Stenamma crypticum* there is considerable variation in size, sculpture and gastral pilosity, with some of this variation observable at single sites. At the type locality, for example, I have sampled *Stenamma crypticum* from 1500 m to about 2200 m elevation. Along this gradient specimens from higher elevation are larger. There is also significant variation in pronotal sculpture within the site, with some specimens having the pronotal dorsum mostly smooth (Figure 69A–C), and others mostly rugose (Figure 69D–F). At first I tried separating these into distinct forms, but I abandoned this scheme after finding specimens with intermediate phenotypes.

In addition to within site variation, there is considerable among population variation, with almost every population displaying some unique feature. Out of this diversity I describe a couple of variants. Variant 1 (Figure 69G–I) occurs at several sites in Guatemala (e.g. Biotopo Quetzal, Purulhá). It is similar to the type form, but has a bulging postpetiole and longer gastral pilosity. Variant 2 (Figure 69J–L) occurs at La Union in Guatemala, with similar-looking specimens at sites in Honduras. Compared to the type form it is smaller and has the suberect layer of gastral pilosity noticeably thickened. Until there is evidence of sympatry, I treat all of this variation as intraspecific.

#### Material examined.

** GUATEMALA:** ***Baja Verapaz***: Biotopo Quetzal, 15.21298°N, 90.21510°W, 1750m, 7 May 2009 (LLAMA); Purulhá, Biotopin, 15.21535°N, 90.21618°W, 1700m, 26–30 Mar 2008 (Méndez et al.); 7km E Purulhá, [ca, 15.2667°N, 90.1348°W], 1600m, 25 May 1991 (R. S. Anderson); 4.5km S Purulhá, [ca. 15.226°N, 90.200°W], 1630m, 21 May 1991 (R. S. Anderson); 7km S Purulhá, [ca. 15.194°N, 90.200°W], 1660m, 20 May 1991 (R. S. Anderson); 4km SSE Purulhá, 15.20522°N, 90.22198°W, 2100m, 20 Sep 2008 (M. G. Branstetter); Ranchito El Quetzal, 15.21443°N, 90.22123°W, 1750m, 20 Sep 2008 (R. S. Anderson); Salamá, Cerro Verde, 15.17446°N, 90.19353°W, 1800m, 26–30 Mar 2008 (Méndez et al.); Salamá, Hotel Posada del Quetzal 1, 15.19710°N, 90.21169°W, 1600m, 26–30 Mar 2008 (Méndez et al.) ***El Progresso***: 20km N Estancia de la Virgen, 15.1141°N, 89.8833°W, 1850m, 8 Jun 1991 (R. S. Anderson); ***Guatemala***: 1km SE La Pueblito, [ca. 14.6211°N, 90.5269°W], 1800m, 10 Jun 1991 (R. S. Anderson); nr Las Nubes, 14.53349°N, 90.35941°W, 1850m, 18 Sep 2008 (R. S. Anderson); 5.9km ESE San José Pinula, 14.53349°N, 90.35941°W, 1850m, 18 Sep 2008 (M. G. Branstetter); 7km ESE San José Pinula, 14.53915°N, 90.34933°W, 2060m, 18 Sep 2008 (M. G. Branstetter); ***Jalapa***: Aldea Manzano, 1.8km WNW San José La Sierra, 14.50476°N, 90.25613°W, 1990m, 18 Sep 2008 (M. G. Branstetter); El Manzano, 14.50476°N, 90.25613°W, 2150m, 18 Sep 2008 (R. S. Anderson); ***San Marcos***: La Fraternidad, 14.93604°N, 91.86778°W, 1920m, 11 Sep 2008 (M. G. Branstetter); Rd. Bojonal-Fraternidad, 14.94533°N, 91.88038°W, 1580m, 11 Sep 2008 (R. S. Anderson); 9.8km WSW San Marcos, 14.94427°N, 91.87990°W, 1600m, 11 Sep 2008 (M. G. Branstetter);***Suchitepéquez***: Finca Sn. Jerónimo, 14.55914°N, 91.16705°W, 1790m, 11 Dec 2010 (L. Sáenz); Volcán Atitlán, 9.5km SE Santiago Atitlán, 14.55828°N, 91.19133°W, 2000m, 10 Sep 2008 (M. G. Branstetter); 4km S Vol. Atitlán, 14.54830°N, 91.19115°W, 1625m, 15 Jun 2009 (LLAMA); 4km S Vol. Atitlán, 14.55112°N, 91.19848°W, 1750m, 15 Jun 2009 (LLAMA); ***Zacapa***: 14km NNE Teculután, 15.1134°N, 89.6781°W, 2270m, 6 Jul 2007 (R. S. Anderson); 2km SE La Unión, 14.94701°N, 89.27594°W, 1550m, 12 May 2009 (LLAMA);** HONDURAS: *Comayagua***: 10km E Comayagua, 14.45973°N, 87.54609°W, 2000m, 15 May 2010 (LLAMA); ***Cortés***: PN Cusuco, 15.50739°N, 88.23373°W, 2030m, 3 Jun 2010 (LLAMA);***Lempira***: PN Celaque, 8.3km SW Graçias, 14.56132°N, 88.65768°W, 1860m, 30 Sep 2008 (M. G. Branstetter); PN Celaque, 8.7km SW Graçias, 14.5877°N, 88.66117°W, 2100m, 30 Sep 2008 (M. G. Branstetter); ***Ocotepeque***: 13km E Nuevo Ocotopeque, 14.45685°N, 89.06856°W, 2200m, 25 May 2010 (LLAMA); ***Santa Barbara***: 15km SE Santa Barbara, [ca. 14.906°N, 88.100°W], 24 Aug 1991 (S. B. Peck);** MÉXICO: *Chiapas***: Cacahuatan, Las Nubes, [ca. 15.097°N, 92.140°W], 1770m, 1–7 Aug 1950 (Goodnights); Cerro Huitepec (Pico), 5km W San Cristobal, [ca. 16.7500°N, 92.6802°W], 2750m, 18 Sep 1991 (R. S. Anderson); Cerro de Tapalapa, 17.18786°N, 93.12308°W, 2260m, 28 May 2008 (R. S. Anderson); 5km NE Coapilla, 17.17557°N, 93.13187°W, 1990m, 25 May 2008 (LLAMA); 2km SE Custepec, 15.72099°N, 92.95050°W, 1520m, 17 May 2008 (LLAMA); 4km SE Custepec, 15.70658°N, 92.93126°W, 2125m, 20 May 2008 (LLAMA); Lagunas de Montebello, Cinco Lagos, [ca. 15.2667°N, 90.1348°W], 1660m, 23 May 1991 (R. S. Anderson); 7.4km SSW Motozintla de Mendoza, [ca. 15.367°N, 92.233°W], 2000m, 21 Sep 1992 (R. S. Anderson); Najá, 16.97417°N, 91.58592°W, 950m, 14 Jul 2007 (R. S. Anderson); 13km N Pueblo Nuevo Solistahuacán, [ca. 17.211°N, 92.964°W], 1860m, 26–27 Aug 1973 (A. F. Newton); 8.9km E Rayon, 17.20000°N, 92.91633°W, 1500m, 19 Sep 1991 (R. S. Anderson); Sierra Morena, C. Bola, 16.13464°N, 93.60077°N, 1950m, 14 May 2008 (R. S. Anderson); 17km ENE Tonalá, 16.14153°N, 93.60958°W, 1650m, 16 Jul 2007 (M. G. Branstetter); 4km N Union Juarez, Volcan Tacana, lower slopes, [ca. 15.133°N, 92.100°W], 2000m, 19 Sep 1992 (R. S. Anderson); ***Veracruz***: 11km N San Andrés Tuxtla, 18.55°N, 96.00°W, 1600m, 23 Mar 1985 (P. S. Ward).

### Stenamma cusuco sp. n.

urn:lsid:zoobank.org:act:C2EF644D-B275-46A5-A2A7-323451BC17B9

http://species-id.net/wiki/Stenamma_cusuco

**Figure 72.**
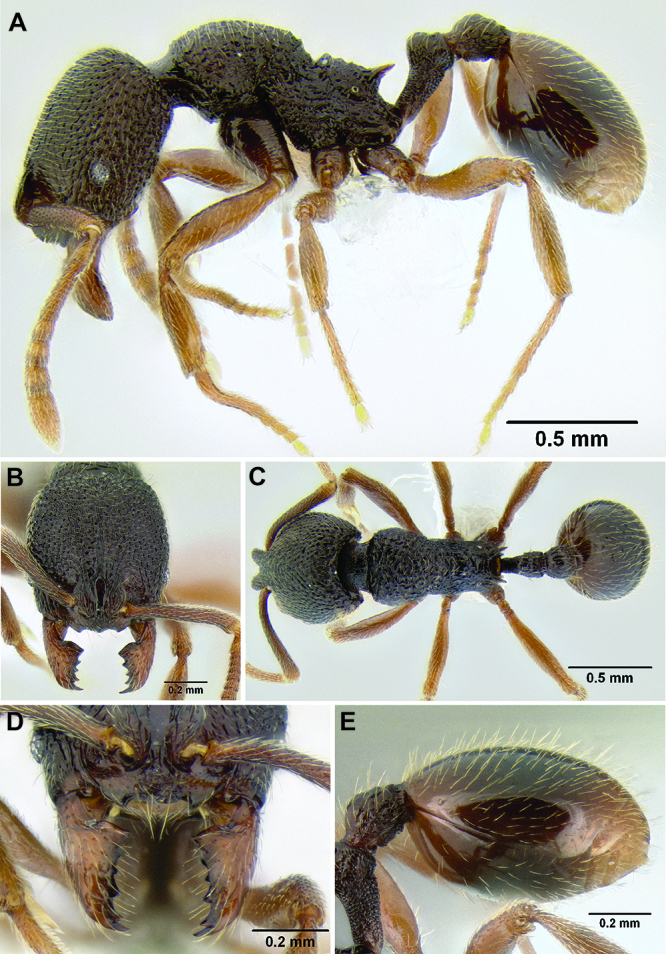
*Stenamma cusuco* holotype worker (CASENT0622137) **A** Profile **B** Face **C** Dorsum **D** Anterior clypeal margin in anterodorsal view **E** Gaster.

**Figure 73.**
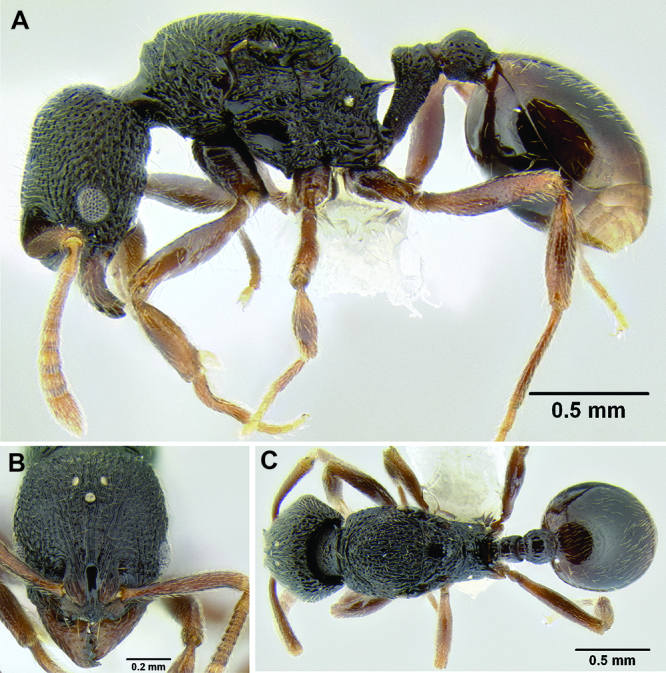
*Stenamma cusuco* paratype queen (CASENT0622136) **A** Profile **B** Face **C** Dorsum.

Worker: Figure 72 ; Queen: Figure 73 ; Map: Figure 67

Stenamma mgb66 Branstetter, 2012: phylogeny.

#### Type material.

*Holotype worker*. HONDURAS, Cortés: Parque Nacional Cusuco, 15.48965°N, 88.23383°W ±35m, 1300m, 31 May 2010, mesophyll forest, ex sifted leaf litter (LLAMA, collection Wm-C-06-1-04) [USNM, specimen CASENT0622137]. *Paratypes*: same data as holotype [1w, CAS, CASENT0623296], [1w, EAPZ, CASENT0623297], [1w, ECOSCE, CASENT0623298], [1w, FMNH, CASENT0623299], [1w, INBio, CASENT0623300], [1w, LACM, CASENT0623301], [1dq, 1w, MCZ, CASENT0623295, CASENT0623302], [1w, MZSP, CASENT0623303], [1dq, 1w, USNM, CASENT0622136, CASENT0622138]; same data but 15.48940°N, 88.23598°W ±20m, 1290m, 30 May 2010 (LLAMA, Wa-C-06-2-07) [1w, UCD, CASENT0621743], [1w, UNAM, CASENT0621741], [1w, UVGC, CASENT0621742]; 15.49037°N, 88.23402°W ±40m, 1330m, 31 May 2010 (LLAMA, Wm-C-06-1-07) [1w, JTLC, CASENT0623538], [1w, MGBPC, CASENT0623539].

#### Worker diagnosis.

Integument mostly red-black; small- to medium-sized species (see HL, ML, and PrW below); anterior clypeal margin in full-face view, with three projecting teeth, two well-defined sharp outer teeth, and a blunt central tooth, formed by a projecting median clypeal lobe; anterior clypeal margin underneath median clypeal lobe with two blunt teeth; basal margin of mandible sinuous, with a distinct basal depression, but no basal tooth; frontal lobes markedly expanded (FLD 0.19–0.22, FLI 24–26), completely obscuring the torular lobes in full-face view; eye of moderate size (EL 0.09–0.11, REL 14–17), with 5–6 ommatidia at greatest diameter; propodeal spines present and of moderate length (PSL 0.16–0.19, PSI 1.8–2.2); gastral setae short, and clearly bilayered, with a layer of suberect setae, and a layer of slightly shorter but denser subdecumbent setae;. *Similar species*: *Stenamma catracho,* *Stenamma hojarasca*, *Stenamma ochrocnemis*.

#### Geographic range.

Honduras.

#### Worker description.

(5 measured) HL 0.74–0.80 (0.78), HW 0.63–0.69 (0.66), FLD 0.24–0.26 (0.26), PCW 0.02–0.03 (0.03), SL 0.54–0.59 (0.59), EL 0.09–0.11 (0.10), ACL 0.54–0.57 (0.57), ML 0.88–0.97 (0.94), PrW 0.45–0.49 (0.48), PSL 0.16–0.19 (0.18), SDL 0.08–0.10 (0.09), PL 0.34–0.37 (0.37), PH 0.18–0.20 (0.20), PW 0.14–0.16 (0.16), PPL 0.18–0.20 (0.20), PPH 0.16–0.19 (0.19), PPW 0.19–0.20 (0.20), MFL 0.60–0.64 (0.63), MTL 0.50–0.52 (0.51), CI 83–87 (86), SI 84–89 (89), REL 14–17 (15), FLI 36–39 (39), PSI 1.8–2.2 (2.1), MFI 104–111 (106), ACI1 66–68 (67), ACI2 96–100 (96).

Small- to medium-sized species; general body color red black, with patches of brown on gaster; mandibles and appendages lighter, mostly brown to orange-brown; setae pale golden brown; mandible usually with 6 teeth, sometimes with 1–2 small denticles, basal tooth well-defined; basal margin of mandible sinuous, with a distinct basal depression, but no basal tooth; mandible mostly smooth and shiny, with scattered piligerous punctae and basal striae; anterior clypeal margin in full-face view, with three projecting teeth, two well-defined outer teeth, and a more blunt central tooth, formed from a projecting median clypeal lobe; anterior clypeal margin underneath median clypeal lobe with two blunt teeth (only visible with mandibles open and from an anterodorsal view); median clypeal lobe surface usually with a single median longitudinal carinula and a variable number of irregular foveolae, remainder of clypeus smooth and shiny; posterior extension of clypeus between antennal insertions hourglass-shaped, with middle of hourglass narrow (PCW 0.02–0.03); frontal lobes noticeably expanded (FLD 0.19–0.22, FLI 24–26), completely obscuring the torular lobes in full-face view; head roughly oval-shaped (CI 83–87), posterior margin with a slight median depression; eye of moderate size (EL 0.09–0.11, REL 14–17), oval-shaped, with 5–6 ommatidia at greatest diameter; head strongly rugoreticulate (not quite foveate), with a few short costae extending back from the frontal lobes and posterior clypeal extension; scape somewhat short (SI 84–89), not reaching posterior margin of head when laid back; scape surface shiny, but somewhat rough, with scattered piligerous punctae, punctulae, and fine striae; flagellum with a somewhat distinct 4-segmented antennal club; mesosoma mostly strongly rugose to rugoreticulate, with finer reticulae, becoming punctae, on pronotal side and anepisternum, rugae near anterior margin of pronotal dorsum usually with a transverse orientation; propodeal dorsum and declivity with transverse carinulae; promesonotum in profile low-domed and roughly symmetrical, but anterior declivity somewhat shorter and steeper; metantoal grove well-demarcated, of moderate width and depth; anterodorsal margin of propodeum with a small welt; propodeal spines present and of moderate length (PSL 0.16–0.19, PSI 1.8–2.2); petiole appearing of moderate length (PL/HW 0.52–0.55); petiolar node relatively small (PH/PL 0.53–0.56), domed, but slightly asymmetrical in profile, with the anterior face longer and more sloping than posterior face; postpetiole subspherical, without a prominent dorsal node; anterior faces of petiolar and postpetiolar nodes smooth and shiny, anterior and lateral sides with a variable number of rugulae and faint punctae; ventral surfaces of waist segments punctate; most of body dorsum with short standing pilosity; setae on scape mostly subdecumbent; gastral setae short, and clearly bilayered, with a layer of suberect setae, and a layer of slightly shorter but denser subdecumbent setae; setae on legs mostly appressed with some suberect setae on coxae and femoral venters.

#### Queen description.

 (1 measured) HL 0.78, HW 0.71, FLD 0.26, PCW 0.04, SL 0.59, EL 0.17, ACL 0.57, ML 1.10, PrW 0.63, PSL 0.21, SDL 0.12, PL 0.41, PH 0.22, PW 0.18, PPL 0.22, PPH 0.21, PPW 0.18, MFL 0.66, MTL 0.55, CI 90, SI 84, REL 24, FLI 37, PSI 1.8, MFI 107, ACI1 66, ACI2 97.

Same as worker except for standard queen modifications and as follows: pronotum with transverse rugae/rugoreticulae; mesoscutum more rugose than rugoreticulate, with rugae longitudinal in orientation; scutellum with a central patch of smooth cuticle surrounded by longitudinal rugae; mesopleuron partly smooth and shiny.

#### Male.

Unknown.

#### Biology.

This species has been collected from only a few samples of sifted leaf litter from the forest floor. It inhabits montane wet forest from approximately 1200–1400 m elevation. The shape of the anterior margin of the clypeus is unique among *Stenamma* species. Most peculiar is the strongly projecting median lobe, which forms a tooth-like protuberance. This trait probably correlates with diet specialization, but nothing is known yet about prey choice in this species.

#### Comments.

The structure of the clypeus is unique among known *Stenamma* species. Thus, *Stenamma cusuco* should not be confused with any other species.

Although support values are low, molecular phylogenetic results indicate that *Stenamma cusuco* belongs to a clade that includes *Stenamma hojarasca* and *Stenamma ochrocnemis* (Branstetter unpublished data).

#### Material examined.

See type material.

### Stenamma diversum

Mann

http://species-id.net/wiki/Stenamma_diversum

**Figure 74.**
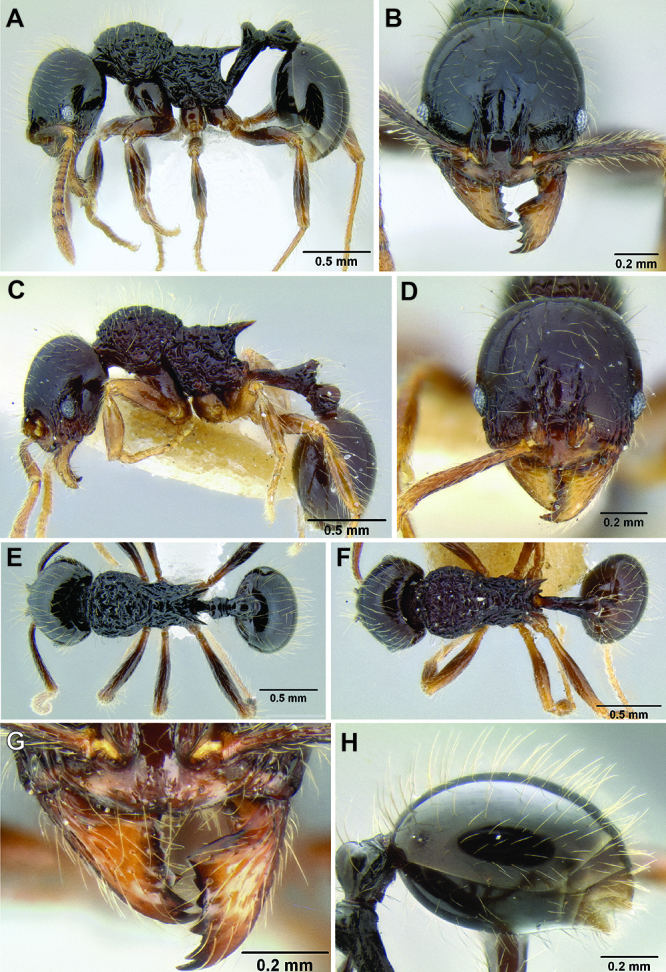
*Stenamma diversum*. **A, B, E, G, H** Worker (CASENT0606723) **C, D, F** Lectotype worker (USNMCOTYPE24447).

**Figure 75.**
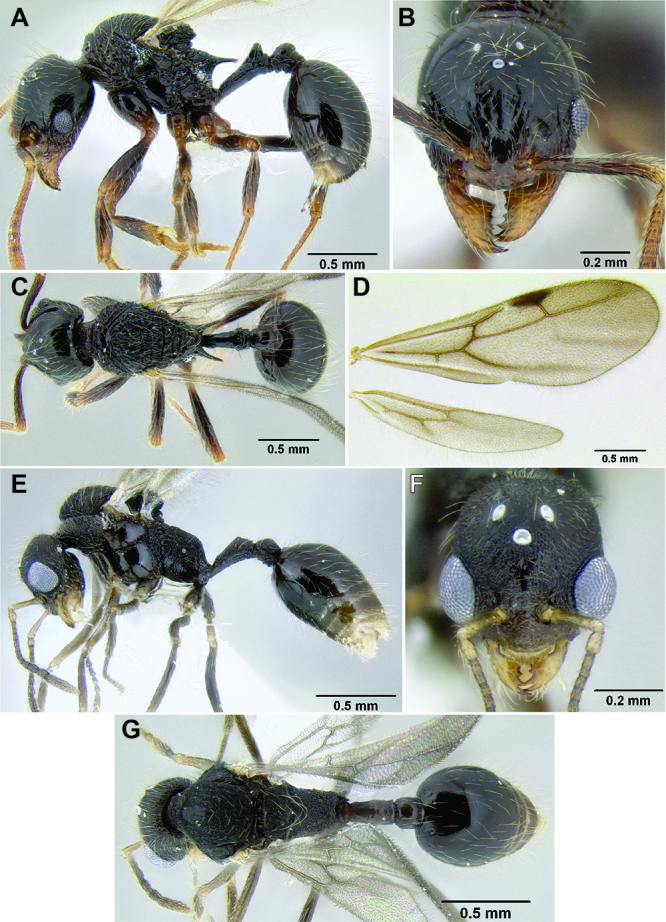
*Stenamma diversum* **A** Queen (CASENT0606782), profile **B** Same, face **C** Same, dorsum **D **Same, wings **E** Male (CASENT0622357), profile **F** Same, face **G** Same, dorsum.

**Figure 76.**
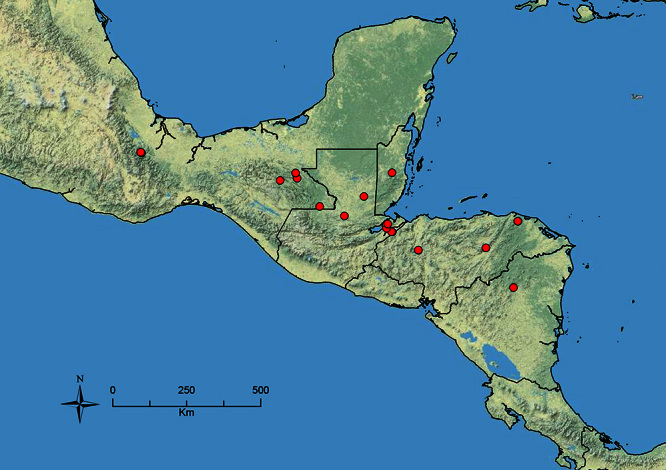
Distribution map of *Stenamma diversum*.

Worker: Figure 74 ; Queen: Figure 75A–D , Male: Figure 75E–G ; Map: Figure 76

Stenamma diversum Mann, 1922: 20. Lectotype worker (**here designated**): HONDURAS, [Atlántida]: Lombardia, [ca. 15.567°N, 87.283°W], Feb-Mar 1920, collected beneath a stone (W. M. Mann) (USNM, Cotype No. 24447, specimen CASENT0194018) [examined]. Smith, 1962: 33, worker description. Branstetter, 2009: worker images. Branstetter, 2012: phylogeny.

#### Worker diagnosis.

Integument mostly black and shining; medium-sized species (see HL, ML, PrW below); head mostly smooth and shiny; mesosoma reticulately costate to coarsely rugoreticulate; propodeal spines long and robust (PSL 0.28–0.34, PSI 3.0-3.7); frontal lobes dorsolaterally expanded, obscuring the torular lobes in full-face view (FLD 0.25-0.29, FLI 35-38); eye of moderate to large size (EL 0.11–0.15, REL 16–20), oval-shaped, with 7–9 ommatidia at greatest diameter; anterior margin of clypeus with shallow median emargination; basal margin of mandible straight, without a notch or substantial depression; pilosity on gastral dorsum long, flexuous, and relatively sparse. *Similar species*: *Stenamma lobinodus*, *Stenamma tico*.

#### Geographic range.

Southern Mexico to Nicaragua.

#### Worker description.

(11 measured) HL 0.74–0.81 (0.76), HW 0.69–0.78 (0.72), FLD 0.25–0.29 (0.27), PCW 0.06–0.09 (0.08), SL 0.58–0.66 (0.62), EL 0.11–0.15 (0.13), ACL 0.52–0.56 (0.54), ML 0.95–1.08 (0.99), PrW 0.55–0.61 (0.56), PSL 0.28–0.34 (0.29), SDL 0.08–0.11 (0.08), PL 0.44–0.50 (0.44), PH 0.22–0.25 (0.23), PW 0.18–0.24 (0.20), PPL 0.18–0.21 (0.19), PPH 0.19–0.22 (0.20), PPW 0.18–0.23 (0.21), MFL 0.71–0.78 (0.72), MTL 0.56–0.63 (0.59), CI 93–97 (95), SI 84–88 (85), REL 16–20 (18), FLI 35–38 (37), PSI 3.0–3.7 (3.5), MFI 96–100 (100), ACI1 65–67 (65), ACI2 85–90 (89).

Medium-sized species; general body color black, with mandibles, clypeus and appendages dark brown to yellow-brown; setae golden; mandible with 6 teeth, consisting of 3 distinct apical teeth, a basal tooth, and 2–3 inner teeth, which are often worn and indistinct; basal margin of mandible straight, without a distinct notch or depression; dorsal surface of mandible mostly smooth and shining, with scattered piligerous punctae, and several weak striae extending from base; anterior clypeal margin with shallow median emargination; median lobe of clypeus with a pair of faint carinulae that diverge toward anterior margin, apex of lobe with a faint transverse carinula, remainder of clypeus mostly smooth and shining; posterior extension of clypeus between frontal lobes broad, with subparallel sides (PCW 0.06–0.09); frontal lobes expanded dorsolaterally (FLD 0.25-0.29, FLI 35-38), with torular lobes obscured in full-face view; head roughly oval-shaped, slightly longer than broad (CI 93-97), with posterior margin flat or gently curving, never depressed medially; eyes of moderate size (EL 0.11–0.15, REL 16-20), oval-shaped, with 7–9 ommatidia at greatest diameter; face largely smooth and shining, with faint carinulae and punctae on gena, scattered piligerous punctae elsewhere; scape short, not surpassing posterior margin of head when laid back (SI 84-88); dorsal surface of scape striate; funiculus with distinct 4-segmented antennal club; mesosoma compact, shiny, and almost entirely reticulately costate (specimens vary considerably in sharpness, coarseness, and orientation of costae); propodeal declivity smooth; promesonotum in profile dome-shaped, roughly symmetrical; propodeal spines long and robust (PSL 0.28–0.34, PSI 3.0-3.7), usually projecting dorsoposteriorly; petiole relatively long and wedge-shaped (PL/HW 0.60-0.65), node variable, appearing rather robust to slightly more gracile, always angled so that the apex points posteriad; anterior slope of petiole usually long and rising gradually from peduncle, but sometimes shorter and rising more abruptly; posterior slope of petiole short and nearly vertical; dorsum of petiolar node viewed from posterior side flat, to depressed medially, to slightly convex; dorsal portion of petiolar node distinctly wider than ventral portion; postpetiolar node in profile smaller than petiolar node (PPH/PH 0.85–0.94), dome-shaped, slightly asymmetrical, with anterior slope longer and more sloping than posterior slope; petiole and postpetiole generally smooth and shiny, nodes with several deep furrows, ventral surfaces faintly punctate; gaster mostly smooth and shiny, with scattered piligerous punctae, and furrows on anterior constriction where gaster inserts into postpetiole; most of body with a relatively sparse layer of long, flexuous setae; setae on scapes and legs varying from mostly suberect to mostly decumbent; setae on femoral venters and coxae always longer and suberect to subdecumbent.

#### Queen description.

(5 measured) HL 0.73–0.80 (0.80), HW 0.69–0.78 (0.78), FLD 0.25–0.30 (0.30), PCW 0.07–0.10 (0.10), SL 0.58–0.65 (0.65), EL 0.17–019 (0.18), ACL 0.51–0.59 (0.59), ML 1.05–1.17 (1.17), PrW 0.63–0.71 (0.69), PSL 0.30–0.33 (0.32), SDL 0.09–0.11 (0.09), PL 0.46–0.53 (0.51), PH 0.24–0.26 (0.26), PW 0.21–0.23 (0.23), PPL 0.21–0.25 (0.22), PPH 0.23–0.25 (0.25), PPW 0.23–0.25 (0.25), MFL 0.70–0.79 (0.79), MTL 0.56–0.64 (0.64), CI 94–98 (97), SI 82–86 (84), REL 23–27 (24), FLI 26–38 (28), PSI 2.9–3.4 (3.4), MFI 98–99 (99), ACI1 65–66 (65), ACI2 86–90 (90).

Same as worker except for standard queen modifications and the following: costae on mesoscutum with a decidedly longitudinal orientation, but often wavy, and usually with some reticulation anteriorly; costae on side of propodeum longitudinal in orientation; mesopleuron mostly smooth and shiny; wing venation as in Figure 75D.

#### Male.

See Figure 75E–G.

#### Biology.

*Stenamma diversum* occurs in relatively pristine wet forest habitats from sea level to about 1,100 m elevation. It is a particularly interesting species because it has convergently evolved many of the same nesting behaviors as *Stenamma alas* and *Stenamma expolitum*, even though it is not closely related (Branstetter 2012, unpublished data). Like these species, *Stenamma diversum* is a specialist inhabitant of areas dominated by red clay substrate (Branstetter pers. obs.). Nests are commonly found in banks bordering streams or trails, in steep slopes, and sometimes in small patches of vertical clay that form under roots at tree bases. The architecture of a nest of *Stenamma diversum* is very similar to that of *Stenamma alas*. The external portion of the nest consists of a small, ear-like turret that is usually sunk into a shallow alcove. At the center of the ear is the nest entrance, which leads to a single, small chamber. Unlike *Stenamma alas* or *Stenamma expolitum*, there is only a single nest per colony and colonies are quite small, with a single queen and maybe a dozen workers. Also, *Stenamma diversum* does not have a “door pebble” to be used to block the nest entrance from predators, as reported by Longino (2005) for *Stenamma alas* and *Stenamma expolitum*. Workers of *Stenamma diversum* are slow moving and appear to be most active during the day. The convergent nature of *Stenamma alas* and *Stenamma diversum* nests is striking and begs further investigation into the adaptive significance of these structures. It is possible that the structure is an adaptation to living in very wet, exposed environments. Inside mature *Stenamma diversum* nests I have often noticed a dark material covering the nest walls. What this material is and what function it serves also needs investigation. Given their nesting habits, it is somewhat odd that the collection of the type series was made “beneath a stone.” Perhaps this was on clay bank.

#### Comments.

*Stenamma diversum* and *Stenamma tico* are sister species and together form the *diversum* species group. This group is defined by the following characters: head mostly smooth and shiny; mesosoma mostly reticulately costate; promesonotum in profile low-domed, roughly symmetrical; postpetiole in profile without a distinct dorsal lobe that projects posteriad over postpetiole (as in *Stenamma lobinodus* and other *lobinodus* group species); basal margin of mandible straight; anterior clypeal margin with a median emargination. Based on DNA sequence data *Stenamma vexator* is likely sister to the *diversum* species group, but morphological characters could not be found to diagnose the entire clade.

*Stenamma diversum* is probably the most distinctive *Stenamma* species, but it does bear some similarity to its sister taxon *Stenamma tico* and the more distantly related *Stenamma lobinodus*, both of which share roughly the same color and sculpture pattern. It is easy to separate *Stenamma diversum* from these other species by comparing the propodeal spines and the frontal lobes. In *Stenamma tico*, the spines are absent or at most form small dorsally projecting tubercles (PSL 0.14–0.18, PSI 1.4–1.9). In *Stenamma lobinodus*, the propodeal spines are usually well developed, but they are shorter than those of *Stenamma diversum* (PSL < 0.23 vs. > 0.27, PSI < 2.6 vs. > 3.0). *Stenamma diversum* is the only species among these three to have greatly expanded frontal lobes, causing the underlying torular lobes to be covered in full-face view. In the other species, the torular lobes are always visible. The frontal lobes of *Stenamma lobinodus* are especially reduced. Geography also can help separate these species. *Stenamma diversum* occurs in sympatry with *Stenamma lobinodus* only in Oaxaca, Mexico (although never collected together at the same site) and with *Stenamma tico* only in northern Nicaragua (found in sympatry at Cerro Saslaya and Cerro Musún).

#### Material examined.

**BELIZE: *Cayo***: Caves Branch, [ca. 17.13°N, 88.70°W], 4-14 Aug 1972 (S. & J. Peck); **GUATEMALA: *Alta Verapaz***: Muchbilhá, 15.86966°N, 90.14254°W, 184m, 9 Nov 2009 (L. Sáenz); ***Izabal***: Carboneras Sn. Gil, 15.63728°N, 88.82826°W, 415m, 19 Nov 2009 (L. Sáenz); 5km NW Morales, 15.51441°N, 88.86459°W, 250m, 17 May 2009 (LLAMA); 16km ESE Morales, 15.41186°N, 88.71671°W, 410m, 19 May 2009 (LLAMA); Finca La Firmeza, 10.7km SE Morales, 15.4067°N, 88.6967°W, 500m, 19 Sep 2008 (M. G. Branstetter); ***Petén***: 13km NW Machaquilá, 16.44603°N, 89.54940°W, 400m, 27 May 2009 (LLAMA); **HONDURAS: *Comayagua***: P.N. Cerro Azul Meambar, 14.86601°N, 87.89955°W, 880m, 21 May 2010 (LLAMA); P.N. Cerro Azul Meambar, 3.4km ESE La Guama, 14.8722°N, 87.9053°W, 760m, 26 Sep 2008 (M. G. Branstetter); ***Gracias a Dios***: Las Marias, 15.72245°N, 84.88078°W, 510m, 10 Jun 2010 (LLAMA); Las Marias, 15.71844°N, 84.87811°W, 370m, 10 Jun 2010 (M. G. Branstetter); ***Olancho***: 11km NNE Catacamas, 14.94493°N, 85.84928°W, 1100m, 12 May 2009 (J. Longino); **MEXICO:** ***Chiapas***: Lago Metzabok, 17.12622°N, 91.63056°W, 570m, 5 Jun 2008 (LLAMA); Nahá, 16.97444°N, 91.58702°W, 180m, 24 Jun 2008 (LLAMA); Ocosingo, [approx. 16.906°N, 92.099°W], 2 Jun 1969 (J. M. Campbell); Playón de la Gloria, 16.14826°N, 90.89704°W, 180m, 24 Jun 2008 (LLAMA); ***Oaxaca***: 5.7km SW Valle Nacional, 17.73067°N, 96.3304°W, 560m, 13 Aug 2009 (M. G. Branstetter); 7.5km S Valle Nacional, 17.70752°N, 96.30516°W, 680m, 12 Aug 2009 (M. G. Branstetter); **NICARAGUA: *Jinotega***: P.N. Cerro Saslaya, 13.77171°N, 85.01263°W, 1110m, 12 May 2011 (LLAMA).

### Stenamma excisum sp. n.

urn:lsid:zoobank.org:act:D105B73A-B44E-4420-882E-6C4180D8E149

http://species-id.net/wiki/Stenamma_excisum

**Figure 77.**
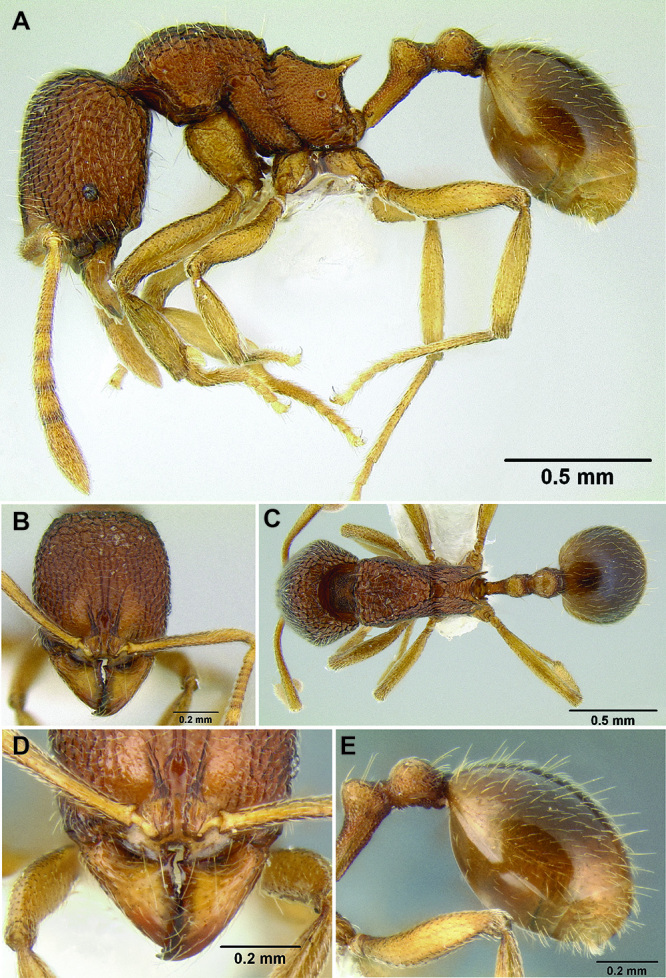
*Stenamma excisum* holotype worker (CASENT0621834) **A** Profile **B** Face **C** Dorsum **D **Anterior clypeal margin in anterodorsal view **E** Gaster.

**Figure 78.**
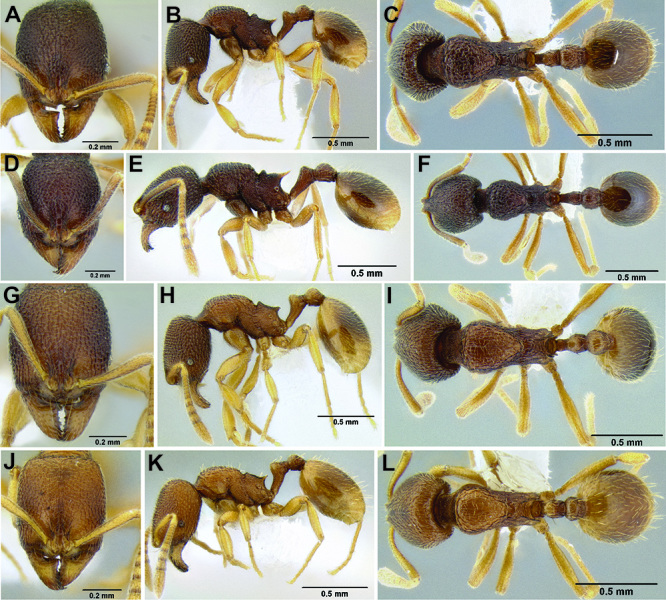
*Stenamma excisum* worker variants 1. Face, profile, dorsal views **A–C** Variant 1 (CASENT0605563) **D–F** Variant 2 (CASENT0605441) **G–I** Variant 3 (CASENT0605500) **J–L** Variant 4 (CASENT0605743).

**Figure 79.**
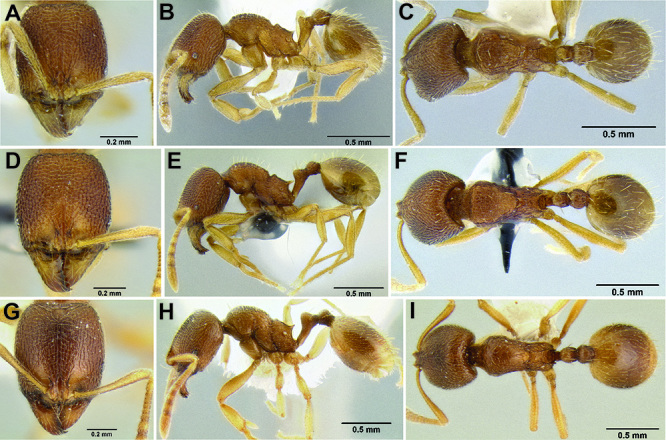
*Stenamma excisum* worker variants 2. Face, profile, dorsal views **A–C** Variant 5 (CASENT0126237) **D–F** Variant 6 (CASENT0126243) **G–I** Variant 7 (CASENT0622434).

**Figure 80.**
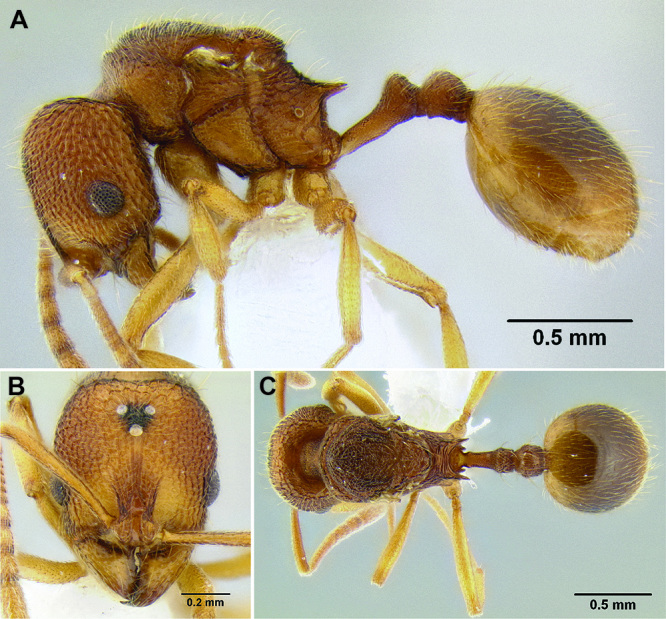
*Stenamma excisum* paratype queen (CASENT0621799) **A** Profile **B** Face **C** Dorsum.

**Figure 81.**
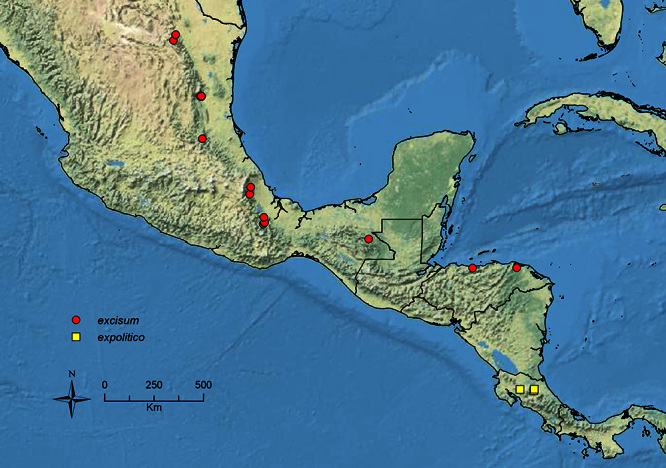
Distribution map of *Stenamma excisum* (circles) and *Stenamma expolitico* (squares).

Worker: Figures 77- 79 ; Queen: Figure 80 ; Map: Figure 81

Stenamma mgb16 [variant 1 below] Branstetter, 2012: phylogeny.

#### Type material.

*Holotype worker*. HONDURAS, Atlántida: 12km SW La Ceiba, 15.69150°N, 86.86151°W ±20m, 280m, 19 Jun 2010, tropical rainforest, ex sifted leaf litter (LLAMA, collection Wa-C-09-2-44) [USNM, specimen CASENT0621834]. *Paratypes*: same data as holotype but 15.69449°N, 86.86330°W ±20m, 200m (Wa-C-09-1-27) [1dq, 1w, CAS, CASENT0621799, CASENT0621801], [1w, EAPZ, CASENT0621800], [1w, FMNH, CASENT0623304], [1w, INBio, CASENT0623305], [1w, LACM, CASENT0623306]; 15.69175°N, 86.86091°W ±20m (LLAMA, Wa-C-09-2-04) [1dq, 1w, MCZ, CASENT0623307, CASENT0623309], [1dq, 1w, USNM, CASENT0623308, CASENT0623310]; 15.69449°N, 86.86344°W ±20m (LLAMA, Wa-C-09-1-24) [1w, UCD, CASENT0621793]; 15.69134°N, 86.86137°W ±20m, 280m, 19 Jun 2010 (LLAMA, Wa-C-09-2-40) [1dq, 1w, MGBPC, CASENT0623541, CASENT0623540].

#### Worker diagnosis.

Integument mostly orange-brown to brown; small-sized species (see HL, ML, PrW below); anterior clypeal margin often with a deep median excision, but sometimes reduced to a shallow median emargination; basal margin of mandible straight; face densely sculptured and mostly rugoreticulate; mesosoma densely sculptured with punctae, rugae, and/or rugoreticulae; eye small (EL 0.04–0.10, REL 8–14), subcircular, with 2–4 ommatidia at greatest diameter; median portion of clypeus angled dorsoventrally, causing it to be mostly hidden in full-face view; posterior extension of clypeus between antennal insertions very narrow (PCW 0.01–0.03), with border of antennal insertions sometimes touching anteriorly; propodeal spines present, short to long (PSL 0.09–0.20, PSI 1.8–5.4); pilosity on gastral tergites distinctly bilayered, with a layer of sparse suberect setae (varying in thickness and length), and a layer of decumbent to appressed setae. *Similar species*: *Stenamma saenzae*.

#### Geographic range.

Mexico (Atlantic slope) to Honduras.

#### Worker description.

(25 measured) HL 0.49–0.73 (0.71), HW 0.43–0.67 (0.63), FLD 0.09–0.14 (0.14), PCW 0.01–0.03 (0.02), SL 0.38–0.61 (0.59), EL 0.04–0.10 (0.06), ACL 0.41–0.62 (0.62), ML 0.57–0.88 (0.88), PrW 0.29–0.45 (0.43), PSL 0.09–0.20 (0.20), SDL 0.04–0.08 (0.08), PL 0.22–0.34 (0.34), PH 0.13–0.19 (0.18), PW 0.10–0.16 (0.14), PPL 0.11–0.20 (0.19), PPH 0.11–0.16 (0.15), PPW 0.14–0.22 (0.19), MFL 0.37–0.68 (0.66), MTL 0.31–0.56 (0.55), CI 86–94, SI 79–97 (94), REL 8–14 (10), FLI 19–24 (22), PSI 1.8–5.4 (2.5), MFI 95–119 (101), ACI1 64–72 (66), ACI2 98–111 (105).

Small-sized species; general body color mostly orange-brown to brown, with appendages orange-brown to yellow-brown, becoming lighter toward extremities; setae golden; mandible with 5–7 teeth, consisting of 3 distinct apical teeth, a basal tooth, and 1–3 smaller inner teeth/denticles, which are often worn and indistinct; basal margin of mandible straight, without a basal notch or depression; mandible mostly smooth and shiny, with scattered piligerous punctae and some basal striae; anterior clypeal margin often with a deep median excision (type population), but sometimes excision reduced to a shallow emargination; median lobe of clypeus flattened, and angled dorsoventrally, causing it to be mostly invisible in full-face view; surface of clypeus mostly smooth and shiny; posterior extension of clypeus between antennal insertions very narrow (PCW 0.01–0.03), sides subparallel, with border of antennal insertions sometimes touching anteriorly; frontal lobes of moderate width (FLD 0.09–0.14, FLI 19–24), not greatly obscuring torular lobes in full-face view; head appearing subrectangular to oval-shaped (CI 86–94), posterior margin flat to slightly depressed medially; eye small (EL 0.04–0.10, REL 8–14), subcircular, with 2–4 ommatidia at greatest diameter; face densely sculptured, usually mostly rugoreticulate and punctate, with some longitudinal rugae along midline (type population), but sometimes mostly rugose; scape of short to moderate length (SI 79–97), usually not reaching posterior margin of head; scape surface mostly smooth, with scattered piligerous punctae; flagellum with a distinct to very distinct 4-segmented antennal club; mesosoma densely sculptured, dorsum of promesonotum ranging from rugoreticulate (type population), to rugose-punctate, to rugose, to mostly punctate, with rugae longitudinal in orientation; side of pronotum usually punctate, with a few rugulae or rugoreticulae (type population), but sometimes mostly rugose, and with a small patch of smooth cuticle; mesopleuron and side of propodeum mostly punctate, with a variable number of rugulae; dorsum and declivity of propodeum with a few transverse carinulae; promesonotum in profile low-domed and roughly symmetrical; metanotal groove usually well-demarcated and somewhat deep; anterodorsal margin of propodeum often raised into a small to large welt, but sometimes average; propodeal spines present, short to long (PSL 0.09–0.20, PSI 1.8–5.4); petiole appearing moderate to slightly elongate (PL/HW 0.45–0.56), usually with a distinct node, but sometimes node less distinct, making petiole look more wedge-shaped; when distinct, petiolar node in profile, average to slightly enlarged (PH/PL 0.48–0.64), and roughly symmetrical, dorsum of node usually gently rounded and pointing vertically (type population), but sometimes broadly rounded, or nearly angulate and pointing slightly posteriad; postpetiole in profile usually subspherical and appearing similar in size to petiolar node (PPH/PH 0.79–0.96), postpetiole in dorsal view often distinctly wider than petiole, sometimes much wider (PPW/PW 0.55–0.83); petiole and postpetiole usually mostly punctate, with only the anterior faces of nodes smooth, but sometimes nodes completely smooth, or completely punctate; gaster mostly smooth and shiny, with scattered piligerous punctae; most of body dorsum with standing pilosity; pilosity on gastral tergites distinctly bilayered, with a layer of sparse suberect setae (varying in thickness and length), and a layer of decumbent to appressed setae; setae on scapes and legs mostly decumbent to appressed, with some longer suberect setae on femoral venters and coxae.

#### Queen description.

(5 measured) HL 0.55–0.73 (0.73), HW 0.50–0.69 (0.69), FLD 0.11–0.16 (0.16), PCW 0.01–0.02 (0.02), SL 0.42–0.63 (0.63), EL 0.13–0.16 (0.16), ACL 0.43–0.62 (0.62), ML 0.73–1.01 (1.01), PrW 0.40–0.57 (0.57), PSL 0.12–0.22 (0.22), SDL 0.07–0.10 (0.10), PL 0.27–0.38 (0.38), PH 0.15–0.20 (0.20), PW 0.13–0.17 (0.17), PPL 0.14–0.20 (0.20), PPH 0.15–0.19 (0.19), PPW 0.17–0.23 (0.23), MFL 0.43–0.69 (0.69), MTL 0.37–0.58 (0.58), CI 89–93 (93), SI 81–92 (92), REL 22–27 (23), FLI 22–24 (24), PSI 1.6–2.2 (2.2), MFI 99–118 (99), ACI1 63–74 (63), ACI2 97–107 (98).

Same as worker except for standard queen modifications and as follows (comparison with worker and queen from type population only): pronotum rugoreticulate laterad, and punctate mesad; mesoscutum and scutellum rugoreticulate to foveate; propodeum with transverse carinulae that wrap around surface; katepisternum mostly smooth; petiole more elongate.

#### Male.

Unknown.

#### Biology.

A rather uncommon species known almost exclusively from Winkler and Berlese samples of sifted leaf litter, except for one collection from under a stone. *Stenamma excisum* has been collected from 60–2280 m elevation and occurs in a variety of wet forest environments (e.g. tropical rainforest, cloud forest, disturbed mesophyll forest, pine-oak forest, riparian wet forest). The excision in the anterior clypeal margin of most specimens is unique and suggests some sort of diet specialization different from other *Stenamma* species.

#### Comments.

Although quite variable across its range (see below), *Stenamma excisum* is separable from similar species using the characters in the key and diagnosis. Several species are superficially similar to *Stenamma excisum*, but no other species has the same unique clypeal structure. Phylogenetic results indicate that *Stenamma excisum* is sister to *Stenamma lagunum* and that this clade is sister to the remainder of MAC *Stenamma* species (Branstetter 2012, unpublished data).

Stenamma excisum forms a difficult species complex composed of several allopatric variants, which probably constitute distinct species. The type form is known only from two sites in Honduras, where it is unusually abundant in leaf litter samples. The most important features of this form are the rugoreticulate sculpture on the pronotal dorsum and the nearly triangular excision in the anterior clypeal margin. Other characteristics specific to this form are indicated in the species description above (see parenthetical comments). Specimens from the populations in Honduras and the next closest population are quite divergent from one another. Furthermore, the type form seems to be confined to lower elevations, whereas the other variants usually occur at mid elevations. For these reasons, I highly suspect that there are multiple species within *Stenamma excisum* as it is described here. However, too few collections have been made in intervening areas to adequately assess variation and species boundaries.

Variant 1 (Figure 78A–C) is known mainly from mid-elevation sites along the wet eastern slopes of the mountains in Oaxaca, Mexico, but a single specimen is also known from Nahá in Chiapas, Mexico. This variant is very similar to the type population, but differs as follows: smaller overall body size; body color darker, mostly dark orange-brown; sculpture on pronotal dorsum longitudinally rugose, without reticulae.

Variant 2 (Figure 78D–F) occurs in the same area as variant 1, but the two forms have not been collected together. It is similar to variant 1 except for the following: anterior clypeal margin with a shallow median emargination, lacking a deep excision; propodeal spines in profile longer and somewhat sinuous; anterior margin of propodeal dorsum with a more distinct welt; postpetiole in dorsal view much wider than petiolar node (PW/PPW 0.55–0.61).

Variant 3 (Figure 78G–I) is known from a single aberrant specimen collected near variant 2, but from a slightly higher elevation. It differs from variant 2 as follows: anterior clypeal margin intermediate between variant 1 and 2, with a shallow excision; propodeal spines short, almost tuberculate; antennal segments 11 and 12 noticeably bulging; postpetiolar node mostly smooth; postpetiole in dorsal view not greatly expanded; upper layer of setae on gastral dorsum shorter, sparser, and subdecumbent.

Variant 4 (Figure 78J–L) is found mainly in northern Mexico in Tamaulipas (El Cielo) and Nuevo León (Monterrey de Chipinque) states. It is a very small version of *Stenamma excisum* and is quite divergent from the type population. I originally intended to describe it as a separate species, but later found intermediate-looking specimens from allopatric populations in Querétaro and Veracruz, Mexico. It differs from the type population as follows: overall body size much smaller; sculpture reduced; face with only light rugoreticulae, punctae, and carinulae; dorsum of pronotum with sparse longitudinal carinulae, interstices shiny; propodeal spines short; decumbent layer of pilosity on gastral dorsum sparse, suberect layer slightly thickened.

Variant 5 (Figure 79A–C) occurs in Veracruz, Mexico. It is intermediate between the type population and variant 4. It differs from variant 4 as follows: head and body densely punctate; dorsum of pronotum with longitudinal rugulae/carinulae, but interstices punctate; petiolar node reaching a sharper apex, which points distinctly posteriad; suberect layer of pilosity on gastral dorsum (and most of body dorsum) longer.

Variant 6 (Figure 79D–F) is known from two localities in Veracruz, Mexico (Paraje Nuevo, Tetzonapa). It is similar to variant 5, but differs as follows: anterior clypeal margin with a median emargination, lacking an excision; body larger and with distinctly elongate appendages; decumbent layer of gastral pilosity very sparse.

Variant 7 (Figure 79G–I) is known from a single locality in Nuevo León, Mexico (38 km SSW Monterrey). It is very similar to variant 4, but differs as follows: anterior clypeal margin with a median emargination, lacking a deep excision; larger overall size; head larger, more robust, with a distinct median depression in posterior margin; petiolar node in profile broadly domed and more asymmetrical, with a longer moresloping anterior face.

#### Material examined.

**HONDURAS: *Atlántida***: 12km SW La Ceiba, 15.69449°N, 86.86544°W, 200m, 19 Jun 2010 (LLAMA); 12km SW La Ceiba, 15.69150°N, 86.86151°W, 280m, 19 Jun 2010 (LLAMA); ***Gracias a Dios***: Las Marias, 15.70861°N, 84.86211°W, 80m, 8 Jun 2010 (LLAMA); **MÉXICO: *Chiapas***: Nahá, 16.96399°N, 91.59283°W, 985m, 8 Jun 2008 (LLAMA); ***Nuevo Leon***: Monterrey, Chipinque Mesa, [ca. 25.61°N, 100.36°W], 1650m, 22 Jun 1969 (S. & J. Peck); 38km SSW Monterrey, [ca. 25.367°N, 100.467°W], 2280m, 15 Jul 1979 (P. Ward); ***Oaxaca***: Mirador Grande, 17.89844°N, 96.36253°W, 990m, 14 Aug 2009 (M. G. Branstetter); 10.8km SW Valle Nacional, 17.68102°N, 96.33026°W, 1120m, 13 Aug 2009 (M. G. Branstetter); 13.2km SW Valle Nacional, 17.65934°N, 96.33426°W, 1360m, 11 Aug 2009 (M. G. Branstetter); 20.6km SW Valle Nacional, 17.60404°N, 96.37786°W, 1733m, 13 Aug 2009 (M. G. Branstetter); 25km S Valle Nacional, [ca. 17.670°N, 96.330°W], 1200m, 21 May 1971 (S. B. Peck); ***Querétaro***: 29km E Landa de Matamoros, [ca. 21.27°N, 99.16°W], 1600m, 14 Jul 1969 (S. & J. Peck); ***Tamaulipas***: El Cielo, nr Alta Cima, 23.06518°N, 99.20433°W, 980m, 21 Aug 2009 (M. G. Branstetter); El Cielo, 1.2km SE Alta Cima, 23.05005°N, 99.19226°W, 24 Aug 2009 (M. G. Branstetter); 2.5km SSE Alta Cima, 23.03787°N, 99.18941°W, 870m, 24 Aug 2009 (M. G. Branstetter); El Cielo, 1.2km N La Gloria, 23.05717°N, 99.25206°W, 1780m, 23 Aug 2009 (M. G. Branstetter); nr Gomez Farias Rancho del Cielo, [ca. 23.063°N, 99.205°W], 1000m, 7 Aug 1983 (S. & J. Peck); ***Veracruz***: Cordoba, Paraje Nuevo, Nacimiento, [ca. 18.88°N, 96.86°W], 7 Aug 1969 (S. & J. Peck); Fortín, Canyon Río Metlac, [ca.18.90°N, 97.00°W], 5 Aug 1969 (S. & J. Peck); 1.9km S Huatusco, [ca. 19.20°N, 96.95°W], 1344m, 2–5 Aug 1969 (S. & J. Peck); Pueblo Nuevo, nr Tetzonapa, Aug 1953 (E. O. Wilson).

### Stenamma expolitico sp. n.

urn:lsid:zoobank.org:act:4CE4E3F9-6FB0-4859-85B4-B4923E513FCE

http://species-id.net/wiki/Stenamma_expolitico

**Figure 82.**
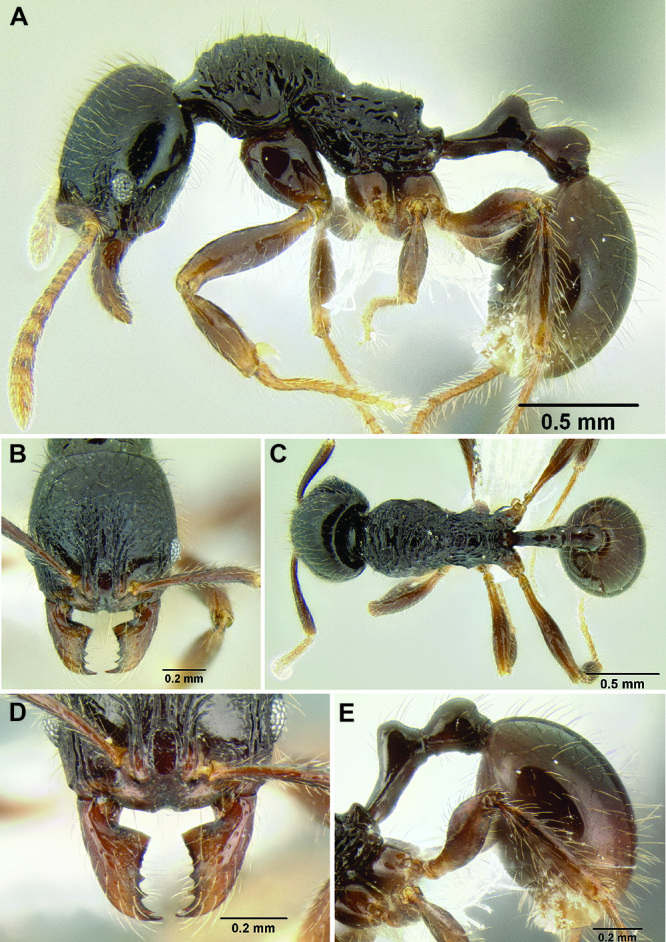
*Stenamma expolitico* holotype worker (INBIO282473) **A** Profile **B** Face **C** Dorsum **D** Anterior clypeal margin in anterodorsal view **E** Gaster.

Worker: Figure 82 ; Map: Figure 81

#### Type material.

*Holotype worker*. COSTA RICA, Alajuela Prov.: Rio Peñas Blancas, 10.3167°N, 84.7167°W, 800m, 23 May 1990, wet forest, ex sifted leaf litter (J. Longino, collection JTL2701-s) [INBio, specimen INBIOCRI001282473]. *Paratypes*: COSTA RICA, Heredia: 16km N Vol. Barba, 10.267°N, 84.083°W, 1020m, 9 Jul 1986 (J. Longino, JTL1340-s) [1w, USNM, CASENT0623094]; same data but 22km N Volcan Barba, 10.333°N, 84.067°W, 500m, 5 Mar 1985 (J. Longino, JTL1340-s) [1w, LACM, CASENT0623093]; 16km SSE La Virgen, 10.267°N, 84.083°W, 1100m, 14–17 Mar 2001 (ALAS, 11/WF/02/28) [1w, UCD, INB0003212462].

#### Worker diagnosis.

Integument mostly black to dark red-brown; medium-sized species (see HL, ML, PrW below); petiole and postpetiole almost completely smooth and shiny, with only a few faint vestigial punctae sometimes present; postpetiole in profile bulging, globular, appearing more voluminous than petiolar node; face with a fan of carinae/rugae extending from frontal lobes to approximately ¾ distance to posterior margin of head, remainder of head smooth and shiny; dorsum of promesonotum with distinctive transverse furrows that reticulate toward posterior margin; propodeal spines absent to tuberculate (PSL 0.13–0.14, PSI 1.0–1.1), dorsal and declivitous faces of propodeum in profile flat, forming a blunt 90° angle; eye relatively large (EL 0.13–0.15, REL 19), with 8 ommatidia at greatest diameter; anterior clypeal margin with median emargination; basal margin of mandible straight; gastral setae long, sparse, and uniformly suberect. *Similar species*: *Stenamma alas*, *Stenamma expolitum*.

#### Geographic range.

Costa Rica.

#### Worker description.

(3 measured) HL 0.77–0.86 (0.77), HW 0.71–0.81 (0.71), FLD 0.21–0.24 (0.22), PCW 0.07–0.08 (0.07), SL 0.62–0.69 (0.62), EL 0.13–0.15 (0.13), ACL 0.60–0.65 (0.60), ML 1.07–1.20 (1.07), PrW 0.54–0.61 (0.54), PSL 0.13–0.14 (0.13), SDL 0.12–0.13 (0.12), PL 0.40–0.42 (0.40), PH 0.23–0.25 (0.23), PW 0.16–0.17 (0.16), PPL 0.24–0.27 (0.27), PPH 0.21–0.22 (0.21), PPW 0.21–0.22 (0.21), MFL 0.75–0.87 (0.75), MTL 0.62–0.70 (0.62), CI 92–94 (92), SI 85–88 (88), REL 19 (19), FLI 29–31 (31), PSI 1.0–1.1 (1.1), MFI 70–73 (70), ACI1 63–64 (64), ACI2 95–98 (97).

Medium-sized species; general body color black to dark red-brown, with brown patches on gaster; appendages mostly brown, changing to yellow-brown at extremities; setae golden brown; mandible with 5–7 teeth, consisting of 4 distinct apical teeth, a distinct basal tooth, and 1–2 worn denticles in between; basal margin of mandible straight, without a basal notch or depression; mandible surface with scattered piligerous punctae and faint striae; anterior clypeal margin with a median emargination; median lobe of clypeus obliquely flattened, mostly smooth and shiny, with a short transverse carinula near anterior margin, remainder of clypeus mostly smooth and shiny; posterior extension of clypeus between antennal insertions somewhat wide (PCW 0.07–0.08), with sides subparallel; frontal lobes somewhat narrow (FLD 0.0.21–0.24, FLI 29–31), not obscuring torular lobes in full-face view, outer margin of frontal lobes nearly parallel, almost without a discernable lateral apex; head subrectangular to oval-shaped (CI 92–94), with posterior margin flat, not depressed medially; eye relatively large (EL 0.13–0.15 REL 19), oval-shaped, with 8 ommatidia at greatest diameter; face with a fan of coarse carinae or costae extending from the frontal lobes to no more than ¾ distance to posterior margin of head; gena with short rugae and rugoreticulae; remainder of head mostly smooth and shiny; scape of moderate length (SI 85–88), not quite reaching posterior margin of head when laid back; scape surface mostly smooth and shiny, with scattered piligerous punctae; flagellum with distinct 4-segmented antennal club; mesosoma densely sculptured, except for a patch of smooth, shiny cuticle on side of pronotum and katepisternum; dorsum of promesonotum with distinctive transverse furrows, which merge together posteriad and appear reticulate; sculpture on propodeum costate to rugose, with orientation transverse on dorsum, and longitudinal on side; mesopleuron rugose; propodeal declivity with faint transverse carinulae; promesonotum in profile low-domed and roughly symmetrical; metanotal grove somewhat indistinct and wide; propodeal spines absent to tuberculate (PSL 0.13–0.14, PSI 1.0–1.1), appearing in profile as a blunt 90° angle that separates dorsal and declivitous faces of propodeum; petiole of moderate length (PL/HW 0.52–0.56); petiolar node in profile robust and somewhat bulging (PH/PL 0.56–0.59), asymmetrical, with a longer more sloping anterior face and a short almost vertical posterior face; node dorsum flat to gently rounded and pointing posteriad; postpetiole in profile large, bulging, appearing slightly more voluminous than petiolar node, outline asymmetrical, with the anterior face longer and more gently sloping, and the posterior face short and vertical, dorsum of node broadly rounded; petiole and postpetiole mostly smooth and shining; gaster mostly smooth and shiny, with scattered piligerous punctae, and very short furrows around anterior constriction; most of body dorsum with long standing pilosity; scape setae suberect to subdecumbent; gastral setae long, somewhat sparse, and uniformly suberect; setae on legs suberect to subdecumbent, with longer suberect setae on coxae and femoral venters.

#### Queen.

Unknown.

#### Male.

Unknown.

#### Biology.

*Stenamma expolitico* is known from only four specimens, collected from sifted leaf litter in wet forest between 500–1100 m elevation.

#### Comments.

*Stenamma expolitico* belongs to the *expolitum* species group, which also includes *Stenamma alas* and *Stenamma expolitum* (a diagnosis for this group is given under *Stenamma expolitum* below). *Stenamma expolitico* can be separated from *Stenamma alas* and *Stenamma expolitum* by the presence of transverse furrows on the promesonotal dorsum, and by the shape of the propodeum in profile, which in *Stenamma expolitico* forms a blunt 90° angle where the dorsal and declivitous faces meet.

I am not completely convinced that *Stenamma expolitico* is a good species because it is known from only a few workers and is somewhat intermediate in morphology between *Stenamma expolitum* and *Stenamma tico*. It could be a hybrid form, or a rare variant of *Stenamma expolitum* or *Stenamma alas*. I choose to recognize it as separate species here, because, although rare, it does occur at several sites in sympatry with the other *expolitum* group species, and it is easily identifiable. Furthermore, many nests of *Stenamma alas* and *Stenamma expolitum* have been excavated, and workers with *Stenamma expolitico* -like morphology have never been found. More material of *Stenamma expolitico*, especially from nest series, is needed to test this hypothesis and confirm its status as a real biological species.

#### Material examined.

See type material.

### Stenamma expolitum

Smith

http://species-id.net/wiki/Stenamma_expolitum

**Figure 83.**
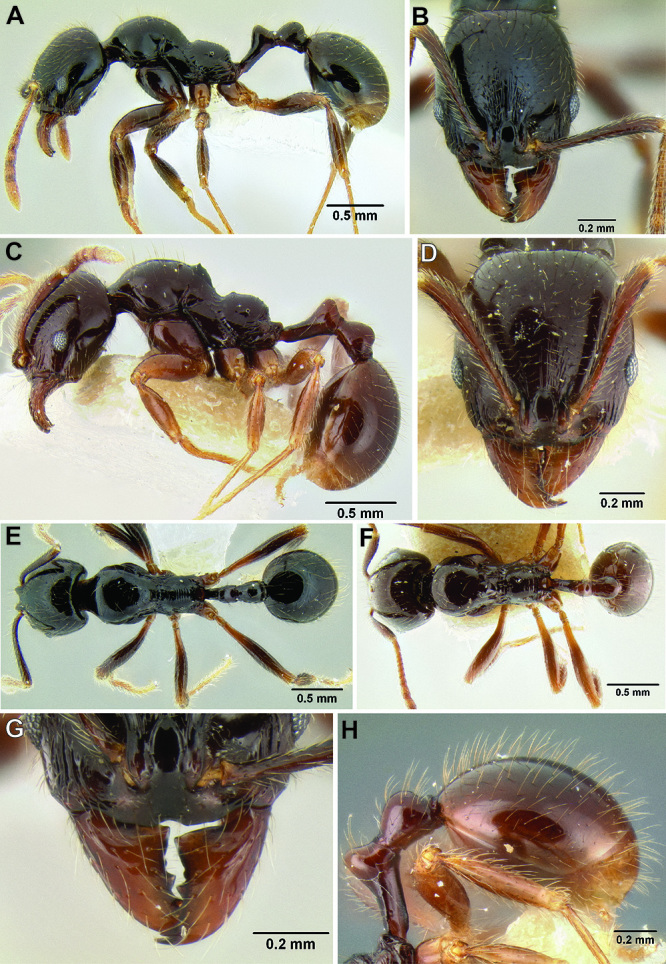
*Stenamma expolitum* **A, B, E, G** Worker (CASENT0600043) **C, D, F, H** Paratype worker (CASENT0126347).

**Figure 84.**
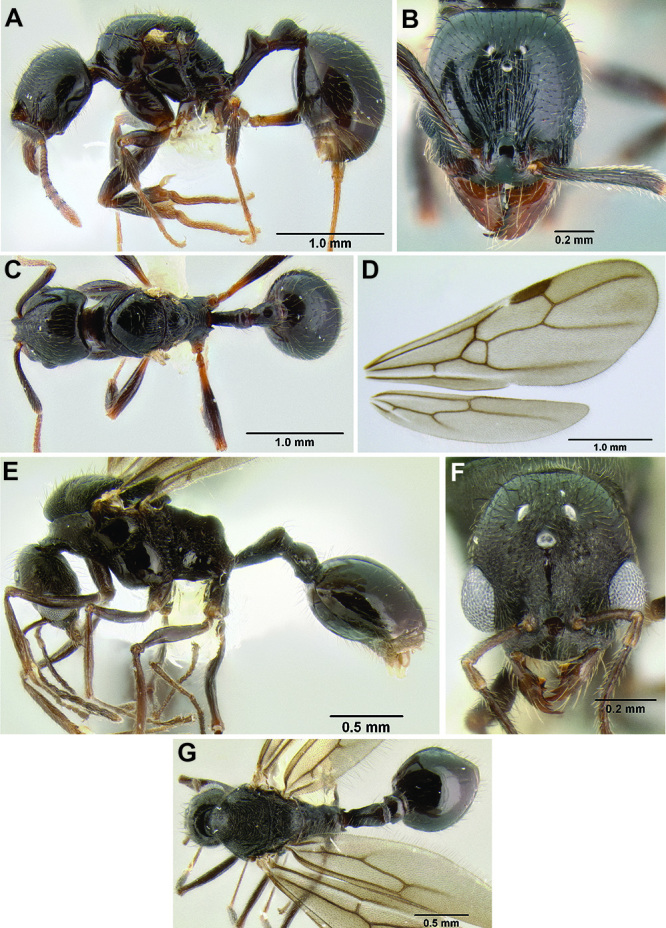
*Stenamma expolitum* **A** Queen (CASENT000005582), profile **B** Same, face **C** Same, dorsum **D** Same, wings **E** Male (JTLC000003501), profile **F** Same, face **G** Same, dorsum.

**Figure 85.**
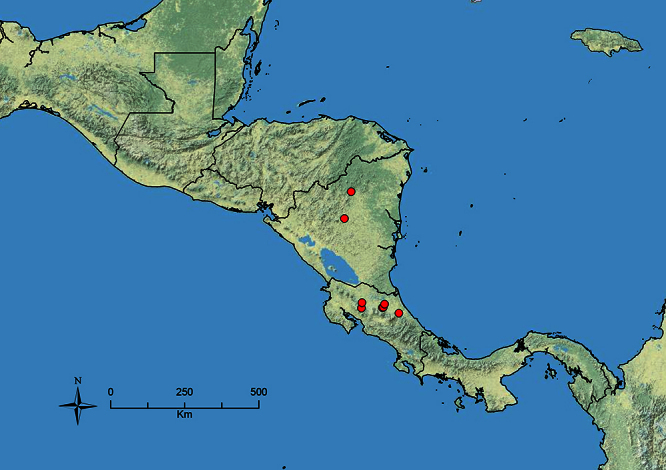
Distribution map of *Stenamma expolitum*.

Worker: Figure 83 ; Queen: Figure 84A–D ; Male: Figure 84E–G ; Map: Figure 85

Stenamma expolitum Smith, M. R. 1957: 36. Holotype worker: COSTA RICA, Santa Clara [Limón] Province: Colombiana Farm, [ca. 10.167°N, 83.583°W], March-April 1924 (W. M. Mann) (USNM, Type No. 65967) [examined]. Longino, 2005: biology. Branstetter, 2009: worker images, phylogeny. Branstetter, 2012: phylogeny.

#### Worker diagnosis.

Integument mostly black, with legs dark brown, changing to orange-brown only at extremities and joints; medium-sized species (see HL, ML, PrW below); petiole and postpetiole almost completely smooth and shiny, with only a few faint vestigial punctae sometimes present; postpetiole in profile bulging, globular, usually appearing more voluminous than petiolar node; face almost completely smooth and shiny, except for variable number of faint carinulae and punctae; promesonotum almost completely smooth and shiny; eye relatively large (EL 0.13–0.16, REL 18–21), oval-shaped, with 7–9 ommatidia at greatest diameter; anterior margin of clypeus with median emargination; basal margin of mandible straight; propodeal spines absent (PSL 0.12–0.14, PSI 0.9). *Similar species*: *Stenamma atribellum*, *Stenamma alas*, *Stenamma expolitico*.

#### Geographic range.

Nicaragua to Costa Rica.

#### Worker description.

(10 measured) HL 0.75–0.93, HW 0.66–0.83 (0.82), FLD 0.20–0.23 (0.23), PCW 0.05–0.08 (0.06), SL 0.63–0.78 (0.76), EL 0.13–0.16 (0.16), ACL 0.63–0.74 (0.73), ML 1.02–1.26 (1.26), PrW 0.51–0.62 (0.61), PSL 0.12–0.14 (0.13), SDL 013–0.15 (0.14), PL 0.39–0.47 (0.47), PH 0.21–0.27 (0.26), PW 0.15–0.19 (0.18), PPL 0.24–0.30 (0.27), PPH 0.20–0.25 (0.24), PPW 0.19–0.24 (0.23), MFL 0.74–0.95 (0.91), MTL 0.58–0.75 (0.73), CI 87–92 (92), SI 91–96 (92), REL 18–21 (19), FLI 27–30 (28), PSI 0.9 (0.9), MFI 85–91 (90), ACI1 60–65 (64), ACI2 92–101.

Medium-sized species; general body color black to dark red-brown, with patches of dark brown on gaster; appendages mostly dark brown with joints and extremities a lighter orange-brown; setae dark golden brown; mandible usually with 6–7 teeth, consisting of 4 distinct apical teeth, a distinct basal tooth, and 1–2 inner teeth/denticles, which are usually worn and indistinct; basal margin of mandible straight, without a basal notch or depression; mandible surface mostly smooth and shiny, with scattered piligerous punctae and a few faint striae; anterior clypeal margin with median emargination; median lobe of clypeus obliquely flattened, mostly smooth and shiny, except for transverse carinula near anterior margin, remainder of clypeus mostly smooth and shiny; posterior extension of clypeus between antennal insertions somewhat wide (PCW 0.05–0.08), with sides subparallel to slightly diverging posteriad; frontal lobes of moderate width (FLD 0.20–0.23, FLI 27–30), not covering torular lobes in full-face view; head roughly oval-shaped (CI 87–92), with posterior margin flat, not depressed medially; eye relatively large (EL 0.13–0.16, REL 18–21), oval-shaped, with 7–9 ommatidia at greatest diameter; face completely smooth and shiny, except for variable number of very faint carinulae extending from frontal lobes to about midpoint of head, a few carinulae on gena, and scattered piligerous punctae; scape of moderate length (SI 91–96), usually reaching posterior margin of head when laid back; scape surface mostly smooth and shiny, with scattered piligerous punctae; funiculus with distinct 4-segmented antennal club; mesosoma almost completely smooth and shiny, except for transverse carinulae on propodeal dorsum, carinae along metanotal grove, and a few scattered rugulae and piligerous punctae; metanotal grove somewhat shallow and wide; propodeal dorsum in profile flat to distinctly convex; propodeal spines absent, at most forming nearly imperceptible nubs (PSL 0.12–0.14, PSI 0.9); petiole of moderate length (PL/HW 0.53–60); petiolar node in profile robust (PH/PL 0.54–0.60), wedge-shaped, with the anterior face long and sloping and the posterior face shorter and nearly vertical; node dorsum rounded, pointing vertically or slightly posteriad; petiolar node in profile robust, more globular than petiolar node, but similarly asymmetrical with long anterior face and short vertical posterior face; petiole and postpetiole mostly smooth and shining, with a few vestigial punctae; gaster smooth and shiny, with only scattered piligerous punctae; most of body dorsum with long standing pilosity; scape setae suberect to subdecumbent; setae on gastral tergites long, sparse, and uniformly suberect; setae on legs suberect to subdecumbent, with longer suberect setae on coxae and femoral venters.

#### Queen description.

(5 measured) HL 0.87–0.96 (0.90), HW 0.78–0.87 (0.81), FLD 0.24–0.27 (0.25), PCW 0.07–0.08 (0.07), SL 0.72–0.79 (0.76), EL 0.21–0.23 (0.23), ACL 0.70–0.77 (0.71), ML 1.23–1.43 (1.32), PrW 0.67–0.78 (0.74), PSL 0.11–0.15 (0.15), SDL 0.13–0.15 (0.15), PL 0.48–0.53 (0.48), PH 0.27–0.31 (0.29), PW 0.19–0.23 (0.21), PPL 0.30–0.33 (0.30), PPH 0.26–0.30 (0.29), PPW 0.26–0.30 (0.27), MFL 0.87–1.00 (0.93), MTL 0.67–0.79 (0.74), CI 90–92 (90), SI 91–93 (93), REL 26–28 (28), FLI 30–31 (30), PSI 0.9–1.0 (1.0), MFI 86–90 (87), ACI1 60–61 (60), ACI2 94–103 (94).

Same as worker except for standard queen modifications and the following: facial sculpture more developed, with distinct carinulae extending from frontal lobes to ocelli, and some carinulae on gena; pronotum with transverse striae near posterior margin; posterior third of mesoscutum with variable amount of striae/costae, orientation variable, most often longitudinal, but sometimes transverse, or obliquely angled mesad toward posterior margin; scutellum with variable number of longitudinal costae; propodeum with more distinct transverse carinae that extend across the dorsum to upper half of side; wing venation as in Figure 84D.

#### Male.

See Figure 84E–G.

#### Biology.

*Stenamma expolitum* is a specialist inhabitant of clay banks. It occurs from 50–1300 m elevation in mature wet forest environments. Nests are generally found in vertical clay banks along streams or cuts along trails. The biology of *Stenamma expolitum* is reviewed in detail in Longino (2005) and in the overview of natural history section above. Also, see also the biology section for *Stenamma alas* above. These two species have nearly identical behaviors with subtle modifications, which I describe here. *Stenamma expolitum* constructs its nests with a vertical turret, rather than a horizontal one. Each nest usually has two chambers instead of one. The main chamber connects to the turret, but there is often a secondary chamber behind the turret. The queen and brood always occur in the main chamber. Colony size is probably similar to *Stenamma alas*, but so far, censused colonies tend to be smaller, with around 100 individuals. In Costa Rica, I have noticed that *Stenamma expolitum* can be abundant at very low elevations, whereas *Stenamma alas* is more common around 300 m and higher. It also seems to be easier to find colonies of *Stenamma expolitum* away from streams in trail cuts or in steep clay slopes, suggesting that the species may be more tolerant of drier substrates.

#### Comments.

*Stenamma expolitum*, along with *Stenamma alas* and *Stenamma expolitico*, belongs to the *expolitum* species group. This group is defined by the following: propodeal spines absent; petiole and postpetiole almost completely smooth and shiny, with only faint punctae sometimes present laterally; postpetiole in profile bulging, globular, appearing more voluminous than petiolar node; anterior clypeal margin with a median emargination; basal margin of mandible straight. Molecular phylogenetic data firmly show that *Stenamma zelum* is sister to the *expolitum* group (Branstetter 2012), but this species is morphologically divergent and shares none of the *expolitum* group’s diagnostic character states.

*Stenamma expolitum* can be separated from other *expolitum* group species by its nearly completely smooth and shiny face. In the field, *Stenamma expolitum* can be separated from *Stenamma alas* by its nest structure, described above. Although they do not co-occur, *Stenamma expolitum* looks superficially like *Stenamma atribellum*, which is restricted to Cusuco, Honduras. Both species have completely smooth sculpturing, but the latter species has the anterior constriction of the gaster distinctly elongate.

#### Material examined.

**COSTA RICA: *Alajuela***: Casa Eladio, Río Peñas Blancas, 10.3167°N, 84.7167°W, 800m, 2 Feb 1994 (J. Longino); 2km N Volcan Arenal, 10.483°N, 84.700°W, 600m, 3 Apr 2005 (J. Longino); ***Heredia***: La Selva Biol. Sta., 10.4333°N, 84.0167°W, 50m, 8 Aug 2004 (J. Longino); P.N. Braulio Carrillo, 10.3378°N, 84.0500°W, 300m, 19 Sep 2006 (TEAM); 11km ESE la Virgen, 10.35°N, 84.05°W, 300m, 5 Nov 2003 (J. Longino); 10km SE La Virgen, 10.3333°N, 84.0833°W, 500m, 16 Feb 2003 (J. Longino); ***Limón***: Colombiana Farm, [ca. 10.167°N, 83.583°W], Apr 1924 (W. M. Mann); **NICARAGUA: *Jinotega***: PN Cerro Saslaya, 13.76650°N, 85.02485°W, 1040m, 12 May 2011 (LLAMA);***Matagalpa***: RN El Musún, 4.9km NNW Río Blanco, 12.97471°N, 85.23318°W, 1205m, 11 Oct 2008 (M. G. Branstetter).

### Stenamma felixi

Mann

http://species-id.net/wiki/Stenamma_felixi

**Figure 86.**
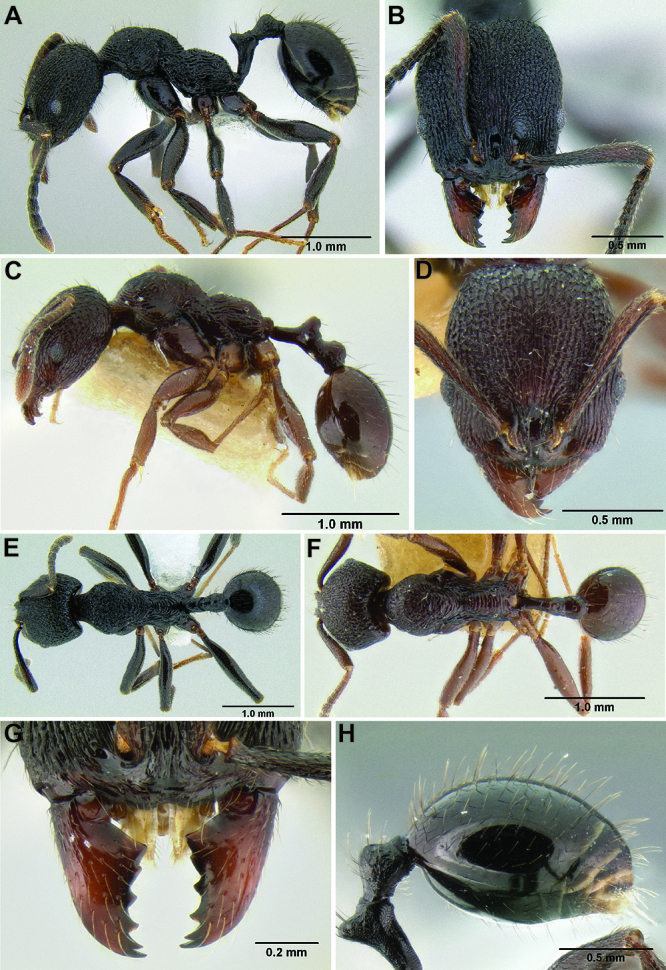
*Stenamma felixi* **A, B, E, G, H** Worker (CASENT0620969) **C, D, F** Lectotype worker (CASENT0126355).

**Figure 87.**
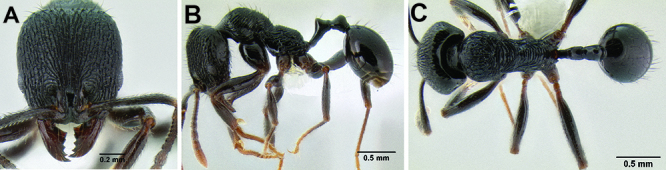
*Stenamma felixi* variant (CASENT0622555) **A** Face **B** Profile **C** Dorsum.

**Figure 88.**
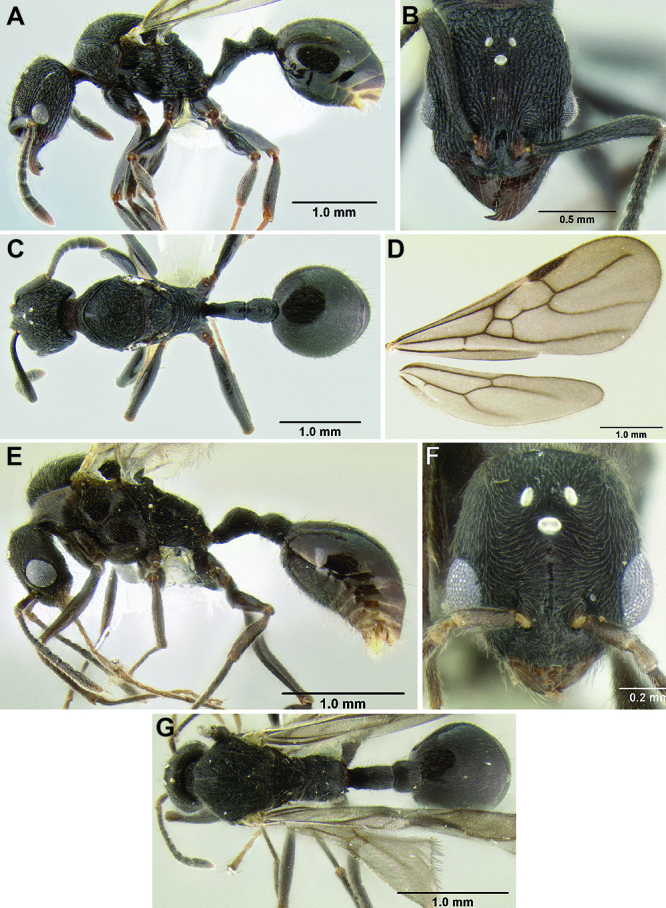
*Stenamma felixi* **A** Queen (CASENT0622308), profile **B** Same, face **C** Same, dorsum **D **Same, wings **E** Male (CASENT0622311), profile **F** Same, face **G** Same, dorsum.

**Figure 89.**
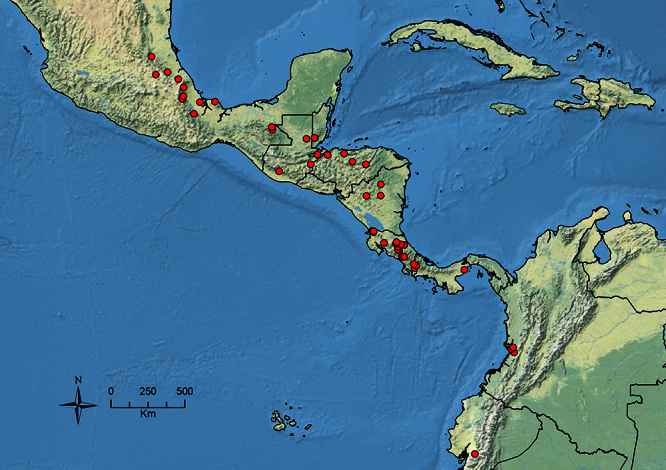
Distribution map of *Stenamma felixi*.

Worker: Figures 86 , 87 ; Queen: Figure 88A–D ; Male: Figure 88E–G ; Map: Figure 89

Stenamma felixi Mann, 1922: 21, fig. 10. Lectotype worker (**here designated**): HONDURAS, [Atlántida]: San Juan Pueblo, [ca. 15.583°N, 87.233°W], Feb–Mar 1920 (W. M. Mann) (USNM, Cotype. No. 24448, pin CASENT0126355, bottom specimen) (USNM) [examined]. Smith, 1962: 34, worker description. Branstetter, 2009: phylogeny. Branstetter, 2012: phylogeny.

#### Worker diagnosis.

Integument mostly black to dark brown; medium- to large-sized species (see HL, ML, PrW below); anterior clypeal margin with a median emargination; basal margin of mandible straight to slightly sinuous, without a basal notch or depression; head and mesosoma usually densely sculptured, with sharp carinae, rugae, or rugoreticulae; eye relatively large (EL 0.16–0.20, REL 18–22), oval-shaped, with 8–11 ommatidia at greatest diameter; propodeal spines absent, propodeum forming shallow, blunt angles where propodeal dorsum and declivity converge (PSL 0.07–0.11, PSI 0.8–1.1); setae on gastral tergites mostly sparse, long, and suberect, only sometimes with a few short decumbent setae; frontal lobes of moderate width (FLD 0.22–0.26, FLI 25–29), not completely obscuring torular lobes in full face view; metafemur relatively long (MFI 75–88). Similar species: *Stenamma manni*, *Stenamma schmidti*.

#### Geographic range.

Mexico (Atlantic slope) to Ecuador.

#### Worker description.

(10 measured) HL 0.90–1.19 (1.00), HW 0.81–1.04 (0.87), FLD 0.22–0.26 (0.22), PCW 0.04–0.09 (0.05), SL 0.69–0.95 (0.81), EL 0.16–0.20 (0.17), ACL 0.70–0.92 (0.79), ML 1.25–1.62 (1.37), PrW 0.58–0.74 (0.62), PSL 0.07–0.11 (0.09), SDL 0.07–0.13 (0.08), PL 0.41–0.52 (0.45), PH 0.25–0.32 (0.29), PW 0.16–0.23 (0.23), PPL 0.24–0.33 (0.28), PPH 0.21–0.27 (0.23), PPW 0.18–0.27 (0.18), MFL 0.93–1.28 (1.08), MTL 0.71–0.98 (0.81), CI 83–90 (87), SI 84–101 (93), REL 18–22 (20), FLI 25–29 (26), PSI 0.8–1.1 (1.1), MFI 75–88 (81), ACI1 57–62 (59), ACI2 93–102 (97).

Medium- to large-sized species; general body color mostly black to dark brown, with appendages lighter, brown to orange-brown toward extremities; setae dark gold-brown; mandible with 5–6 teeth, consisting of 4 distinct apical teeth, a basal tooth, and 1 tooth in between, which is smaller and often effaced; basal margin of mandible straight to slightly sinuous, without a basal notch or depression; mandible mostly smooth, except for scattered piligerous punctae, and some lateral striations; anterior clypeal margin with a shallow median emargination; median lobe of clypeus with at least a pair of distinct longitudinal carinulae that diverge toward anterior margin, sometimes with a few additional faint carinulae, apex of lobe smooth, or with some faint transverse carinulae; remainder of clypeus mostly smooth and shiny; posterior extension of clypeus between frontal lobes of moderate width (PCW 0.04–0.09), sides subparallel to slightly hour-glass-shaped; frontal lobes of moderate width (FLD 0.22–0.26, FLI 25–29), not greatly obscuring torular lobes in full face view; head usually roughly oval-shaped, but some populations with posterior margin distinctly broader than anterior margin, making head appear more triangular (CI 83–90), posterior margin always depressed medially; eye relatively large (EL 0.16–0.20, REL 18–22), oval-shaped, with 8–11 ommatidia at greatest diameter; face densely sculptured, but sculpture type variable, most often with some longitudinal rugae/carinae along midline, transitioning to rugoreticulae toward lateral margins, but sometimes face almost completely rugoreticulate, or completely carinate, depth and sharpness of sculpture variable; scape of moderate length (SI 84–101), just reaching posterior margin of head when laid back; scape surface mostly smooth, but with distinct carinulae, and scattered piligerous punctures; scape sometimes appearing thickened and more robust; flagellum with distinct 4-segmented antennal club; mesosoma mostly densely sculptured, but sculpture type variable; dorsum of pronotum usually rugose (longitudinal orientation) to rugoreticulate, but sometimes carinate, anterior declivity of pronotum with transverse carinulae; dorsum of mesonotum rugoreticulate to transversely carinate, sometimes intermediate; side of pronotum rugulose to carinate; katepisternum mostly smooth, with some rugulae on upper half; dorsum and declivity of propodeum transversely carinate/carinulate; side of propodeum rugose; promesonotum in profile domed (higher than average), symmetrical to slightly asymmetrical, with location of apex variable; metanotal groove distinct, but narrow; dorsum of propodeum in profile distinctly longer than declivity; propodeal spines absent, propodeum forming shallow, blunt angles where dorsum and declivity converge (PSL 0.07–0.11, PSI 0.8–1.1); petiole of moderate length (PL/HW 0.48–0.55); petiolar node of moderate height (PH/PL 0.60–0.64), subconical in shape, usually pointing vertically to only slightly posteriad, dorsum narrowly to somewhat broadly rounded, posterior margin of petiole, where postpetiole inserts, distinctly bent downwards; postpetiole in profile nearly symmetrical, with anterior face slightly longer than posterior face, postpetiole similar in size to petiolar node (PPH/PH 0.78–0.85); petiole and postpetiole usually mostly punctate, with only anterior faces of nodes smooth, but sometimes nodes mostly smooth, with punctae faint; gaster smooth, with scattered piligerous punctae; most of body dorsum with standing pilosity; setae on gastral tergites mostly sparse, long, and suberect, only sometimes with a few short decumbent setae; setae on scapes subdecumbent to appressed; setae on legs decumbent to appressed, with a few suberect setae on femoral venters and coxae.

#### Queen description.

(6 measured) HL 1.00–1.17 (1.07), HW 0.85–0.98 (0.89), FLD 0.25–0.27 (0.25), PCW 0.05–0.08 (0.07), SL 0.76–0.92 (0.84), EL 0.23–0.30 (0.29), ACL 0.69–0.91 (0.83), ML 1.45–1.75 (1.57), PrW 0.79–0.91 (0.83), PSL 0.11–0.15 (0.13), SDL 0.12–0.16 (0.14), PL 0.51–0.59 (0.55), PH 0.31–0.36 (0.33), PW 0.21–0.25 (0.23), PPL 0.30–0.37 (0.35), PPH 0.26–0.31 (0.29), PPW 0.26–0.32 (0.31), MFL 0.96–1.27 (1.11), MTL 0.74–0.95 (0.85), CI 83–88 (83), SI 87–97 (94), REL 27–32 (32), FLI 27–30 (28), PSI 0.9–1.0 (0.9), MFI 76–88 (81), ACI1 58–61 (59), ACI2 90–99 (99).

Same as worker except for standard queen modifications and as follows: pronotum transversely carinulate; mesoscutum longitudinally carinulate, or carinate; scutellum rugoreticulate, or longitudinally carinulate to carinate; propodeum with transverse carinulae/carinae that wrap around surface; mesopleuron usually mostly smooth; pilosity on gastral tergites clearly bilayered, with a sparse layer of long, suberect setae, and a dense layer of appressed pubescence; wing venation in Figure 88D.

#### Male.

See Figure 88E–G.

#### Biology.

*Stenamma felixi* is one of the most widespread and common species of MAC *Stenamma*. It occurs from approximately 50–1600 m, but is most common above 500 m, and is always found in wet forest environments, ranging from lowland rainforest to cloud forest. Workers have been collected by sifting leaf litter, beating and sweeping vegetation, baiting, using pitfall and Malaise traps, and by general searching. Nests are generally quite large and have been found in rotting logs on the ground, in tree stumps, under bark of logs, and rarely in mud banks. A few lone foundresses have been found under rotting epiphyte clumps in old treefalls, and some workers have been collected from orchids at quarantine in the U.S. All colonies collected so far have been monogynous. Workers seem to be epigeic, solitary foragers, but nothing is known about dietary preference. A very common experience is to find lone, stray workers running across medium- to large-sized logs in forest.

#### Comments.

This species is rather distinctive with its large size, lack of propodeal spines, and dense sculpturing. It should not be easily confused with any other MAC species.

Over its range, *Stenamma felixi* shows considerable variation in the density and orientation of its sculpturing as well as in petiole shape. However, I have only identified one distinct variant (Figure 87) worth describing in more detail. Specimens from Nicaragua and Costa Rica have very deep carinate sculpturing on the face and mesosoma. The facial carinae are usually longitudinal, but occasionally reticulate laterad. The pronotal dorsum has longitudinal carinae, but the side of the pronotum has arcuate carinae that wrap across the dorsum of the metanotum. The head in profile view has a very distinctive shape, in which the posterior margin of the head is very wide compared to the anterior margin, giving the head a somewhat triangular appearance. Lastly, the petiolar and postpetiolar nodes are noticeably smooth. Specimens from Colombia and Ecuador and from north of Nicaragua lose the carinate sculpture. I treat all of the slight sculpture differences among popluations as intraspecific variation as I have found no evidence of sympatry among forms.

#### Material examined. 

**BELIZE: *Cayo***: Chiquibul N.P., Doyle’s Delight, 16.48972°N, 89.04583°W, 950m, 20–27 Aug 2007 (P. W. Kovarik); Chiquibul N.P., Doyle’s Delight, 16.4833°N, 89.0333°W, 1000m, 19–22 Aug 2007 (P. W. Kovarik); Chiquibul N.P., Doyle’s Delight, 16.49305°N, 89.04694°W, 1100m, 19–28 Aug 2007 (P. W. Kovarik); **COLOMBIA: *Valle del Cauca***: Buenaventura, Bajo Calima, [ca. 3.996°N, 76.974°W], 30m, 16–17 Mar 1967 (Root & Brown); Buenaventura, 3.2km above Río Aguaclara on old rd to Cali, [ca. 3.693°N, 76.925°W], 17–19 Jun 1971 (W. L. Brown); Reserva Forestal Escalerete, [ca. 3.886°N, 77.069°W], 80m, 29 May 2007 (Usma-Aldana); **COSTA RICA: *Cartago***: La Carpintera, [ca. 9.884°N, 83.983°W], 1500m, Apr 1924 (W. M. Mann); Navarro Farm, [ca. 9.8167°N, 83.8833°W,], 1100m, Mar 1924 (W. M. Mann); ***Guanacaste***: Est. Mengo, SW side Volcan Cacao, [ca. 10.933°N, 85.450°W], 1100m, Feb 1989 (GNP Biodiversity Survey); Est. Pitilla, 9km S Sta. Cecilia, [ca. 10.983°N, 85. 433°W], 700m, Jan 1991 (Curso Microhymenoptera); ***Heredia***: La Selva Biol. St., [ca 10.430°N, 84.007°W], 100m, 16 Jan 1979 (S. P. Cover); 11km ESE La Virgen, 10.35°N, 84.05°W, 300m, 15 Feb 2004 (ALAS); 10km NE Vara Blanca, 10.2333°N, 84.0833°W, 1500m, 12 Feb 2005 (ALAS); 16km SSE La Virgen, 10.2667°N, 84.0833°W, 1100m, 14–17 Mar 2001 (ALAS); ***Limón***: Colombiana Farm, [ca. 10.167°N, 83.583°W], Apr 1924 (W. M. Mann); Río Toro Amarillo, vic. Guapiles, [ca. 10.205°N, 83.789°W], 300m, 25 Feb–9 Mar 1966 (W. L. Brown); ***Puntarenas***: Altamira Biological Station, 9.02922°N, 83.00813°W, 1400m, 30 May 2007 (M. G. Branstetter); 2km W Las Alturas, 8.933°N, 82.850°W, 1260m, 23 Mar 1990 (P. S. Ward); Las Cruces Biological Station, 8.78658°N, 82.95987°W, 1150m, 29 May 2007 (M. G. Branstetter); Monteverde, 0.25km E Lecheria, [ca. 10.307°N, 84.810°W], 1300m, 19 May 1988 (Cover et al.); ***San José***: Bajo La Hondura, Braulio Carillo Nat. Park, [ca. 10.067°N, 83.983°W], 20 Jun 1926 (F. Nevermann); 1km N La Ese, 9.450°N, 83.717°W, 1400m, 5 Aug 1985 (P. S. Ward); Pan-Am Hwy, 23 rd km N San Isidro de General, [ca. 9.466°N, 83.703°W], 1600m, 20–23 Jun 1974 (Harding & Donahue); 2km E San Gerardo, 9.467°N, 83.583°W, 1600m, 4 Aug 1985 (P. S. Ward); **ECUADOR: *Cañar***: 2–6km above Cochancay on Guayaquil-Tambo H’way, [ca. 2.468°S, 79.291°W], 600m, 25 Jul 1973 (W. L. Brown); **GUATEMALA: *Izabal***: 5km NW Morales, 15.51351°N, 88.86647°W, 240m, 18 May 2009 (J. Longino);***Petén***: 13km NW Machaquilá, 16.44202°N, 89.53495°W, 390m, 28 May 2009 (LLAMA);***Zacapa***: 2km SE La Unión, 14.94701°N, 89.27594°W, 1550m, 12 May 2009 (LLAMA); ***Suchitepéquez***: 5.5km S Vol. Atitlán, 14.52857°N, 91.19569°W, 1070m, 18 Jun 2009 (LLAMA); **HONDURAS: *Atlántida***: San Juan Pueblo, [ca. 15.583°N, 87.233°W], 100m, Feb–Mar 1920 (W. M. Mann); ***Cortés***: PN Cusuco, 15.48940°N, 88.23732°W, 1290m, 30 May 2010 (LLAMA); ***Olancho***: 9km N Catacamas, 14.93646°N, 85.90488°W, 1340m, 10 May 2010 (M. G. Branstetter); 10km N Catacamas, 14.94125°N, 85.90385°W, 1320m, 10 May 2010 (LLAMA); 11km NNE Catacamas, 14.95031°N, 85.86229°W, 1470m, 12 May 2009 (J. Longino); PN La Muralla, 15.09721°N, 86.73840°W, 1480m, 5 May 2010 (LLAMA); **MÉXICO: *Chiapas***: Lago Metzabok, 17.12360°N, 91.63766°W, 575m, 5 Jun 2008 (LLAMA); Nahá, 16.94885°N, 91.59489°W, 930m, 8 Jun 2008 (LLAMA);***Hidalgo***: 6.4km SW Chapulhuacán, [ca. 21.155°N, 98.931°W], 1070m, 27 Jun–1 Jul 1973 (A. F. Newton); between Real del Monte and El Chico, [ca. 20.138°N, 98.673°W], Mar–Aug 1913 (W. M. Mann); ***Oaxaca***: Mirador Grande, 17.89844°N, 96.36253°W, 990m, 14 Aug 2009 (M. G. Branstetter);***Puebla***: 17km NE Teziutlán, [ca. 19.877°N, 97.310°W], 1940m, 7 Jun 1988 (W. P. MacKay); 24km N Xicotepec de Juarez, [ca. 20.282°N, 97.963°W], 1070m, 17 Jun 1983 (R. S. Anderson); ***Veracruz***: Canyon Río Metlac, near Fortín, [ca. 18.90°N, 97.00°W], 975m, 7–28 Aug 1973 (A. F. Newton); 10km S Orizaba, 18.750°N, 97.083°W, 1500m, 19 Mar 1985 (P. S. Ward); 2.7km N Teocelo, [ca. 19.40°N, 96.98°W], 1130m, 22–24 Jul 1973 (A. F. Newton); 11km N San Andrés Tuxtla, 18.55°N, 96.00°W, 1400m, 23 Mary 1985 (P. S. Ward); 10km NNW Sontecomapan, 18.583°N, 95.083°W, 200m, 20 Mar 1985 (P. S. Ward); **NICARAGUA: *Jinotega***: PN Cerro Saslaya, 13.77173°N, 85.01286°W, 1110m, 12 May 2011 (LLAMA);RNDatanlí El Diablo, 13.10410°N, 85.01286°W, 1110m, 12 May 2011 (LLAMA);RNDatanlí El Diablo, 13.08051°N, 85.87462°W, 1170m, 20 May 2011 (LLAMA); **PANAMA: *Panama***: Cerro Campana, [ca. 8.678°N, 79.928°W], 875m, 17 Jan 1960 (Fairchild & Brown).

### Stenamma hojarasca sp. n.

urn:lsid:zoobank.org:act:E2855151-38C5-41C4-869E-8879710A8F45

http://species-id.net/wiki/Stenamma_hojarasca

**Figure 90.**
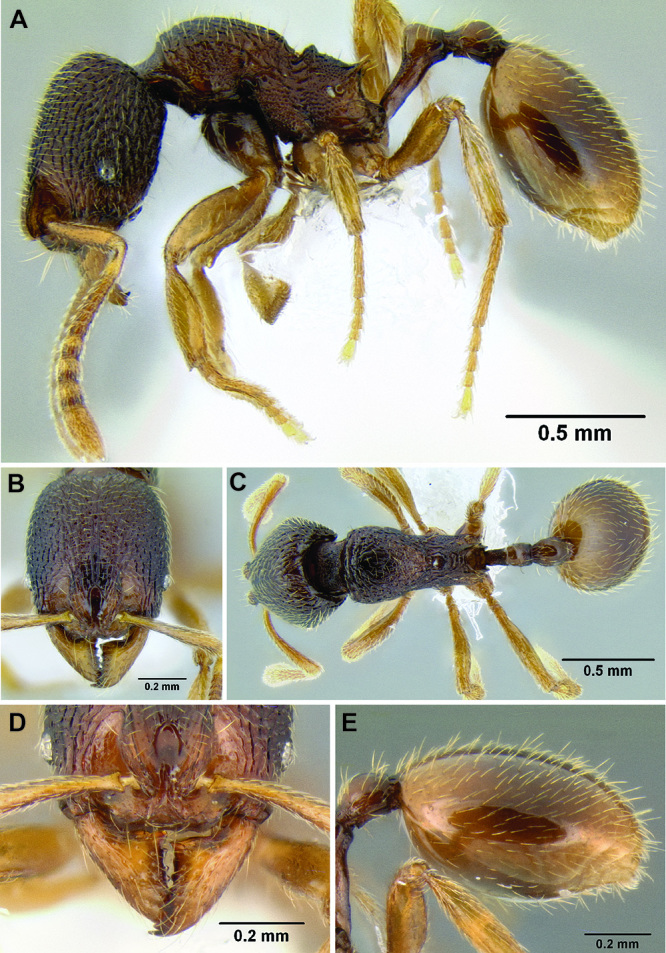
*Stenamma hojarasca* holotype worker (CASENT0622132) **A** Profile **B** Face **C** Dorsum **D **Anterior clypeal margin in anterodorsal view **E** Gaster.

**Figure 91.**
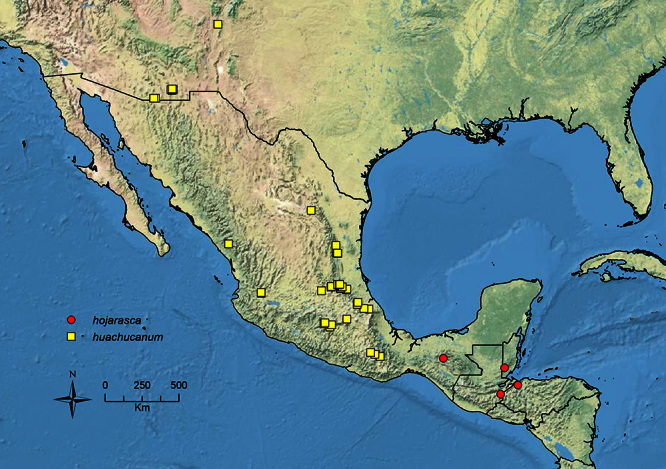
Distribution map of *Stenamma hojarasca* (circles) and *Stenamma huachucanum* (squares).

Worker: Figure 90 ; Map: Figure 91

#### Type material.

*Holotype worker*.HONDURAS: Cortés, Parque Nacional Cusuco, 15.48939°N, 88.23678°W ±300m, 1280m, 31 May 2010, mesophyll forest, ex sifted leaf litter (LLAMA, collection Wm-C-06-1-03) [USNM, specimen CASENT0622132]. *Paratypes*: same data as holotype but 15.48896°N, 88.23439°W ±20m, 1290m, 30 May 2010 (LLAMA, Wm-C-06-1-01) [1w, CAS, CASENT0622124]; 15.48683°N, 88.23422°W ±300m, 1340m, 30 May 2010 (LLAMA, Wm-C-06-1-02) [1w, EAPZ, CASENT0622126]; 15.48723°N, 88.23482°W ±20m, 1330m, 30 May 2010 (LLAMA, Wa-C-06-1-08) [1w, INBio, CASENT0621692]; 15.48717°N, 88.23476°W ±20m, 1330m, 30 May 2010 (LLAMA, Wa-C-06-1-10) [1w, MCZ, CASENT0621693]; 15.48940°N, 88.23695°W ±20m, 1290m, 30 May 2010 (LLAMA, Wa-C-06-2-28) [1w, UCD, CASENT0621752]; 15.48839°N, 88.23592°W ±60m, 1260m, 31 May 2010 (LLAMA, Wm-C-06-2-02) [1w, MGBPC, CASENT0622169].

#### Worker diagnosis.

Integument mostly dark red-brown to orange-brown; small- to medium-sized species (see HL, ML, and PrW below); anterior clypeal margin undulating, with 4 blunt teeth; basal margin of mandible straight to slightly sinuous, without a basal notch or deep depression; gastral pilosity clearly bilayered, with a layer of suberect setae, and a layer of decumbent setae; petiole distinctly elongate (PL/HW 0.60–0.68); postpetiolar node dorsoventrally flattened and slightly elongate (PPH/PH 0.75–0.84); eye relatively small (EL 0.07–0.11, REL 13–18), subcircular, with 4–5 ommatidia at greatest diameter; median clypeal lobe projecting dorsally outward, resulting in rather distinct dorsal and anterior surfaces (visible in profile); dorsal surface of median lobe with a pair of distinct longitudinal carinulae that strongly diverge around median lobe at anterior margin; frontal lobes slightly to very strongly expanded (FLD 0.19–0.25, FLI 31–46), either completely or mostly covering torular lobes in full-face view; propodeal spines present, short to moderate length (PSL 0.12–0.17, PSI 1.6–1.9). *Similar species*: *Stenamma catracho*, *Stenamma cusuco*, *Stenamma ochrocnemis*.

#### Geographic range.

Southern Mexico to Honduras.

#### Worker description.

(7 measured) HL 0.65–0.76 (0.70), HW 0.54–0.66 (0.60), FLD 0.19–0.25 (0.24), PCW 0.03 (0.03), SL 0.47–0.56 (0.49), EL 0.07–0.11 (0.11), ACL 0.46–0.56 (0.50), ML 0.81–0.99 (0.87), PrW 0.40–0.48 (0.42), PSL 0.12–0.17 (0.14), SDL 0.07–0.10 (0.08), PL 0.35–0.42 (0.36), PH 0.16–0.19 (0.17), PW 0.11–0.14 (0.13), PPL 0.18–0.20 (0.19), PPH 0.12–0.15 (0.14), PPW 0.14–0.17 (0.16), MFL 0.52–0.64 (0.55), MTL 0.42–0.54 (0.46), CI 82–88 (86), SI 82–89 (82), REL 13–18 (18), FLI 31–46 (41), PSI 1.6–1.9 (1.9), MFI 102–109 (108), ACI1 65–70 (67), ACI2 96–102 (102).

Small- to medium-sized species; general body color dark red-brown to orange-brown, with patches of lighter brown on gaster; appendages orange-brown to yellow-brown; setae pale golden brown; mandible with 5–7 teeth, consisting of 3 distinct apical teeth, a distinct basal tooth, and 1–3 worn or denticulate inner teeth; basal margin of mandible straight to slightly sinuous, without a basal notch or deep depression; mandible mostly smooth and shiny, with scattered piligerous punctae and faint striae; median clypeal lobe projecting dorsally outward, resulting in rather distinct dorsal and anterior surfaces (visible in profile); anterior clypeal margin undulating, with 4 blunt teeth (best viewed at an anterodorsal angle); dorsal surface of median lobe with a pair of distinct longitudinal carinulae that strongly diverge around median lobe at anterior margin; remainder of clypeus mostly smooth and shiny; posterior extension of clypeus between antennal insertions narrow (PCW 0.03), with sides subparallel to hourglass-shaped; frontal lobes slightly to very strongly expanded (FLD 0.19–0.25, FLI 31–46), either completely or mostly covering torular lobes in full-face view; frontal carinae weakly developed, not extending beyond frontal lobes; head subrectangular to somewhat oval-shaped (CI 82–88), posterior margin slightly depressed medially; eye appearing small (EL 0.07–0.11, REL 13–18), subcircular, with 4–5 ommatidia at greatest diameter; head strongly rugoreticulate, with a few longitudinal costae along midline, interstices faintly punctate; scape somewhat short (SI 82–89), not reaching posterior margin of head when laid back; scape surface mostly smooth and shiny, with scattered piligerous punctae; flagellum with a somewhat distinct 4-segmented antennal club; mesosoma completely sculptured, except for propodeal declivity, which has faint transverse carinulae; promesonotal dorsum rugose to rugoreticulate and punctate, with rugae transverse near anterior margin, becoming arcuate, and then longitudinal posteriad; mesosomal side mostly punctate, with scattered rugulae; anterodorsal margin of promesonotum with a somewhat distinct straight to lightly curving transverse carina, anterolateral margins of pronotum forming distinct shoulders in dorsal view; anterior declivity of pronotum in profile nearly vertical, and forming a sharp transition with the dorsum; promesonotum in profile low-domed, somewhat asymmetrical; metanotal grove distinct, deeper than average; anterodorsal margin of propodeum with a distinct welt; propodeal spines present, short to moderate length (PSL 0.12–0.17, PSI 1.6–1.9); petiole distinctly elongate and gracile (PL/HW 0.60–0.68); petiolar node relatively small (PH/PL 0.45–0.49), subconical and roughly symmetrical in profile, with a rounded dorsum; postpetiolar node dorsoventrally flattened and slightly elongate (PPH/PH 0.75–0.84); petiolar and postpetiolar nodes almost completely smooth and shining, remaining surfaces of waist segments mostly punctate, with a few rugulae surrounding postpetiolar node; gaster mostly smooth and shiny, with scattered piligerous punctae; most of body dorsum with short standing pilosity; gastral pilosity clearly bilayered, with a layer of suberect setae, and a layer of decumbent setae; scape setae decumbent to appressed; setae on legs mostly decumbent to appressed, with suberect setae on coxae and femoral venters.

#### Queen.

Unknown.

#### Male.

Unknown.

#### Biology.

*Stenamma hojarasca* is a rare species known almost exclusively from sifted leaf litter collected from the forest floor. The only exception is a single specimen from a carrion baited pitfall trap in Belize. This species is found in montane mesophyll and cloud forest habitats and has been collected from 1100–1550 m elevation.

#### Comments.

The form of the waist segments combined with the projecting median clypeal lobe, make *Stenamma hojarasca* a very distinctive species. It should not be confused easily with any other MAC species.

The most significant form of variation within this species is the extent to which the frontal lobes are expanded. In the Cusuco population, the frontal lobes are greatly expanded laterally and anteroposteriorly, completely covering the torular lobes in full-face view. In the other populations, the frontal lobes are only moderately expanded and they do not completely cover the torular lobes in full-face view, leaving the outer margins somewhat visible. I treat this as intraspecific variation until more material can be gathered.

Molecular phylogenetic data infer that *Stenamma hojarasca* belongs to a clade that includes *Stenamma cusuco* and *Stenamma ochrocnemis*, with the latter being its sister species (Branstetter unpublished data).

#### Material examined.

**BELIZE: *Cayo***: Chiquibul N.P., Doyle’s Delight, 16.49305°N, 89.04694°W, 1100m, 19–28 Aug 2007 (P. W. Kovarik); **GUATEMALA:** ***Zacapa***: 2km SE La Unión, 14.94460°N, 89.27726°W, 1550m, 12 May 2009 (LLAMA); **HONDURAS:** ***Cortés***: PN Cusuco, 15.48939°N, 88.23678°W, 1280m, 31 May 2010 (LLAMA); PN Cusuco, 15.48683°N, 88.23422°W, 1340m, 30 May 2010 (LLAMA); **MÉXICO: *Chiapas***: 10.6km W El Bosque, 17.043°N, 92.762°W, 1460m, 25–29 Aug 1973 (A. F. Newton).

### Stenamma huachucanum

Smith

urn:lsid:zoobank.org:act:80F4B353-D9ED-4F12-96B6-65BF6075BAEB

http://species-id.net/wiki/Stenamma_huachucanum

**Figure 92.**
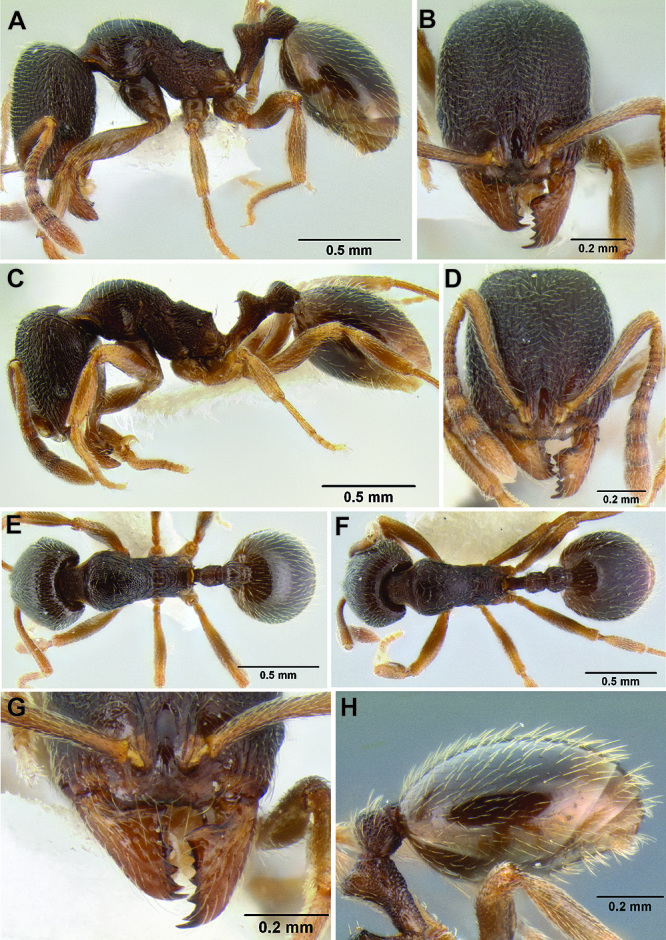
*Stenamma huachucanum* **A, B, E, G, H** Worker (CASENT0126556) **C, D, F** Paratype worker (CASENT010566).

**Figure 93.**
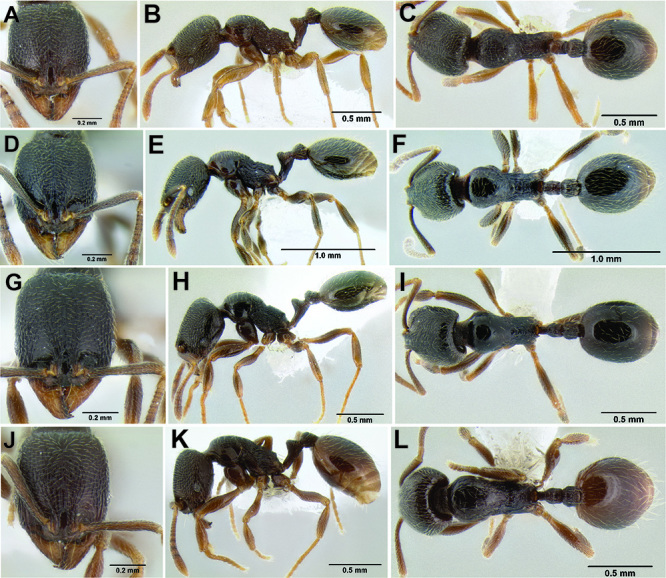
*Stenamma huachucanum* worker variants 1. Face, profile, and dorsal views **A–C** Variant 1 (CASENT0600124) **D–F** Variant 2 (CASENT0605749) **G–I** Variant 3 (CASENT0605412) **J–L** Variant 4 (CASENT0604596).

**Figure 94.**
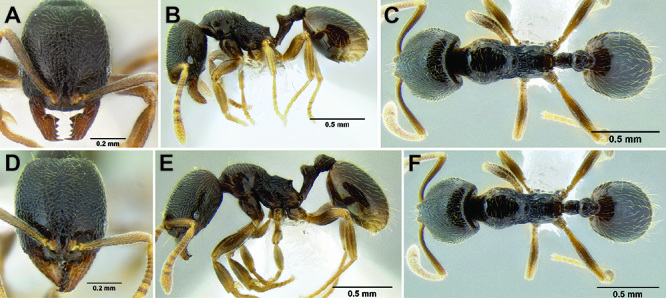
*Stenamma huachucanum* worker variants 2. Face, profile, and dorsal views **A–C** Variant 5 (CASENT0605616) **D–F** Variant 6 (CASENT0605647).

**Figure 95.**
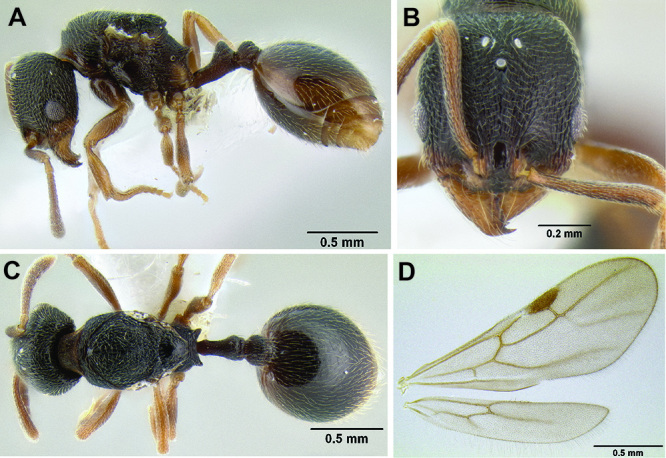
*Stenamma huachucanum* **A** Queen (CASENT0600094), profile **B** Same, face **C** Same, dorsum **D** Same, wings.

Worker: Figures 92- 94 ; Queen: Figure 95 ; Map: Figure 91

Stenamma huachucanum Smith, M. R. 1957: 153, pl 2, fig 8. Holotype worker: USA, Arizona, [Cochise Co.]: Head of Carr Canyon, Huachuca Mts., [ca. 31.432°N, 110.284°W], 8000 ft. [2440 m], 24 July 1950 (W. S. Creighton) (USNM) [examined]. Snelling, 1973: 34, figs 44, 45, 61, notes on worker. Branstetter, 2009: worker images. Branstetter, 2012: phylogeny, worker images.
Stenamma mgb38 [variant 5 below] Branstetter, 2012: phylogeny.

#### Worker diagnosis.

Note this species is variable and difficult to characterize globally. See comments section below, discussing population variants. Integument mostly dark brown to brown; small-sized species (see HL, ML, PrW below); basal margin of mandible sinuous, with a distinct basal depression, but no tooth; anterior clypeal margin undulating, forming 2–4 blunt teeth; eye of moderate size (EL 0.07–0.12, REL 14–21), oval-shaped, with 4–8 (usually 5–6) ommatidia at greatest diameter; propodeal spines tuberculate to short (PSL 0.06–0.11, PSI 1.0–1.4); face usually completely sculptured, with carinulae, rugoreticulae and punctae, but some populations with posterior ¼ or less of head smooth and shiny; pronotal sculpture variable, often with some carinulae and punctae, but some populations completely smooth; remainder of mesosoma sculptured, with punctae, carinulae and/or rugulae; propodeal lobe in profile usually isolated from propodeal spine and with angulate corners, but some populations with lobe appearing broadly rounded and forming a smooth connection with propodeal spine; geography is useful in species determination. *Similar species*: *Stenamma connectum*, *Stenamma crypticum*.

#### Geographic range.

Southwestern U.S.A. to southern Mexico (Oaxaca).

**Worker description.** (28 measured; paratype CASENT0105666 in parentheses) HL 0.52–0.72 (0.72), HW 0.46–0.64 (0.62), FLD 0.11–0.17 (0.17), PCW 0.03–0.05 (0.03), SL 0.43–0.55 (0.55), EL 0.07–0.12 (0.10), ACL 0.43–0.52 (0.52), ML 0.66–0.85 (0.85), PrW 0.33–0.41 (0.41), PSL 0.06–0.11 (0.09), SDL 0.05–0.09 (0.08), PL 0.23–0.35 (0.32), PH 0.15–0.21 (0.21), PW 0.11–0.16 (0.15), PPL 0.13–0.20 (0.17), PPH 0.14–0.19 (0.18), PPW 0.15–0.20 (0.19), MFL 0.44–0.59 (0.59), MTL 0.37–0.48 (0.48), CI 83–91 (86), SI 81–94 (89), REL 14–21 (15), FLI 23–29 (27), PSI 1.0–1.4 (1.1), MFI 101–124 (105), ACI1 67–71 (68), ACI2 92–103 (95).

Small-sized species; general body dark brown to brown (type population), with appendages brown or orange-brown to yellow-brown, usually lighter at joints and toward extremities; setae golden brown; mandible with 6–7 teeth, 2–3 teeth near basal tooth sometimes worn and indistinct; basal margin of mandible sinuous, with a distinct basal depression, but no tooth; mandible mostly smooth and shiny, with some scattered piligerous punctae, and some striations near base and on lateral surface; anterior clypeal margin undulating, forming 2–4 sharp to blunt teeth (4 sharp teeth in type population); median lobe of clypeus with a pair of longitudinal carinulae that diverge toward anterior margin, apex with a short transverse carinula, area in between median lobe and anterior clypeal margin forming a shallow concavity; remaining surface of clypeus mostly smooth; posterior extension of clypeus between antennal insertions somewhat narrow to moderate width (PCW 0.03–0.05), with sides subparallel; frontal lobes of moderate width (FLD 0.11–0.17, FLI 23–29), not greatly obscuring torular lobes in full-face view; head subrectangular to roughly oval-shaped (CI 83–92), posterior margin slightly depressed medially; eye of moderate size (EL 0.07–0.12, REL 14–21), oval-shaped, with 4–8 (usually 5–6) ommatidia at greatest diameter; face sculpture variable, usually completely sculptured, with light rugoreticulae, longitudinal carinulae, and/or punctae, but sometimes sculpture reduced, with posterior 1/4 or less of head becoming smooth and shiny; scape of short to moderate length (SI 81–94), usually not quite reaching posterior margin of head when laid back; scape surface mostly smooth to somewhat rough (type population), with variable density of piligerous punctae; flagellum with a distinct 4-segmented antennal club; pronotal sculpture highly variable, often with some longitudinal carinulae/rugulae and faint punctae (type population), but some populations completely smooth; remainder of mesosoma completely sculptured with punctae and a variable amount of rugulae/carinulae; promesonotum usually low-domed, and asymmetrical, with the anterior face longer and steeper than posterior face (type population), but some populations with promesonotum distinctly domed, and roughly symmetrical; metantoal groove present and distinct, of average width and depth; propodeal lobe in profile usually isolated from propodeal spine and with angulate corners, but some populations with lobe appearing broadly rounded and forming a smooth connection with propodeal spine; propodeal spines tuberculate to short (PSL 0.06–0.11, PSI 1.0–1.4); petiole length and shape variable, often short and stocky, with a somewhat large node that points vertically, and a sinuous venter (type population), but sometimes more elongate, with node smaller and pointing distinctly posteriad, or sometimes anteroposteriorly compressed, making it very narrow (PL/HW 0.46–0.59); postpetiole usually forming a small node, similar in size or smaller than petiolar node (type population) (PPH/PL 0.79–0.97); petiole and postpetiole usually mostly punctate, with only anterior faces of nodes smooth (type population), but some populations with punctae reduced and nodes mostly smooth; gaster usually completely smooth, but some populations with first sternite and tergite lightly to strongly punctate (variable in type population); most of body dorsum with relatively short standing pilosity; gastral pilosity distinctly to indistinctly bilayered, with a layer of longer suberect to subdecumbent setae, and a layer of decumbent setae, density of setae variable, usually relatively dense (type population); setae on scape decumbent to appressed; setae on legs decumbent to appressed with longer setae on femoral venters and coxae.

#### Queen description.

(8 measured) HL 0.55–0.71 (0.68), HW 0.50–0.65 (0.58) FLD 0.13–0.18 (0.16), PCW 0.03–0.05 (0.04), SL 0.43–0.56 (0.49), EL 0.15–0.18 (0.15), ACL 0.42–0.52 (0.49), ML 0.78–0.98 (0.85), PrW 0.43–0.59 (0.44), PSL 0.09–0.16 (0.12), SDL 0.06–0.10 (0.09), PL 0.29–0.38 (0.34), PH 0.16–0.23 (0.22), PW 0.13–0.18 (0.16), PPL 0.13–0.20 (0.18), PPH 0.15–0.21 (0.20), PPW 0.16–0.23 (0.21), MFL 0.46–0.60 (0.51), MTL 0.41–0.52 (0.45), CI 86–94 (86), SI 82–87 (85), REL 25–31 (25), FLI 23–28 (27), PSI 1.3–1.8 (1.3), MFI 105–114 (113), ACI1 66–71 (68), ACI2 92–100 (99).

Same as worker except for standard queen modifications and as follows (comparison with queens from near type locality only): pronotum with transverse carinulae/rugulae; mesoscutum longitudinally carinulate, with a small patch of smooth cuticle anteromesad; scutellum smooth along midline, with longitudinal rugulae mesad; anepisternum partly smooth, remainder carinulate; katepisternum mostly smooth; propodeum with transverse carinulae that wrap around propodeum; propodeal spines longer than worker; gastral pilosity denser; wing venation as in Figure 95D.

#### Male.

Unknown.

#### Biology.

*Stenamma huachucanum* is a cryptic leaf litter ant known mostly from Winkler or Berlese samples of sifted leaf litter. As defined here, the species is widely distributed, occupying both relatively dry, seasonal habitats (e.g. tropical deciduous forest with juniper, oak-pine-juniper woodland, oak woodland, oak-pine-douglas fir forest) and tropical wet forest habitats (e.g. mesophyll forest, cloud forest, oak-pine forest). Collections have been made from 1000–2900 m, but the species is most common between 1600–2500 m. In seasonal habitats, workers have been found underneath rocks, in addition to the leaf litter.

#### Comments.

*Stenamma huachucanum* forms a difficult complex composed of many divergent allopatric populations. The complex probably includes several good biological species, but I have chosen to lump most forms into a single entity, because there is no clear evidence of sympatry among forms, and some specimens appear to have intermediate phenotypes that connect distinct forms. The exceptions to this lumping approach are the similar species *Stenamma connectum* and *Stenamma crypticum*. These species are morphologically similar to *Stenamma huachucanum*, but molecular phylogenetic results provide strong evidence that they represent separate lineages (Branstetter unpublished data).

There are some key morphological differences separating populations of *Stenamma huachucanum* from *Stenamma connectum* and *Stenamma crypticum*, but considering all of the variation among populations, the easiest way to identify species is with geography. *Stenamma huachucanum* occurs from the southwestern U.S.A to Oaxaca, where it is found only in the drier, interior of the state. In eastern Mexico, the species is found from Tamaulipas to Puebla, with no records from Veracruz. *Stenamma connectum* is found in Veracruz, Mexico and on the wetter, Caribbean slope of Oaxaca. *Stenamma crypticum* occurs mainly from Chiapas, Mexico to Nicaragua. However, as noted above under both *Stenamma crypticum* and *Stenamma connectum*, a few putative *Stenamma crypticum* specimens are known from one sample taken in Veracruz, nearly in sympatry with *Stenamma connectum*. If confirmed, this latter case is the only evidence of sympatry among any of these similar-looking species.

Within the *Stenamma huachucanum* complex there is considerable variation among populations. To help in the identification of *Stenamma huachucanum*, and to aid future taxonomic efforts, I describe several *Stenamma huachucanum* variants below.One observation about variation within the complex is that specimens from western and central Mexico, where it is drier, tend to look more like the type population. Specimens from the eastern slope, where it is wetter, become smoother and more aberrant, in general. The most problematic areas are in central Mexico where it transitions from wet to dry. At these localities I find specimens with intermediate features. This is one of the main reasons I have lumped what seem like very different populations together.

The main features of the type form of *Stenamma huachucanum* are indicated in the species description above (see parenthetical comments). The key characters for the type form are sculpturing and the shape of the petiole. The head is completely sculptured and mostly rugoreticulate-punctate, with some longitudinal carinulae along the midline. The pronotum is lightly carinulate-punctate (longitudinal orientation), with small smooth patches on the dorsum. One characteristic unseen by Smith (1957) or Snelling (1973) is that some specimens of *Stenamma huachucanum* from the southwestern U.S.A. have the first gastral tergite and sternite punctate. I treat this as intraspecific variation as no other characters within these specimens vary significantly. The petiole of the type form has a distinctive shape, shared by several of the variants. It is rather short in length, but with a tall vertically projecting node that is roughly symmetrical in profile. Also, the venter of the petiole usually has a distinct sinuosity that often includes a small anteroventral process. A peculiarity I have noticed among worker specimens from Arizona is that they vary significantly in size, with some appearing to have allometrically enlarged heads.

Variant 1 (Figure 93A–C) was collected in Sinaloa, Mexico. It is very similar to the type form except as follows: promesonotum more domed; pronotal dorsum strongly carinulate, without punctae.

Variant 2 (Figure 93D–F) was collected from Volcán de Tequila in Jalisco, Mexico. It is similar to the type form except as follows: pronotum mostly smooth; petiolar node in profile broader; petiolar venter in profile straight, not sinuous.

Variant 3 (Figure 93G–I) is from the drier portion of Oaxaca (Asunción Nochixtlan). It varies from the type form as follows: promesonotum distinctly domed and mostly smooth; petiolar node broader, more robust; face sculpture reduced, with posterior margin becoming smooth, and rugoreticulae less visible. Similar-looking specimens also occur in Mexico state (Temascaltepec).

Variant 4 (Figure 93J–L) is from another site in Oaxaca (10.6km N Jct 190/135). It varies from the type as follows: promesonotum in profile low-domed and appearing very long, with the anterior face sloping gently into the metanotal groove; pronotum with many longitudinal carinulae around humeri, and a few on dorsum; gastral pilosity longer.

Variant 5 (Figure 94A–C) is a wet forest version of *Stenamma huachucanum*. It occurs at several sites on the eastern slope of the mountains in Querétaro. There are similar looking specimens from San Luis Potosí and Hidalgo states. It differs from the type population as follows: overal body size smaller; face sculpture reduced, with posterior ¼ or less of head smooth, remaining sculpture consisting of fine punctae, carinulae, and rugoreticulae; pronotum and dorsum of mesonotum smooth; promesonotum in profile variable, sometimes more flat, with posterior face merging smoothly into metanotum, sometimes more distinctly domed; petiolar node in profile narrow, appearing anteroposteriorly compressed. The specimens with a more domed promesonotum appear intermediate with variant 6.

Variant 6 (Figure 94D–F) is another wet forest version of *Stenamma huachucanum*. It occurs throughout the El Cielo reserve in Tamaulipas, Mexico, and differs from variant 5 as follows: head more robust, slightly broader, with posterior margin distinctly depressed medially; promesonotum domed, only slightly asymmetrical; propodeal spines forming well-developed, blunt tubercles; outline of propodeum in profile sinuous, with propodeal spine and lobe smoothly connected; petiole appearing elongate; petiolar node small, with a rounded dorsum that points distinctly posteriad.

#### Material examined.

**MÉXICO: *Coahuila***: H’way 57, pass 25km E Saltillo, [ca. 25.436°N, 100.806°W], 11 Aug 1965 (Cornell Univ. Mexico Field Party); ***Hidalgo***: 6.4km SW Chapulhuacán, [ca. 21.155°N, 98.931°W], 1070m, 27 Jun–1 Jul 1973 (A. F. Newton);11km SW Chapulhuacán, [ca. 21.147°N, 98.966°W], 1200m, 5 Jul 1976 (A. F. Newton); 18km NE Jacala, nr El Alamo, [ca. 21.058°N, 99.052°W], 1700m, 10 Jun 1988 (S. & J. Peck); 16km NE Rancho Viejo, [ca. 21.07°N, 99.02°W], 1550m, 5 Jul 1976 (A. F. Newton); Tlanchinol, 43km SW Huejutla, [ca. 20.988°N, 98.662°W], 1 Aug 1983 (S. & J. Peck); 4–6km N Tlanchinol, [ca. 21.026°N, 98.644°W], 1590m, 6–11 Jul 1973 (A. F. Newton);***Jalisco***: Volcán de Tequila, 20.82517°N, 103.85509°W, 1840m, 26 Aug 2009 (M. G. Branstetter); Volcán de Tequila, 20.79293°N, 103.85388°W, 2660m, 26 Aug 2009 (M. G. Branstetter); ***México***: 1.6km E Ixtapan de la Sal, [ca. 18.85°N, 99.67°W], 1890m, 10 Aug 1973 (A. F. Newton); 4.8km NE Temascaltepec, [ca. 19.05°N, 100.03°W], 1920m, 14 Sep 1973 (A. F. Newton); 11km NE Temascaltepec, [ca. 19.096°N, 99.983°W], 2100m, 21–15 Sep 1973 (A. F. Newton); 3.2km NE Tenancingo, [ca. 18.985°N, 99.575°W], 2160m, 11 Sep 1973 (A. F. Newton); ***Oaxaca***: 9.4km SE Asunción Nochixtlan, 17.37632°N, 97.19976°W, 2240m, 10 Aug 2009 (M. G. Branstetter); 10.6km N Jct 190/135 (on 135), [ca. 17.282°N, 96.929°W], 1920m, 21 Jul 1987 (R. S. Anderson); 14km NE Oaxaca, km 10 Mex. 175, [ca. 17.145°N, 96.622°W], 1890m, 20 Aug 1973 (A. F. Newton); ***Puebla***: 2.7km S Apulco, nr Zacapoaxtla, [ca. 19.911°N, 97.606°W], 22 Jul 1987 (R. S. Anderson); 7.6km SW La Cumbre, [ca. 20.1416°N, 98.0217°W], 1585m, 23 Jul 1987 (R. S. Anderson); H’way 190, E Río Frío [ca. 19.310°N, 98.638°W], 2900m, 7 Aug 1965 (Cornell Univ. Mexico Field Party); 2km NE Teziutlan, [ca. 19.892°N, 97.296°W], 1220m, 19 Jun 1983 (S. & J. Peck); 24km N Xicotepec de Juarez, [ca. 20.282°N, 97.963°W], 1070m, 17 Jun 1983 (R. S. Anderson); ***Querétaro***: Cerro Zamorano, 20.9328°N, 100.1840°W, 2770m, 26 Jul 2006 (R. S. Anderson); 29km E Landa de Matamoros, [ca. 21.27°N, 99.16°W], 1600m, 14 Jul 1969 (S. & J. Peck); 40km E Landa de Matamoros, [ca. 21.297°N, 99.090°W], 1520m, 14 Jul 1969 (S. & J. Peck); 4.6km SW Pinal de Amoles, 21.16479°N, 99.59419°W, 1960m, 18 Aug 2009 (M. G. Branstetter); 1.9km NE Pinal de Amoles, 21.14974°N, 99.61576°W, 2250m, 18 Aug 2009 (M. G. Branstetter); 7km NE Pinal de Amoles, 21.17601°N, 99.57341°W, 1700m, 18 Aug 2009 (M. G. Branstetter); ***San Luis Potosí***: Taman, 20km SW Tamazunchale, [ca. 21.153°N, 98.947°W], 11 Jun 1983 (S. & J. Peck); 20km33/ W Xilitla, [ca. 21.293°N, 99.194°W], 1600m, 12 Jun 1983 (S. & J. Peck); 22.5km W Xilitla, [ca. 21.300°N, 99.086°W], 1460m, 29 Jun 1973 (A. F. Newton);40km W Xilitla, [ca. 21.259°N, 99.194°W], 1700m, 6 Aug 1983 (S. & J. Peck); ***Sinaloa***: 1.7km NNE El Palmito, 23.5783°N, 105.835°W, 2100m, 15 Jan 2007 (P. S. Ward); ***Tamaulipas***: El Cielo, 1.2km SE Alta Cima, 23.05005°N, 99.19226°W, 920m, 24 Aug 2009 (M. G. Branstetter); El Cielo, 1.8km W Alta Cima, 23.06110°N, 99.21564°W, 1340m, 23 Aug 2009 (M. G. Branstetter); El Cielo, 1.8km NW La Gloria, 23.05871°N, 99.26543°W, 2030m, 23 Aug 2009 (M. G. Branstetter); 0.8km N Conrado Castillo, [ca. 23.491°N, 99.308°W], 4 Sep 1970 (W. Elliott); **U.S.A.:** ***Arizona***: Cochise Co.: Carr Canyon, 11.6km from jct. 92, [ca. 31.428°N, 110.29°W], 2195m, 17 Aug 2002 (Cover & Deyrup); Hd. Carr Canyon, Huachuca Mts., [ca. 31.432°N, 110.284°W], 2440m, 24 Jul 1950 (W. S. Creighton); Chiricahua Mts, [ca. 31.829°N, 109.267°W], 2 Aug 1954 (A. C. Cole); Chiricahua Mts, trail to Barfoot Lookout from rd., [ca. 31.829°N, 109.267°W], 2560m, 26 Jun 1987 (S. P. Cover); Chiricahua Mtns, 8km WSW Portal, 31.883°N, 109.217°W, 1710m, 12 Aug 2002 (P. S. Ward); Chiricahua Mtns., 11.9km 260°W Portal, 31.917°N, 109.267°W, 2440m, 11 Aug 2003 (P. S. Ward); Chiricahua Mtns., W Turkey Creek, 20km 244° WSW Portal, 31.867°N, 109.350°W, 1800m, 13 Aug 2002 (P. S. Ward); Sunnyside Canyon, 5.1km SE jct. FSR48 on FSR227, [ca. 31.437°N, 110.401°W], 1810m, 18 Aug 2002 (Cover & Deyrup); ***New Mexico***: Bernanillo Co.: 15km E Alameda, 35.200°N, 106.483°W, 1950m, 14 Aug 1997 (P. S. Ward).

### Stenamma ignotum sp. n.

urn:lsid:zoobank.org:act:0198200D-E517-48E3-BE98-9A6F3606A651

http://species-id.net/wiki/Stenamma_ignotum

**Figure 96.**
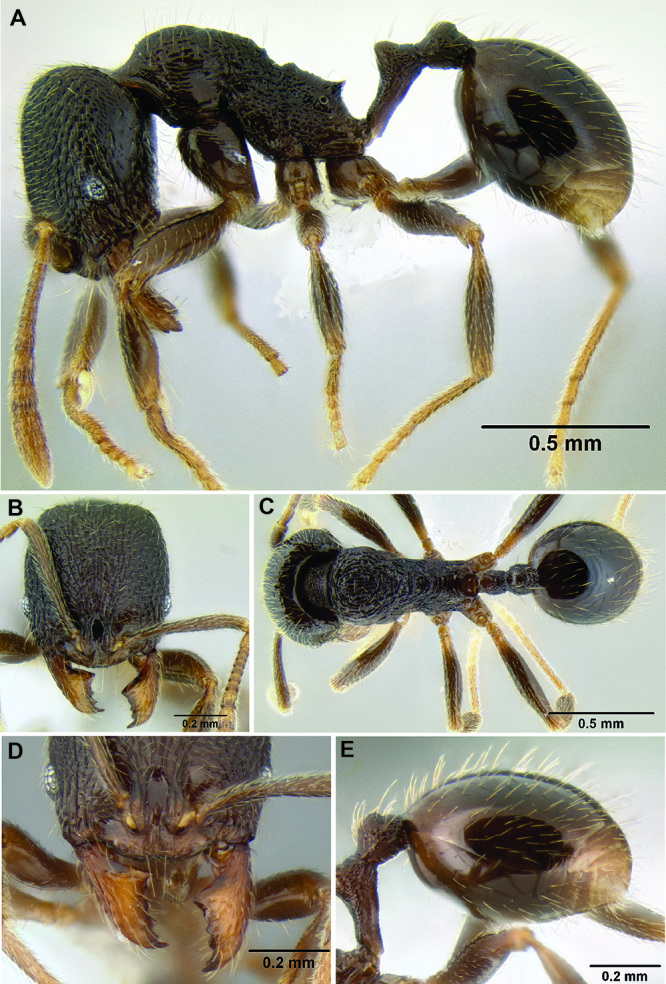
*Stenamma ignotum* holotype worker (CASENT0603762) **A** Profile **B** Face **C** Dorsum **D **Anterior clypeal margin in anterodorsal view **E** Gaster.

**Figure 97.**
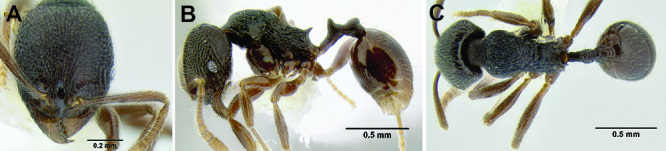
*Stenamma ignotum* worker variant (CASENT0604307) **A** Face **B** Profile **C** Dorsum.

**Figure 98.**
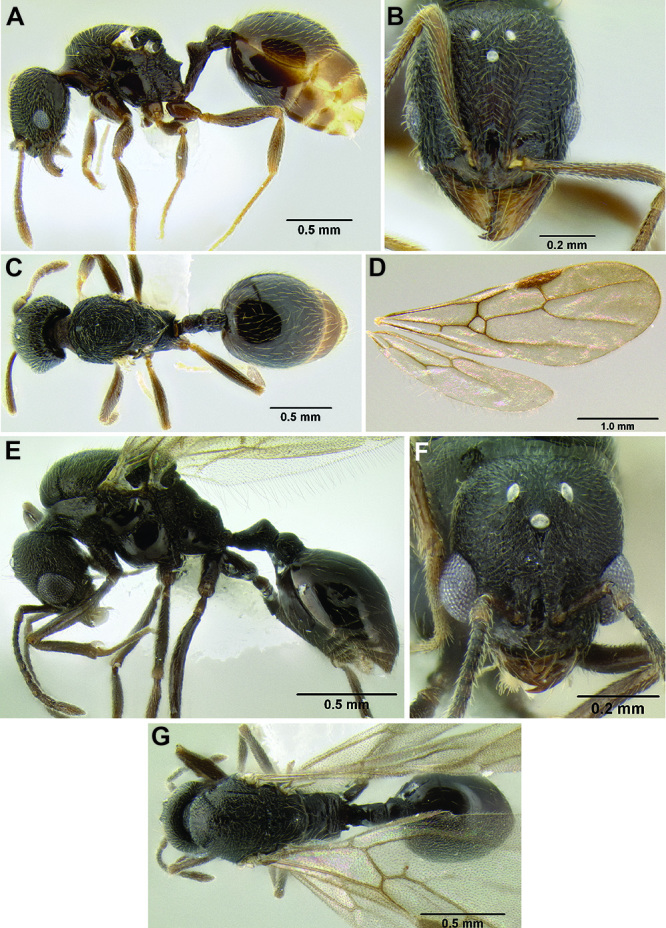
*Stenamma ignotum* **A** Paratype queen (CASENT0600182), profile **B** Same, face **C** Same, dorsum **D** Same, wings **E** Male (CASENT0607085), profile **F** Same, face **G** Same, dorsum.

**Figure 99.**
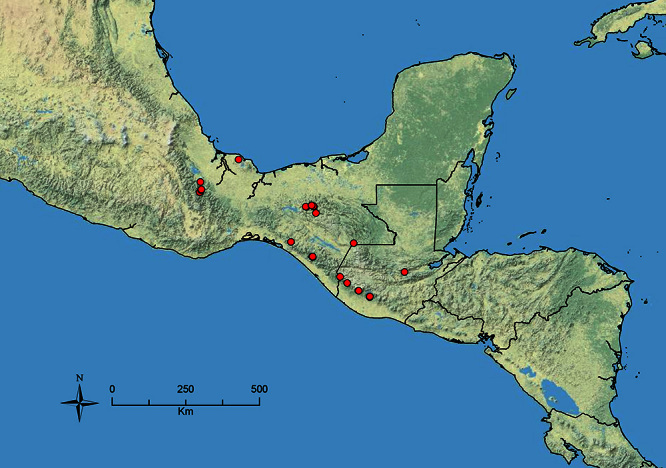
Distribution map of *Stenamma ignotum*.

Worker: Figures 96 , 97 ; Queen: Figure 98A–D ; Male: Figure 98E–G ; Map: Figure 99

Stenamma mgb10 Branstetter, 2012: phylogeny.

#### Type material.

*Holotype worker*. MÉXICO: Chiapas, 2km SE Custepec, 15.72102°N, 92.95013°W ±50m, 1520m, 17 May 2008, mesophyll forest, ex sifted leaf litter (LLAMA, collection Wa-A-02-1-09) [USNM, specimen CASENT0603762]. *Paratypes*: same data as holotype [1w, CAS, CASENT0623311], [1w, EAPZ, CASENT0623312]; same data but 3km ESE Custepec, 15.71660°N, 92.93715°W ±50m, 1700m, 18 Jul 2007 (J. Longino, JTL6073) [1w, ECOSCE, CASENT0620158], [1w, FMNH, CASENT0620159], [1w, ICN, CASENT0620160], [1w, INBio, CASENT0620161], [1w, LACM, CASENT0620162], [1w, MCZ, CASENT0622764], [1q, 1w, USNM, CASENT0600182, CASENT0622765]; 15.71508°N, 92.93822°W ±50m, 1700m, 17 May 2008 (LLAMA, Wa-A-02-2-45) [1w, MZSP, CASENT0623320], [1w, UCD, CASENT0623321], [1w, UNAM, CASENT0623322], [1w, UVGC, CASENT0623324], [1w, JTLC, CASENT0623323], [2w, MGBPC, CASENT0623326, CASENT0623325].

#### Worker diagnosis.

Integument mostly dark brown to brown; small-sized species (see HL, ML, PrW below); basal margin of mandible straight, never with a basal notch or depression; anterior clypeal margin with a shallow median emargination (best viewed from an anterodorsal angle); face completely sculptured, mostly rugoreticulate; mesosoma mostly sculptured, dorsum of promesonotum with dense longitudinal rugae; eye of moderate size (EL 0.10–0.12, REL 18–21), oval-shaped, with 5–7 ommatidia at greatest diameter; gastral pilosity relatively long, sparse, and mostly suberect, sometimes with a few underlying decumbent setae; petiole in profile appearing somewhat elongate; petiolar node reaching a distinct apex, which points vertically; propodeal spines tuberculate to short (PSL 0.07–0.12, PSI 1.2–2.1); frontal lobes of moderate width (FLD 0.14–0.16, FLI 24–27), not greatly obscuring torular lobes in full-face view. *Similar species*: *Stenamma crypticum*, *Stenamma nonotch*, *Stenamma picopicucha*.

#### Geographic range.

Southern Mexico to Guatemala.

#### Worker description.

(18 measured) HL 0.59–0.68 (0.64), HW 0.53–0.61 (0.55), FLD 0.14–0.16 (0.14), PCW 0.02–0.03 (0.02), SL 0.47–0.55 (0.50), EL 0.10–0.12 (0.10), ACL 0.45–0.52 (0.49), ML 074–0.86 (0.74), PrW 0.36–0.43 (0.37), PSL 0.07–0.12 (0.07), SDL 0.05–0.07 (0.05), PL 0.25–0.32 (0.27), PH 0.15–0.18 (0.15), PW 0.11–0.16 (0.12), PPL 0.14–0.18 (0.15), PPH 0.13–0.17 (0.13), PPW 0.15–0.19 (0.15), MFL 0.52–0.62 (0.54), MTL 0.42–0.50 (0.45), CI 87–92 (87), SI 85–96 (89), REL 18–21 (18), FLI 24–27 (26), PSI 1.2–2.1 (1.6), MFI 96–104 (103), ACI1 66–70 (67), ACI2 92–100 (98).

Small-sized species; general body color mostly dark brown to brown, with appendages brown to orange-brown, becoming lighter toward extremities; setae golden brown; mandible with 5–6 teeth, consisting of 3 distinct apical teeth, a basal tooth, and 1–2 smaller inner teeth/denticles, which are often worn and indistinct; basal margin of mandible straight, without basal notch or depression; mandible mostly smooth and shiny, with scattered piligerous punctae, and some striations around base and along lateral surface; anterior clypeal margin with a shallow median emargination (best viewed from anterodorsal angle); median lobe of clypeus with a pair of faint longitudinal carinulae that diverge toward anterior margin, apex of lobe with a short transverse carinula, remainder of clypeus mostly smooth; posterior extension of clypeus between antennal insertions of moderate width (PCW 0.02–0.03), sides subparallel to slightly hour-glass-shaped; frontal lobes of moderate width (FLD 0.14–0.16, FLI 24–27), not greatly obscuring torular lobes in full-face view; head roughly oval-shaped (CI 87–92), with posterior margin slightly depressed medially; eye of moderate size (EL 0.10–0.12, REL 18–21), oval-shaped, with 5–7 ommatidia at greatest diameter; face mostly rugoreticulate, with some longitudinal rugae along midline; scape of moderate length (SI 85–96), almost reaching posterior margin when laid back; scape surface smooth and shiny, with scattered piligerous punctae; flagellum with distinct 4-segmented antennal club; mesosoma almost completely sculptured except for a small patch of smooth cuticle on side of pronotum; dorsum of pronotum densely rugose (longitudinal orientation), transitioning to rugoreticulae on mesonotum; upper half of side of mesonotum rugose; mesopleuron mostly punctate, with some rugulae; side of propodeum rugulose-punctate; dorsum and declivity of propodeum with transverse carinulae; promesonotum in profile low-domed, slightly asymmetrical with apex shifted anterior of midpoint; metanotal groove of moderate width and depth; propodeal spines tuberculate to short (PSL 0.07–0.12, PSI 1.2–2.1); petiole appearing of moderate length to slightly elongate (PL/HW 0.47–0.54); petiolar node in profile of moderate height (PH/PL 0.55–0.66), and roughly symmetrical, dorsum pointing vertically to slightly posteriad, and usually reaching a well-defined apex; postpetiole in profile slightly asymmetrical, with anterior face longer and more sloping than posterior face, overal size similar to petiolar node (PPH/PH 0.85–0.98); anterior faces of petiolar and postpetiolar nodes mostly smooth and shiny, remaining surfaces faintly punctate; gaster smooth and shiny, with scattered piligerous punctae; most of body dorsum with short to long standing pilosity; setae on gastral dorsum relatively sparse, mostly long and suberect, with a few shorter decumbent setae underneath; setae on scape and legs decumbent to appressed, with some longer suberect setae on femoral venters and coxae.

#### Queen description.

(5 measured) HL 0.66–0.74 (0.66), HW 0.61–0.67 (0.61), FLD 0.15–0.18 (0.15), PCW 0.02–0.03 (0.03), SL 0.52–0.58 (0.52), EL 0.17–0.20 (0.17), ACL 0.49–0.55 (0.49), ML 0.94–1.09 (0.94), PrW 0.54–0.63 (0.54), PSL 0.13–0.17 (0.13), SDL 0.09–0.10 (0.10), PL 0.37–0.40 (0.37), PH 0.19–0.22 (0.19), PW 0.16–0.19 (0.16), PPL 0.16–0.22 (0.16), PPH 0.18–0.23 (0.18), PPW 0.20–0.24 (0.20), MFL 0.59–0.70 (0.59), MTL 0.50–0.58 (0.50), CI 90–93 (92), SI 85–91 (85), REL 28–30 (28), FLI 25–27 (25), PSI 1.4–1.7 (1.4), MFI 95–103 (1.03), ACI1 64–68 (67), ACI2 94–96 (96).

Same as worker except for standard queen modifications and as follows: pronotum with transverse rugae/carinulae; mesoscutum densely longitudinally carinulae; scutellum rugose to rugoreticulate; propodeum with transverse carinulae that wrap around entire surface; mesopleuron mostly smooth; gastral pilosity denser, especially lower decumbent layer; wing as in Figure 98D.

#### Male.

See Figure 98E–G.

#### Biology.

*Stenamma ignotum* is found in a diversity of wet forest habitats (e.g. cloud forest, mesophyll forest, wet oak forest) from 500 m to 2070 m elevation, but is most common above 1,000 m. Most collections are from sifted leaf litter taken from the forest floor, but it is also known from cookie baits and by general searching. A couple of nests have been found: one underneath a rock, and the other under epiphytes on the ground, both in montane wet forest. A single stray worker has been collected from a mud bank.

#### Comments.

This species is very similar to *Stenamma crypticum*, *Stenamma nonotch*, and *Stenamma picopicucha*, but should always be separable by its having the basal margin of the mandible straight and the anterior clypeal margin with a distinct median emargination. Some specimens of *Stenamma nonotch* have a nearly imperceptible notch in the anterior clypeal margin, but this is always very insignificant compared to the emargination present in *Stenamma ignotum*. Sculpture on the pronotal dorsum can be compared to confirm species status, with *Stenamma ignotum* always having distinct longitudinal carinulae and *Stenamma nonotch* having rugoreticulae or very irregular rugae. As described in the comments section of *Stenamma crypticum* and *Stenamma picopicucha*, the latter species is intermediate between *Stenamma crypticum* and *Stenamma ignotum* and it is unclear to which it is most closely related. There is a significant gap in distribution between *Stenamma ignotum* and *Stenamma picopicucha*, so I highly doubt that they are conspecific, but this has yet to be tested by including *Stenamma picopicucha* in a molecular phylogeny.

There is some variation in size and sculpture among populations of *Stenamma ignotum*, but the differences are minute. The only exception is a distinct variant (Figure 97) known from a single specimen from Guatemala (7.3km E Purulhá). This specimen has very long propodeal spines, and the dorsum of the petiolar node in profile reaches a rather sharp apex, which points distinctly posteriad. Until more specimens are found, I treat this variation as intraspecific. I have noticed that many species become more aberrant in the area near Purulhá, possibly because the area is very wet and cold.

I have sampled a number of populations of *Stenamma ignotum* for molecular phylogenetic analysis (Branstetter unpublished data). The data indicate that the species is monophyletic and is sister to *Stenamma stictosomum*. I have not been able to find any morphological characters to adequately diagnose this clade.

#### Material examined.

**GUATEMALA: *Baja Verapaz***: 7.3km E Purulhá, 15.267°N, 90.132°W, 1700m, 19 May 1991 (R. S. Anderson); ***Quetzaltenango***: Km 240 road from coast-Zunil, Finca Santa Maria, 14.71686°N, 91.53481°W, 1500m, 13 Sep 2008 (R. S. Anderson); 1.7km SW Santa Maria, 14.7169°N, 91.5348°W, 1515m, 13 Sep 2008 (L. Sáenz); ***San Marcos***: Rd. Bojonal-Fraternidad, 14.94533°N, 91.88038°W, 1580m, 11 Sep 2008 (R. S. Anderson); 9.8km WSW San Marcos, 14.9443°N, 91.8799°W, 1600m, 11 Sep 2008 (M. G. Branstetter); ***Suchitepéquez***: 4km S Vol. Atitlán, 14.54804°N, 91.19191°W, 1575m, 16 Jun 2009 (LLAMA); 4km S Vol. Atitlán, 14.54391°N, 91.19434°W, 1435m, 17 Jun 2009 (LLAMA); 5.5km S Vol. Atitlán, 14.52857°N, 91.19569°W, 1070m, 18 Jun 2009 (J. Longino); **MÉXICO:** ***Chiapas***: 5.9km E Bochil, [ca. 16.9947°N, 92.8357°W], 1300m, 15 Sep 1992 (R. S. Anderson); 5km NE Coapilla, 17.17602°N, 93.13293°W, 1990m, 26 May 2008 (LLAMA); 5km NNW Coapilla, 17. 18355°N, 93.15222°W, 1915m, 26 May 2008 (LLAMA); 5.8km NE Coapilla, 17.17453°N, 93.13152°W, 2030m, 12 Jul 2007 (R. S. Anderson); Custepec, 15.71264°N, 92.94037°W, 1660m, 18 May 2008 (J. Longino); 2km SE Custepec, 15.72099°N, 92.95045°W, 1520m, 17 May 2008 (LLAMA); 2km SE Custepec, 15.7212°N, 92.9391°W, 1830m, 18 May 2008 (R. S. Anderson); 3km SE Custepec, 15.71512°N, 92.93797°W, 1700m, 17 May 2008 (LLAMA); Lagos de Montebello, Cinco Lagos, [ca. 16.1167°N, 91.6833°W], 1500m, 22 Sep 1992 (R. S. Anderson); 2.1km NW Pueblo Nuevo Solistahuacan, Yerbabuena Preserve, [ca. 17.183°N, 92.900°W], 2070m, 23 Sep 1992 (R. S. Anderson); 4.8km N Pueblo Nuevo Solistahuacán, [ca. 17.185°N, 92.905°W], 1860m, 26–27 Aug 1973 (A. F. Newton); 5km E Rayón, 17.217°N, 92.967°W, 1700m, 23 Dec 1991 (P. S. Ward); 8.9km E Rayon, 17.20000°N, 92.91633°W, 1500m, 19 Sep 1991 (R. S. Anderson); Sierra Morena, 16.15292°N, 93.60051°W, 1330m, 12 May 2008 (LLAMA); 4km N Union Juarez, Volcan Tacana, lower slopes, [ca. 15.133°N, 92.100°W], 1800m, 18 Sep 1992 (R. S. Anderson); ***Oaxaca***: Mirador Grande, 17.89844°N, 96.36253°W, 990m, 14 Aug 2009 (M. G. Branstetter); 10.8km SW Valle Nacional, 17.68102°N, 96.33026°W, 1120m, 13 Aug 2009 (M. G. Branstetter); 13.2km SW Valle Nacional, 17.65934°N, 96.33426°W, 1360m, 11 Aug 2009 (M. G. Branstetter); 20.5km SW Valle Nacional, 17.60560°N, 96.38298°W, 1770m, 12 Aug 2009 (M. G. Branstetter); 20.6km SW Valle Nacional, 17.60404°N, 96.37786°W, 1733m, 13 Aug 2009 (M. G. Branstetter); ***Veracruz***: Los Tuxtlas, 10km NNW Sontecomapan, 18.583°N, 95.083°W, 500m, 21 Mar 1985 (P. S. Ward); 11km N San Andrés Tuxtla, 18.550°N, 95.200°W, 1400m, 23 Mar 1985 (P. S. Ward).

### Stenamma lagunum sp. n.

urn:lsid:zoobank.org:act:9F8628F3-6901-42BD-924E-FF6B22F6B1B6

http://species-id.net/wiki/Stenamma_lagunum

**Figure 100.**
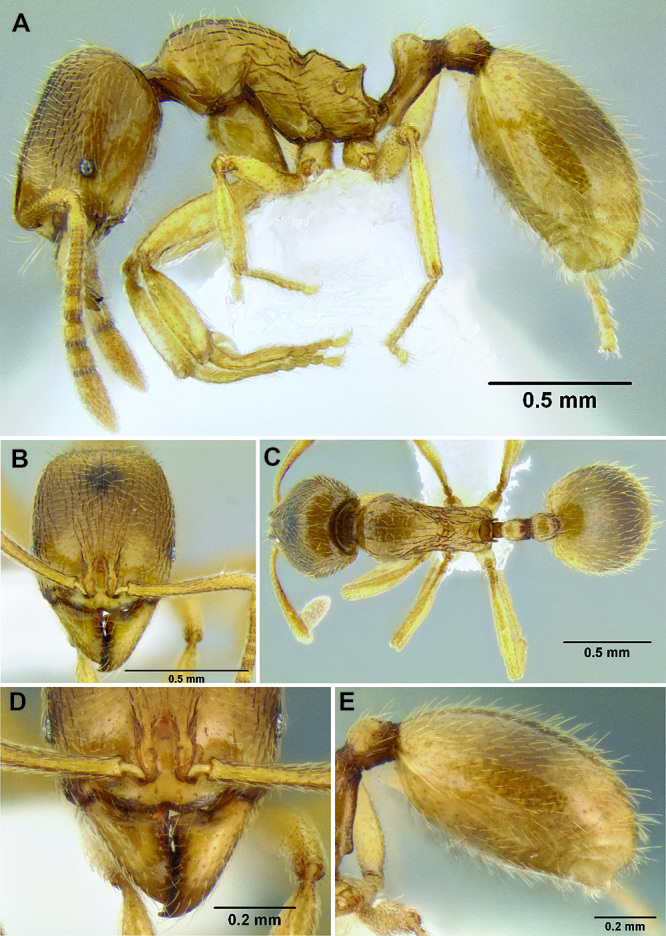
*Stenamma lagunum* holotype worker (CASENT0622371) **A** Profile **B** Face **C** Dorsum **D **Anterior clypeal margin in anterodorsal view **E** Gaster.

**Figure 101.**
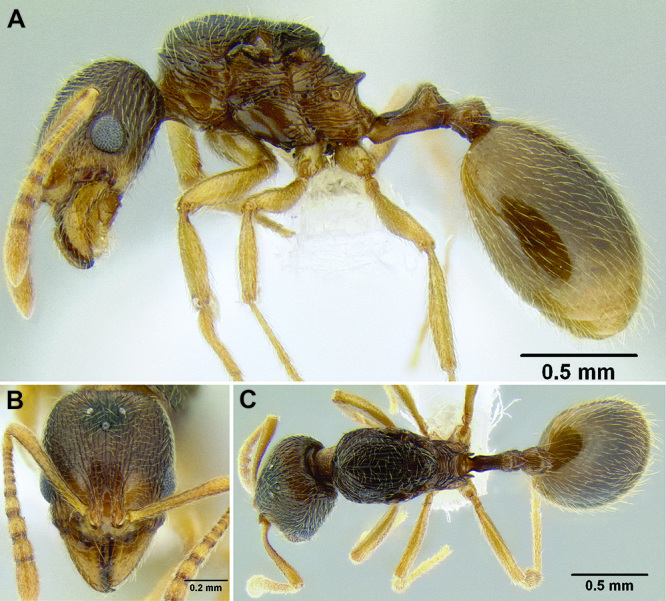
*Stenamma lagunum* paratype queen (CASENT0622384) **A** Profile **B** Face **C** Dorsum.

**Figure 102.**
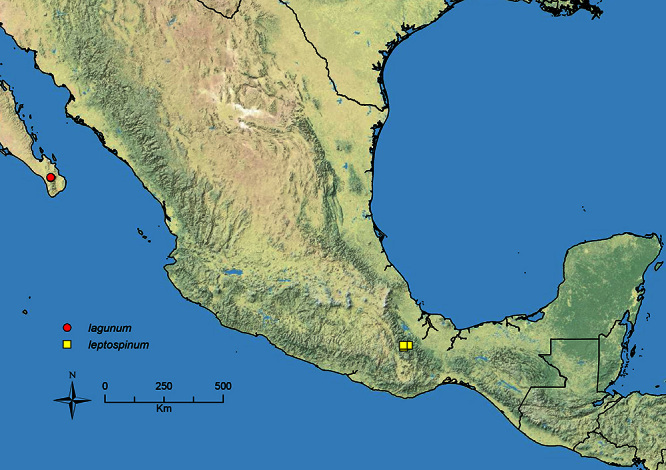
Distribution map of *Stenamma lagunum* (circles) and *Stenamma leptospinum* (squares).

Worker: Figure 100 ; Queen: Figure 101 ; Map: Figure 102

Stenamma mgb78 Branstetter, 2012: phylogeny.

#### Type material.

*Holotype worker*.MÉXICO: Baja California Sur, Sierra La Laguna, 23.54600°N, 110.00215°W ±10m, 1860m, 2 Oct 2010 (M. G. Branstetter, collection MGB1676) [USNM, specimen CASENT0622371]. *Paratypes*: same data as holotype but 23.56106°N, 110.01026°W ±10m, 1890m, 4 Oct 2010 (M. G. Branstetter, MGB1691) [1dq, 1w, USNM, CASENT0622384, CASENT0622386]; 23.54580°N, 110.01047°W ±10m, 1730m, 3 Oct 2010 (M. G. Branstetter, MGB1684) [1w, CAS, CASENT0622377], [1w, EAPZ, CASENT0622378], [1w, ECOSCE, CASENT0622379], [1w, FMNH, CASENT0622380], [1w, ICN, CASENT0622376], [1w, INBio, CASENT0623327], [1w, JTLC, CASENT0623328], [1w, LACM, CASENT0623329], [1w, MGBPC, CASENT0623330], [1w, MCZ, CASENT0623331], [1w, MZSP, CASENT0623332], [1w, UCD, CASENT0623333], [1w, UNAM, CASENT0623334], [1w, UVGC, CASENT0623335].

#### Worker diagnosis.

Integument mottled, pale yellow-brown with patches of darker brown; small-sized species (see HL, ML, PrW below); head and mesosoma almost completely covered with light longitudinal rugulae; eye relatively small (EL 0.06–0.08, REL 10–13), subcircular, with 3–5 ommatidia at greatest diameter; basal margin of mandible slightly sinuous, without a basal notch or deep depression; anterior clypeal margin with a single median emargination; frontal lobes somewhat narrow, not completely obscuring torular lobes in full-face view (FLD 0.15-0.16, FLI 25-28); propodeal spines present, forming short broad triangles (PSL 0.11–0.12, PSI 1.4-1.6); gastral pilosity short and clearly bilayered, with a layer of suberect setae and a layer of decumbent setae. *Similar species*: *Stenamma excisum*, *Stenamma californicum* (HOC species).

#### Geographic range.

Northwestern Mexico (Baja California Sur).

#### Worker description.

(5 measured) HL 0.66–0.70 (0.68), HW 0.55–0.61 (0.58), FLD 0.15–0.16 (0.15), PCW 0.03–0.04 (0.04), SL 0.54–0.56 (0.56), EL 0.06–0.08 (0.06), ACL 0.54–0.58 (0.59), ML 0.79–0.85 (0.83), PrW 0.38–0.40 (0.39), PSL 0.11–0.12 (0.11), SDL 0.05–0.07 (0.06), PL 0.29–0.32 (0.30), PH 0.18–0.19 (0.19), PW 0.12–0.13 (0.13), PPL 0.16–0.17 (0.16), PPH 0.14–0.16 (0.15), PPW 0.15–0.18 (0.17), MFL 0.59–0.60 (0.59), MTL 0.49–0.52 (0.52), CI 83–87 (86), SI 92–98 (95), REL 10–13 (10), FLI 25–28 (26), PSI 1.4–1.6 (1.4), MFI 94–101 (98), ACI1 65–67 (67), ACI2 97–103 (97).

Small-sized species; general body color a mottled pale yellow-brown with patches of darker brown, appendages similar; setae golden brown; mandible with 6 teeth, consisting of 2–3 distinct apical teeth, a basal tooth, and 2-3 middle teeth, which are usually worn and indistinct; basal margin of mandible straight to slightly sinuous, without a basal notch or deep depression; mandible mostly smooth and shiny, with scattered piligerous punctae; anterior clypeal margin with a median emargination, clypeus bordering emargination slightly projecting and attenuate, appearing translucent; median lobe of clypeus obliquely flattened, lacking longitudinal carinulae, apex near anterior clypeal margin with a short transverse carinula, remainder of clypeus smooth and shiny; posterior extension of clypeus between antennal insertions of moderate width (PCW 0.03–0.04), sides subparallel; frontal lobes somewhat narrow (FLD 0.15–0.16, FLI 25–28), with underlying torular lobes clearly visible in full-face view; head appearing subrectangular to oval-shaped (CI 83–87), posterior margin flat to very slightly depressed medially; eye relatively small (EL 0.06–0.08, REL 10-13), subcircular, with 3–5 ommatidia at greatest diameter; face completely covered with longitudinal rugulae and piligerous punctae, rugulae sometimes merging, but never becoming truly reticulate, posterior margin of head near occipital foramen mostly smooth and shiny; scape of moderate length (SI 92–98), not quite reaching posterior margin of head when laid back; scape surface mostly smooth and shiny, with scattered piligerous punctae and fine striations; flagellum with a somewhat distinct 4-segmented antennal club; mesosoma mostly with longitudinal carinulae and rugulae, some faint punctae also present on most surfaces (a few aberrant specimens with transverse carinulae on pronotum); propodeal dorsum and declivity with faint transverse carinulae; promesonotum in profile low-domed, roughly symmetrical; metanotal grove well demarcated, of moderate width and depth; propodeal spines present, forming short broad triangular projections (PSL 0.11–0.12, PSI 1.4–1.6); petiole in profile somewhat compact (PL/PW 51–55), node domed and pointing slightly posteriad, node dorsum flat to gently rounded; postpetiole somewhat dorsoventrally compressed, clearly smaller than petiolar node (PPH/PH 0.77–0.82); petiolar and postpetiolar nodes mostly smooth and shiny, remaining waist surfaces faintly punctate; gaster mostly smooth and shiny, with scattered piligerous punctae; most of body with short standing pilosity; setae on scape decumbent to appressed; gastral pilosity short, somewhat dense, and clearly bilayered, with a layer of suberect setae and a layer of decumbent setae (setae on head similar); setae on legs mostly decumbent to appressed, with some suberect setae on venter of profemur.

#### Queen description.

(1 measured) HL 0.71, HW 0.61, FLD 0.16, PCW 0.04, SL 0.57, EL 0.17, ACL 0.59, ML 0.97, PrW 0.50, PSL 0.15, SDL 0.15, PL 0.34, PH 0.21, PW 0.14, PPL 0.17, PPH 0.17, PPW 0.20, MFL 0.64, MTL 0.55, CI 86, SI 94, REL 29, FLI 26, PSI 1.7, MFI 96, ACI1 62, ACI2 103.

Same as worker except for standard queen modifications and as follows: body color a darker yellow-brown; pronotum with transverse carinulae on sides, smooth in middle; mesoscutum carinulate, with carinulae long and distinct; katepisternum mostly smooth; propodeum with transverse carinulae that wrap around surface; propodeal spines blunt, slightly longer.

#### Male.

Unknown.

#### Biology.

*Stenamma lagunum* is known only from extractions of sifted leaf litter. It is a high-elevation species, occurring from 1730–1900 m and is found in mesic forest habitats, such as oak-pine forest, oak forest, and montane scrub.

**Comments.** *Stenamma lagunum* is a very distinctive species that appears to be endemic to the mountains of southern Baja CA. It might be confused with its sister species *Stenamma excisum*, but can be easily separated by its carinulate facial sculpture and more projecting median lobe of the clypeus. There is the chance that if *Stenamma lagunum* occurs farther north in Baja CA, it might overlap with Holarctic *Stenamma* species, such as *Stenamma californicum*. HOC species should be easy to separate out by their distinct clypeal carinae.

#### Material examined.

**MÉXICO:** ***Baja California Sur***: Sierra La Laguna, 23.54580°N, 110.01047°W, 1730m, 3 Oct 2010 (M. G. Branstetter); Sierra La Laguna, 23.54967°N, 109.98320°W, 1770m, 5 Oct 2010 (M. G. Branstetter); Sierra La Laguna, 23.54600°N, 110.00215°W, 1860m, 2 Oct 2010 (M. G. Branstetter); Sierra La Laguna, 23.56318°N, 110.01337°W, 1900m, 4 Oct 2010 (M. G. Branstetter).

### Stenamma leptospinum sp. n.

urn:lsid:zoobank.org:act:C1DF56CC-F740-4D7D-9E25-7842EFADEEE0

http://species-id.net/wiki/Stenamma_leptospinum

**Figure 103.**
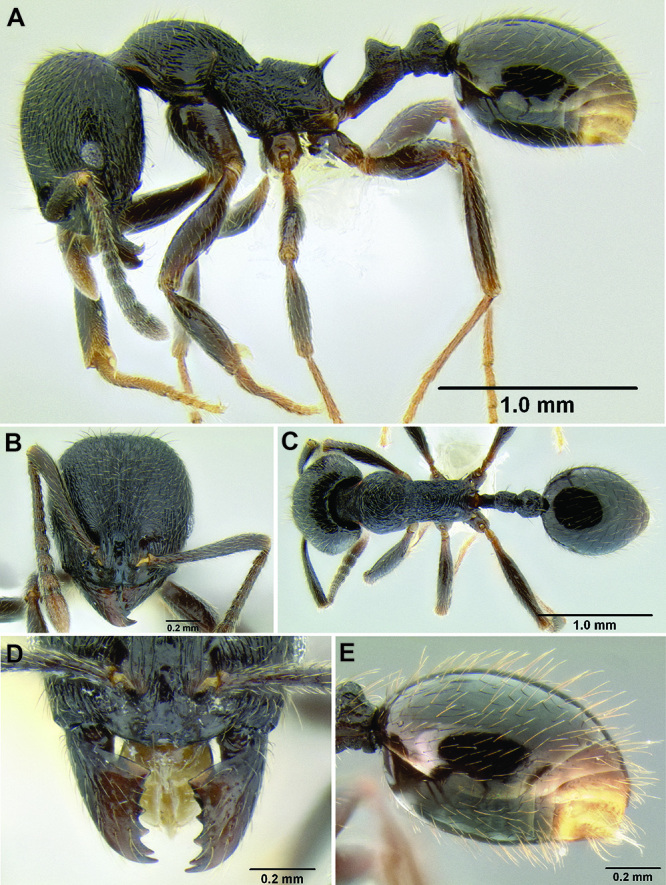
*Stenamma leptospinum* holotype worker (CASENT0605530) **A** Profile **B** Face **C** Dorsum **D** Anterior clypeal margin in anterodorsal view **E** Gaster.

Worker: Figure 103 ; Map: Figure 102

#### Type material.

*Holotype worker*. MEXICO: Oaxaca, 22.4km SW Valle Nacional, 17.59112°N, 96.39138°W ±20m, 1990m, 13 Aug 2009, cloud forest, ex sifted leaf litter (M. G. Branstetter, collection MGB1391) [USNM, specimen CASENT0605530]. *Paratypes*: same data as holotype [1w, CAS, CASENT0605531], [1w, MGBPC, CASENT0605555], [1w, MCZ, CASENT0605532], [1w, UCD, CASENT0605529], [1w, UNAM, CASENT0605533], [1w, USNM, CASENT0605554].

#### Worker diagnosis.

Integument mostly dark brown to brown; medium-sized species (see HL, HW, ML below); lateral margin of hypostomal bridge with a projecting subquadrate to broadly-rounded lobe (usually visible behind mandible in profile); propodeal spines long and rather slender (PSL 00.17–0.22, PSI 1.9–2.2); anterior clypeal margin with a median emargination; basal margin of mandible straight, without a basal notch or depression; head and mesosoma densely carinulate; eye large (EL 0.13–0.15, REL 16–19), with 6–8 ommatidia at greatest diameter; petiolar node in profile subconical, with apex well-defined, almost sharp; postpetiolar node anteroposteriorly compressed; gastral pilosity with a layer of long suberect setae and a sparse layer of shorter decumbent setae; frontal lobes of moderate width (FLD 0.21–0.23, FLI 26–31), not completely obscuring torular lobes in full-face view. *Similar species*: *Stenamma longinoi*, *Stenamma manni*, *Stenamma muralla*.

#### Geographic range.

Southern Mexico.

#### Worker description.

(10 measured) HL 0.80–0.93 (0.91), HW 0.70–0.87 (0.87), FLD 0.21–0.23 (0.23), PCW 0.04–0.08 (0.04), SL 0.69–0.80 (0.75), EL 0.13–0.15 (0.15), ACL 0.62–0.68 (0.68), ML 1.02–1.18 (1.17), PrW 0.47–0.55 (0.55), PSL 0.17–0.22 (0.22), SDL 0.08–0.10 (0.10), PL 0.35–0.43 (0.42), PH 0.22–0.26 (0.26), PW 0.15–0.19 (0.19), PPL 0.19–0.25 (0.25), PPH 0.20–0.25 (0.25), PPW 0.19–0.25 (0.24), MFL 0.79–0.92 (0.89), MTL 0.63–0.74 (0.73), CI 87–96 (96), SI 87–98 (87), REL 16–19 (18), FLI 26–31 (26), PSI 1.9–2.2 (2.2), MFI 88–97 (97), ACI1 63–66 (64), ACI2 85–92 (91).

Medium-sized species; general body color dark brown to brown, with appendages brown to orange-brown; setae dark golden brown; mandible with 6 teeth, with two teeth nearest basal tooth smaller, often worn and indistinct; basal margin of mandible straight, without a basal notch or depression; mandible surface mostly smooth and shiny, with scattered piligerous punctae; anterior clypeal margin with a median emargination; median lobe of clypeus with a pair of faint longitudinal carinulae that diverge anteriorly, apex with a short transverse carinula, remainder of clypeus mostly smooth and shiny, with a few faint striae on median lobe; posterior extension of clypeus between antennal insertions of moderate to somewhat wide width (PCW 0.04–0.08); frontal lobes of moderate width (FLD 0.21–0.23, FLI 26–31), not completely obscuring torular lobes in full-face view; lateral margin of hypostomal bridge with a projecting subquadrate to broadly-rounded lobe that is usually visible behind mandible in profile, but some specimens with lobe reduced; head subcircular to oval-shaped (CI 87–96), posterior margin with a slight median depression; eye large (EL 0.13–0.15, REL 16–19), roughly oval-shaped, with 6–8 ommatidia at greatest diameter; face densely sculptured with a fan of longitudinal carinulae that extend outward from frontal lobes, lateral margins with shorter less densely spaced rugulae, interstices faintly punctate; scape of moderate length (SI 87–98), nearly reaching posterior margin of head when laid back; scape surface mostly smooth and shiny with scattered piligerous punctures; flagellum with a somewhat distinct 4-segmented antennal club; mesosoma densely sculptured; promesonotum mostly longitudinally carinulate, with some transverse carinulae wrapping around anterior declivity; mesopleuron and side of propodeum with irregular rugulae, rugoreticulae, and punctae; propodeal dorsum and part of declivity with transverse carinulae, remainder of declivity smooth and shining; promesonotum in profile low-domed and somewhat asymmetrical, with posterior slope longer and shallower than anterior slope; metanotal grove well demarcated and moderately deep, forming a rather smooth transition with promesonotum; anterior margin of propodeal dorsum in profile sometimes distinctly raised and separated from metanotal grove; propodeal spines long and rather slender (PSL 00.17–0.22, PSI 1.9–2.2), pointing anteroposteriorly and outward from body; petiole of moderate length (PL/HW 0.47–0.51), peduncle in profile somewhat slender; petiolar node in profile somewhat high (PH/PL 0.59–0.69), subconical and roughly symmetrical, with a well-defined apex that points vertically; postpetiolar node in profile usually somewhat anteroposteriorly compressed, with apex rounded and pointing vertically or slightly posteriad; anterior faces of petiolar and postpetiolar nodes smooth and shiny, posterior faces with punctae and a few rugulae, remaining surface of waist punctate; gaster mostly smooth and shiny, with scattered piligerous punctae and a few short striae around anterior constriction; most of body with rather long standing pilosity; scape with a uniform layer of subdecumbent to appressed setae; gaster with a layer of long suberect setae and a sparse layer of shorter decumbent setae; setae on legs mostly subdecumbent to appressed, with suberect setae on coxae and femoral venters.

#### Queen.

Unknown.

#### Male.

Unknown.

#### Biology.

*Stenamma leptospinum* is known only from Winkler and Berlese samples of sifted leaf litter taken from the forest floor. It has been collected from 1650–2000 m elevation in montane wet forest habitats, including cloud forest and montane oak forest.

#### Comments.

This species could be confused with *Stenamma longinoi*, *Stenamma manni*, or *Stenamma muralla*, but it should be easy to distinguish by its dense carinulate sculpture and long, slender propodeal spines.

It is worth noting that the lateral hypostomal lobe that groups *Stenamma leptospinum* with the other similar species is sometimes reduced, usually becoming shorter and only broadly rounded. When the lobe is reduced it can be more difficult to see in profile view, but it should still be visible from a lateroventral view. I have not seen any specimens where it is completely reduced to a point, as in most other species of *Stenamma*.

#### Material examined.

**MÉXICO:** ***Oaxaca***: 22.4km SW Valle Nacional, 17.59112°N, 96.39133°W, 1990m, 13 Aug 2009 (M. G. Branstetter); 40km SW Valle Nacional, Km93, [ca. 17.5278°N, 96.5834°W], 1900m, 26 Jul 1992 (R. S. Anderson); 48km S Valle Nacional, km 97, [ca. 17.595°N, 96.600°W], 2012m, 25 Jun 1983 (S. & J. Peck).

### Stenamma llama sp. n.

urn:lsid:zoobank.org:act:041377B8-5DA9-4C7A-B55F-62EF37C5BC51

http://species-id.net/wiki/Stenamma_llama

**Figure 104.**
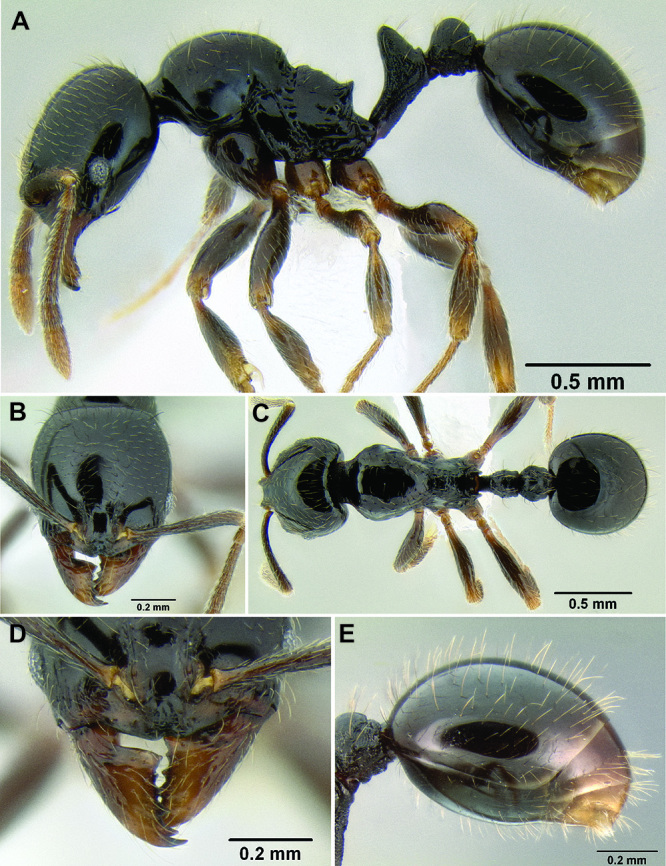
*Stenamma llama* holotype worker (CASENT0605236) **A** Profile **B** Face **C** Dorsum **D **Anterior clypeal margin in anterodorsal view **E** Gaster.

**Figure 105.**
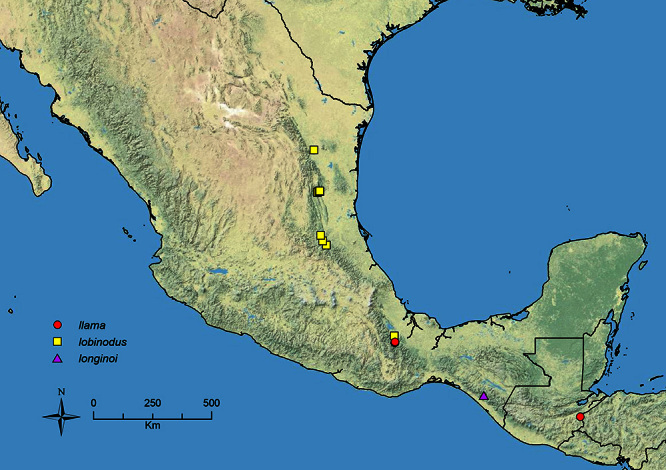
Distribution map of *Stenamma llama* (circles), *Stenamma lobinodus* (squares), and *Stenamma longinoi* (triangles).

Worker: Figure 104 ; Map: Figure 105

#### Type material.

*Holotype worker*. GUATEMALA: Zacapa, 2km SE La Unión, 14.95284N 89.27655W ±60m, 1450m, 14 May 2009 (LLAMA, collection Ba-B-03-4-04-13) [USNM, specimen CASENT0605236]. *Paratypes*: same data as holotype but 14.94654°N, 89.27600°W ±6m, 1550m, 12 May 2009 (LLAMA, Wa-B-03-1-01) [1w, CAS, CASENT0604931]; 14.94711°N, 89.27677°W ±50m, 1550m, 12 May 2009 (LLAMA, Wa-B-03-1-36) [1w, MGBPC, CASENT0604952]; 14.95436°N, 89.27690°W ±50m, 1430m, 12 May 2009 (LLAMA, Wa-B-03-2-10) [1w, MCZ, CASENT0604975], [1w, UCD, CASENT0606186], [1w, UNAM, CASENT0604974]; 14.95369°N, 89.27614°W ±50m, 1430m, 12 May 2009 (LLAMA, Wa-B-03-2-32) [1w, UVGC, CASENT0604982].

#### Worker diagnosis.

Integument black to brown-black; medium-sized species (see HL, ML, PrW below); anterior margin of clypeus with shallow median emargination; basal margin of mandible straight, without notch or substantial depression; head and mesosoma mostly smooth and shining; mesosoma compact, with promesonotum distinctly bulging; petiolar node robust, tall, and distinctly angled posteriad; propodeal spines forming short broad triangles (PSL 0.09–0.14, PSI 1.7-2.5); setae on gastral tergites sparse, long, and suberect; eye of moderate size (EL 0.11–0.15, REL 19-23), oval-shaped, with 6–8 ommatidia at greatest diameter; frontal lobes narrow, not obscuring torular lobe in full-face view (FLD 0.14-0.18, FLI 25-28). *Similar species*: *Stenamma lobinodus*, *Stenamma tiburon*.

#### Geographic range.

Southern Mexico to Guatemala.

#### Worker description.

(8 measured) HL 0.59–0.72 (0.70), HW 0.56–0.69 (0.64), FLD 0.14–0.18 (0.17), PCW 0.05–0.07 (0.06), SL 0.46–0.56 (0.55), EL 0.11–0.15 (0.14), ACL 0.45–0.53 (0.52), ML 0.76–0.99 (0.90), PrW 0.41–0.55 (0.50), PSL 0.09–0.14 (0.12), SDL 0.05–0.08 (0.07), PL 0.31–0.41 (0.38), PH 0.23–0.31 (0.31), PW 0.14–0.20 (0.19), PPL 0.17–0.21 (0.21), PPW 0.18–0.26 (0.23), MFL 0.52–0.65 (0.63), MTL 0.39–0.50 (0.48), CI 92–96 (92), SI 80–85 (85), REL 19–23 (21), FLI 25–28 (27), PSI 1.7–2.5 (1.8), MFI 101–113 (101), ACI1 65–70 (68), ACI2 91–98 (95).

Medium-sized species; general body color black to red- or brown-black, with mandibles and appendages lighter, usually dark brown to yellow-brown; setae light brown; mandible with 6 teeth, consisting of 3 distinct apical teeth, a basal tooth, and 2 inner teeth, which are often worn and indistinct; basal margin of mandible relatively straight, without any notch or significant depression; dorsal surface of mandible mostly smooth and shining, with scattered piligerous punctae and a few short basal striae; median lobe of clypeus with pair of very faint vestigial carinulae that diverge toward anterior margin, apex with a short transverse carinula, remainder of clypeus mostly smooth and shiny; posterior extension of clypeus between frontal lobes moderately broad (PCW 0.05-0.07), with subparallel to slightly diverging sides; frontal lobes narrow, not obscuring torular lobes in full-face view; head roughly oval-shaped (CI 92-96), but appearing somewhat tear drop-shaped because of the angled anterior margin of the clypeus and position of eyes; posterior margin of the head flat, never distinctly depressed medially; eye of moderate size (EL 0.11–0.15, REL 19-23), oval-shaped, with 6–8 ommatidia at greatest diameter; face almost completely smooth and shining, with scattered piligerous punctae and a few longitudinal carinulae on gena; scape short (SI 80-85), not reaching posterior margin of head when laid back; scape mostly smooth and shining, with scattered piligerous punctae, and sometimes a few fine striae; flagellum with a distinct 4-segmented antennal club; mesosoma usually mostly smooth and shiny, with some longitudinal carinae in metanotal groove, and a few scattered rugulae on propodeum, but some specimens with more developed sculpture on mesonotum, mesopleuron, and propodeum, consisting of faint carinulae and punctae; promesonotum domed and distinctly bulging upwards above head and propodeum; promesonotal suture usually completely effaced dorsally, but in a few specimens pronotum appears separated from mesonotum; metanotal groove distinct and of moderate depth; propodeal spines short, forming broad triangles (PSL 0.09–0.14, PSI 1.7-2.5); petiole of moderate length (PL/HW 0.52–0.62), node in profile robust and very tall (PH/PL 0.66–0.80), with anterior face longer and more sloping than posterior face, dorsum of node distinctly angled posteriad, and almost reaching a sharp apex; postpetiole in profile subcircular to oval-shaped, somewhat globular, always smaller than petiolar node (PPH/PH 0.70–0.84), dorsum usually with a somewhat distinct longitudinal median lobe; petiole and postpetiole mostly punctate, sometimes with faint rugulae, anterior faces of nodes smooth and shiny; gaster smooth and shiny except scattered piligerous punctae; most of body with moderately long, erect to subdecumbent setae; scapes with subdecumbent to decumbent setae; setae on legs mostly decumbent to appressed, with some suberect setae on femoral venters and coxae.

#### Queen.

Unknown.

#### Male.

Unknown.

#### Biology.

*Stenamma llama* is a cloud forest specialist occurring from approximately 1100 m to 1600 m elevation. All specimens are known from sifted leaf litter collected from the forest floor, except for one, which was found at a cookie bait card. It is a rare species currently known from only one site in Oaxaca, Mexico, and one site in Guatemala. At the Guatemala site, out of 100 mini-Winkler and six maxi-Winkler samples, only six specimens were collected. The completely smooth and shiny integument of *Stenamma llama* suggests that it nests in a relatively wet microhabitat.

#### Comments.

With its mostly smooth sculpture and bulging promesonotum, *Stenamma llama* is a very recognizable species. These characters, along with those in the key and diagnosis, should make it easy to separate from all other *Stenamma* species.

*Stenamma llama*, along with *Stenamma lobinodus* and *Stenamma tiburon*, belongs to the *lobinodus* species group. Diagnostic features of the group are discussed below under *Stenamma lobinodus*.

#### Material examined.

**GUATEMALA:** ***Zacapa***: 2km SE La Unión, 14.94711°N, 89.27677°W, 1550m, 12 May 2009 (LLAMA); **MEXICO:** ***Oaxaca***: 10.8km SW Valle Nacional, 17.68102°N, 96.33026°W, 1120m, 13 Aug 2009 (M. G. Branstetter); 13.2km SW Valle Nacional, 17.65934°N, 96.33426°W, 1360m, 11 Aug 2009 (M. G. Branstetter); 14.8km SSW Valle Nacional, 17.64483°N, 96.33637°W, 1370m, 13 Aug 2009 (M. G. Branstetter).

### Stenamma lobinodus sp. n.

urn:lsid:zoobank.org:act:E63E10BA-80CA-45F6-A305-0DFAF39B9596

http://species-id.net/wiki/Stenamma_lobinodus

**Figure 106.**
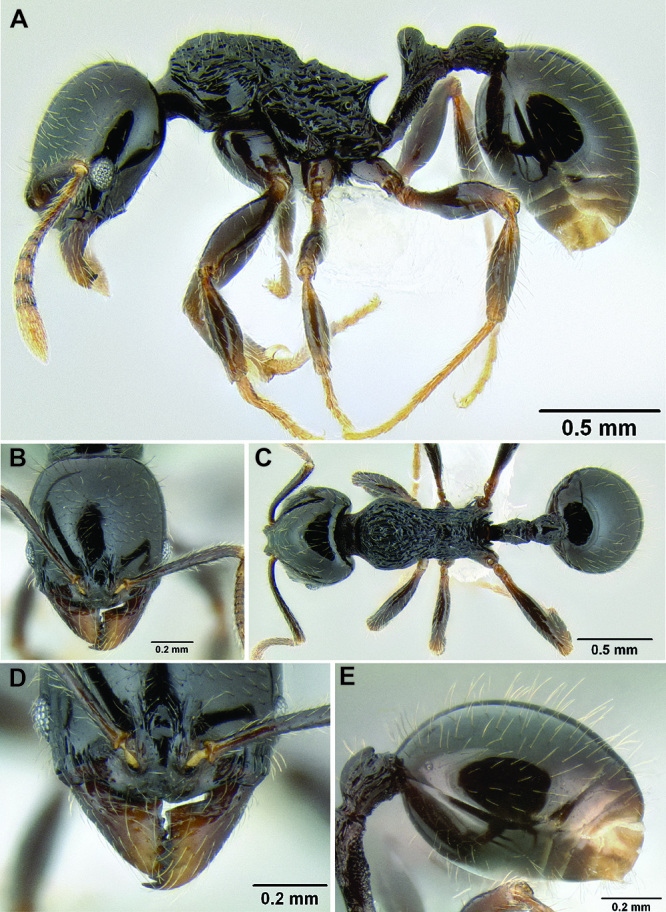
*Stenamma lobinodus* holotype worker (CASENT0605658) **A** Profile **B** Face **C** Dorsum **D **Anterior clypeal margin in anterodorsal view **E** Gaster.

**Figure 107.**
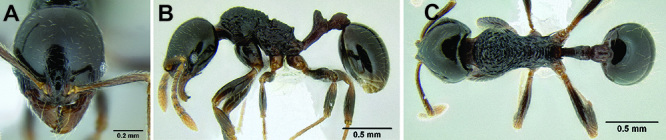
*Stenamma lobinodus* worker variant (CASENT0605571) **A** Face **B** Profile **C** Dorsal.

**Figure 108.**
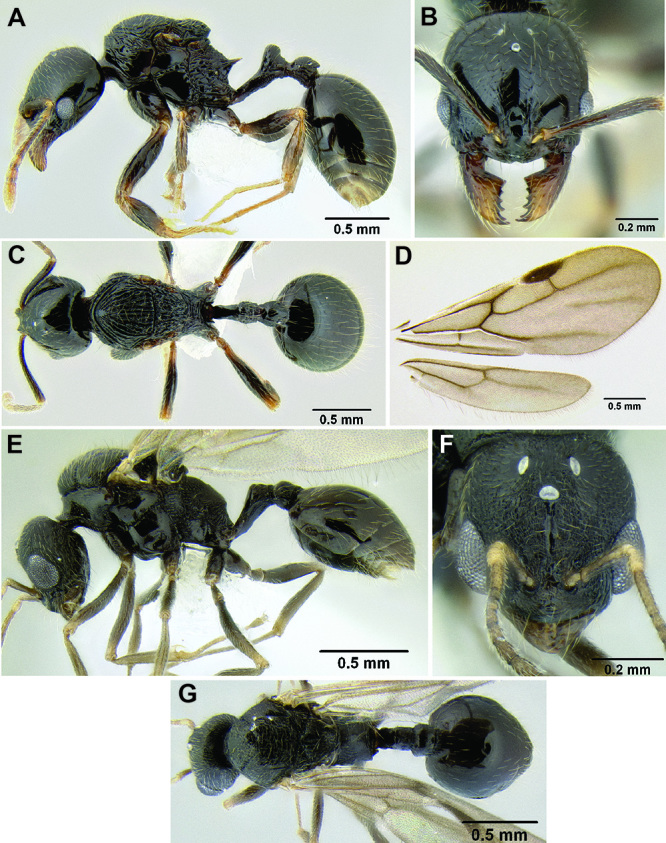
*Stenamma lobinodus* **A** Paratype queen (CASENT0605638), profile **B** Same, face **C** Same, dorsum **D** Queen (CASENT0605697) **E** Male (CASENT0605698), profile **F** Same, face **G** Same, dorsum.

Worker: Figures 106 , 107 ; Queen: Figure 108A–D ; Male: Figure 108E–G ; Map: Figure 105

Stenamma mgb45 Branstetter, 2012: phylogeny.

#### Type material.

*Holotype worker*. MÉXICO: Tamaulipas, El Cielo, 1km NE La Gloria, 23.05060°N, 99.24269°W ±30m, 1570m, 22 Aug 2009 (M. G. Branstetter, collection MGB1450) [USNM, specimen CASENT0605658]. *Paratypes*: same data as holotype [1w, CAS, CASENT0605656], [1w, EAPZ, CASENT0605657], [1w, ECOSCE, CASENT0623336], [1w, FMNH, CASENT0623338]; same data but El Cielo, Joya de Manantiales, 23.00835°N, 99.28511°W ±20m, 1430m, 22 Aug 2009 (M. G. Branstetter, MGB1446) [1w, ICN, CASENT0605641], [1w, INBio, CASENT0605643], [1w, LACM, CASENT0622422], [1w, MGBPC, CASENT0605644], [1w, MCZ, CASENT0623339], [1w, MZSP, CASENT0623340], [1w, UCD, CASENT0623341], [1w, UNAM, CASENT0623342], [1dq, 1w, USNM, CASENT0605638, CASENT0623337], [1w, UVGC, CASENT0623343].

#### Worker diagnosis.

Integument black to brown-black and shining; medium-sized species (see HL, ML, PrW below); anterior clypeal margin with shallow median emargination; basal margin of mandible relatively straight, without notch or substantial depression; face mostly smooth and shiny; mesosoma with coarse, rugose to rugoreticulate sculpture on most surfaces; promesonotum in profile distinctly asymmetrical, with anterior face gently rounded, dorsal surface flattened, and posterior face short, straight and forming a relatively sharp angle with dorsum; petiolar node, generally robust, and distinctly angled posteriad; postpetiolar node in profile with a longitudinal dorsal lobe that projects out posteriad over postpetiole; eye of moderate size (EL 0.11–0.14; REL 18-22), oval-shaped, with 7–8 ommatidia at greatest diameter; propodeal spines ranging from short tubercles to long spines (PSL 0.10–0.22, PSI 1.3-2.6); setae on gastral tergites relatively sparse, long, and mostly suberect; frontal lobes narrow, not obscuring torular lobe in full-face view (FLD 0.13-0.16, FLI 21-25). *Similar species*: *Stenamma diversum*, *Stenamma tico*.

#### Geographic range.

Mexico (Atlantic slope).

#### Worker description.

(10 measured) HL 0.65–0.76 (0.73), HW 0.57–0.71 (0.67), FLD 0.13–0.16 (0.15), PCW 0.03–0.05 (0.04), SL 0.52–0.62 (0.61), EL 0.11–0.14 (0.14), ACL 0.50–0.58 (0.56), ML 0.84–1.03 (0.97), PrW 0.41–0.51 (0.49), PSL 0.10–0.22 (0.20), SDL 0.06–0.09 (0.08), PL 0.35–0.44 (0.42), PH 0.23–0.29 (0.27), PW 0.14–0.18 (0.18), PPL 0.16–0.22 (0.21), PPH 0.16–0.19 (0.18), PPW 0.16–0.21 (0.20), MFL 0.59–0.71 (0.70) MTL 0.47–0.57 (0.55), CI 89–95 (92), SI 85–92 (90), REL 18–22 (21), FLI 21–25 (23), PSI 1.3–2.6 (2.4), PI 58–65 (62) MFI 96–103 (96), ACI1 64–68 (66), ACI2 90–100 (92).

Medium-sized species; general body color black to brown- or red-black, with mandibles and appendages lighter, usually dark brown to yellow-brown; setae golden; mandible with 6 teeth, consisting of 3 distinct apical teeth, a basal tooth, and 2 inner teeth, which are often worn and indistinct; basal margin of mandible relatively straight, without a notch or significant depression; dorsal surface of mandible mostly smooth and shiny, with scattered piligerous punctae and a few short basal striae; median lobe of clypeus with a pair of vestigial longitudinal carinulae that diverge toward the anterior margin, apex with a short transverse carinula, remainder of clypeus mostly smooth and shiny; posterior extension of clypeus between frontal lobes moderately broad (PCW 0.03-0.05), with subparallel to slightly diverging sides; frontal lobes narrow (FLD 0.13–0.16, FLI 21–25), not obscuring torular lobes in full-face view; head roughly oval-shaped (CI 89–95), but appearing somewhat tear drop-shaped because of the angled anterior margin of clypeus and position of eyes; posterior margin of head flat, never distinctly depressed medially; eye of moderate size (EL 0.11–0.14; REL 18-22), oval-shaped, with 7–8 ommatidia at greatest diameter; face almost completely smooth and shiny, with scattered piligerous punctae and few longitudinal carinulae on genae; scape moderately long (SI 85-92), nearly reaching posterior margin of head when laid back; scape surface largely smooth and shiny, with scattered piligerous punctae, and sometimes with a few faint striae; flagellum with distinct 4-segmented antennal club; mesosoma almost entirely with coarse rugae and rugoreticulae; rugae usually with a predominately longitudinal orientation, but in some specimens rugae on mesonotum have a transverse orientation; katepisternum, metapleuron, and lateral portion of pronotum, with a variably sized patch of smooth cuticle; propodeal dorsum and declivity with transverse carinulae; promesonotum in profile distinctly asymmetrical, with anterior face gently rounded, dorsal surface flattened, and posterior face short, straight and forming a relatively sharp angle with dorsum; promesonotal suture indistinct, but usually discernable, especially when the orientation of the pronotal and mesonotal rugae differ; metanotal grove distinct and of moderate depth; propodeal spines present and often relatively long, but sometimes (usually specimens from lower elevations) becoming reduced to short tubercles (PSL 0.10–0.22, PSI 1.3–2.6); petiole in profile relatively long (PH/HW 0.58–0.65) and very distinctive, having a long peduncle and robust node, which is markedly angled posteriad; petiolar node in profile with long sloping anterior face that begins at about middle of peduncle, posterior face shorter, but usually at a similar angle, dorsum of node broadly rounded to subquadrate; postpetiole in profile asymmetrical, with a long sloping anterior face, and a short nearly vertical posterior face, dorsum with a distinct longitudinal lobe that projects out slightly posteriad over postpetiole, lobe in dorsal view attenuating posteriad, giving it a pinched-in appearance; petiole and postpetiole with variable amount of rugae and punctae; anterior faces of nodes mostly smooth and shiny with remaining surfaces punctatorugose; punctae most visible on ventral surfaces; posterior slope of petiolar node sometimes with a median keel; in a few aberrant populations the petiole and postpetiole lack rugae and are almost completely punctate; gaster smooth and shiny, with scattered piligerous punctae; most of body with sparse layer of moderately long standing seate; setae on scape mostly subdecumbent; setae on remaining appendages suberect to decumbent, with longer suberect setae on femoral venters and coxae.

#### Queen description.

(5 measured) HL 0.71–0.77 (0.72), HW 0.67–0.73 (0.67), FLD 0.16–0.17 (0.16), PCW 0.03–0.05 (0.03), SL 0.58–0.63 (0.58), EL 0.17–0.19 (0.17), ACL 0.55–0.60 (0.56), ML 1.03–1.16 (1.03), PrW 0.60–0.65 (0.60), PSL 0.17–0.23 (0.17), SDL 0.08–0.11 (0.09), PL 0.41–0.49 (0.41), PH 0.26–0.31 (0.28), PW 0.19–0.22 (0.19), PPL 0.20–0.24 (0.22), PPH 0.20–0.24 (0.21), PPW 0.23–0.26 (0.23), MFL 0.67–0.74 (0.67), MTL 0.53–0.60 (0.53), CI 93–97 (93), SI 84–89 (87), REL 26–27 (26), FLI 23–24 (24), PSI 1.9–2.5 (1.9), MFI 98–102 (100), ACI1 66–69 (66), ACI2 94–96 (95).

Same as worker except for standard queen modifications and as follows: lateral surfaces of pronotum transversely rugose to rugoreticulate, median area smooth and shiny; mesoscutum longitudinally carinate, with carinae fanning out from middle of anterior margin; mesopleuron mostly smooth and shiny; wing venation as in Figure 108D.

#### Male.

See Figure 108E–G.

#### Biology.

This species is known only from Winkler or Berlese extractions of sifted leaf litter taken from montane, mesophyll forests. Nests have never been collected. It is likely a mid-elevation specialist given its occurrence between 860 to 1620 m.

At the El Cielo reserve in northern Mexico, I found a lone worker that looked like *Stenamma lobinodus* foraging on a mud/clay bank bordering the main road (specimen viewed with a hand lens). Unfortunately the worker escaped and no nest was located. The presence of the worker on a mud bank suggests that *Stenamma lobinodus* may nest in banks like the similar-looking *Stenamma diversum* (see biology above for this species). Both species have almost the same sculpture, color and propodeal spines.

#### Comments.

*Stenamma lobinodus*, *Stenamma llama* and *Stenamma tiburon* constitute the *lobinodus* species group. This group can be diagnosed by the following characters: postpetiole with a distinctive longitudinal dorsal lobe, which sometimes projects posteriad over postpetiole (lobe less discernable in *Stenamma llama*); petiolar node well-developed, and pointing distinctly posteriad, node usually very large in *Stenamma llama* and *Stenamma lobinodus*. *Stenamma lobinodus* and *Stenamma tiburon* also share the feature that the promesonotum in profile is asymmetrical, with a long, rounded anterior face, flat top, and short posterior face. Sister to the *lobinodus* group is *Stenamma pelophilum*. This species does not have the characteristic lobe on the postpetiole that defines this group.

*Stenamma lobinodus* can be separated easily from *Stenamma diversum* and *Stenamma tico* by observing its asymmetrical promesonotum and dorsal postpetiolar lobe. Although it has never been collected in sympatry with either species, it may co-occur with *Stenamma diversum* in Oaxaca, Mexico.

*Stenamma lobinodus* varies significantly across its range. Generally specimens at lower elevations (800–1100 m) are smaller and have shorter propodeal spines than specimens from higher sites. This gradation of sizes is very clear at the El Cielo reserve in Tamaulipas, Mexico, where I collected a series of Winkler samples from 860 to over 1600 m. The specimens from 1300 m and higher have substantially longer spines and more robust petioles. These larger specimens more closely match the ones from the populations in Querétaro.

The populations from Taman in San Luis Potosí and Mirador Grande in Oaxaca are particularly aberrant and I describe them here as variant 1 of *Stenamma lobinodus* (Figure 107). Variant 1 specimens are smaller and have the propodeal spines reduced to small tubercles. Also, the petiole and postpetiole lack distinct rugae and are instead covered in a dense network of foveolae and punctae. Specimens of both populations are from lower elevation (1000 m) and they most closely resemble the low elevation specimens from El Cielo. However, the latter specimens have better developed spines and the petiole and postpetiole are rugose.

I have decided not to split these variants into a separate species because of lack of sympatry between the two forms and the fact that substantial variation exists along elevational gradients and among sites. Also, the Mirador Grande site, which is where most of the aberrant specimens are from, is very far away from the remaining populations. With that said, there is some evidence to suggest that variant 1 does indeed represent a distinct species. First, there is one record of variant 1 from Taman in San Luis Potosí, which is very near the El Madroño site in Querétaro (< 30 km). Second, the intrapopulation variation at El Cielo is substantial, yet it does not encompass the morphology of the aberrant specimens, even at the same elevation. Lastly, preliminary molecular evidence shows that a specimen from Mirador Grande is as divergent from El Madroño and El Cielo specimens as some clearly distinct (and sympatric) sister species pairs. To solve this problem, more collecting should be done in the region of Taman and El Madroño in order to find an area of sympatry or to provide additional fresh specimens for molecular work.

#### Material examined.

**MÉXICO:** ***Oaxaca***: Mirador Grande, 17.89844°N, 96.36253°W, 990m, 14 Aug 2009 (M. G. Branstetter); ***Querétaro***: 3.7km ENE El Madroño, 21.30000°N, 99.08570°W, 1470m, 19 Aug 2009 ( M. G. Branstetter); Estación El Pilón, 21.497°N, 99.168°W, 1100m, 4 Feb 2005 (M. Pérez); ***San Luis*** ***Potosí***: 17.7km W El Naranjo, [ca. 24.490°N, 99.420°W], 980m (A. F. Newton); Taman, 20km SW Tamazunchale, [ca. 21.153°N, 98.947°W], 11 June 1983 (S. & J. Peck); ***Tamaulipas***: El Cielo, 2.5km SSE Alta Cima, 23.03787°N, 99.18941°W, 870m, 24 Aug 2009 (M. G. Branstetter); El Cielo, nr. Alta Cima, 23.06518°N, 99.20433°W, 980m, 21 Aug 2009 (M. Vásquez-Bolaños); El Cielo, 1km NE La Gloria, 23.05060°N, 99.24269°W, 1570m, 22 Aug 2009 (M. G. Branstetter); El Cielo, Joy de Manantiales, 23.000835°N, 99.28511°W, 1430m, 22 Aug 2009 (L. Sáenz); El Cielo, 1.3km NW Joya de Manantiales, 23.02331°N, 99.28830°W, 1620m, 22 Aug 2009 (M. G. Branstetter); nr. Gomez Farias Rancho del Cielo, [ca. 23.063°N, 99.205°W], 1000m, 7 Aug 1983, (S. & J. Peck).

### Stenamma longinoi sp. n.

urn:lsid:zoobank.org:act:D3A56607-85F0-45D9-80C0-8E0DB6EC432D

http://species-id.net/wiki/Stenamma_longinoi

**Figure 109.**
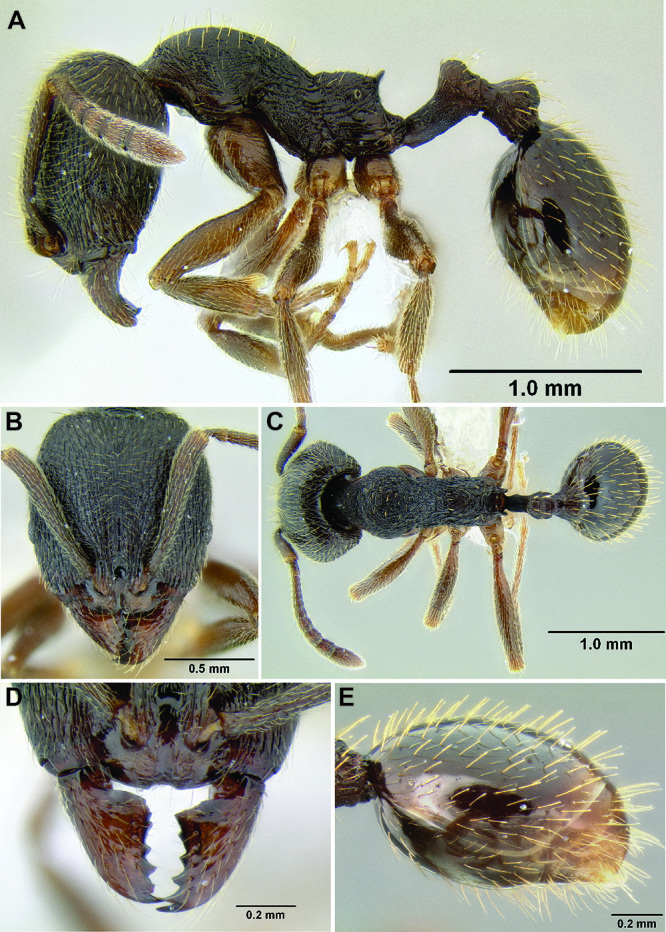
*Stenamma longinoi* **A** Holotype worker (JTLC000007475), profile **B** Same, face **C** Same, dorsum **D** Paratype worker (JTLC000007476), anterior clypeal margin in anterodorsal view **E** Holotype worker (JTLC000007475), gaster.

Worker: Figure 109 ; Map: Figure 105

#### Type material.

*Holotype worker*. MÉXICO: Chiapas, Custepec, 15.72212°N, 92.94428°W ±30m, 1680m, 19 May 2008, mesophyll forest, under epiphytes in treefall (J. Longino, collection JTL6297) [USNM, specimen JTLC000007475]. *Paratypes*: same data as holotype [1w, ECOSCE, JTLC000007476], [1w, MGBPC, CASENT0603913], [1w, MCZ, CASENT0623106].

#### Worker diagnosis.

Integument mostly dark brown to dark red-brown; large-sized species(see HL, ML, PrW below); lateral margin of hypostomal bridge with a projecting quadrate lobe (visible behind mandible in profile); gaster, and most dorsal surfaces, with a layer of thickened suberect to subdecumbent setae, gaster also with sparse layer of thinner appressed setae; mesosoma, waist, and head densely punctate (punctae fainter on head); head and mesosoma with fine rugulae and rugoreticulae, which on the mesosoma merge into the punctae, making them less obvious; eye relatively small (EL 0.13–0.16, REL 13–15), oval-shaped, with 6–7 ommatidia at greatest diameter; anterior clypeal margin with a shallow median emargination; basal margin of mandible straight to slightly sinuous, without a basal notch or depression; propodeal spines short (PSL 0.16–0.19, PSI 1.4–1.6), frontal lobes narrow (FLD 0.24–0.27, FLI 26), not obscuring torular lobes in full-face view. *Similar species*: *Stenamma leptospinum*, *Stenamma manni*, *Stenamma muralla*.

#### Geographic range.

Southern Mexico.

#### Worker description.

(4 measured) HL 1.02–1.13 (1.12), HW 0.92–1.05 (1.01), FLD 0.24–0.27 (0.26), PCW 0.05–0.08 (0.08), SL 0.79–0.85 (0.83), EL 0.13–0.16 (0.13), ACL 0.67–0.74 (0.74), ML 1.29–1.41 (1.36), PSL 0.16–0.19 (0.17), SDL 0.10–0.13 (0.10), PrW 0.57–0.64 (0.61), PL 0.46–0.49 (0.49), PH 0.29–0.32 (0.30), PW 0.22–0.25 (0.24), PPL 0.27–0.30 (0.30), PPH 0.26–0.30 (0.29), PPW 0.28–0.31 (0.30), MFL 0.93–1.03 (1.02), MTL 0.57–0.80 (0.57), CI 91–93 (91), SI 81–88 (82), REL 13–15 (13), FLI 26 (26), PSI 1.4–1.6 (1.6), MFI 99–102 (99), ACI1 64–66 (64), ACI2 84–89 (89).

Large species; general body color dark brown (almost black) to dark red-brown, with appendages and some parts of the gaster lighter brown; setae a rich golden brown; mandible with 6 teeth, middle 2 teeth near basal tooth sometimes worn and indistinct; basal margin of mandible straight to slightly sinuous, without a basal notch or significant depression; mandible mostly smooth and shining with scattered piligerous punctae and striae; anterior clypeal margin with a median emargination; median lobe of clypeus with a pair of faint longitudinal carinulae that diverge anteriorly, area in between carinulae slightly depressed, apex of median lobe with a short transverse carinula, remainder of clypeus mostly smooth and shiny; posterior extension of clypeus between antennal insertions of moderate width (PCW 0.05–0.08), sides slightly hourglass-shaped; frontal lobes narrow (FLD 0.24–0.27, FLI 26), not obscuring torular lobes in full-face view; lateral margin of hypostomal bridge with a projecting quadrate lobe (visible behind mandible in profile); head robust, somewhat heart-shaped (CI 91–93), with posterior margin broadly depressed medially; eye relatively small compared to HW (REL 13–15), but appearing of moderate size (EL 0.13–0.16), with 6–7 ommatidia at greatest diameter, oval-shaped; face densely sculptured with longitudinal carinulae and rugulae, becoming rugoreticulae toward lateral margins, interstitial areas noticeably punctate; scape relatively short (SI 81–88), not reaching posterior margin of head when laid back; scape surface with scattered carinulae and faint piligerous punctae; flagellum with a somewhat indistinct 4-segmented antennal club; mesosoma densely punctate, and with fine rugulae that merge into surrounding punctae; rugulae on anterior half of pronotal dorsum transversely arcuate across surface; rugulae on remainder of promesonotal dorsum longitudinal in orientation; pronotal side completely punctate; rugulae on mesopleuron somewhat reticulate; part of propodeal dorsum and declivity with transverse carinulae; promesonotum in profile low-domed and asymmetrical, with apex occurring anterior of midpoint; metanotal grove somewhat indistinct with anterior margin merging smoothly with promesonotal declivity; propodeal spines well-developed, short (PSL 0.16–0.19, PSI 1.4–1.6); petiole of moderate length and robust (PL/HW 0.47–0.50), node of moderate height (PH/PL 0.62–0.65), broadly domed, and pointing slightly posteriad; postpetiole in profile nearly symmetrical, somewhat bulging, about as high as petiolar node (PPH/PH 0.90–0.96); petiole and postpetiole completely punctate and with a few rugulae on posterior sides of nodes; gaster mostly smooth and shiny, with scattered piligerous punctae; much of dorsal body surface with a layer of stout standing setae; setae on scape thin, dense, and decumbent (almost pubescent); gaster with a short layer of thickened suberect to subdecumbent setae and a very sparse layer of thinner appressed setae; setae on legs mostly appressed, with a few suberect setae on the coxae and femoral venters.

#### Queen.

Unknown.

#### Male.

Unknown.

#### Biology.

This species is known from a single collection event made in mesophyll forest at 1680 m elevation. A few workers were found under epiphytes in a treefall. Over 100 leaf litter samples were collected from the same site and no additional specimens were retrieved. Consequently, this species is likely to be arboreal in its habits.

#### Comments.

*Stenamma longinoi* is similar to *Stenamma leptospinum*, *Stenamma manni*, and *Stenamma muralla*, mainly because all share the lateral hypostomal lobe. However, *Stenamma longinoi* is very distinct and should be easily separated from these other species by its punctate sculpture and thickened setae. Given the diversity of *Stenamma manni* variants (see *Stenamma manni* below), I do find it possible that *Stenamma longinoi* is an arboreal version of *Stenamma manni*. However, molecular data place *Stenamma longinoi* firmly outside of the clade that includes *Stenamma manni* and variants of *Stenamma manni* that are from all over Mexico and Central America (Branstetter unpublished data). Furthermore, *Stenamma longinoi* was collected in sympatry with *Stenamma manni* and no intermediates were found.

#### Material examined.

Known only from the type locality.

### Stenamma manni

Wheeler

http://species-id.net/wiki/Stenamma_manni

**Figure 110.**
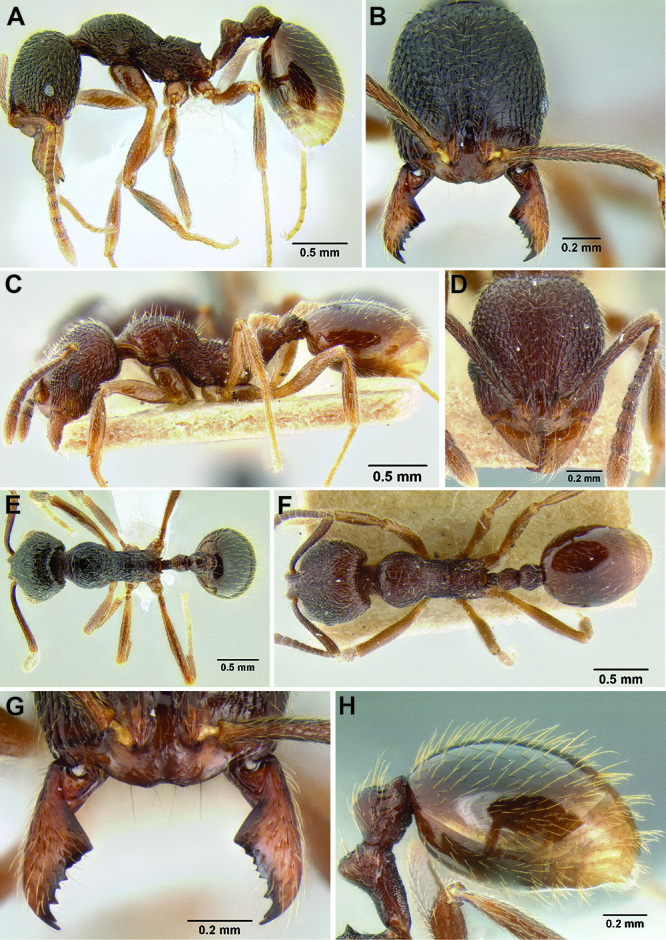
*Stenamma manni* **A, B, E, G, H** Worker variant 1 (CASENT0604893) **C, D, F** Lectotype worker (CASENT0126265).

**Figure 111.**
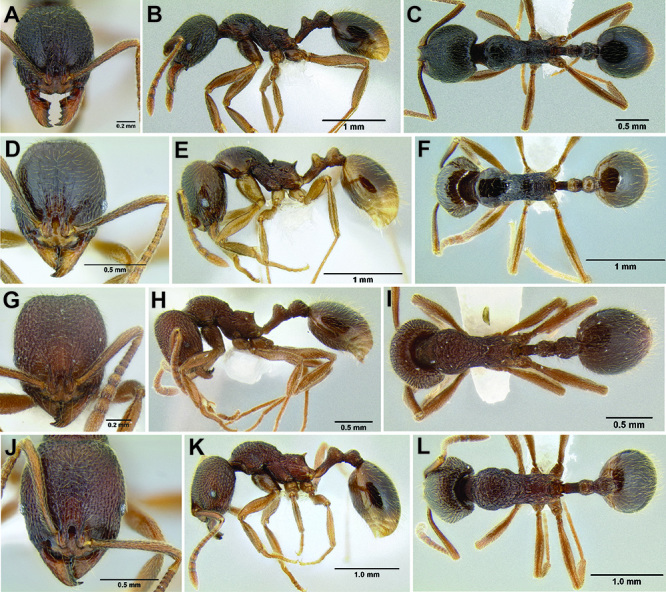
*Stenamma manni* worker variants 1. Face, profile, and dorsal views **A–C** Variant 1 (CASENT0621574) **D–F** Variant 2 (CASENT0605527) **G–I** Variant 3 (CASENT0126280) **J–L** Variant 4 (CASENT0605592).

**Figure 112.**
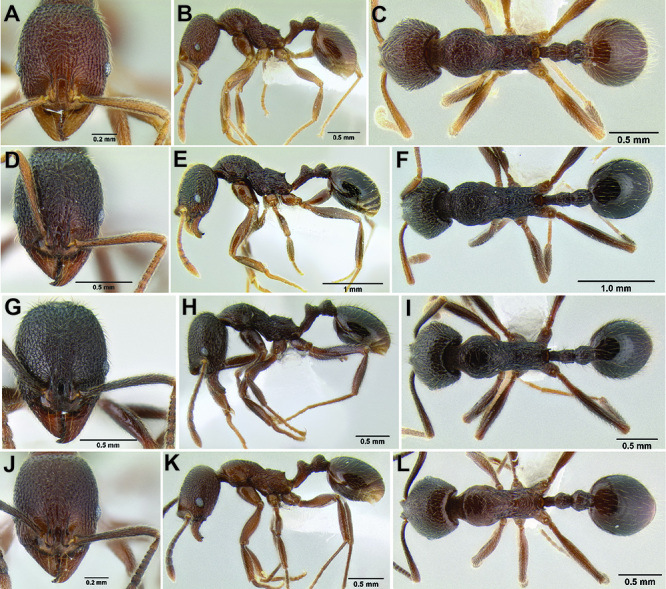
*Stenamma manni* worker variants 2. Face, profile, and dorsal views **A–C** Variant 5 (CASENT0194011) **D–F** Variant 6 (CASENT0606975) **G–I** Variant 7 (CASENT0606002).

**Figure 113.**
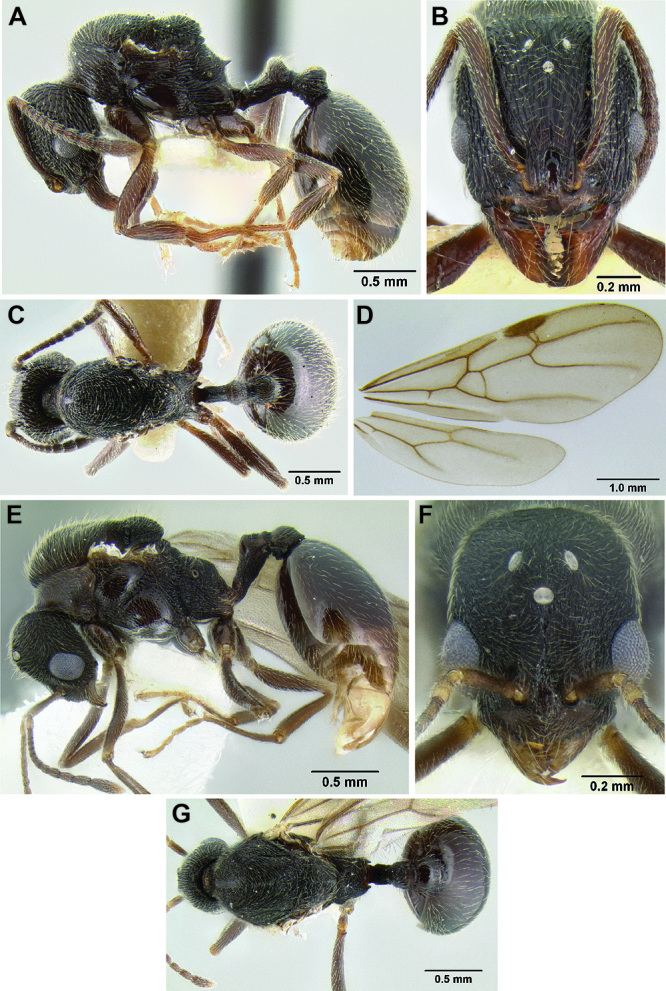
*Stenamma manni* **A** Queen (CASENT0126273), **B** Same, face **C** Same, dorsum **D** Same, wings **E** Male (CASENT0126289), profile **F** Same, face **G** Same, dorsum.

**Figure 114.**
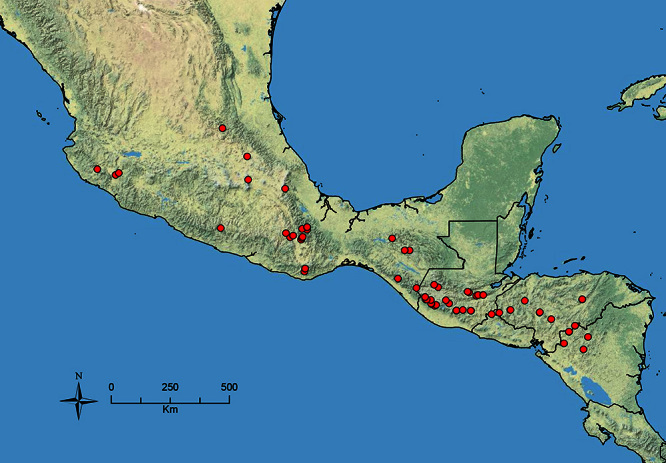
Distribution map of *Stenamma manni*.

Worker: Figures 110- 112 ; Queen: Figure 113A–D ; Male: Figure 113E–G ; Map: Figure 114

Stenamma manni Wheeler, W. M. 1914: 51. Lectotype worker (**here designated)**: MÉXICO, Hidalgo: on the trail between Real del Monte and El Chico, [ca. 20.138°N, 98.673°W], 10000-11000ft [3050–3350m], spring–summer 1913, pine forest, under large stone in damp spot (W. M. Mann) (MZC, Type 2 -3 8672, pin CASENT0126265, specimen furthest from pin) [examined]. Smith, 1962: 35, worker description. Branstetter, 2009: worker images. Branstetter, 2012: phylogeny.
Stenamma mgb28 [variant 4 below] Branstetter 2012: phylogeny.

#### Worker diagnosis.

Note that this species is highly variable. See comments section below discussing population variants. Integument color variable; medium- to large-sized species (see HL, ML, PrW below); lateral apex of hypostomal bridge projecting ventrally as a subquadrate to broadly rounded lobe, which is usually visible behind base of mandible in profile view (sometimes visible only in lateroventral view); propodeal spines tuberculate to short (PSL 0.09–0.19, PSI 1.0–1.6); basal margin of mandible straight; anterior clypeal margin with a single median emargination; face usually completely sculptured, mostly rugoreticulate, with some longitudinal rugulae/carinulae along midline, but sometimes face mostly smooth, with only some longitudinal carinulae; mesosoma usually mostly sculptured with carinae, rugae, rugoreticulae, or punctae, only sometimes with pronotum mostly or completely smooth; eye of moderate size (EL 0.10–0.16, REL 13–21), oval-shaped, with 5–8 ommatidia at greatest diameter; frontal lobes of moderate width (FLD 0.19–0.29, FLI 25–30); first gastral sternite and tergite sometimes punctate. *Similar species*: *Stenamma felixi*, *Stenamma leptospinum*, *Stenamma megamanni*, *Stenamma muralla*.

#### Geographic range.

Mexico to Nicaragua.

#### Worker description.

(25 measured, lectotype in parentheses) HL 0.81–1.13 (0.91), HW 0.70–1.01 (0.78), FLD 0.19–0.27 (0.23), PCW 0.05–0.08 (0.08), SL 0.68–1.04 (0.75), EL 0.10–0.16 (0.14), ACL 0.62–0.88 (0.67), ML 1.05–1.50 (1.17), PrW 0.45–0.64 (0.53), PSL 0.09–0.19 (0.12), SDL 0.08–0.14 (0.10), PL 0.36–0.54 (0.44), PH 0.21–0.32 (0.25), PW 0.17–0.25 (0.21), PPL 0.21–0.33 (0.25), PPH 0.21–0.33 (0.25), PPW 0.22–0.34 (0.26), MFL 0.77–1.28 (0.85), MTL 0.61–0.97 (0.67), CI 82–91 (86), SI 88–109 (96), REL 13–21 (18), FLI 26–30 (29), PSI 1.0–1.6 (1.2), MFI 75–100 (92), ACI1 61–66 (65), ACI2 84–93 (89).

Medium- to large-sized species; general body color highly variable, ranging from mostly black (type population), to red-brown, to brown, to yellow-brown, with appendages lighter, especially at joints and toward extremities, generally brown or orange-brown to yellow-brown; setae golden brown; mandible with 6–7 teeth (usually 6), consisting of 3 distinct apical teeth, a basal tooth, and 2–3 smaller teeth in between, which are often worn and indistinct; basal margin of mandible straight, without a basal notch or depression; mandible mostly smooth, with scattered piligerous punctae, and a variable number of longitudinal striations, mostly at base and on lateral surface; anterior clypeal margin with a shallow median emargination; median lobe of clypeus often with a pair of faint longitudinal carinulae (type population) that diverge toward anterior margin, but sometimes distinct carinulae replaced or hidden by a variable number of irregular striations, apex of lobe usually with a short transverse carinula, remainder of clypeus mostly smooth; posterior extension of clypeus between frontal lobes of moderate width (PCW 0.05–0.08), with sides subparallel; frontal lobes of moderate width (FLD 0.19–0.27, FLI 26–30), never greatly obscuring torular lobes in full-face view; lateral apex of hypostomal bridge projecting ventrally as a subquadrate to broadly rounded lobe, which is usually visible behind base of mandible in profile view (reduced in type population; sometimes visible only in lateroventral view); head usually roughly oval-shaped (type population), but sometimes slightly elongate, or more often broad, becoming slightly heart-shaped (CI 82–91), posterior margin slightly to distinctly depressed medially; eye of moderate size (EL 0.10–0.16, REL 13–21), oval-shaped, with 5–8 ommatidia at greatest diameter; face usually completely sculptured, mostly rugoreticulate (rarely completely), with some longitudinal rugulae/carinulae along midline extending from frontal lobes to posterior margin, coarseness of sculpture variable (average in type population); face rarely mostly smooth, with only some longitudinal carinulae along midline and on gena; scape usually of moderate length (type population), but sometimes relatively long and slender (SI 88–109), scape when laid back reaching and often surpassing posterior margin of head; scape surface variable, usually with scattered piligerous punctae and some carinulae (type population), but sometimes scape more smooth with carinulae reduced, or scape more robust, with carinulae coarser; flagellum with distinct 4-segmented antennal club; mesosoma sculpture highly variable, usually completely sculptured, without large patches of smooth cuticle, but sometimes pronotum mostly to completely effaced; dorsum of promesonotum carinulate (type population), rugose, or rugoreticulate (often with punctae), almost always with longitudinal orientation (some aberrant specimens with transverse orientation); side of pronotum carinulate (type population), rugulose, or punctate; mesopleuron and side of propodeum punctate to rugulose-punctate, generally with more rugulae on the propodeum; dorsum and declivity of propodeum with transverse carinulae; promesonotum in profile low-domed, and usually slightly asymmetrical, with apex shifted anterior of midpoint, and anterior face steeper than posterior face (some populations roughly symmetrical); metanotal groove usually well-demarcated, width and depth variable (average in type population); propodeal spines tuberculate to short (PSL 0.09–0.19, PSI 1.0–1.6); petiole in profile appearing average (type population) to slightly elongate (PL/HW 0.48–0.62), with peduncle usually thick and robust; petiolar node in profile usually of moderate size (PH/PL 0.51–0.66), with a broadly rounded dorsum that points vertical to slightly posteriad, rarely pointing distinctly posteriad; node sometimes somewhat compressed anteroposteriorly; posterior margin of petiole in profile sometimes distinctly bent downwards, creating a slight concavity below node; postpetiolar node in profile similar in size to petiolar node (type population) or bulging (PPH/PH 0.79–1.05, PW/PPW 0.70–0.86), shape of node subcircular to asymmetrical (slightly asymmetrical in type population); petiole and postpetiole usually mostly punctate, with anterior faces of nodes variably smooth and shiny (type population), sometimes nodes with rugulae, or rarely rugoreticulae; gaster usually smooth and shiny, within scattered piligerous punctae, but sometimes (mostly northern populations in drier habitats) first gastral tergite and sternite lightly to strongly punctate; pilosity highly variable, pilosity on gastral dorsum usually clearly bilayered, with a layer of longer suberect setae, and a layer of shorter decumbent setae, but length and density of each layer variable, lower layer sometimes very dense (almost pubescent), or somewhat sparse and more subdecumbent, causing it to blend in with upper layer, rarely upper layer very long and more dense (type population average), gastral setae never greatly strongly thickened; setae on scapes uniformly suberect to subdecumbent, never with a separate layer of longer suberect setae; setae on legs decumbent to appressed, with longer suberect setae on femoral venters and coxae.

#### Queen description.

(7 measured) HL 0.92–1.13 (0.98), HW 0.81–1.01 (0.87), FLD 0.23–0.30 (0.25), PCW 0.07–0.10 (0.07), SL 0.81–1.00 (0.82), EL 0.23–0.27 (0.25), ACL 0.72–0.87 (0.75), ML 1.38–1.69 (1.48), PrW 0.82–1.01 (0.82), PSL 0.15–0.29 (0.21), SDL 0.13–0.16 (0.13), PL 0.52–0.68 (0.54), PH 0.28–0.36 (0.29), PW 0.23–0.32 (0.23), PPL 0.27–0.37 (0.28), PPH 0.27–0.39 (0.27), PPW 0.31–0.42 (0.32), MFL 0.98–1.30 (0.99), MTL 0.78–1.01 (0.79), CI 87–92 (89), SI 87–105 (94), REL 26–28 (28), FLI 28–30 (29), PSI 1.1–2.1 (1.6), MFI 78–95 (87), ACI1 60–66 (63), ACI2 83–91 (91).

Same as worker except for standard queen modifications and as follows (comparing queen and worker of type population form only; queen from Rancho Somecla): pronotum with transverse carinulae; mesoscutum and scutellum longitudinally carinulate; propodeum with transverse carinulae that wrap around entire surface; katepisternum mostly smooth; lower layer of setae on gastral dorsum denser; wing venation as in Figure 113D (cell underneath stigma probably aberrant, not present in other *Stenamma manni* queens).

#### Male.

See Figure 113E–G. Note that the male is from the locality Omilteme in Guerrero, Mexico, not the type locality. Males show some variation across the range of the species.

#### Biology.

This species is one of the largest and most conspicuous species of *Stenamma*, which is probably why it was the first Middle American species to be described. As defined here, *Stenamma manni* occurs from 1200–3700 m elevation, but it seems to be most common between 2000–2500 m. It occurs in wet montane forests like cloud forest, and drier, more seasonal habitats, such as oak woodland. Specimens have been collected in leaf litter samples, at bait cards, in Malaise traps, and by general searching. I have found nests in logs, in the leaf litter, under rocks, and in the ground. In Central America, *Stenamma manni* is commonly found at the edge of cloud forest in logs and in the ground under logs. All *Stenamma manni* nests tend to be very large, with hundreds to perhaps over a thousand workers (a complete colony census has not been carried out). Nests usually have brood, alates, and a single egg-laying queen. *Stenamma manni* is one of the most common *Stenamma* species to be found at bait cards, suggesting that they are active epigeic foragers. All foragers I have observed have been solitary.

#### Comments.

*Stenamma manni* should be separable from most similar species by the combination of presence of a lateral hypostomal lobe; presence of tuberculate to short propodeal spines; and large body size. However, it should be evident from the worker description above that *Stenamma manni*, as circumscribed here, is highly variable, and thus difficult to characterize in a very satisfactory way. Indeed, the *manni* complex, with all of its different variants, is a taxonomic nightmare. Nearly every population has unique, often distinctive features, and there is no evidence of sympatry among forms. It is also common to have populations that are intermediate in phenotype between variants. Because of this diversity, I have united most forms, even if very distinct, into one polytypic species. I describe many variants below, providing distinguishing characters for each and approximate geographic ranges. This should help in identification of *Stenamma manni* as I have defined it, and will provide guidelines for future work on the complex.

One *Stenamma manni* -like variant that I did recognize as a distinct species is *Stenamma megamanni*. This species occurs from Chiapas, Mexico to Nicaragua and is sympatric with *Stenamma manni* at many sites. However, it is a variable species and difficult to recognize from *Stenamma manni* at a large geographic scale. Within the range where both species occur, I often find two forms in sympatry. What I am calling *Stenamma manni* always has lighter body color (usually dark red-brown), a smaller eye (usually 5–6 ommatidia at greatest diameter), and a larger, more bulging postpetiole. *Stenamma megamanni*, in contrast, is always black, has a larger eye (8 or more ommatidia at greatest diameter), and usually has the postpetiole similar in size to the petiolar node. In addition, there seems to be some ecological separation between the species. Even though *Stenamma megamanni* does occur at high elevation and in leaf litter, I commonly find it nesting at lower elevations in riparian areas. I have found nests both in clay banks and under rocks along streams. I have never found *Stenamma manni* in these environments. It could be that *Stenamma megamanni* is an ecomorph of *Stenamma manni*, but I have found both species together in samples of leaf litter. Molecular phylogenetic data so far show that *Stenamma megamanni* forms a clade nested within the larger *Stenamma manni* complex. My current hypothesis is that *Stenamma manni* and possibly *Stenamma megamanni*, include more than one species, making it difficult to adequately separate each at a wide regional scale.

As a preface to describing each variant of *Stenamma manni*, I have noticed several phenotypic trends within the complex that correlate with habitat. Populations from drier areas, especially those from central and western Mexico, tend to be lighter in color, have more developed sculpturing, and have denser pilosity, with the lower layer of gastral pilosity often pubescent. In contrast, specimens from wet forest environments are often shinier and less sculptured and have sparser pilosity. They also tend to be darker in color, but not always.

Characteristics of the type population (Figure 110C, D, F) are indicated within the worker and queen descriptions (see parenthetical comments). It is important to note that although these specimens now appear brown in color, the original description says that they were black, with the tarsi, leg joints, and tips of the mandibles and antennae dark red. Overall, these specimens seem to be rather average looking in size, sculpture, and pilosity, compared with many other *Stenamma manni* populations. Only the eyes seem somewhat large relative to head size. Most other *Stenamma manni* populations have one or more characters exaggerated in some way. Another important observation is that these specimens were collected from between 3050–3350 m under a rock in pine forest. This is very high for tropical ants in general, and is one of the highest records for *Stenamma*. The only report of MAC *Stenamma* from a higher elevation is from 3700 m at the locality Rancho Somecla on Pico de Orizaba in Veracruz, Mexico. These specimens are also *Stenamma manni* and they are the most similar in morphology to the type population. I should comment that in color and eye size, the type population might appear more similar to *Stenamma megamanni* than some of the variants below. However, specimens from the type population are much smaller than *Stenamma megamanni* and the sculpture and pilosity are different. Also, I see intermediate forms within Mexico that seem to connect the type population with the other variants.

Variant 1 (Figure 110A, B, E, G, H, 111A–C) is the form that co-occurs with *Stenamma megamanni* in Central America. It has been collected from Chiapas, Mexico, to Nicaragua, and as mentioned above, can be distinguished by its dark red-brown coloration, smaller eye, and bulging postpetiole, which is usually distinctly larger than the petiolar node in profile view. It occurs almost exclusively above 2000 m. Some populations (e.g. Huitepec) have extremely large workers with allometrically enlarged scapes and metafemurs. Compared to the type population, these larger specimens look like giants. Among populations of this variant within Central America, there is considerable sculpture and size variation.

Variant 2 (Figure 111D–F) is known only from the Atlantic slope of the Sierra Juarez Mountains between Oaxaca and Valle Nacional in Oaxaca, Mexico. All collections were above 1650 m in cloud forest habitat. I tend to think of this variant as a high elevation, wet forest-adapted version of *Stenamma manni*. Variant 2 is characterized by the following: general body color brown to yellow-brown, mottled; face mostly smooth and shining, with only some faint carinulae extending back from frontal lobes; pronotum almost completely smooth and shiny; scape slender, somewhat elongate (SI 102–106); metafemur relatively elongate (MFI 82–83); propodeal spines well developed, short (PSL 0.13–0.17, PSI 1.4–1.6); anterodorsal margin of propodeum forming a distinct welt, causing the metanotal groove to appear deep; petiolar and postpetiolar nodes somewhat compressed anteroposteriorly; postpetiolar node enlarged, somewhat bulging; gastral pilosity indistinctly bilayered, with lower subdecumbent layer of setae sparse. The gestalt of this variant is most similar to variant 1. Both have the postpetiole more bulging, and the scape and metafemur longer. The shape of the propodeum in profile is also very similar. Confirming the similarity, molecular phylogenetic data show variant 2 being more closely related to a specimen of variant 1 from Chiapas, Mexico, than to specimens further north in Mexico (Branstetter unpublished data). It is important to note that this variant occurs in sympatry with *Stenamma leptospinum*. Intriguingly, both share the anteroposteriorly compressed petiolar and postpetiolar nodes.

Variant 3 (Figure 111G–I) occurs at Nevado de Colima in Jalisco, Mexico, with similar forms at other nearby sites. It seems to be a version of *Stenamma manni* that is adapted to drier habitats. It is characterized by the following: eye somewhat small, with 6 ommatidia at greatest diameter; dorsum of promesonotum rugoreticulate; side of mesosoma strongly punctate; petiole and postpetiole completely sculptured, mostly punctate, with rugoreticulae on dorsal surfaces of nodes; posterior portion of petiole bent downward, creating a small concavity under node; first gastral sternite strongly punctate; first gastral tergite punctate, but punctae broader, more like small dents, and less dense than sternal punctae; pilosity on gastral dorsum distinctly bilayered, with a layer of long suberect setae, and a layer of very dense (almost pubescent) decumbent setae, dorsum of petiolar and postpetiolar nodes with similar pilosity. There is some evidence for sympatry at Nevado de Colima. Variant 3 was collected at 2070 m. A few specimens of a similar *Stenamma manni* form were collected at 2440 m (same label as variant 3, but different elevation). These specimens do not have distinct rugoreticulae and the dense pubescence, but seem otherwise similar. Because of the difference in elevation between the specimens, the paucity of material, and the existence of intermediate specimens at other sites, I do not consider this potential sympatry as sufficient evidence for calling variant 3 a new species. However, Nevado de Colima would be a good site to visit to further study species boundaries in this complex.

Variant 4 (Figure 111J–L) is known only from Pinal de Amoles, Querétaro, Mexico and is similar to variant 3. It could be a mesic forest version of variant 3, as it occurs in wetter oak-pine forest on the Atlantic slope of the Sierra Gorda. It is characterized by the following: general body color dark red-brown to brown; head noticeably broad and thick; eye small, with 5–6 ommatidia at greatest diameter; dorsum of promesonotum rugoreticulate-punctate, but rugoreticulae poorly developed on middle of dorsum, strongest on humeri; side of mesosoma strongly punctate; propodeal spines well-developed, somewhat robust, and distinctively shaped, being broad at the base and then curving outward, appearing somewhat hook-like; petiole noticeably elongate and bent downward at posterior margin; petiolar node in profile broad and subconical, reaching a defined apex; petiole and postpetiole almost completely punctate; first gastral sternite and tergite lightly punctate; pilosity on gastral dorsum somewhat dense, and clearly bilayered, with a layer of longer suberect to subdecumbent setae, and a layer of decumbent setae, both layers similar in density. Specimens with intermediate morphology between variant 3 and 4 occur in Oaxaca on the western, drier side of the state. Among these specimens there is variation in the development of punctae and pubescence on the gaster, which seems to be clearly linked with elevation. One population from 2350 m (15.5km NE Oaxaca) has no punctae on the first gastral sternite and reduced gastral pilosity. In all other respects it is identical to populations from lower elevations within the state.

Variant 5 (Figure 112A–C) is known from a single collection in Jalisco, Mexico. It is characterized by two main features: pronotal dorsum and face densely rugoreticulate; pilosity on most of dorsal surface of mesosoma, waist, and gaster very dense, long, and flexuous. This variant is probably most similar to variant 3.

Variant 6 (Figure 112D–F) is known from several sites in Guatemala (Cerro Carmona, Salama, Guatemala City), and Honduras (PN Celaque), ranging from 1500–2000 m approximately. It has the following distinctive features: head oval-shaped, somewhat narrow; pronotal dorsum rugoreticulate; side of pronotum punctate; body in profile appearing somewhat elongate and gracile; petiole long and gracile, node asymmetrical, with a long sloping anterior face and a short posterior face, dorsum usually pointing distinctly posteriad; postpetiole somewhat smaller than petiolar node, not bulging. I suspect this variant could be a distinct species. It occurs in sympatry with variant 1 of *Stenamma manni* at Cerro Carmona and has been collected near *Stenamma megamanni*. However, it is very rare (only ten specimens known), and no nests or queens have been found. Molecular phylogenetic data infer it to be closely related to variant 1 and *Stenamma megamanni*. More sampling is needed to test its status.

Variant 7 (Figure 112G–I) is known only from RN Datanlí El Diablo in Nicaragua. It is similar to variant 6, except as follows: pronotal sculpture mostly longitudinally carinulate, with a patch of smooth cuticle in middle of dorsum; pilosity more dense; setae on legs noticeably thickened and subdecumbent (best observed in dorsal view).

Variant 8 (Figure 112J–L) is known from two specimens collected at almost 3000 m at the Sendero Ecologico La Maceta locality in Guatemala. It has the following distinguishing features: eye large, with 9 ommatidia at greatest diameter; pronotum mostly smooth, with some transverse carinulae; postpetiole similar in size to petiolar node; gastral pilosity sparse, and weekly bilayered. This variant is most similar to the type population, in terms of sculpture, body and eye size. Unfortunately, neither specimens from the type population, nor this variant have been included in phylogenetic analyses, so it is unclear how they are related. A few faded specimens from Tajumulco, Guatemala look similar.

#### Material examined.

**EL SALVADOR: *Santa Ana***: Hacienda Montecristo, 23km NE Metapan, [ca. 14.406°N, 89.372°W], 2300m, 8 May 1871 (S. B. Peck); **GUATEMALA:** ***Baja Verapaz***: 4km SSE Purulhá, 15.20522°N, 90.22198°W, 2100m, 20 Sep 2008 (M. G. Branstetter); 16.5km N Salama, [ca. 15.2350°N, 90.2833°W], 1660m, 23 May 1991 (R. S. Anderson); ***Chimaltenango***: Finca Chincharas, Rincón Suizo, 14.79775°N, 90.98380°W, 2490m, 17 Sep 2008 (R. S. Anderson); 3.8km NNE Tecpan, 14.79694°N, 90.98171°W, 2500m, 17 Sep 2008 (M. G. Branstetter);***El Progreso***: Cerro Pinalón, 15.08389°N, 89.94456°W, 2560m, 30 Apr 2009 (LLAMA); 20km N Estancia de la Virgen, [ca. 15.1141°N, 89.8833°W], 1850m, 8 Jun 1991 (R. S. Anderson); ***Guatemala***: Guatemala City, 14.55871°N, 90.46275°W, 1800m, 5 Jul 2007 (R. S. Anderson); ***Huehuetenango***: La Capellania, 15.39867°N, 91.40363°W, 3050m, 14 Sep 2008 (R. S. Anderson); Sendero Ecologico La Maceta, 15.48915°N, 91.55492°W, 2950m, 14 Sep 2008 (R. S. Anderson); ***Jalapa***: 5km ENE Mataquescuintla, 14.53355°N, 90.14323°W, 2550m, 5 Jul 2007 (R. S. Anderson); Miramundo, Pino Dulce, 14.53388°N, 90.15236°W, 2300m, 18 Sep 2008 (R. S. Anderson); ***Quetzaltenango***: Aldea Las Nubes, Volcán Chicabal, 14.80086°N, 91.66803°W, 2280m, 12 Sep 2008 (M. G. Branstetter); nr Roble Grande, 14.92719°N, 91.68271°W, 2750m, 11 Sep 2008 (R. S. Anderson); 11.5km ESE San Marcos, 14.92719°N, 91.68271°W, 2800m, 11 Sep 2008 (M. G. Branstetter); 1.7km SW Santa María, 14.71686°N, 91.53481°W, 1515m, 13 Sep 2008 (L. Sáenz); Volcán Chicabal, 14.78720°N, 91.65839°W, 2700m, 12 Sep 2008 (M. G. Branstetter); 12km SE Zunil, Fuentes Georginas, 14.7488°N, 91.4800°W, 2460m, 27 May 1991 (R. S. Anderson); ***Quiché***: 2.9km SSE Chichicastenango, 14.91861°N, 91.10449°W, 2000m, 17 Sep 2008 (L. Sáenz); ***San Marcos***: 6.3km WSW San Marcos, 14.94686°N, 91.83771°W, 2620m, 11 Sep 2008 (M. G. Branstetter); 9.8km WSW San Marcos, 14.94427°N, 91.87990°W, 1600m, 11 Sep 2008 (M. G. Branstetter); Tajumulco, [ca. 15.044°N, 91.901°W], 2960m, Oct 1934 (F. X. Williams); ***Sacatepéquez***: 5km SE Antigua, 14.54124°N, 90.71026°W, 1740m, 12 Jun 2009 (LLAMA); Cerro Carmona, Finca El Pilar, 14.54115°N, 90.70483°W, 1980m, 9 Sep 2008 (R. S. Anderson); ***Suchitepéquez***: 5km SE Antigua, 14.53581°N, 90.69430°W, 2150m, 10 Jun 2009 (LLAMA); ***Zacapa***: 14km NNE Teculután, 15.11440°N, 89.68047°W, 2270m, 6 Jul 2007 (6 Jul 2007); **HONDURAS: *Comayagua***: 10km E Comayagua, 14.45973°N, 87.54609°W, 2000m, 15 May 2010 (LLAMA); 12km ENE Comayagua, 14.48045°N, 87.53258°W, 2140m, 15 May 2010 (LLAMA); ***Francisco Morazán***: PN La Tigra, 2.6km SW San Juancito, 14.21778°N, 87.09101°W, 1870m, 25 Sep 2008 (M. G. Branstetter); ***Lempira***: PN Celaque, 7.3km SW Graçias, 14.56370°N, 88.64923°W, 1530m, 30 Sep 2008 (M. G. Branstetter); 8.3km SW Graçias, 14.56132°N, 88.65768°W, 1860m, 30 Sep 2008 (M. G. Branstetter); ***Ocotepeque***: 13km E Nueva Ocotepeque, 14.45697°N, 89.06849°W, 2200m, 25 May 2010 (LLAMA); ***Olancho***: 11km N Catacamas, 14.94838°N, 85.91439°W, 2000m, 12 May 2010 (LLAMA); 12km N Catacamas, 14.95787°N, 85.91673°W, 2320m, 9 May 2010 (LLAMA); ***Santa Barbara***: 15km SE Santa Barbara, [ca. 14.906°N, 88.100°W], 24 Aug 1991 (S. & J. Peck); **MÉXICO: *Chiapas***: Cerro Huitepec (Pico), ca. 5km W San Cristobal, [ca. 16.7500°N, 92.6802°W, 2750m, 18 Sep 1991 (R. S. Anderson); 5km NNW Coapilla, 17.18355°N, 93.15222°W, 1915m, 26 May 2008 (LLAMA); 4km E Custepec, 15.71294°N, 92.92761°W, 2180m, 23 May 2008 (M. G. Branstetter); 2km SE Custepec, 15.72188°N, 92.93677°W, 1900m, 19 May 2008 (R. S. Anderson); Huitepec, S. Cristóbal, 16.75181°N, 92.68270°W, 2480m, 29 May 2008 (LLAMA); 7.4km SSW Motozintla de Mendoza, 15.3667°N, 92.2333°W, 2000m, 21 Sep 1992 (R. S. Anderson); 15km San Cristóbal, 16.74707°N, 92.49011°W, 2500m, 29 May 2008 (LLAMA); ***Colima***: 19km NNE Comala, 19.482°N, 103.683°W, 1650m, 25 Dec 1987 (P. S. Ward);***Guerrero***: vic. Omilteme, [ca. 17.555°N, 99.687°W], 2400m, 29–30 Jul 1965 (Cornell Univ. Mexico Field Party);***Jalisco***: 10km S Autlán, 19.6833°N, 104.3830°W, 1600m, 20 Dec 1987 (P. S. Ward); E slope Nevado de Colima, [ca. 19.552°N, 103.559°W], 2400m, 21 Sep 1973 (A. F. Newton); ***Hidalgo***: between Real del Monte and El Chico, [ca. 20.138°N, 98.673°W], 3050m, Mar–Aug 1913 (W. M. Mann); ***Oaxaca***: 9.4km SE Asunción Nochixtlan, 17.37632°N, 97.19976°W, 2240m, 10 Aug 2009 (M. G. Branstetter); 33km S Ixtlan de Juarez, [ca. 17.204°N, 96.589°W], 2400m, 10 Aug 1973 (A. F. Newton); 10.6km N Jct 190/135 (on 135), [ca. 17.282°N, 96.929°W], 1920m, 21 Jul 1987 (R. S. Anderson); La Herradura, [ca. 17.221°N, 97.042°W], 1959 (C. Gans); 14km NE Oaxaca, km 10 Mex. 175, 17.145°N, 96.622°W, 1890m, 20 Aug 1973 (A. F. Newton); 15.5km NE Oaxaca, 17.23695°N, 96.56533°W, 2350m, 11 Aug 2009 (M. G. Branstetter); 14.8km SSW Valle Nacional 17.64483°N, 96.33637°W, 1370m, 13 Aug 2009 (M. G. Branstetter); 22.4km SW Valle Nacional, 17.59112°N, 96.39113°W, 1990m, 13 Aug 2009 (M. G. Branstetter); 32km SW Valle Nacional, Km85, [ca. 17.5046°N, 96.3897°W], 1650m, 26 Jul 1992 (R. S. Anderson); 40km SW Valle Nacional, Km93, [ca. 17.5278°N, 96.5834°W], 1900m, 26 Jul 1992 (R. S. Anderson); 5.1km S Suchixtepec, [ca. 16.0833°N, 96.4667°W], 2150m, 25 Jul 1992 (R. S. Anderson); 30.6km S Suchixtepec, [ca. 15.976°N, 96.498°W], 12 Jul 1987 (R. S. Anderson); 47.5km SW Valle Nacional, Km100.5, [ca. 17.350°N, 96.5333°W], 2125m, 26 Jul 1992 (R. S. Anderson); ***Puebla***: H’way 190, E Río Frío, [ca. 19.310°N, 98.638°W], 2900m, 7 Aug 1965 (Cornell Univ. Mexico Field Party); ***Querétaro***: 1.9km NE Pinal de Amoles, 21.14974°N, 99.61576°W, 2250m, 18 Aug 2009 (M. G. Branstetter); ***Veracruz***: Rancho Somecla, Pico Orizaba, [ca. 18.987°N, 97.227°W], 3700m, 24 Aug 1953 (E. O. Wilson); **NICARAGUA: *Jinotega***: RN Cerro Kilambé, 13.56941°N, 85.69479°W, 1540m, 24 May 2011; RN Datanlí El Diablo, 13.10449°N, 85.86774°W, 1400m, 18 May 2011 (LLAMA); ***Madriz***: Apante, 9km S Somoto, 13.40481°N, 86.57944°W, 1550m, 22 Apr 2011 (M. G. Branstetter); 1.9km SE Las Sabanas, 13.33012°N, 86.60723°W, 1650m, 23 Apr 2011 (M. G. Branstetter); 16km S Somoto, 13.32726°N, 86.61001°W, 1730m, 23 Apr 2011 (R. S. Anderson); ***Nueva Segovia***: Cerro Mogotón, 10km NNE Mozonte, 13.75599°N, 86.42062°W, 24 Apr 2011 (M. G. Branstetter); 9km NW Jalapa, 13.98271°N, 86.18933°W, 1700m, 28 May 2011; 14km NNE Ocotal, 13.75409°N, 86.42094°W, 1900m, 24 Apr 11 (R. S. Anderson).

### Stenamma maximon sp. n.

urn:lsid:zoobank.org:act:ED4A6E0D-05EF-4954-921E-9375046E8F0F

http://species-id.net/wiki/Stenamma_maximon

**Figure 115.**
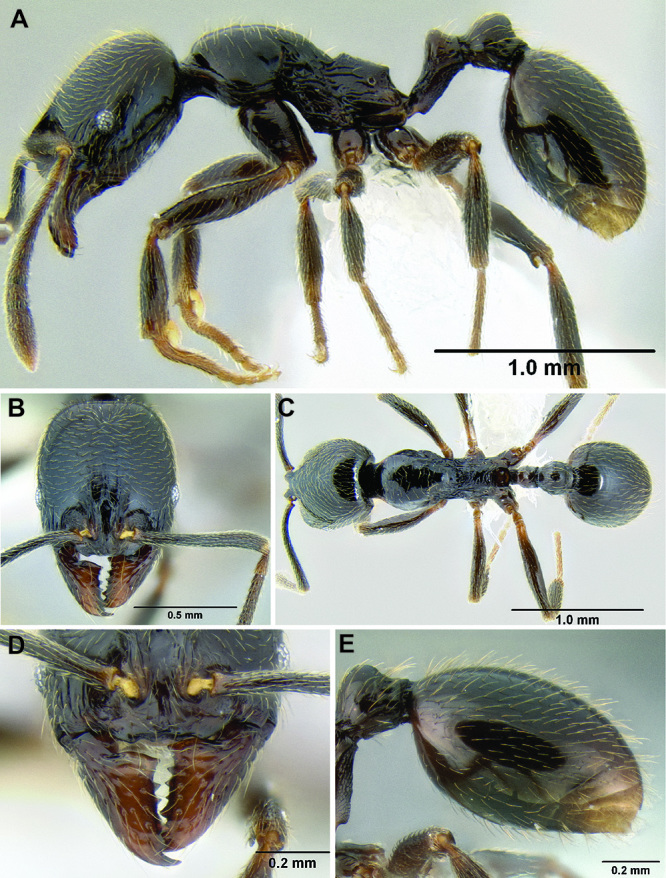
*Stenamma maximon* holotype worker (CASENT0604875) **A** Profile **B** Face **C** Dorsum **D **Anterior clypeal margin in anterodorsal view **E** Gaster.

**Figure 116.**
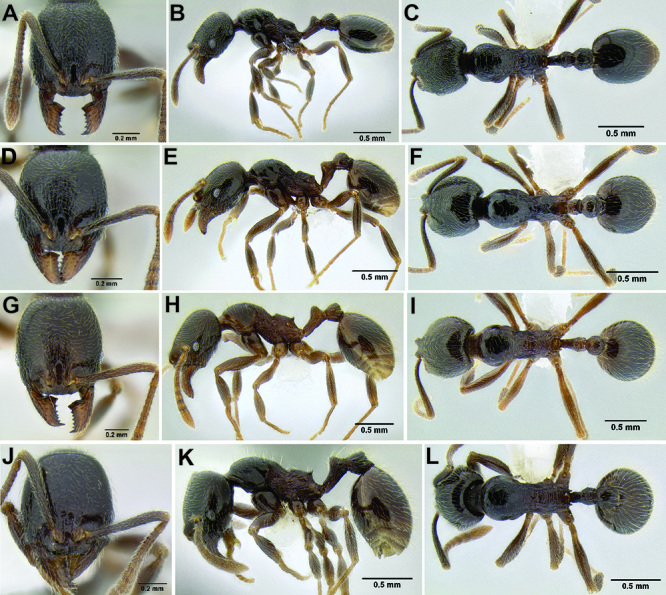
*Stenamma maximon* worker variants. Face, profile, and dorsal views **A–C** Variant 1 (CASENT0605136) **D–F** Type form (CASENT0605089) **G–I** Variant 2 (CASENT0605448) **J–L** Variant 3 (CASENT0623062).

**Figure 117.**
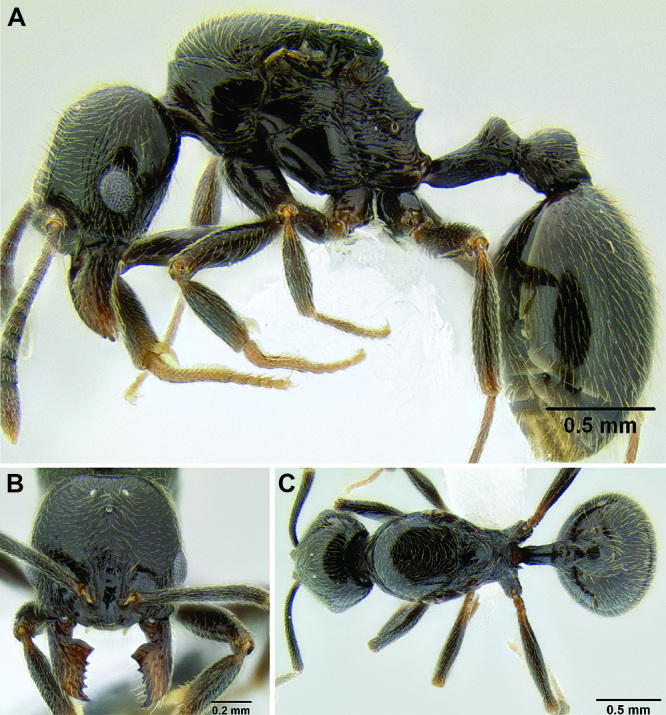
*Stenamma maximon* queen (CASENT0604871) **A** Profile **B** Face **C** Dorsum.

**Figure 118.**
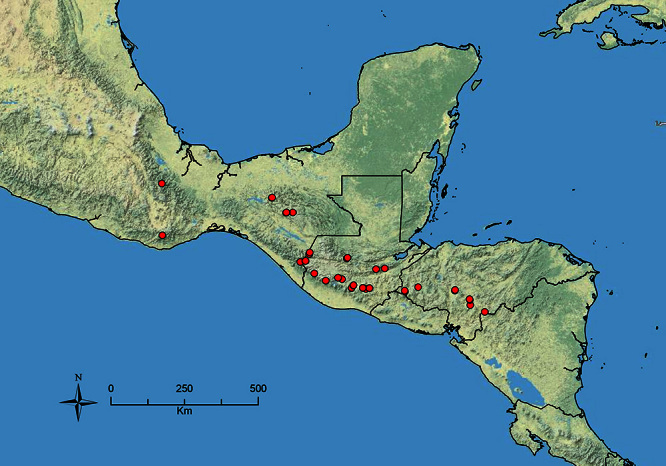
Distribution map of *Stenamma maximon*.

Worker: Figures 115 , 116 ; Queen: Figure 117 ; Map: Figure 118

Stenamma mgb01 Branstetter, 2012: phylogeny.

#### Type material.

*Holotype worker*. GUATEMALA: El Progresso, Cerro Pinalón, 15.08654°N, 89.94445°W ±50m, 2550m, 30 Apr 2009, cloud forest, ex sifted leaf litter (LLAMA, collection Wa-B-01-2-02) [USNM, specimen CASENT0604875]. *Paratypes*: same data as holotype but 15.08753°N, 89.94446°W ±50m, 2550m, 30 Apr 2009 (LLAMA, Wa-B-01-2-38) [1w, CAS, CASENT0623344], [1w, EAPZ, CASENT0623345], [1w, ECOSCE, CASENT0623346], [1w, FMNH, CASENT0623347], [1w, ICN, CASENT0623348], [1w, INBio, CASENT0623349], [1w, JTLC, CASENT0623350], [1w, LACM, CASENT0623351], [1w, MGBPC, CASENT0623352], [1w, MCZ, CASENT0623353], [1w, MZSP, CASENT0623354], [1w, UCD, CASENT0623355], [1w, UNAM, CASENT0623356], [1w, UVGC, CASENT0623357]; 15.08350°N, 89.95319°W ±55m, 2500m, 2 May 2009 (LLAMA, Wm-B-01-2-02) [1dq, 2w, USNM, CASENT0623358, CASENT0623359, CASENT0623360].

#### Worker diagnosis.

Integument mostly black, or dark brown to brown; medium-sized species (see HL, ML, PrW below); basal margin of mandible usually sinuous, with a distinct basal depression, but without a basal tooth (rarely basal margin appearing nearly straight); anterior clypeal margin undulating, with 2–4 distinct to somewhat blunt teeth; face sculpture variable, usually mostly smooth and shiny, with, at most, faint carinulae extending to about ¾ the distance to posterior margin of head, but some populations with face mostly rugoreticulate, with only area around posterior margin becoming smooth; pronotum sculpture variable, usually mostly smooth and shiny, but sometimes with some longitudinal rugulae on humeri, remainder of mesosoma with rugulae and punctae; postpetiole bulging, appearing distinctly larger than petiolar node (PPH/PH 0.96–1.14; PW/PPW 0.65–0.78); eye of moderate size (EL 0.11–0.16, REL 16–22), oval-shaped, with 6–8 ommatidia at greatest diameter (rarely 9 or 10); propodeal spines usually tuberculate, or at least forming a sharp angle, but sometimes forming a blunt angle, or becoming more developed, short (PSL 0.07–0.12, PSI 1.0–2.0). *Similar species*: *Stenamma crypticum*, *Stenamma huachucanum*, *Stenamma ochrocnemis*.

#### Geographic range.

Southern Mexico to Honduras.

#### Worker description.

(23 measured) HL 0.67–0.86 (0.79), HW 0.57–0.76 (0.68), FLD 0.16–0.21 (0.19), PCW 0.02–0.0.05 (0.04), SL 0.53–0.74 (0.68), EL 0.11–0.16 (0.12), ACL 0.49–0.65 (0.59), ML 0.84–1.09 (1.02), PrW 0.39–0.50 (0.46), PSL 0.07–0.12, SDL 0.06–0.09, PL 0.29–0.38 (0.37), PH 0.19–0.24 (0.23), PW 0.15–0.19 (0.18), PPL 0.21–0.27 (0.24), PPH 0.20–0.25 (0.25), PPW 0.22–0.27 (0.26), MFL 0.57–0.82 (0.76), MTL 0.48–0.65 (0.61), CI 83–93 (85), SI 80–101 (100), REL 16–22 (18), FLI 25–30 (28), PSI 1.0–2.0 (1.1), MFI 89–108 (89), ACI1 66–70 (68), ACI2 85–96 (88).

Medium-sized species; general body color mostly black, or dark brown to brown, with appendages dark brown to orange-brown, lighter at joints and toward extremities; setae golden brown; mandible with 6–8 teeth (usually 7), consisting of 3 distinct apical teeth, a basal tooth, and 2–4 smaller teeth/denticles, which are often worn and indistinct; basal margin of mandible usually sinuous, with a distinct basal depression, but without a basal tooth, sometimes basal margin only slightly sinuous or nearly straight; mandible often mostly smooth and shiny, with scattered piligerous punctae and some striations (type population), but sometimes surface completely carinulate, with carinulae extending from base to near teeth; anterior clypeal margin forming 2–4 distinct to somewhat blunt teeth, outer teeth often larger and sharper than inner teeth (best viewed in anterodorsal view); median clypeal lobe with a pair of faint longitudinal carinulae, apex of lobe with a short transverse carinula, remainder of clypeus mostly smooth and shiny; area in between median lobe of clypeus and anterior clypeal margin forming a distinct cavity where mandibles insert; posterior extension of clypeus between antennal insertions of moderate width (PCW 0.02–0.0.05), with sides subparallel to slightly hour-glass-shaped; frontal lobes of moderate width (FLD 0.16–0.21, FLI 25–30), not greatly covering torular lobes in full-face view; head roughly oval-shaped (CI 83–93), with posterior margin flat to slightly depressed mesad; face sculpture variable, usually mostly smooth and shiny (type population), with, at most, faint carinulae extending to about ¾ the distance to posterior margin of head, but some populations with face mostly rugoreticulate, with only area around posterior margin becoming smooth; scape of moderate length (SI 80–101), reaching posterior margin of head when laid back; scape surface mostly smooth and shiny, with scattered piligerous punctae; flagellum with a distinct 4-segmented antennal club; pronotum and mesonotal dorsum sculpture variable, usually mostly smooth and shiny, but sometimes with longitudinal rugulae on humeri, remainder of mesosoma mostly sculptured (contrast between smooth pronotum, and sculptured mesonotum, mesopleuron, and propodeum distinctive); mesopleuron mostly punctate, with a few rugulae; side of propodeum with rugulae and some faint punctae; dorsum and declivity of propodeum with variable number of transverse carinulae; promesonotum in profile low-domed, and roughly symmetrical, but anterior declivity sometimes more sharply angled than posterior face (type population); metanotal groove in profile well-demarcated, anterior slope often smoothly transitioning with promesonotum; propodeal spines usually tuberculate (type population), or at least forming sharp angles, but sometimes forming blunt angles, or more developed and short (PSL 0.07–0.12, PSI 1.0–2.0); petiole of moderate length (PL/HW 0.46–0.56), with peduncle thick, often with anteroventral edge sharp; petiolar node in profile somewhat small (PH/PL 0.58–0.68), wedge-shaped, with anterior face longer and more sloping than almost vertical posterior face, dorsum of node rounded, and distinctly pointed posteriad; postpetiole bulging, globular, appearing distinctly larger than petiolar node (PPH/PH 0.96–1.14; PW/PPW 0.65–0.78), anterior face of node much longer than posterior face; petiolar and postpetiolar nodes mostly smooth and shiny, remaining waist surfaces punctate, with a few rugulae around bases of nodes; gaster smooth and shiny, with scattered piligerous punctae; pilosity on face short, with a layer of sparse suberect setae, and a denser layer of decumbent setae, remainder of body dorsum with longer standing pilosity; pilosity on gastral dorsum usually distinctly bilayered, with a layer of longer suberect to subdecumbent setae, and a layer of shorter decumbent setae, both layers similar in density; setae on scape and legs mostly decumbent to appressed, with some longer suberect setae on femoral venters and coxae.

#### Queen description.

(10 measured) HL 0.73–0.84 (0.84), HW 0.67–0.76 (0.76), FLD 0.19–0.23 (0.23), PCW 0.03–0.06 (0.06), SL 0.57–0.71 (0.71), EL 0.19–0.22 (0.22), ACL 0.47–0.62 (0.62), ML 1.05–1.34 (1.34), PrW 0.56–0.74 (0.74), PSL 0.13–0.17 (0.14), SDL 0.09–0.10 (0.10), PL 0.37–0.47 (0.47), PH 0.23–0.27 (0.27), PW 0.19–0.22 (0.22), PPL 0.20–0.29 (0.27), PPH 0.24–0.30 (0.30), PPW 0.27–0.33 (0.33), MFL 0.63–0.84 (0.84), MTL 0.55–0.70 (0.70), CI 91–93 (91), SI 82–94 (94), REL 26–30 (29), FLI 27–30 (30), PSI 1.4–1.7 (1.4), MFI 90–108 (90), ACI1 64–68 (65), ACI2 76–93 (87).

Same as worker except for standard queen modifications and as follows (comparison between worker and queen of type population form only): facial sculpture usually more developed (carinulae/rugulae longer and more distinct); mesopleuron mostly smooth and shiny; propodeum with transverse carinulae that wrap around entire surface; lower layer of gastral setae more dense, almost pubescent; propodeal spines (compared to worker from same population) slightly longer, more developed (PSL 0.13–0.17, PSI 1.4–1.7).

#### Male.

Unknown.

#### Biology.

*Stenamma maximon* is an inhabitant of high elevation wet forest habitats (e.g. cloud forest, wet oak forest, hardwood forest, wet oak-pine forest), ranging from approximately 1700–2800 m elevation. When present, *Stenamma maximon* workers are one of the most common components of the leaf-litter microenvironment, with most collections of the species made by sifting leaf litter from the forest floor. It has been collected also fairly commonly at cookie baits, suggesting that workers do forage epigeically some of the time. This is not surprising since *Stenamma maximon* is a larger species of *Stenamma*. I have found only one nest of *Stenamma maximon*, which was in a small log in leaf litter. A few stray workers have been collected also from underneath a rock. Given these observations, I would guess that *Stenamma maximon* nests in wood in the leaf litter and forages both within the leaf litter and sometimes above it.

#### Comments.

The presence of a bulging postpetiole, a sinuous basal margin of the mandible, and a toothed anterior clypeal margin, makes *Stenamma maximon* easily separable from most other *Stenamma* species. However, *Stenamma maximon* forms a complex of phenotypic variants (described below) and some populations lose the distinctiveness of the type population form and are more difficult to separate from similar species.

Distinctive features of the type population form (Figures 115, 116) are indicated in the species description (see parenthetical comments). Most notable are the characters mentioned in the previous paragraph, combined with a mostly smooth head and promesonotum. This form occurs mainly at high elevation sites in Chiapas, Mexico, and throughout most of Guatemala. It co-occurs with variant 1 at several sites in Guatemala.

Variant 1 (Figure 116A–C) includes many populations in Guatemala and Honduras. Each is variable and some are intermediate in morphology between variant 1 and the type form. Variant 1 differs from the type form as follows: basal margin of mandible only slightly sinuous, without a distinct basal depression; teeth of anterior clypeal margin reduced, sometimes only two blunt middle teeth or undulations perceptible; face strongly sculptured with rugoreticulae and carinulae, only sometimes posterior ¼ of head smooth; lateral margins of promesonotal dorsum with distinctive oblique carinulae/rugulae that cross from upper portion of pronotal side to dorsum, middle of promesonotum smooth; propodeal spines reduced to blunt angles. I have found variant 1 with the type form most often at the locality 5km SE Antigua in Guatemala. Figure 116A–F shows two specimens from this locality, with D–F corresponding to the type form. This sympatry suggests that the two forms are somewhat reproductively isolated from one another. I have not separated variant 1 as a distinct species, however, because some populations, especially in Honduras, appear intermediate in phenotype. The general pattern I see is that the type form occurs in Chiapas, Mexico, to Guatemala; variant 1 occurs in Guatemala, often in sympatry with the type form; and then populations in Honduras appear intermediate. I also see some similarity between variant 1 and certain populations of *Stenamma crypticum*, perhaps indicating that these two species occasionally hybridize.

Variant 2 (Figure 116G–I) is known from a few collections made from the wet side of the Sierra Juarez Mountains in Oaxaca, Mexico. These specimens are larger than average, have short propodeal spines (longer than in type form), and have lighter brown-black body coloration.

Variant 3 (Figure 116J–L) is known from a single collection at 4.6km S Suchixtepec in Oaxaca, Mexico. It is similar to variant 2, except that the basal margin of the mandible is nearly straight and the anterior clypeal margin appears as a median emargination, without teeth. It is possible this variant is not conspecific with *Stenamma maximon*, but has evolved similar features due to convergence. I have not been able to test this hypothesis with molecular data, but it should be done.

#### Material examined.

**EL SALVADOR: *Santa Ana***: Cerro Verde, [ca. 13.827°N, 86.631°W], 1760m, 14 May 1971 (S. B. Peck); **GUATEMALA: *Chimaltenango***: Finca Chincharas, Rincón Suizo, 14.79775°N, 90.98380°N, 2490m, 17 Sep 2008 (R. S. Anderson); 3.8km NNE Tecpán, 14.7969°N, 90.9817°W, 2500m, 17 Sep 2008 (M. G. Branstetter);***El Progresso***: Cerro Pinalón, 15.08654°N, 89.94445°W, 2550m, 30 Apr 2009 (M. G. Branstetter); ***Guatemala***: nr Las Nubes, 14.53975°N, 90.34882°W, 2050m, 18 Sep 2008 (R. S. Anderson); 7km ESE San José Pinula, 14.5392°N, 90.3493°W, 2060m, 18 Sep 2008 (M. G. Branstetter); ***Jalapa***: Aldea Manzano, 1.8km WNW San José La Sierra, 14.5048°N, 90.2561°W, 1990m, 18 Sep 2008 (M. G. Branstetter); El Manzano, 14.50476°N, 90.25613°W, 2150m, 18 Sep 2008 (R. S. Anderson); 4km E Mataquescuintla, 14.53329°N, 90.15314°W, 2400m, 1 Jun 2009 (LLAMA); 4km E Mataquescuintla, 14.52952°N, 90.14806°W, 2600m, 1 Jun 2009 (LLAMA);5km ENE Mataquescuintla, 14.5336°N, 90.1432°W, 255 0m, 5 Jul 2007 (J. L. Cozar); ***Huehuetenango***: above Bojonal, 15.53697°N, 91.98158°W, 2350m, 16 Sep 2008 (R. S. Anderson); 3.3km WSW El Paraíso, 15.5370°N, 91.9816°W, 2350m, 16 Sep 2008 (M. G. Branstetter); San Miguel Chicharro, 4km NNW El Paraíso, 15.5796°N, 91.9692°W, 2100m, 16 Sep 2008 (L. Sáenz); ***Quetzaltenango***: Fuentes Georginas, 5.1km SW Santa Maria, 14.7489°N, 91.4806°W, 2500m, 13 Sep 2008 (M. G. Branstetter);12km SE Zunil, NW face Cerro Zunil, [ca. 14.7488°N, 91.4800°N,], 2700m, 28 May 1991 (R. S. Anderson); 12km SE Zunil, Fuentes Georginas, [ca. 14.7488°N, 91.4800°W], 2460m, 27 May 1991 (R. S. Anderson); ***Quiché***: Laguna Danta, 15.42729°N, 90.81689°W, 2300m, 15 Sep 2008 (R. S. Anderson); 5.1km SE Los Encuentros, 14.83598°N, 91.10867°W, 2660m, 17 Sep 2008 (L. Sáenz); 10km E Los Encuentros, 14.83598°N, 91.10867°W, 2620m, 17 Sep 2008 (R. S. Anderson); 1.4km SW Macalajau, 15.4056°N, 90.8381°W, 2360m, 15 Sep 2008 (M. G. Branstetter); 3km S Joya Larga, 15.40538°N, 90.83805°W, 2350m, 15 Sep 2008 (R. S. Anderson); ***Sacatepéquez***: 5km SE Antigua, 14.53651°N, 90.69480°W, 2150m, 10 Jun 2009 (LLAMA); 5km SE Antigua, 14.52848°N, 90.68884°W, 2350m, 10 Jun 2009 (LLAMA);5km SE Antigua, 14.52357°N, 90.68800°W, 2470m, 11 Jun 2009 (LLAMA); Cerro Alux, 14.61053°N, 90.64191°W, 2190m, 9 Sep 2008 (R. S. Anderson); Cerro Carmona, 6km SE Antigua, 14.5345°N, 90.6945°W, 2180m, 9 Sep 2008 (L. Sáenz); Guatemala City, Cerro Alux, [ca. 14.6167°N, 90.6333°W], 2260m, 9 Jun 1991 (R. S. Anderson); ***San Marcos***: 5.3km WSW San Marcos, 14.9635°N, 91.8314°W, 2520m, 11 Sep 2008 (M. G. Branstetter); ***Zacapa***: 14km NNE Teculután, 15.1144°N, 89.6805°W, 2270m, 6 Jul 2007 (M. G. Branstetter) **HONDURAS:** ***Comayagua***: 10km E Comayagua, 14.45976°N, 87.54591°W, 2000m, 15 May 2010 (LLAMA); 12km ENE Comayagua, 14.48045°N, 87.53238°W, 2140m, 15 May 2010 (M. G. Branstetter); 18km ENE Comayagua, [ca. 14.456°N, 87.541°W], 17 Jun 1994 (S. & J. Peck);***Francisco Morazán***: PN La Tigra, 3.6km SW San Juancito, 14.2073°N, 87.0943°W, 25 Sep 2008 (M. G. Branstetter); 23km N Tegucigalpa, PN La Tigre, Esperanza Trail, [ca. 14.181°N, 87.090°W], 15 Jul 1994 (S. B. Peck); Reserva Uyuca, 4.8km WNW Zamorano, 14.02695°N, 87.07077°W, 1900m, 24 Sep 2008 (M. G. Branstetter);***Lempira***: PN Celaque, 8.7km SW Graçias, 14.5588°N, 88.6612°W, 2100m, 30 Sep 2008 (M. G. Branstetter); ***Ocotepeque***: 13km E Nueva Ocotepeque, 14.42478°N, 89.0606°W, 2140m, 26 May 2010 (LLAMA); 13km E Nueva Ocotopeque, 14.45697°N, 89.06849°W, 2200m, 25 May 2010 (LLAMA); 13km E Nueva Ocotopeque, 14.41807°N, 89.06915°W, 2210m, 26 May 2010 (LLAMA); **MÉXICO:** ***Chiapas***: Cerro Huitepec (Pico), ca. 5km W San Cristobal, [ca. 16.7500°N, 92.6802°W], 2750m, 18 Sep 1991 (R. S. Anderson); Cerro de Tapalapa, 17.18786°N, 93.12308°W, 2260m, 28 May 2008 (R. S. Anderson); 5km NE Coapilla, 17.17602°N, 93.13293°W, 1990m, 26 May 2008 (M. G. Branstetter); 7.3km NE Coapilla, 17.1826°N, 93.1182°W, 2200m, 12 Jul 2007 (M. G. Branstetter); Huitepec, S. Cristóbal, 16.75018°N, 92.68331°W, 2480m, 29 May 2008 (LLAMA); 7.4km SSW Montozintla de Mendoza, [ca. 15.3049°N, 92.2597°W], 2000m, 17 Sep 1992 (R. S. Anderson); Reserva Huitepec, 16.7448°N, 92.6885°W, 2600m, 11 Jul 2007 (J. Longino); 5km W San Cristobal, Cerro Huitepec, 16.75000°N, 92.68023°W, 2700m, 14 Sep 1992 (R. S. Anderson); 15km E San Cristóbal, 16.74713°N, 92.49002°W, 2500m, 29 May 2008 (LLAMA); 4km N Union Juarez, Volcan Tacana, lower slopes, [ca. 15.333°N, 92.100°W], 2000m, 20 Sep 1992 (R. S. Anderson); ***Oaxaca***: 5.1km S Suchixtepec, [ca. 16.0833°N, 96.4667°W], 2150m, 25 Jul 1992 (R. S. Anderson); 27km SW Valle Nacional, 17.59582°N, 96.47572°W, 2290m, 11 Aug 2009 (M. G. Branstetter).

### Stenamma megamanni sp. n.

urn:lsid:zoobank.org:act:AC3D916C-B156-4A58-88CD-6476825F3194

http://species-id.net/wiki/Stenamma_megamanni

**Figure 119.**
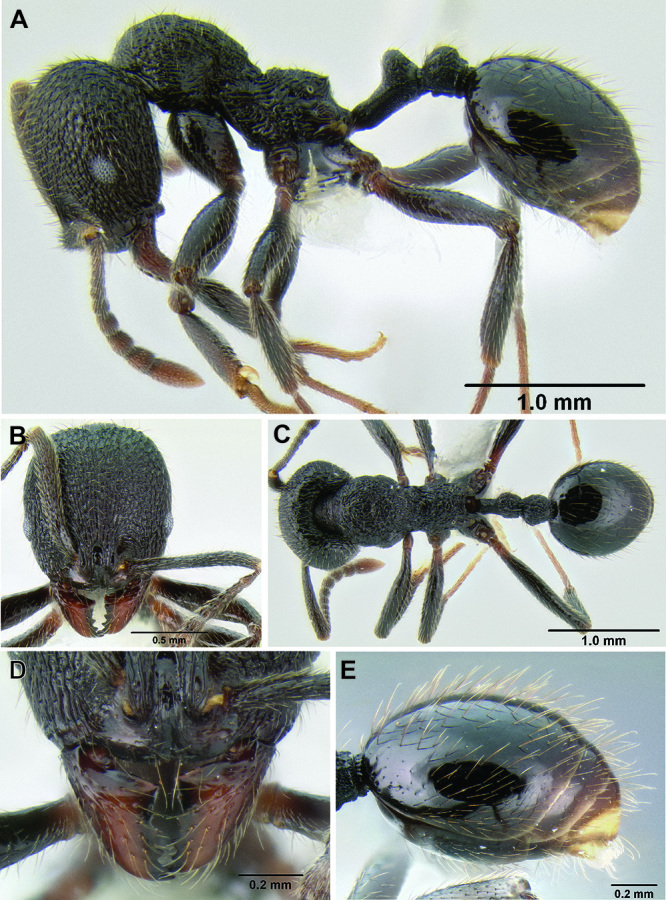
*Stenamma megamanni* holotype worker (CASENT0622851) **A** Profile **B** Face **C** Dorsum **D** Anterior clypeal margin in anterodorsal view **E** Gaster.

**Figure 120.**
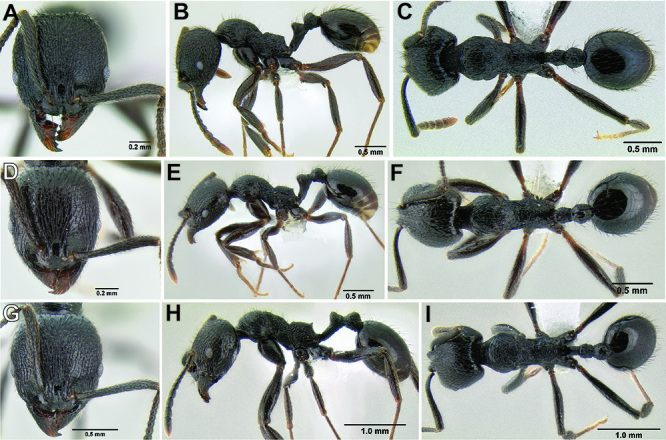
*Stenamma megamanni* worker variants. Face, profile, dorsal views **A–C** Variant 1 (CASENT0621836) **D–F** Variant 2 (CASENT0606734) **G–I** Variant 3 (CASENT0603924).

**Figure 121.**
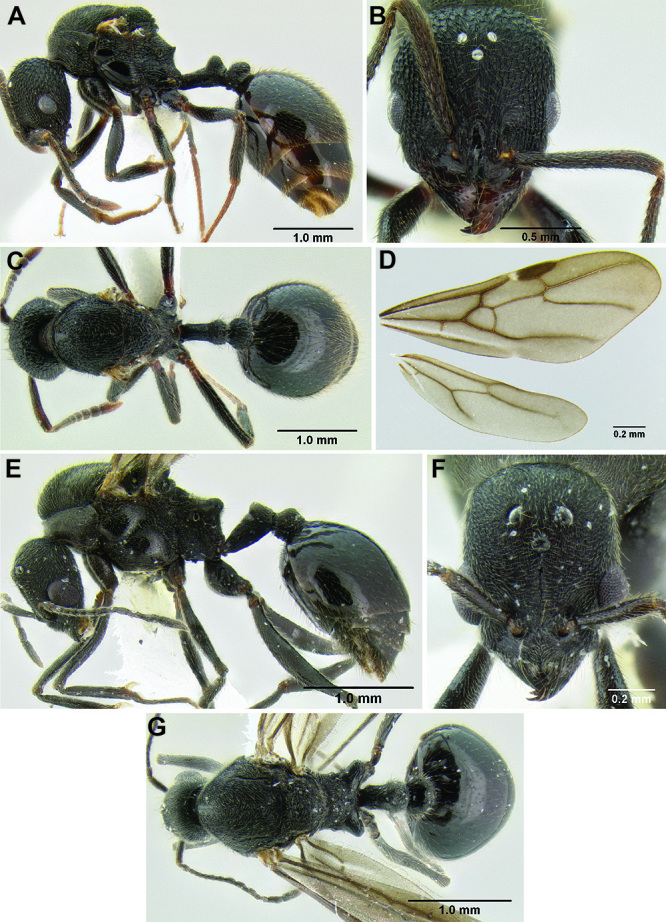
*Stenamma megamanni* **A** Paratype queen (CASENT0604840), profile **B** Same, face **C** Same, dorsum **D** Same, wings **E** Male (CASENT000007293), profile **F** Same, face **G** Same, dorsum.

**Figure 122.**
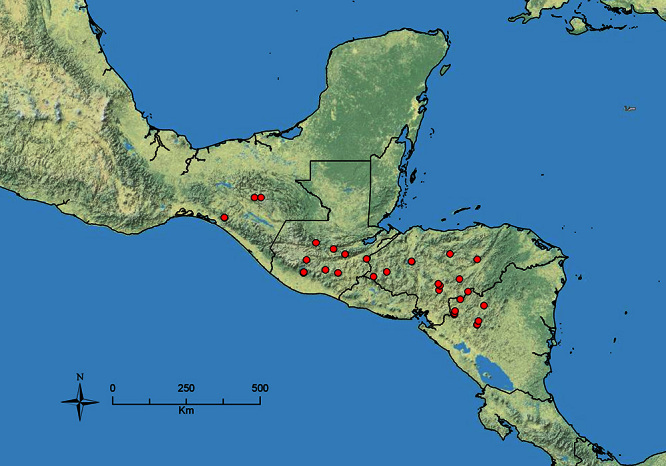
Distribution map of *Stenamma megamanni*.

Worker: Figures 119 , 120 ; Queen: Figure 121A–D ; Male: Figure 121E–G ; Map: Figure 122

#### Type material.

*Holotype worker*. MÉXICO, Chiapas: Sierra Morena, 16.16121°N, 93.60024°W ±50m, 1320m, 15 May 2008, riparian mesophyll forest, nest under stone (M. G. Branstetter, collection MGB750) [USNM, specimen CASENT0622851]. *Paratypes*: same data as holotype [1w, CAS, CASENT0622852], [1w, EAPZ, CASENT0622853], [1w, ECOSCE, CASENT0622854], [1w, FMNH, CASENT0622855], [1w, ICN, CASENT0622856], [1w, INBio, CASENT0623361], [1w, JTLC, CASENT0623362], [1w, LACM, CASENT0623363], [1w, MGBPC, CASENT0623364], [1w, MCZ, CASENT0623365], [1w, MZSP, CASENT0623366], [1w, UCD, CASENT0623367], [1w, UNAM, CASENT0623368, [1w, UVGC, CASENT0623369], [1w, USNM, CASENT0623370]; same data but 16.16016°N, 93.60572°W ±15m, 1360m, 15 May 2008 (LLAMA, Ma-A-01-1-01) [1dq, USNM, CASENT0604840].

#### Worker diagnosis.

Integument mostly black to red-black; medium- to large-sized species (see HL, ML, PrW below); lateral apex of hypostomal bridge projecting ventrally as a subquadrate to broadly rounded lobe, visible behind base of mandible in profile view; propodeal spines tuberculate (PSL 0.10–0.15, PSI 1.0–1.2); face and mesosoma usually completely sculptured with rugoreticulae and some carinulae (rarely with sculpture reduced); eye of moderate to large size (EL 0.14–0.19, REL 15–20), oval-shaped, with 8–11 ommatidia in greatest diameter; postpetiole usually appearing smaller than petiolar node (PPH/PH 0.85–0.98); basal margin of mandible straight to slightly sinuous; anterior clypeal margin with a median emargination. *Similar species*: *Stenamma manni*.

#### Geographic range.

Southern Mexico to Nicaragua.

#### Worker description.

(12 measured) HL 0.93–1.15 (1.07), HW 0.80–1.08 (0.98), FLD 0.22–0.29 (0.25), PCW 0.05–0.10 (0.07), SL 0.79–0.98 (0.91), EL 0.14–0.19 (0.17), ACL 0.70–0.86 (0.78), ML 1.18–1.48 (1.36), PrW 0.53–0.69 (0.63), PSL 0.10–0.15 (0.12), SDL 0.09–0.14 (0.11), PL 0.40–0.54 (0.45), PH 0.25–0.32 (0.30), PW 0.18–0.25 (0.23), PPL 0.23–0.33 (0.26), PPH 0.21–0.31 (0.26), PPW 0.23–0.30 (0.28), MFL 0.97–1.23 (1.14), MTL 0.72–0.93 (0.85), CI 86–96 (91), SI 89–100 (93), REL 15–20 (18), FLI 25–28 (26), PSI 1.0–1.2 (1.1), MFI 82–90 (86), ACI1 61–63 (62), ACI2 84–89 (86).

Medium- to large-sized species; general body color black to red-black, with appendages black or dark brown to orange-brown, becoming lighter toward extremities at joints; setae dark golden brown to golden brown; mandible with 6 teeth, 2 teeth nearest basal tooth sometimes worn and indistinct; basal margin of mandible straight to slightly sinuous, without a basal depression or notch; mandible mostly smooth, with scattered piligerous punctae and a few striations near base and on lateral surface; anterior clypeal margin with a simple median emargination; median lobe of clypeus usually with a pair of longitudinal carinulae (type population), but sometimes surface smooth, or with several irregular carinulae, area in between carinulae often depressed; apex of lobe usually with a single transverse short carinula (type population), but sometimes with several transverse carinulae/rugulae; remainder of clypeus smooth and shiny; posterior extension of clypeus between antennal insertions of moderate width (PCW 0.05–0.10), sides subparallel; frontal lobes of moderate width (FLD 0.22–0.29; FLI 25–28) not greatly obscuring torular lobes in full-face view; lateral margin of hypostomal bridge with a projecting subquadrate lobe, visible in profile view (very rarely reduced and only visible from a lateroventral view); head roughly oval-shaped to more robust, becoming strongly heart-shaped (CI 86–96), posterior margin of head with a distinct median depression (especially in larger specimens); eye moderately large (EL 0.14–0.19, REL 15–20), oval-shaped, with 8–11 ommatidia at greatest diameter; face sculpture variable, usually strongly rugoreticulate, with some carinulae along midline (type population), but sometimes sculpture more polished, with area near posterior margin of head becoming somewhat smooth; scape of moderate length (SI 89–100), reaching posterior margin of head when laid back; scape usually thick and robust, surface carinate to carinulae with piligerous punctae; flagellum with a somewhat indistinct 4-segmented antennal club; mesosoma sculpture variable, often densely rugose, with rugae wavy, almost becoming reticulate (type population), but sculpture often reduced, with promesonotum longitudinally carinulate to nearly smooth, and side of propodeum and mesopleuron more punctate; promesonotum in profile domed, and usually asymmetrical (type population), with apex shifted anterior of midpoint, but sometimes promesonotum symmetrical; metanotal groove well-demarcated, sometimes somewhat wide and with metanotum forming a small welt (type population); propodeal spines tuberculate (PSL 0.10–0.15, PSI 1.0–1.2); petiole of moderate length, usually somewhat robust (PL/HW 0.45–0.52); petiolar node in profile of moderate height (PH/PL 0.59–0.68), dorsum usually broadly rounded and pointing vertically, only rarely forming a sharp apex; postpetiole in profile usually appearing smaller than petiolar node (PPH/PH 0.85–0.98); petiole and postpetiole usually strongly punctate, with a few rugulae on dorsum and anterior face of postpetiolar node (type population), sometimes anterior faces of petiolar and petiolar nodes mostly smooth; gaster mostly smooth, with scattered piligerous punctae; most of body dorsum with relatively long standing pilosity; pilosity on gastral dorsum sparse and mostly suberect (type population), usually with some shorter subdecumbent setae, suberect setae often somewhat stout; setae on promesonotum often noticeably erect; setae on scape uniformly suberect to decumbent; setae on legs mostly decumbent to appressed, with some suberect setae on femoral venters and coxae.

#### Queen description.

(5 measured) HL 1.01–1.12 (1.11), HW 0.92–1.03 (0.99), FLD 0.27–0.31 (0.31), PCW 0.07–0.10 (0.09), SL 0.87–0.97 (0.95), EL 0.26–0.28 (0.28), ACL 0.76–0.83 (0.83), ML 1.55–1.72 (1.71), PrW 0.93–1.04 (0.99), PSL 0.20–0.23 (0.20), SDL 0.15–0.17 (0.16), PL 0.57–0.60 (0.60), PH 0.33–0.39 (0.36), PW 0.26–0.31 (0.30), PPL 0.30–0.34 (0.34), PPH 0.32–0.39 (0.38), PPW 0.35–0.40 (0.38), MFL 1.13–1.26 (1.26), MTL 0.86–0.96 (0.93), CI 89–92 (89), SI 93–96 (96), REL 28–29 (28), FLI 28–31 (31), PSI 1.2–1.4 (1.2), MFI 78–84 (78), ACI1 60–62 (60), ACI2 83–89 (87).

Same as worker except for standard queen modifications and as follows: pronotum with transverse rugulae; mesoscutum longitudinally costulate; scutellum rugose to rugoreticulate; propodeum rugose to rugoreticulate; postpetiolar node larger, and somewhat anteroposteriorly compressed; mesopleuron mostly smooth; setae on mesoscutum short; setae on gastral dorsum often more dense, and more strongly bilayered, with layer of decumbent setae more dense; anterior quarter of gastral dorsum often with a layer of pubescence under stouter suberect and decumbent setae; wing venation as in Figure 121D.

#### Male.

See Figure 121E–G. Note the rather peculiar propodeal protuberances. I have observed these in several specimens from different sites. Many males, however, including males from the same nest, have only small sharp tubercles.

#### Biology.

*Stenamma megamanni* occurs from 700–2800 m, but it is most common from 1400–2000 m (there is also one dubious record from 150 m at Pico Bonito). It mainly inhabits montane wet forest environments, such as cloud forest, oak-pine forest, riparian forest, and mesophyll forest. Specimens have been collected in samples of sifted leaf litter, by baiting, by use of a Malaise trap, and by searching. Nests are known from rotting logs, under rocks, in mud and clay banks, and in dead bark. Most collections of this species have been inside closed-canopy forest, but it has been found several times nesting in clearings near cloud forest (similar to *Stenamma manni*). The highest record of the species was a log nest in a clearing between lower elevation cloud forest and higher elevation pine forest.

The type population was found nesting under rocks along a stream in mesophyll forest. This population was rather intriguing, because the nests were common and foragers were very abundant. In fact, *Stenamma megamanni* workers were more common at cookie bait cards placed along the stream than the usually more dominant ant genus *Pheidole*. This phenomenon of becoming locally abundant and dominant seems a frequent characteristic of this species. At Cerro Kilambé in Nicaragua, for example, *Stenamma megamanni* was nesting in clay banks bordering a small stream and was the dominant ant in the area.

Nests of this species are large for *Stenamma*, often containing several hundred workers. One nest at Cerro Kilambé, which was excavated from a mud bank, had 2-3 nest entrances and many chambers all filled with brood. The nest went about 10 cm into the bank, contained about 250 workers and had abundant larvae and pupae. Another nest from the same site was similar in size, but the chambers were constructed around small roots. All excavated nests have had single dealate queens.

#### Comments.

As described above under *Stenamma manni*, separating *Stenamma megamanni* from *Stenamma manni* is difficult, because of the great diversity in *Stenamma manni* phenotypes across its range. At a local scale, however, separating *Stenamma megamanni* from *Stenamma manni* is usually easy. *Stenamma megamanni* is black and has a rather large eye with 8 or more ommatidia at greatest diameter. *Stenamma manni* in contrast is usually a dark red-brown color and has a smaller eye with 5–6 ommatidia at greatest diameter. Preliminary phylogenetic data show that specimens from the type population and several divergent populations of *Stenamma megamanni* form a clade nested within the *Stenamma manni* complex (Branstetter unpublished data). This result has provided evidence that *Stenamma megamanni* is a good species.

Within my concept of *Stenamma megamanni* there is significant variation in sculpture, size and pilosity among populations. Because I perceive the variation to be somewhat continuous, I do not describe separate variants, but I do provide some images depicting the variation (Figure 120A–F). The type form appears rather robust with the promesonotum high-domed, and asymmetrical, and the sculpture very dense (Figure 119A–E). The pronotal sculpture has very wavy, almost reticulate rugae. Most populations do not match the type population exactly. The closest is a collection at the locality 2km NE Macalajau in Guatemala, which was made at high elevation (2320 m) from a ground nest under a rock in a cloud forest clearing. This habit is quite different from that of the type population, which was found at lower elevation along a stream (see the biology section above). More commonly, workers appear somewhat less robust (Figure 120A–C), with less dense sculpturing. A few populations have workers with very reduced face and promesonotal sculpture, and with more erect pilosity (Figure 120D–F). One aberrant high-elevation collection from Guatemala (Pinalón) has very large workers, with transverse carinulae on the pronotum (Figure 120G–I).

#### Material examined.

**GUATEMALA: *Baja Verapaz***: nr Purulhá (Old Salama Road), 15.24114°N, 90.28392°W, 1660m, 20 Sep 2008 (R. S. Anderson); ***El Progreso***: Cerro Pinalón, 15.08324°N, 89.92681°W, 2800m, 4 May 2009 (LLAMA);***Guatemala***: 1km SE La Pueblito, [ca. 14.6211°N, 90.5269°W], 1800m, 10 Jun 1991 (R. S. Anderson); ***Jalapa***: Miramundo, Pino Dulce, 14.53388°N, 90.15236°W, 2300m, 18 Sep 2008 (R. S. Anderson); ***Quiché***: 2.9km SSE Chichicastenango, 14.91861°N, 91.10449°W, 2000m, 17 Sep 2008 (M. G. Branstetter);2km NE Macalajau, 15.42729°N, 90.81580°W, 2320m, 15 Sep 2008 (M. G. Branstetter); ***Suchitepéquez***: Vol. Atitlán, Ref. El Quetzal, 14.55785°N, 91.19203°W, 2000m, 10 Sep 2008 (R. S. Anderson); 3km S Vol. Atitlán, 14.55558°N, 91.19285°W, 1875m, 16 Jun 2009 (J. Longino); 5km S Vol. Atitlán, 14.53500°N, 91.19871°W, 1195m, 17 Jun 2009 (J. Longino); ***Zacapa***: 2km SE La Unión, 14.94460°N, 89.27726°W, 1550m, 14 May 2009 (LLAMA); **HONDURAS: *Atlántida***: PN Pico Bonito, 7.8km SSW La Ceiba, 15.69040°N, 86.90272°W, 150m, 2 Oct 2008 (M. G. Branstetter); ***Comayagua***: PN Cerro Azul Meambar, 14.86613°N, 87.89736°W, 940m, 21 May 2010 (LLAMA); PN Cerro Azul Meambar, 3.4km ESE La Guama, 14.87221°N, 87.90535°W, 760m, 26 Sep 2008 (M. G. Branstetter); ***Francisco Morazán***: 21km S Guaimaca, 14.35444°N, 86.4444°W, 1360m, 14 May 2009 (J. Longino); Mina Las Animas, above Valle de Angeles, [ca. 14.16°N, 87.04°W], 1580m, 25 Mar 1979 (W. L Brown); PN La Tigra, 3.6km SW San Juancito, 14.20731°N, 87.09428°W, 1830m, 25 Sep 2008 (M. G. Branstetter); Reserva Uyuca, 4.9km WNW Zamorano, 14.02685°N, 87.06995°W, 1890m, 24 Sep 2008 (M. G. Branstetter); ***Lempira***: PN Celaque, 8.3km SW Graçias, 14.56170°N, 88.6583°W, 1860m, 30 Sep 2008 (C. Viquez); ***Ocotepeque***: 13km E Nueva Ocotepeque, 14.42478°N, 89.06060°W, 2140m, 26 May 2010 (LLAMA); ***Olancho***: 9km N Catacamas, 14.93503°N, 85.90748°W, 1350m, 11 May 2010 (LLAMA); PN La Muralla, 15.09697°N, 86.73624°W, 1520m, 4 May 2010 (LLAMA); **MÉXICO:** ***Chiapas***: 15km E San Cristóbal, 16.74687°N, 92.48985°W, 2500m, 29 May 2008 (D. J. Cox); Reserva Huitepec, 16.74476°N, 92.68853°W, 2600m, 11 Jul 2007 (J. Longino); Sierra Morena, 16.15946°N, 93.60510°W, 1360m, 12 May 2008 (LLAMA); Sierra Morena, 16.16466°N, 93.60573°W, 1570m, 15 May 2008 (R. S. Anderson); 18.5km ENE Tonalá, 16.15888°N, 93.60378°W, 1330m (R. S. Anderson); **NICARAGUA: *Jinotega***: RN Cerro Kilambé, 13.56915°N, 85.69747°W, 1500, 23 May 2011 (LLAMA); RN Datanlí El Diablo, 13.10883°N, 85.86763°W, 1440m, 18 May 2011 (LLAMA); ***Madriz***: Apante, 9km S Somoto, 13.40481°N, 86.57944°W, 1550m, 22 Apr 2011 (M. G. Branstetter); 2km SE Las Sabanas, 13.3006°N, 86.60582°W, 1640m, 23 Apr 2011 (L. Sáenz); 2.3km E Las Sabanas, 13.34028°N, 86.59924°W, 1640m, 23 Apr 2011 (M. G. Branstetter); 16km S Somoto, 13.33607°N, 86.60313°W, 1660m, 23 Apr 2011 (R. S. Anderson); 16km S Somoto, 13.32821°N, 86.61028°W, 1705m, 23 Apr 2011 (R. S. Anderson); ***Matagalpa***: Fuente Pura, [ca. 12.999°N, 85.910°W], 10 Apr 1994 (Maes & De La Fuente) Selva Negra, 9.2km NNE Matagalpa, 13.00283°N, 85.90646°W, 1330m, 7 Oct 2008 (M. G. Branstetter); ***Nueva Segovia***: Cerro Mogotón, 13.75460°N, 86.4229°W, 1980m, 24 Apr 2011 (L. Sáenz); 9km NW Jalapa, 13.98207°N, 86.18916°W, 1675m, 28 May 2011 (LLAMA).

### Stenamma monstrosum sp. n.

urn:lsid:zoobank.org:act:BD4D739F-D7C5-48F3-B9E9-AB55A1B18A23

http://species-id.net/wiki/Stenamma_monstrosum

**Figure 123.**
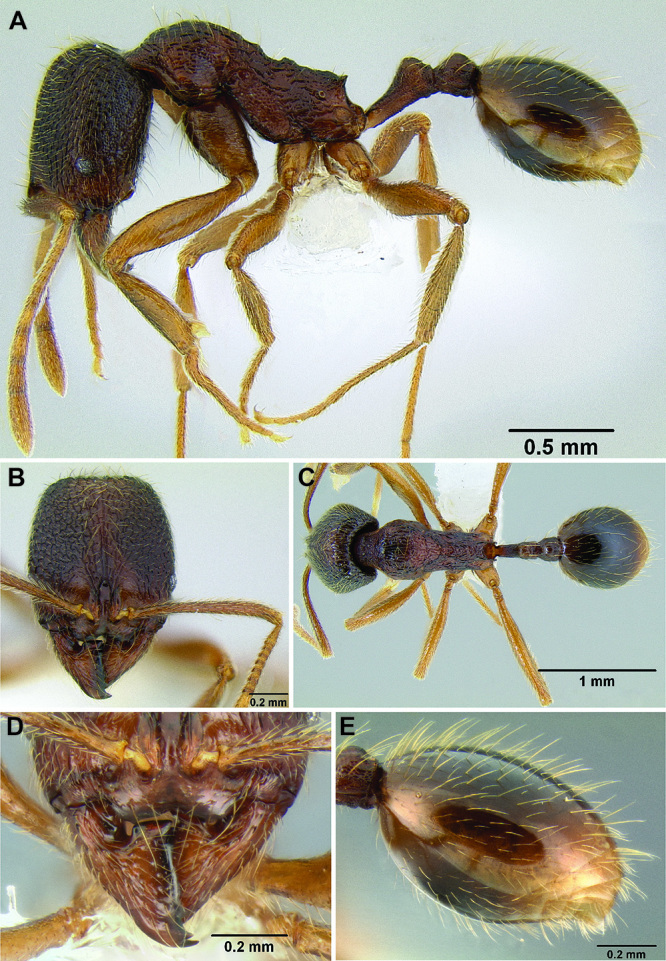
*Stenamma monstrosum* holotype worker (CASENT0621327) **A** Profile **B** Face **C** Dorsum **D** Anterior clypeal margin in anterodorsal view **E** Gaster.

**Figure 124.**
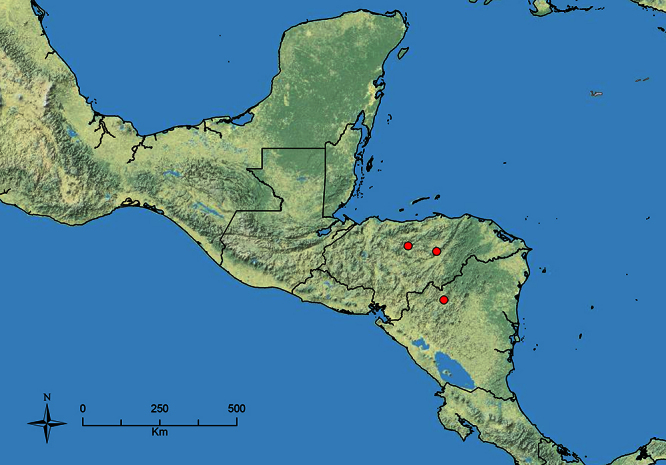
Distribution map of *Stenamma monstrosum*.

Worker: Figure 123 ; Map: Figure 124

#### Type material.

*Holotype worker*. HONDURAS: Olancho, Parque Nacional La Muralla, 15.09537°N, 86.73887°W ±20m, 1420m, 2 May 2010, cloud forest, ex sifted leaf litter (LLAMA, collection Wa-C-01-2-06) [USNM, specimen CASENT0621327]. *Paratypes*: same data as holotype [1w, EAPZ, CASENT0621329], [1w, MGBPC, CASENT0621330], [1w, MCZ, CASENT0621328], [1w, UCD, CASENT0621326].

#### Worker diagnosis.

Integument mostly mottled brown to red-brown or orange-brown; medium-sized species (see HL, ML, PrW below); basal margin of mandible with a deep proximal notch containing a small tooth; anterior clypeal margin with a deep unevenly rounded depression that, when viewed from an anterodorsal angle, has two distinct outer teeth and two indistinct inner teeth (all pointing ventrally); mesosoma profile appearing elongate, almost gracile; metanotal grove relatively wide and shallow, not well demarcated by the promesonotal and propodeal margins; propodeal dorsum elongate, distinctly longer than propodeal declivity; gastral pilosity clearly bilayered, with a layer of long suberect setae and a layer of short subdecumbent setae; eye relatively small (EL 0.09–0.11, REL 13–15), with 5–6 ommatidia at greatest diameter; scape and metafemur relatively long (SI 104–120, MFI 70–80); frontal lobes well-developed (FLD 0.16–0.19, FLI 23–25), but not completely covering torular lobes in full-face view; 4-segmented antennal club very distinct. *Similar species*: *Stenamma manni*, *Stenamma saenzae*.

#### Geographic range.

Honduras to Nicaragua.

#### Worker description.

(5 measured) HL 0.79–0.94 (0.91), HW 0.63–0.82 (0.78), FLD 0.16–0.19 (0.17), PCW 0.03–0.03 (0.03), SL 0.76–0.86 (0.83), EL 0.09–0.11 (0.11), ACL 0.75–0.80 (0.78), ML 1.03–1.21 (1.15), PrW 0.46–0.55 (0.52), PSL 0.12–0.15 (0.15), SDL 0.11–0.12 (0.11), PL 0.38–0.42 (0.42), PH 0.19–0.23 (0.23), PW 0.14–0.17 (0.15), PPL 0.19–0.20 (0.20), PPH 0.17–0.21 (0.20), PPW 0.18–0.22 (0.21), MFL 0.90–1.03 (1.10), MTL 0.71–0.83 (0.82), CI 80–87 (85), SI 104–120 (107), REL 13–15 (14), FLI 23–25 (23), PSI 1.1–1.3 (1.3), MFI 70–80 (77), ACI1 62–64 (63), ACI2 93–98 (95).

Medium-sized species; general body color a mottled brown to red-brown or orange-brown, with patches of yellow-brown, especially on gaster; appendages generally lighter, orange-brown to yellow-brown; setae golden brown; mandible with 2–3 distinct apical teeth, 1–2 distinct basal teeth, and a variable number of almost imperceptible denticles in between (usually appearing as a long, flat diastema), basal tooth well-defined and robust; basal margin of mandible with a very deep proximal notch containing a small tooth; mandible mostly smooth and shiny, with scattered piligerous punctae and a variable number of striae; anterior clypeal margin with a deep unevenly rounded depression, that, when viewed anteriorly, has two distinct outer teeth and two indistinct inner teeth (all pointing ventrally); median lobe of clypeus somewhat obliquely flattened and concave, without a pair of longitudinal carinulae, only sometimes with a short transverse carinula before anterior margin, remainder of clypeus mostly smooth and shining; posterior extension of clypeus between antennal insertions somewhat narrow (PCW 0.03), sides subparallel; frontal lobes well-developed (FLD 0.16–0.19, FLI 23–25), but not completely covering torular lobes in full-face view; head elongate and roughly oval-shaped (CI 80–87), with posterior margin depressed medially; eye relatively small (EL 0.09–0.11, REL 13–15), subcircular to oval-shaped, with 5–6 ommatidia at greatest diameter; face densely rugoreticulate, with a few longitudinal rugulae medially, interstices with faint piligerous punctae; scape relatively long (SI 104–120), surpassing posterior margin of head when laid back; scape cuticle mostly smooth and shiny, with scattered piligerous punctae and fine striations; flagellum with a very distinct 4-segmented antennal club; mesosoma densely sculptured, promesonotal dorsum mostly with longitudinal rugae and rugoreticulae, but a few transverse rugulae present around anterior declivity; lateral side of mesosoma with irregular rugulae and punctae; propodeal declivity mostly smooth and shining, sometimes with faint transverse striae; promesonotum in profile low-domed and slightly asymmetrical, with the apex occurring anterior of the midpoint, anterior face more strongly defined and longer than posterior face; metanotal grove relatively wide and shallow, not well demarcated by the promesonotal and propodeal margins; propodeal dorsum elongate, distinctly longer than propodeal declivity; mesosomal outline in profile appearing elongate, almost gracile; propodeal spines forming sharp tubercles (PSL 0.12–0.15, PSI 1.1–1.3); petiole somewhat elongate and gracile (PL/HW 0.51–0.60), node small (PH/PL 0.53–0.56), with a long sloping anterior face, and a short vertical posterior face, dorsal surface in profile view rounded, pointing vertically; postpetiolar node slightly smaller than petiolar node (PPH/PH 0.90–0.97), anterior face longer and more sloping angled than posterior face; petiole and postpetiole mostly punctate, with anterior face of postpetiolar node largely smooth and shiny; gaster mostly smooth and shiny, with scattered piligerous punctae; much of body with long suberect to subdecumbent pilosity; scapes with mostly subdecumbent setae; gastral pilosity somewhat sparse and clearly bilayered, with a layer of long suberect setae and a layer of short subdecumbent setae; setae on legs mostly subdecumbent to appressed, with suberect setae on ventral surface of profemur.

#### Queen.

Unknown.

#### Male.

Unknown.

#### Biology.

*Stenamma monstrosum* is a rare species known from only three collections of sifted leaf litter taken from the forest floor. It occurs in montane wet forest (e.g. cloud forest, mixed hardwood forest) and has been recorded from 1400–1650 m elevation. Because of its rarity in leaf litter, and its gracile phenotype, I hypothesize that *Stenamma monstrosum* is arboreal.

#### Comments.

*Stenamma monstrosum* looks like a cross between *Stenamma manni* and *Stenamma saenzae*. However, I doubt that a hybridization scenario is likely, because the species occurs at three different sites, and maintains a consistent phenotype among sites. It also is quite distinctive, with some of its own unique features, such as the shallow metanotal groove, and the deeply emarginate clypeus. These character states, along with the tooth in the basal margin of the mandible, will easily separate *Stenamma monstrosum* from any other species.

#### Material examined.

**HONDURA**S: ***Olancho***: 10km N Catacamas, 14.94359°N, 85.91056°W, 1650m, 9 May 2010 (R. S. Anderson); PN La Muralla, 15.09537°N, 86.73887°W, 1420m, 2 May 2010 (LLAMA); **NICARAGUA: *Jinotega***: RN Cerro Kilambé, 13.57109°N, 85.69764°W, 1500m, 23 May 2011 (LLAMA).

### Stenamma muralla sp. n.

urn:lsid:zoobank.org:act:8E1C2421-AADD-44A3-A453-39C3648655AF

http://species-id.net/wiki/Stenamma_muralla

**Figure 125.**
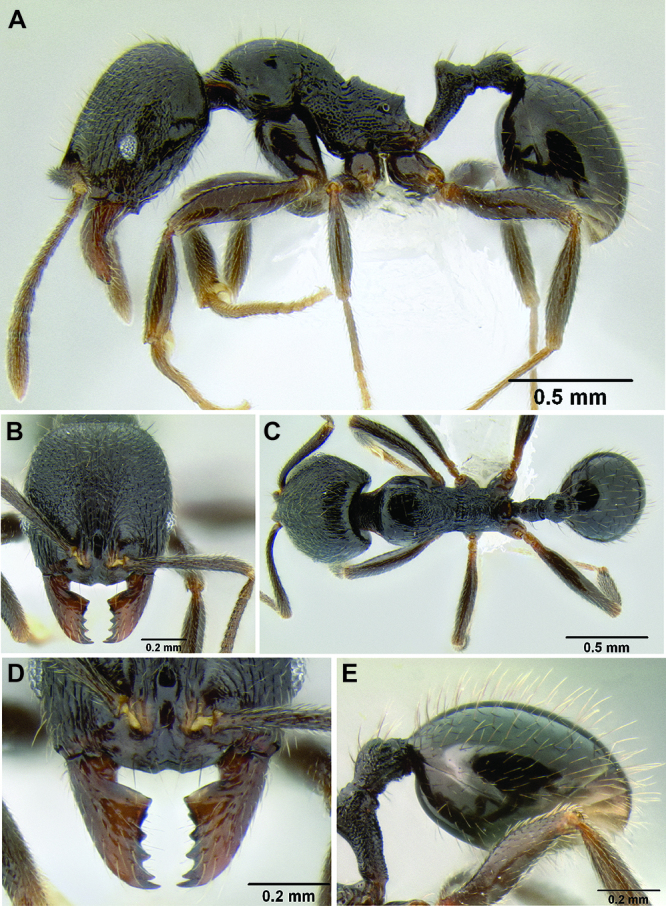
*Stenamma muralla* holotype worker (CASENT0621311) **A** Profile **B** Face **C** Dorsum **D **Anterior clypeal margin in anterodorsal view **E** Gaster.

**Figure 126.**
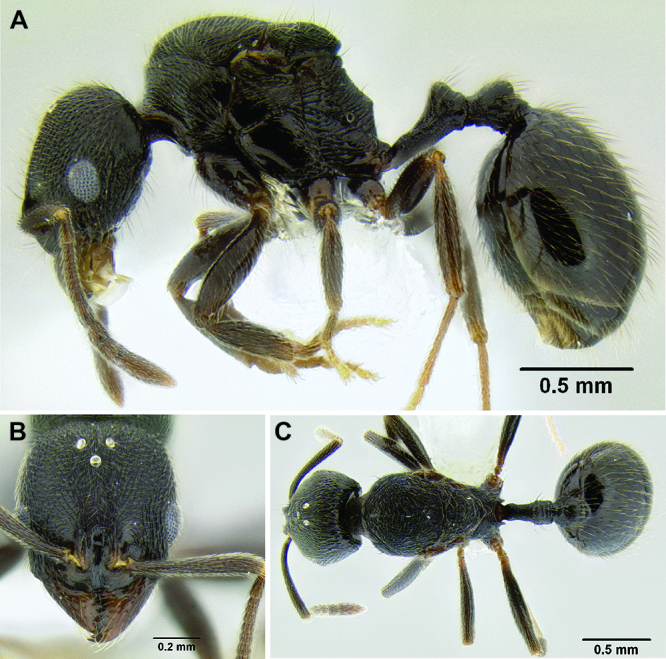
*Stenamma muralla* paratype queen (CASENT0622025) **A** Profile **B** Face **C** Dorsum.

**Figure 127.**
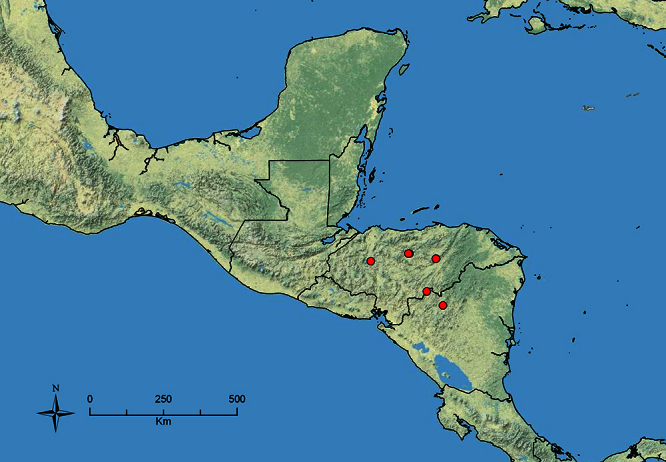
Distribution map of *Stenamma muralla*.

Worker: Figure 125 ; Queen: Figure 126 ; Map: Figure 127

#### Type material.

*Holotype worker*. HONDURAS: Olancho, Parque Nacional La Muralla, 15.09969°N, 86.74673°W ±20m, 1530m, 2 May 2010, cloud forest, ex sifted leaf litter (LLAMA, collection Wa-C-01-1-37) [USNM, specimen CASENT0621311]. *Paratypes*: same data as holotype but 15.09859°N, 86.74216°W ±10m, 1510m, 2 May 2010 (LLAMA, Wm-C-01-1-03) [1w, CAS, CASENT0622027], [1w, EAPZ, CASENT0623371], [1w, ECOSCE, CASENT0623372], [1w, FMNH, CASENT0623373], [1w, ICN, CASENT0623374], [1w, INBio, CASENT0623375], [1w, JTLC, CASENT0623376], [1w, LACM, CASENT0623377], [1w, MGBPC, CASENT0623378], [1w, MCZ, CASENT0623379], [1w, MZSP, CASENT0623380], [1w, UCD, CASENT0623381], [1w, UNAM, CASENT0623382], [1dq, 1w, USNM, CASENT0622025, CASENT0622026] [1w, UVGC, CASENT0623383].

#### Worker diagnosis.

Integument mostly black or dark brown to brown; small- to medium-sized species (see HL, ML, PrW below); lateral margin of hypostomal bridge with a projecting subquadrate lobe, visible behind mandible in profile view; waist segments appearing somewhat slender, with postpetiole particularly small; eye relatively large (EL 0.13–0.16, REL 19–22), with 6–9 ommatidia at greatest diameter; anterior clypeal margin with a median emargination; basal margin of mandible straight; propodeal spines tuberculate (PSL 0.08–0.11, PSI 1.1–1.4); frontal lobes well-developed, but not completely covering torular lobes in full-face view (FLD 0.16–0.19, FLI 24–28); head densely sculptured with carinulae, rugoreticulae, and faint punctae; mesosoma mostly sculptured, but pronotal sculpture variably effaced, sometimes mostly smooth and shiny; gastral setae mostly suberect, long, and relatively sparse. *Similar species*: *Stenamma leptospinum*, *Stenamma longinoi*, *Stenamma manni*.

#### Geographic range.

Honduras to Nicaragua.

#### Worker description.

(10 measured) HL 0.72–0.83 (0.74), HW 0.63–0.75 (0.67), FLD 0.16–0.19 (0.17), PCW 0.03–0.06 (0.04), SL 0.57–0.67 (0.59), EL 0.13–0.16 (0.13), ACL 0.56–0.62 (0.56), ML 0.89–1.08 (0.90), PrW 0.43–0.50 (0.44), PSL 0.08–0.11 (0.09), SDL 0.07–0.09 (0.08), PL 0.31–0.42 (0.32), PH 0.19–0.24 (0.19), PW 0.13–0.19 (0.15), PPL 0.18–0.24 (0.18), PPH 0.14–0.20 (0.15), PPW 0.17–0.24 (0.18), MFL 0.68–0.79 (0.70), MTL 0.54–0.65 (0.56), CI 87–90 (90), SI 88–95 (88), REL 19–22 (19), FLI 24–28 (25), PSI 1.1–1.4 (1.2), MFI 90–98 (96), ACI2 63–66 (65), ACI2 92–99 (96).

Small- to medium-sized species; general body color black or dark brown to brown, with appendages brown to yellow-brown; setae golden brown; mandible with 6 teeth, but two teeth nearest the basal tooth smaller, often worn and indistinct or denticulate; basal margin of mandible straight, without basal notch or depression; mandible surface mostly smooth and shiny, with scattered piligerous punctae and basal striae; anterior clypeal margin with a median emargination; median lobe of clypeus with a few faint punctae, but lacking longitudinal carinulae, apex with a faint transverse carinula; remainder of clypeus mostly smooth and shiny; posterior extension of clypeus between antennal insertions of moderate width (PCW 0.03–0.06), sides subparallel; frontal lobes well-developed (FLD 0.16–0.19, FLI 24–28), but not completely obscuring torular lobes in full-face view; frontal carinae weak, fading into facial sculpture beyond frontal lobes, not surpassing level of anterior margin of eye; head oval-shaped (CI 87–90), posterior margin slightly depressed medially; eye relatively large (EL 0.13–0.16, REL 19–22), oval-shaped, with 6–9 ommatidia at greatest diameter; face densely sculptured with longitudinal carinulae along midline, turning into rugulae and rugoreticulae toward lateral margins, interstices punctate; scape of moderate length (SI 88–95), nearly reaching posterior margin of head when laid back; scape surface with a variable amount of fine punctae and striae; flagellum with distinct 4-segmented antennal club; pronotal sculpture mostly smooth and shiny, or with dense longitudinal rugulae and faint punctae, or with intermediate states with rugulae somewhat effaced (especially on pronotal side); mesonotal dorsum with fine carinulae and punctae; mesopleuron mostly punctate, with a few rugulae; propodeal side with rugulae and some punctae; propodeal dorsum and declivity with transverse carinulae; promesonotum in profile usually low-domed and roughly symmetrical, some specimens with promesonotum more robust, higher; metanotal grove well demarcated, of moderate depth and width; propodeal spines present as small tubercles (PSL 0.08–0.11, PSI 1.1–1.4); petiole and postpetiole appearing somewhat delicate with nodes relatively small (PL/HW 0.48–0.57, PH/PL 0.56–0.63, PPH/PH 0.74–0.84); petiolar node in profile pointing posteriad, with dorsum gently rounded; ventral surface of node concave; postpetiole in profile with a long shield-like anterior face and a short vertical posterior face, anterior half constricted; anterior faces of petiolar and postpetiolar nodes usually mostly smooth and shiny, remaining surface of waist segments mostly punctate; gaster mostly smooth and shiny, with scattered piligerous punctae and a few short striae around anterior constriction; most of body dorsum with moderately long standing pilosity; setae on scape suberect to appressed; gastral setae mostly suberect, long, and relatively sparse, a few shorter decumbent setae also present; setae on legs mostly subdecumbent to appressed, with suberect setae on coxae and femoral venters.

#### Queen description.

(5 measured) HL 0.76–0.79 (0.79), HW 0.68–0.72 (0.72), FLD 0.19–0.21 (0.20), PCW 0.04–0.06 (0.05), SL 0.62–0.63 (0.63), EL 0.21–0.22 (0.22), ACL 0.59–0.61 (0.59), ML 1.11–1.19 (1.18), PrW 0.63–0.67 (0.67), PSL 0.12–0.13 (0.13), SDL 0.10–0.12 (0.12), PL 0.42–0.45 (0.44), PH 0.23–0.25 (0.25), PW 0.17–0.19 (0.18), PPL 0.21–0.24 (0.22), PPH 0.20–0.23 (0.20), PPW 0.24–0.25 (0.25), MFL 0.74–0.79 (0.77), MTL 0.61–0.65 (0.63), CI 89–91 (91), SI 89–91 (89), REL 30–31 (30), FLI 28–29 (28), PSI 1.1–1.3 (1.1), MFI 90–93 (93), ACI1 63–65 (65), ACI2 94–98 (94).

Same as worker except for standard queen modifications and as follows: pronotum transversely carinulate/rugulose; mesoscutum and scutellum densely carinulate; mesopleuron mostly smooth and shiny; propodeum transversely carinulate, with carinulae wrapping around entire surface; petiole and postpetiole slightly more robust, with dorsum of petiolar node reaching a slightly more defined apex; gastral pilosity more dense.

#### Male.

Unknown.

#### Biology.

*Stenamma muralla* occurs in montane wet forest habitats (e.g. cloud forest, mixed hardwood forest, pine-mesophyll forest, ridgetop cloud forest, riparian cloud forest) from 1100–1900 m. It has been collected in extracts of sifted leaf litter, at cookie baits, beating vegetation, and in a flight intercept trap (single worker). Nests have been found by general searching in a variety of microhabitats, including wet clay banks along streams, dry clay banks along road cuts (in forest), in dead wood, in the base of a tree fern, and in mud at the base of a fallen tree. Nests are of moderate size and usually contain a few chambers.

#### Comments.

*Stenamma muralla* is similar to *Stenamma leptospinum*, *Stenamma longinoi*, and *Stenamma manni*, because all have the lateral margin of the hypostomal bridge enlarged into a subquadrate lobe. However, *Stenamma muralla* is easy to identify by its smaller size, more slender waist segments, and largely effaced pronotal sculpture. A few other species might look superficially like *Stenamma muralla*, but these will not have the enlarged hypostomal lobe.

An interesting phenomenon in *Stenamma muralla* is the regular occurrence of workers that are distinctly larger, and have the pronotum more densely sculptured with longitudinal rugulae/carinulae. Interestingly, the size difference seems to be discrete, rather than continuous. These specimens have been found in leaf litter samples as well as in nests, so they must be conspecific. They could be workers with queen-like traits (queens are larger than standard workers), but other than size and sculpture there are no further indications that they are intercastes. Another hypothesis is that they are a soldier-like worker caste. However, they do not show any allometric size differences.

#### Material examined.

**HONDURAS: *Comayagua***: PN Cerro Azul Meambar, 14.86951°N, 87.89696°W, 1140m, 20 May 2010 (LLAMA); ***Olancho***: 10km N Catacamas, 14.94125°N, 85.90385°W, 1320m, 10 May 2010 (LLAMA); 10km N Catacamas, 14.94359°N, 85.91056°W, 1650m, 9 May 2010 (LLAMA); PN La Muralla, 15.0969°N, 86.7453°W, 1460m, 5 May 2010 (LLAMA); PN La Muralla, 15.09969°N, 86.74673°W, 1530m, 2 May 2010 (LLAMA); PN La Muralla, 15.09542°N, 86.73879°W, 1530m, 2 May 2010 (LLAMA); PN La Muralla, 15.09599°N, 86.72974°W, 1560m, 3 May 2010 (LLAMA); PN La Muralla, 15.09841°N, 86.73298°W, 1630m, 5 May 2010 (LLAMA); PN La Muralla, 15.0979°N, 86.7203°W, 1860m, 3 May 2010 (J. Longino); **NICARAGUA:** ***Jinotega***: RN Cerro Kilambé, 13.56929°N, 85.69746°W, 1500m, 23 May 2011 (LLAMA); RN Cerro Kilambé, 13.56730°N, 85.69774°W, 1400m, 23 May 2011 (LLAMA); ***Nueva Segovia***: 9km NW Jalapa, 13.97925°N, 86.18832°W, 1530m, 19 May 2011 (LLAMA).

### Stenamma nanozoi sp. n.

urn:lsid:zoobank.org:act:CE6EF9A0-9DCF-4149-A85C-C2F0EC8272F0

http://species-id.net/wiki/Stenamma_nanozoi

**Figure 128.**
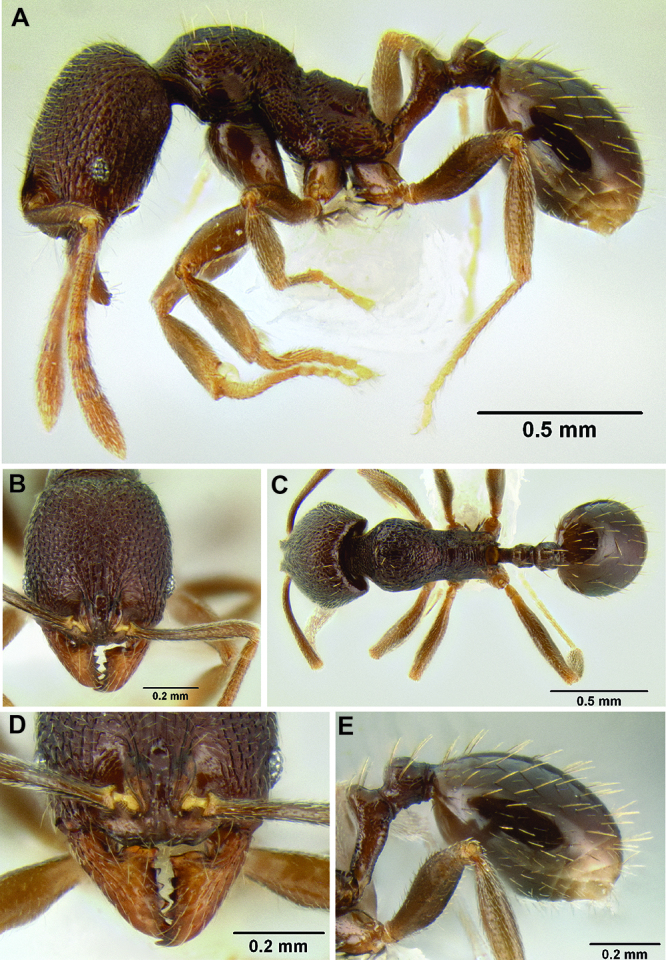
*Stenamma nanozoi* holotype worker (CASENT0621828) **A** Profile **B** Face **C** Dorsum **D **Anterior clypeal margin in anterodorsal view **E** Gaster.

**Figure 129.**
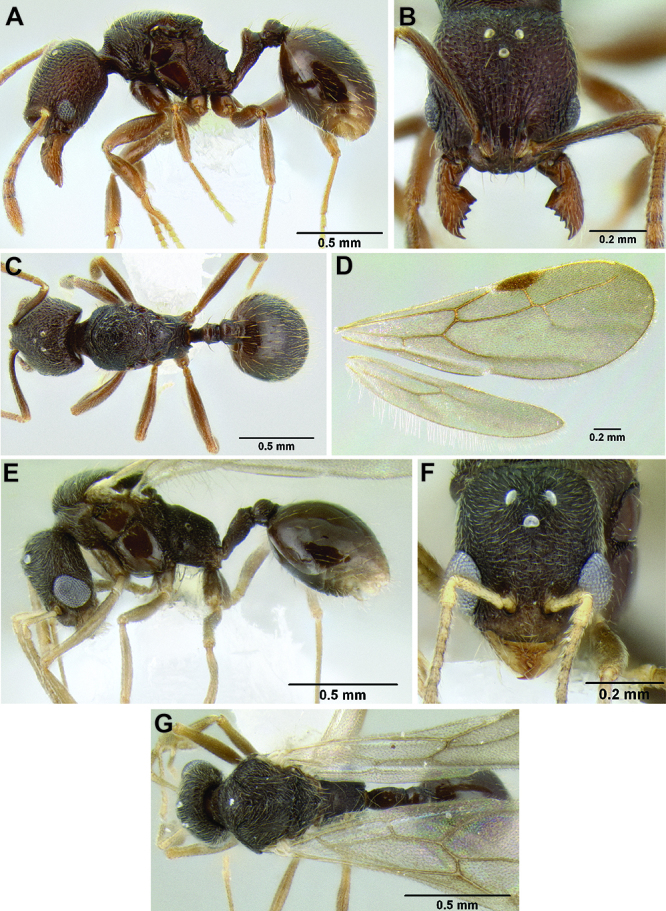
*Stenamma nanozoi* **A** Queen (CASENT0622005), profile **B** Same, face **C** Same, dorsum **D **Same, wings **E** Male (CASENT0622004), profile **F** Same, face **G** Same, dorsum.

**Figure 130.**
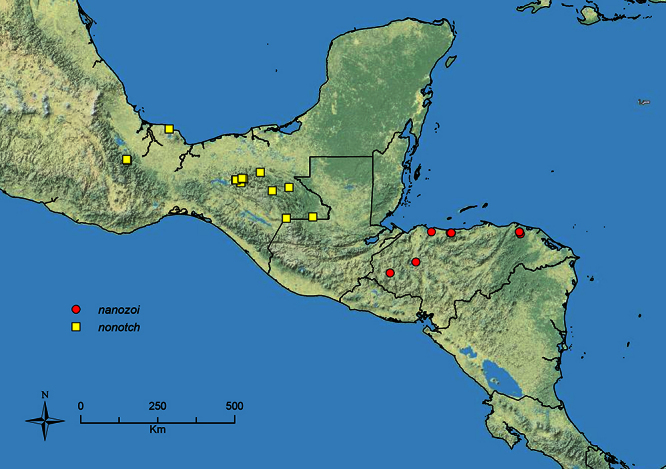
Distribution map of *Stenamma nanozoi* (circles) and *Stenamma nonotch* (squares).

Worker: Figure 128 ; Queen: Figure 129A–D ; Male: Figure 129E–G : Map: Figure 130

#### Type material.

*Holotype worker*. HONDURAS, 12km SW La Ceiba La Ceiba, 15.69175°N, 86.86977°W ±20m, 200m, 19 Jun 2010, tropical rainforest, ex sifted leaf litter (LLAMA, collection Wa-C-09-2-07) [USNM, specimen CASENT0621828]. *Paratypes*: same data as holotype [1dq, 1w, USNM, CASENT0621826, CASENT0621827]; same data but 15.69449°N, 86.86339°W ±20m, 200m, 19 Jun 2010 (LLAMA, Wa-C-09-2-25) [1dq, 1w, CAS, CASENT0623384, CASENT0623388], [1dq, 1w, EAPZ, CASENT0623385, CASENT0623389], [1w, ECOSCE, CASENT0623390], [1w, FMNH, CASENT0623391], [1w, ICN, CASENT0623392], [1dq, 1w INBio, CASENT0623386, CASENT0623393], [1w, JTLC, CASENT0623394], [1w, LACM, CASENT0623395], [1dq, 2w, MGBPC, CASENT0623387, CASENT0623396, CASENT0623397]; 15.69449°N, 86.86311°W ±20m, 200M, 19 Jun 2010 (LLAMA, Wa-C-09-1-31) [1dq, 1w, MCZ, CASENT0623400, CASENT0623402], [1w, MZSP, CASENT0623403], [1dq, 1w, UCD, CASENT0623401, CASENT0623404], [1w, UNAM, CASENT0623405], [1w, UVGC, CASENT0623406].

#### Worker diagnosis.

Integument mostly dark red-brown to brown; small-sized species (see HL, ML, PrW below); basal margin of mandible sinuous, with a small basal notch and accompanying tooth; anterior clypeal margin viewed at anterodorsal angle forming 4 blunt teeth (only outer teeth visible in full-face view); pilosity on gastral tergites mostly forming a moderately sparse layer of thickened suberect setae, with only a few thin decumbent setae present; face mostly rugoreticulate; mesosoma mostly rugulose and punctate, without large areas of smooth cuticle; eye of small to moderate size (EL 0.08–0.10, REL 15–18), oval-shaped, with 4–5 ommatidia at greatest diameter; propodeal spines tuberculate (PSL 0.07–0.09, PSI 1.2–1.4); frontal lobes somewhat expanded (FLD 0.14–0.16, FLI 28–31), but not completely obscuring torular lobes in full-face view. *Similar species*: *Stenamma nonotch*, *Stenamma saenzae*, *Stenamma sandinista*, *Stenamma schmidti*.

#### Geographic range.

Honduras.

#### Worker description.

(10 measured) HL 0.56–0.63 (0.61), HW 0.49–0.56 (0.54), FLD 0.14–0.16 (0.16), PCW 0.03 (0.03), SL 0.48–0.53 (0.51), EL 0.08–0.10 (0.10), ACL 0.48–0.53 (0.53), ML 0.68–0.76 (0.74), PrW 0.35–0.40 (0.38), PSL 0.07–0.09 (0.09), SDL 0.05–0.07 (0.07), PL 0.25–0.28 (0.27), PH 0.14–0.17 (0.15), PW 0.11–0.14 (0.13), PPL 0.13–0.16 (0.15), PPH 0.12–0.15 (0.14), PPW 0.14–0.16 (0.15), MFL 0.48–0.55 (0.55), MTL 0.39–0.44 (0.43), CI 87–92 (89), SI 90–99 (95), REL 15–18 (18), FLI 28–31 (30), PSI 1.2–1.4 (1.3), MFI 97–104 (98), ACI1 65–69 (66), ACI2 96–103 (103).

Small-sized species; general body color dark red-brown to brown, with appendages lighter, brown to yellow-brown toward extremities; setae golden brown; mandible with 6 teeth, basal tooth often well-defined, projecting; basal margin of mandible sinuous, with a distinct basal notch and accompanying small tooth; mandible mostly smooth, except for scattered piligerous punctae, and some striae around the basal and along lateral surface; anterior clypeal margin in anterodorsal view undulating, forming 4 blunt teeth (only outer teeth visible in full-face view); median lobe of clypeus with a pair of somewhat distinct longitudinal carinulae, that diverge toward anterior margin, apex of lobe with a short transverse carinula, remainder of clypeus mostly smooth; posterior extension of clypeus between antennal insertions somewhat narrow (FLD 0.14–0.16, FLI 28–31), with sides diverging slightly posteriad; frontal lobes somewhat expanded (FLD 0.14–0.16, FLI 28–31), but not completely obscuring torular lobes in full-face view; head roughly oval-shaped (CI 87–92), with posterior margin distinctly depressed medially; eye of small to moderate size (EL 0.08–0.10, REL 15–18), oval-shaped, with 4–5 ommatidia at greatest diameter; face mostly strongly rugoreticulate, with a few longitudinal carinulae along midline near frontal lobes; scape of moderate length (SI 90–99), not quite reaching posterior margin when laid back; scape surface mostly smooth, with faint striations and piligerous punctae; flagellum with a distinct 4-segmented antennal club; dorsum of pronotum with irregular longitudinal rugulae that sometimes merge, dorsum of metanotum more rugoreticulate-punctate, side of pronotum mostly carinulate, with a small patch of smooth cuticle near ventral margin, mesopleuron and side of propodeum rugulose-punctate, propodeal dorsum and upper half of declivity with transverse carinulae; promesonotum in profile low-domed, roughly symmetrical, but anterior face distinctly longer than posterior face; metanotal groove of moderate depth and width, sometimes with a small central welt (metanotum); propodeal spines tuberculate (PSL 0.07–0.09, PSI 1.2–1.4); petiole in profile of moderate length (PL/HW 0.48–0.54), peduncle somewhat slender, narrowing toward body; petiolar node of moderate height (PH/PL 0.52-0.61), asymmetrical, with anterior face slightly longer and more sloping than posterior face, dorsum of node narrowly rounded, almost becoming a well-defined apex, node always pointing distinctly posteriad; postpetiole in profile subspherical to oblong, appearing somewhat compressed anteroposteriorly, roughly symmetrical, and similar in size to slightly smaller than petiolar node (PPH/PH 0.83–0.99); anterior faces of petiolar and postpetiolar nodes mostly smooth, remaining surfaces mostly punctate, with some rugulae on posterior half of postpetiole; gaster mostly smooth and shiny, with scattered piligerous punctae; face pilosity short, bilayered; dorsum of mesosoma and gaster mostly with a sparse layer of thickened, suberect setae, gaster with a few thin decumbent setae; setae on scapes and legs mostly appressed, with some suberect setae on femoral venters and coxae.

#### Queen description.

(5 measured) HL 0.57–0.63 (0.62), HW 0.51–0.58 (0.57), FLD 0.16–0.18 (0.17), PCW 0.03–0.04 (0.04), SL 0.48–0.52 (0.52), EL 0.14–0.16 (0.16), ACL 0.48–0.52 (0.51), ML 0.75–0.85 (0.85), PrW 0.43–0.49 (0.49), PSL 0.09–0.11 (0.11), SDL 0.07–0.09 (0.08), PL 0.27–0.32 (0.31), PH 0.16–0.17 (0.17), PW 0.12–0.15 (0.15), PPL 0.14–0.15 (0.15), PPH 0.14–0.17 (0.17), PPW 0.15–0.18 (0.18), MFL 0.50–0.56 (0.56), MTL 0.40–0.45 (0.45), CI 90–92 (92), SI 90–97 (91), REL 26–28 (28), FLI 31–32 (31), PSI 1.2–1.4 (1.4), MFI 99–104 (102), ACI1 65, ACI2 99–101 (100).

Same as worker except for standard queen modifications and as follows: pronotum transversely carinulate, with dorsum of cervical shield punctate; mesoscutum longitudinally rugulose, with some irregular reticulations; scutellum longitudinally rugulose to rugoreticulate, usually with a small patch of smooth cuticle mesad; propodeum with transverse carinulae that wrap around surface; mesopleuron mostly smooth; pilosity on gaster distinctly bilayered, with a stout layer of long suberect to subdecumbent setae, and a very dense layer of decumbent to appressed pubescence; wing venation as in Figure 129D.

#### Male.

See Figure 129E–G.

#### Biology.

*Stenamma nanozoi* is mainly an inhabitant of lowland wet forest, with most specimens collected between 0–600 m elevation. However, a few specimens have been collected from montane *Liquidambar* forest at Celaque in Honduras between 1500–1600 m. All collections, except for one cookie bait sample, were made from sifted leaf litter taken from the forest floor.

#### Comments.

This species can be separated from similar forms by its mandible structure, eye size, gastral pilosity, facial sculpture, and overall size. Geography is also useful, as *Stenamma nanozoi* is known from only a few sites in Honduras, mostly at low elevation.

Given the complexity of the *Stenamma schmidti* complex (see below), it could be that *Stenamma nanozoi* represents an allopatric variant of *Stenamma schmidti*. I have decided to separate it out as a distinct species because it occurs at several sites in Honduras and maintains its distinctive features, and molecular data show it to be sister to *Stenamma sandinista* and outside of the clade that includes most *Stenamma schmidti* -complex forms. However, within the *Stenamma schmidti* complex there are several low-elevation variants that have some of the same characteristics as *Stenamma nanozoi*, mainly small size and thickened gastral setae. Thus, I view the species status of *Stenamma nanozoi* as somewhat tentative.

#### Material examined.

**HONDURAS: *Atlántida***: 12km SW La Ceiba, 15.69161°N, 86.86677°W, 280m, 19 Jun 2010 (LLAMA); PN Pico Bonito, 7.8km SSW La Ceiba, 15.69040°N, 86.90272°W, 150m, 2 Oct 2008 (M. G. Branstetter); 7km SW Tela, 15.72346°N, 87.45178°W, 190m, 15 Jun 2010; ***Comayagua***: PN Cerro Azul Meambar, 14.87038°N, 87.89896°W, 1120m, 20 May 2010; ***Gracias a Dios***: Las Marias, 15.66352°N, 84.85799°W, 60m, 8 Jun 2010 (LLAMA); Las Marias, 15.72200°N, 84.88325°W, 380m, 10 Jun 2010 (LLAMA); Las Marias, 15.72153°N, 84.88123°W, 560m, 10 Jun 2010 (LLAMA); Las Marias, 15.72235°N, 84.88480°W, 620m, 10 Jun 2010 (LLAMA);***Lempira***: PN Celaque, 7.3km SW Graçias, 14.56370°N, 88.64923°W, 1530m, 30 Sep 2008 (M. G. Branstetter).

### Stenamma nonotch sp. n.

urn:lsid:zoobank.org:act:D8476855-E0CF-4042-AC45-E4AF73F9B95B

http://species-id.net/wiki/Stenamma_nonotch

**Figure 131.**
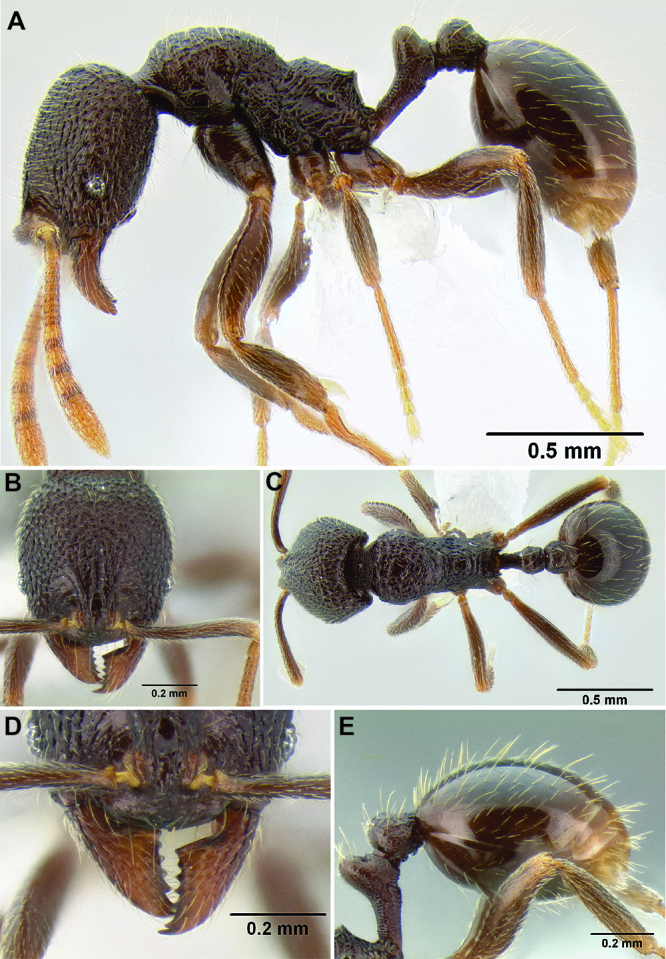
*Stenamma nonotch* holotype worker (CASENT060578) **A** Profile **B** Face **C** Dorsum **D **Anterior clypeal margin in anterodorsal view **E** Gaster.

**Figure 132.**
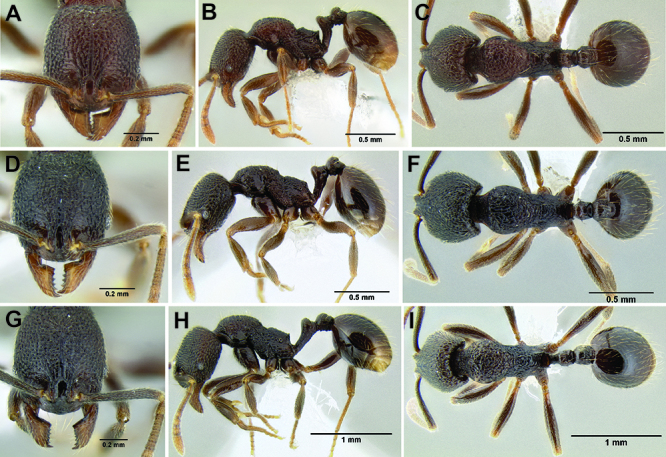
*Stenamma nonotch* worker variants. Face, profile, dorsal views **A–C** Variant 1 (CASENT0603920) **D–F** Variant 2 (CASENT0603896) **G–I** Variant 3 (CASENT0604711).

**Figure 133.**
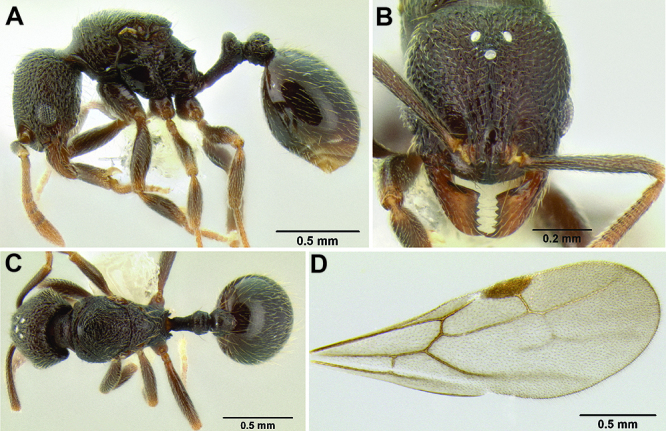
*Stenamma nonotch* **A** Queen (CASENT0605471), profile **B** Same, face **C** Same, dorsum **D **Queen (CASENT0605470), forewing.

Worker: Figures 131 , 132 ; Queen: Figure 133 ; Map: Figure 130

Stenamma mgb11 [variant 2 below] Branstetter, 2012: phylogeny.

#### Type material.

*Holotype worker*. MÉXICO, Oaxaca: 4.3km SW Valle Nacional, 17.74320°N, 96.32729°W ±40m, 370m, 13 Aug 2009, disturbed tropical rainforest, ex sifted leaf litter (L. Sáenz, collection LSD329) [USNM, specimen CASENT0605789]. *Paratypes*: same data as holotype [1dq, 1w, CAS, CASENT0605782, CASENT0605788], [1w, EAPZ, CASENT0605790], [1w, ECOSCE, CASENT0623408], [1w, FMNH, CASENT0623409], [1w, ICN, CASENT0623410], [1w, INBio, CASENT0623411], [1w, JTLC, CASENT0623412], [1w, LACM, CASENT0623413], [1w, MGBPC, CASENT0623414], [1dq, 1w, MCZ, CASENT0605784, CASENT0623415], [1w, MZSP, CASENT0623416], [1w, UCD, CASENT0623417], [1dq, 1w, UNAM, CASENT0605783, CASENT0623418], [1dq, 1w, USNM, CASENT0623407, CASENT0623419], [1w, UVGC, CASENT0623420].

#### Worker diagnosis.

Integument mostly dark red-brown (almost black) to brown; small- to medium-sized species (see HL, ML, PrW below); anterior clypeal margin entire, or at most with a nearly imperceptible median notch (only visible from anterodorsal angle); basal margin of mandible straight; face densely sculptured, mostly rugoreticulate; dorsum of promesonotum rugoreticulate, or with many irregular rugulae; pilosity on gastral tergites indistinctly bilayered, forming a somewhat sparse layer of long suberect setae, and a very sparse layer of short decumbent setae; eye of small to moderate size (EL 0.09–0.11, REL 15–19), oval-shaped, with 4–7 ommatidia at greatest diameter; propodeal spines reduced to small triangular tubercles (PSL 0.08–0.14, PSI 1.1–1.4); frontal lobes of moderate width (FLD 0.14–0.19, FLI 25–29). *Similar species*: *Stenamma ignotum*, *Stenamma nanozoi*, *Stenamma picopicucha*, *Stenamma sandinista*, *Stenamma schmidti*.

#### Geographic range.

Southern Mexico to Guatemala.

#### Worker description.

(17 measured) HL 0.57–0.76 (0.61), HW 0.51–0.68 (0.57), FLD 0.14–0.19 (0.15), PCW 0.03–0.07 (0.03), SL 0.46–0.64 (0.50), EL 0.09–0.11 (0.09), ACL 0.45–0.59 (0.49), ML 0.68–1.00 (0.78), PrW 0.36–0.49 (0.40), PSL 0.08–0.14 (0.11), SDL 0.06–0.11 (0.09), PL 0.26–0.40 (0.31), PH 0.16–0.23 (0.19), PW 0.14–0.19 (0.16), PPL 0.12–0.22 (0.15), PPH 0.13–0.21 (0.16), PPW 0.16–0.22 (0.18), MFL 0.50–0.72 (0.55), MTL 0.41–0.57 (0.45), CI 86–92 (92), SI 88–97 (89), REL 15–19 (16), FLI 25–29 (26), PSI 1.1–1.4 (1.3), MFI 93–104 (103), ACI1 65–69 (67), ACI2 92–99 (97).

Small- to medium-sized species; general body color dark red-brown (almost black) to brown, with appendages lighter, brown to yellow-brown toward extremities; setae golden; mandible with 6–7 teeth (usually 7), consisting of 3 larger, more distinct apical teeth, a basal tooth, and 2–3 smaller denticulate teeth, which are usually more worn, and sometimes indistinct; basal margin of mandible straight, without a basal notch or depression; mandible mostly smooth, except for scattered piligerous punctae, and a few basal striae; anterior clypeal margin entire, or at most with a nearly imperceptible median notch, only visible from anterodorsal view; median lobe of clypeus with faint longitudinal carinulae and some punctations, apex of lobe with a few short transverse carinulae, remainder of clypeus mostly smooth; posterior extension of clypeus between antennal insertions of moderate width (PCW 0.03–0.07), with sides subparallel to slightly hour-glass-shaped; frontal lobes of moderate width (FLD 0.14–0.19, FLI 25–29), not obscuring torular lobes in full-face view; head roughly oval-shaped (CI 86–92), posterior margin slightly depressed medially; eye of small to moderate size (EL 0.09–0.11, REL 15–19), oval-shaped, with 4–7 ommatidia at greatest diameter; face densely sculptured, mostly rugoreticulate, with a few longitudinal rugae along midline near frontal lobes; scape of moderate length (SI 88–97), almost reaching posterior margin of head when laid back; scape surface mostly smooth, with scattered piligerous punctae; flagellum with a distinct 4-segmented antennal club; most of mesosoma densely sculptured, dorsum of promesonotum rugoreticulate, or with irregular longitudinal rugae that only sometimes merge, side of pronotum variably rugulose-punctate, sometimes with a large patch of smooth cuticle, mesopleuron mostly punctate, with a few rugulae, side of propodeum rugulose-punctate, propodeal dorsum and upper half of declivity with a few transverse carinulae; promesonotum in profile low-domed, roughly symmetrical, sometimes dorsum slightly flattened; metanotal groove well-demarcated, moderate; propodeal spines tuberculate, often forming somewhat broad triangles (PSL 0.08–0.14, PSI 1.1–1.4); petiole appearing of moderate length (PL/HW 0.50–0.59), peduncle usually somewhat slender; petiolar node of moderate height to somewhat small (PH/PL 0.54–0.65), dorsum broadly rounded, and always pointing distinctly posteriad, area underneath node usually with a small concavity, or notch; postpetiole in profile subspherical to oblong, appearing slightly compressed anteroposteriorly; anterior faces of petiolar and postpetiolar nodes smooth, remaining surfaces mostly punctate, with a few rugulae on posterior half of postpetiole; gaster smooth, with scattered piligerous punctae; face with a mostly uniform layer of short decumbent setae; remainder of body dorsum with longer suberect setae; pilosity on gastral tergites indistinctly bilayered, with a relatively sparse layer of longer suberect to subdecumbent setae, and a sparser layer of decumbent to appressed setae; setae on scapes and legs mostly decumbent to appressed, with some suberect setae on femoral venters and coxae.

#### Queen description.

(5 measured) HL 0.61–0.76 (0.61), HW 0.56–0.70 (0.57), FLD 0.15–0.21 (0.16), PCW 0.04–0.08 (0.04), SL 0.50–0.64 (0.50), EL 0.15–0.20 (0.15), ACL 0.48–0.61 (0.49), ML 0.84–1.11 (0.86), PrW 0.49–0.63 (0.49), PSL 0.11–0.16 (0.14), SDL 0.09–0.12 (0.10), PL 0.33–0.44 (0.34), PH 0.19–0.25 (0.19), PW 0.17–0.22 (0.18), PPL 0.15–0.22 (0.18), PPH 0.19–0.25 (0.19), PPW 0.18–0.27 (0.21), MFL 0.55–0.73 (0.55), MTL 0.45–0.58 (0.45), CI 89–94 (94), SI 86–96 (86), REL 26–28 (26), FLI 27–31 (27), PSI 1.3–1.4 (1.4), MFI 93–104 (104), ACI1 64–67 (66), ACI2 94–98 (98).

Same as worker except for standard queen modifications and as follows: side of pronotum rugoreticulate to transversely rugose, center of pronotum transversely carinulate; mesoscutum mostly foveolate to longitudinally rugose, usually with some smooth cuticle along midline, foveolae when present piligerous; propodeum with transverse carinulae that wrap around surface; mesopleuron mostly smooth; pilosity on gastral tergites distinctly bilayered, with a sparse layer of longer suberect to subdecumbent setae and a dense layer of decumbent setae, lower layer usually almost pubescent, but sometimes less dense and longer; wing venation as in Figure 133D.

#### Male.

Unknown.

#### Biology.

This species is known only from extracts of sifted leaf litter collected from the forest floor. It occupies a variety of wet forest habitat types (e.g. rainforest, mesophyll forest, 2° wet forest, *Liquidambar* forest, cloud forest, oak-pine forest) and occurs from near sea level to 2000 m. Dealate queens are commonly collected in samples, suggesting that the species may nest in the leaf litter microenvironment.

#### Comments.

*Stenamma nonotch* forms a clade with *Stenamma nanozoi*, *Stenamma sandinista*, and *Stenamma schmidti* and is very similar to these species (Branstetter unpublished data). However, it is the only species within the clade to have the anterior clypeal margin entire, and the basal margin of the mandible straight. This feature makes it easy to distinguish from its close relatives. *Stenamma nonotch* is also similar to *Stenamma ignotum* and *Stenamma picopicucha*, which both have a straight basal margin of the mandible. However, neither of these species have the anterior clypeal margin entire.

There is some variation among populations of *Stenamma nonotch*, with workers differing in size, sculpture, and shape of the propodeal spines and petiolar node. I split this diversity into three variant forms. The type form (Figure 131) includes all of the collections from Oaxaca, Mexico, which were collected below 700 m on the wetter side of the state. Variant 1 (Figure 132A–C) occurs at the low-elevation locality Salto de Agua in Chiapas, Mexico. Compared to the type population, specimens are smaller, have a lighter red-brown coloration, have the propodeal spines slightly more robust and projecting dorsally, and have the petiolar node in profile somewhat anteroposteriorly compressed. Variant 2 (Figure 132A–C) occurs at the mid-elevation locality Nahá in Chiapas, Mexico. It is smaller than the type form and has the propodeal spines noticeably reduced to sharp angles, rather than sharp, projecting tubercles. Lastly, variant 3 (Figure 132D–F) is a high elevation version of *Stenamma nonotch*, occurring mainly at the locality Coapilla in Chiapas, Mexico. Compared with the type form, it is larger, has darker coloration, and has a thicker petiolar node. Also, the ventral margin of the petiolar node is flat, rather than with a small concavity. Molecular data suggest that there is significant divergence among populations, but I have not seen sympatry among forms, and some populations have intermediate phenotypes. Thus, I treat the above as intraspecific variants until more data are available.

#### Material examined.

**MÉXICO: *Chiapas***: 15.1km NW Bochil, [ca. 17.0912°N, 92.9914°W], 1930m, 24 Sep 1992 (R. S. Anderson); 5km NE Coapilla, 17.17558°N, 93.15012°W, 1770m, 26 May 2008 (M. G. Branstetter); 5km NE Coapilla, 17.17602°N, 93.13293°W, 1990m, 25 May 2008 (LLAMA); Lagos de Montebello, Cinco Lagos, 16.1012°N, 91.6740°W, 1600m, 21 Jul 2007 (R. S. Anderson); Lagos de Montebello, L. Pojoj, 16.09913°N, 91.67125°W, 1510m, 21 Jul 2007 (R. S. Anderson); Nahá, 16.96426°N, 91.59234°W, 985m, 8 Jun 2008 (LLAMA); 6km SW Ocosingo, 16.8672°N, 92.0787°W, 1400m, 22 Sep 1991 (R. S. Anderson); 2km S Playón de la Gloria, 16.1385°N, 90.9015°W, 170m, 22 Jul 2007 (R. S. Anderson); 13km N Pueblo Nuevo Solistahuacán, [ca. 17.211°N, 92.964°W], 1860m, 26–27 Aug 1973 (A. F. Newton); 5km E Rayón, 17.217°N, 92.967°W, 1700m, 23 Dec 1991 (P. S. Ward); 8.9km E Rayon, 17.2000°N, 92.9163°W, 1500m, 19 Sep 1991 (R. S. Anderson); 21km SW Salto de Agua, 17.38542°N, 92.42802°W, 180m, 15 Jun 2008 (M. G. Branstetter);***Oaxaca***: 4.3km SW Valle Nacional, 17.74320°N, 96.32729°W, 370m, 13 Aug 2009 (L. Sáenz); 7.5km S Valle Nacional, 17.70752°N, 96.30516°W, 680m, 12 Aug 2009 (M. G. Branstetter); ***Veracruz***: Los Tuxtlas, 10km NNW Sontecomapan, 18.583°N, 95.083°W, 200m, 20 Mar 1985 (P. S. Ward).

### Stenamma ochrocnemis sp. n.

urn:lsid:zoobank.org:act:FC6636D2-7498-4BDE-ABFC-9AF833C613E3

http://species-id.net/wiki/Stenamma_ochrocnemis

**Figure 134.**
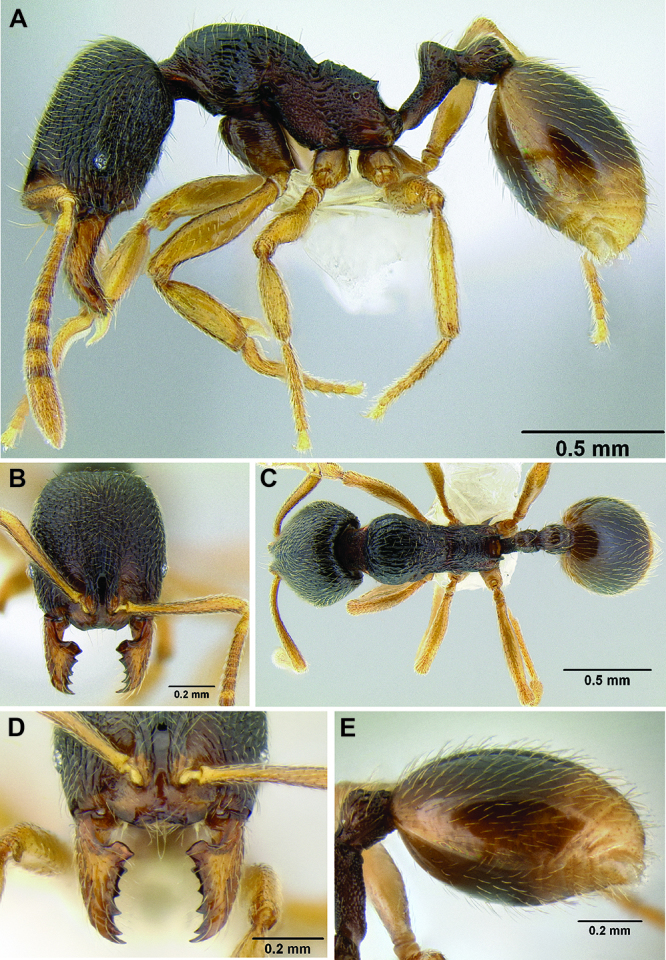
*Stenamma ochrocnemis* holotype worker (CASENT0621468) **A** Profile **B** Face **C** Dorsum **D** Anterior clypeal margin in anterodorsal view **E** Gaster.

**Figure 135.**
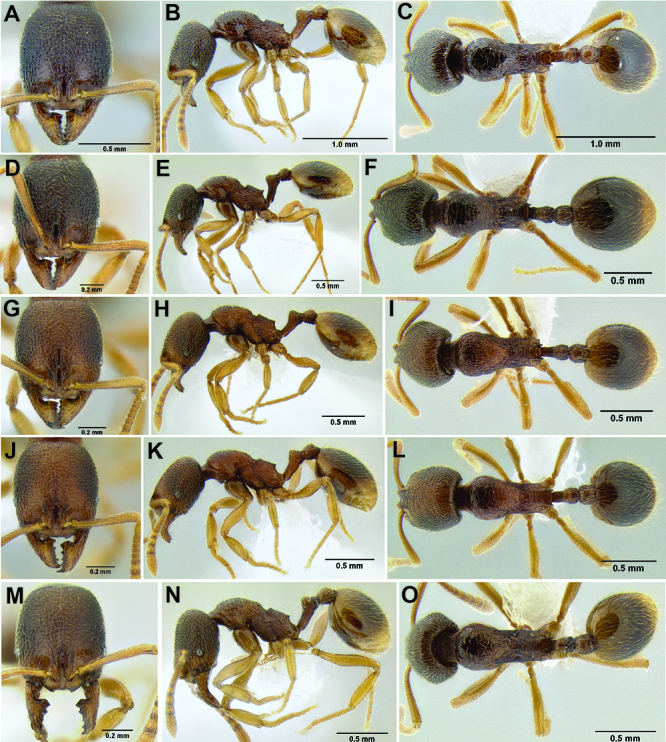
*Stenamma ochrocnemis* worker variants **A–C** Variant 1 (CASENT0603793) **D–F** Variant 2 (CASENT0603884) **G–I** Variant 3 (CASENT0605066), **J–L** Variant 1 (CASENT0605129) **M–O **Variant 4 (CASENT0622160).

**Figure 136.**
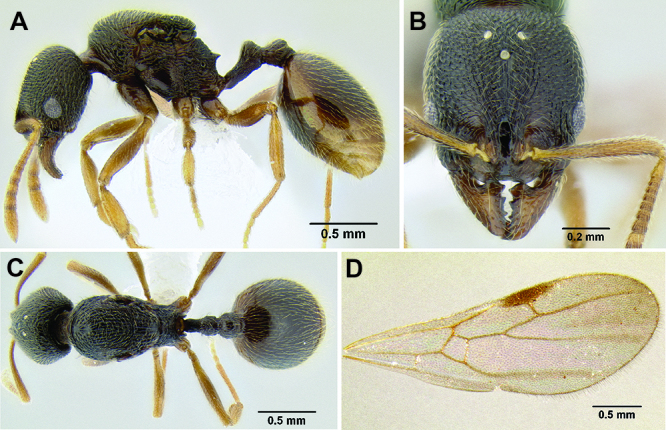
*Stenamma ochrocnemis* **A** Paratype queen (CASENT0621449), profile **B** Same, face **C** Same, dorsum **D** Queen (CASENT0600213), forewing.

**Figure 137.**
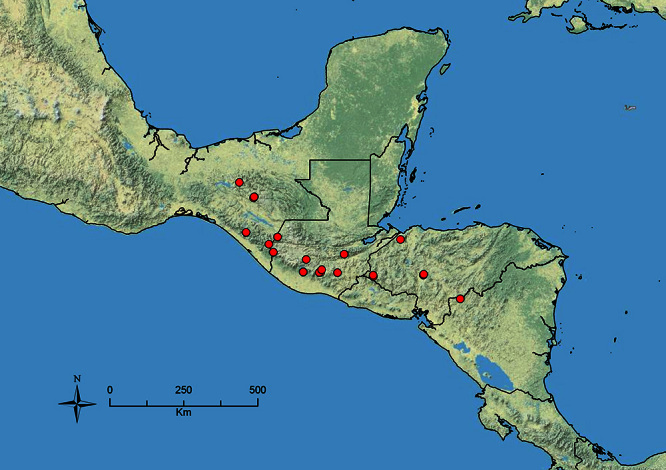
Distribution map of *Stenamma ochrocnemis*.

Worker: Figures 134 , 135 ; Queen: Figure 136 ; Map: Figure 137

Stenamma mgb02 [variant 2 below] Branstetter, 2012: phylogeny.

#### Type material.

*Holotype worker*. HONDURAS, Comayagua: 10km ENE Comayagua, 14.45982°N, 87.54564°W ±20m, 2000m, 15 May 2010, cloud forest, ex sifted leaf litter (LLAMA, collection Wa-C-03-1-11) [USNM, specimen CASENT0621468]. *Paratypes*: same data as holotype but 14.45980°N, 87.54573°W ±20m, 2000m, 15 May 2010 (LLAMA, Wa-C-03-1-09) [1dq, 1w, USNM, CASENT0621449, CASENT0621450], [1w, CAS, CASENT0621451]; 14.45994°N, 87.54523°W ±20m, 2000m, 15 May 2010 (LLAMA, Wa-C-03-1-21) [1dq, 1w, EAPZ, CASENT0621501, CASENT0621502], [1w, ECOSCE, CASENT0621503], [1w, FMNH, CASENT0623437], [1w, ICN, CASENT0623438], [1w, INBio, CASENT0623439], [1w, JTLC, CASENT0623440], [1w, LACM, CASENT0623442], [1dq, 1w, MGBPC, CASENT0623441 CASENT0623443]; 14.46032°N, 87.54512 ±20m, 2000m, 15 May 2010 (LLAMA, Wa-C-03-1-30) [1w, MCZ, CASENT0623444], [1w, MZSP, CASENT0623445], [1w, UCD, CASENT0623446], [1w, UNAM, CASENT0623447], [1w, UVGC, CASENT0623448].

#### Worker diagnosis.

Integument mostly dark red-brown to orange-brown, with appendages a distinctly lighter, uniform yellow-brown; small- to medium-sized species (see HL, ML, PrW below); anterior clypeal margin undulating, usually with 4 sharp to blunt teeth, outer teeth often larger and sharper; basal margin of mandible usually sinuous, with a shallow to very deep basal depression, but without a basal tooth; gastral pilosity usually appearing somewhat dense, with setae subdecumbent to decumbent and not obviously bilayered, but sometimes more clearly bilayered, with subdecumbent setae becoming suberect; head completely sculptured, mostly rugoreticulate; mesosoma usually mostly sculptured with rugae and punctate, but pronotum sometimes with sculpture largely effaced; petiole of moderate length, somewhat robust; eye relatively small (EL 0.07–0.12, REL 12–17), subcircular to oval-shaped, with 4–6 ommatidia at greatest diameter; propodeal spines tuberculate (PSL 0.07–0.14, PSI 1.0–1.4). *Similar species*: *Stenamma catracho*, *Stenamma manni*.

#### Geographic range.

Southern Mexico to Honduras.

#### Worker description.

(20 measured) HL 0.63–0.83 (0.71), HW 0.54–0.73 (0.60), FLD 0.14–0.20 (0.15), PCW 0.02–0.03 (0.02), SL 0.47–0.69 (0.55), EL 0.07–0.12 (0.08), ACL 0.48–0.66 (0.56), ML 0.76–1.09 (0.86), PrW 0.37–0.50 (0.41), PSL 0.07–0.14 (0.10), SDL 0.06–0.10 (0.08), PL 0.28–0.41 (0.33), PH 0.16–0.22 (0.18), PW 0.13-0.18 (0.14), PPL 0.14–0.21 (0.16), PPH 0.14–0.20 (0.16), PPW 0.16–0.22 (0.17), MFL 0.51–0.77 (0.60), MTL 0.43–0.63 (0.50), CI 83–88 (85), SI 82–95 (92), REL 12–17 (13), FLI 25–31 (26), PSI 1.0–1.4 (1.2), MFI 94–110 (100), ACI1 65–70 (69), ACI2 92–104 (101).

Small- to medium-sized species; general body color mostly dark red-brown (almost black) to orange-brown, with head and pronotum darker, and gaster with lighter patches of brown; appendages mostly a uniform and distinctly lighter orange-brown to yellow-brown; setae golden brown; mandible usually with 6–7 teeth (usually 6), but sometimes with 1–3 additional small denticles, middle 2–3 teeth nearest basal tooth often worn and indistinct; basal margin of mandible usually sinuous (rarely straight), with a shallow to very deep basal depression, but without a basal tooth; mandible mostly smooth and shining, with scattered piligerous punctae and striae (mostly on lateral side); anterior clypeal margin usually with 4 sharp to blunt teeth, outer teeth often larger and sharper, sometimes teeth reduced (especially inner two teeth), almost absent, and clypeal margin strongly emarginate; median clypeal lobe with a pair of very faint longitudinal carinulae, and a short transverse carinula near apex, remainder of clypeus smooth and shiny; posterior extension of the clypeus between the antennal insertions narrow (PCW 0.02–0.03), with sides subparallel; frontal lobes usually of moderate width, but somewhat expanded in Cusuco population (FLD 0.14–0.20, FLI 25–31); torular lobes not completely covered in full-face view; frontal carinae blending into head sculpture, not extending beyond about midpoint level of eye; head subrectangular to oval-shaped (CI 83–88), with posterior margin flat to slightly depressed medially; face mostly rugoreticulate, with a few longitudinal rugae along the midline, sometimes sculpture more polished and posterior quarter of head becoming smooth and shiny, interstices faintly punctate; scape of moderate length (SI 82–95), usually not reaching posterior margin in full-face view; scape surface mostly smooth and shiny, with scattered piligerous punctae; funiculus with a somewhat distinct 4-segmented antennal club (ACI2 65–70); pronotum sculpture varying from nearly completely smooth and shiny to strongly rugose (dorsum) and punctate or rugulose (side), with rugae mostly longitudinal in orientation; mesopleuron and propodeal side mostly punctate, with a few rugulae; propodeal dorsum and declivity (to a lesser degree) with punctae and transverse carinulae; promesonotum in profile low-domed, roughly symmetrical; location of promesonotal suture obscured to well-defined, depending on degree of pronotal sculpturing; metanotal grove well-demarcated, of moderate width and depth; propodeal spines tuberculate (PSL 0.07–0.14, PSI 1.0–1.4); petiole of average length (PL/HW 0.51–0.57); petiolar node of moderate to small size (PH/PL 0.53–0.62) and variable in shape, being either wedge shaped and strongly asymmetrical, or subconical, with the apex gently rounded and pointing only slightly posteriad; postpetiole in profile average-looking, about the same size as petiolar node (PPH/PH 0.83–0.96); anterior faces of petiolar nodes smooth and shiny, remaining surfaces of waist mostly punctate, with a few rugulae around nodes; gaster mostly smooth and shiny, with scattered piligerous punctate and a ring of short striae around the anterior constriction; most of body dorsum with somewhat short standing pilosity; scape decumbent to appressed; gastral pilosity variable, usually appearing somewhat dense, with setae subdecumbent to decumbent and not obviously bilayered, but sometimes more clearly bilayered, with subdecumbent setae becoming suberect; setae on legs decumbent to appressed, with suberect to subdecumbent setae on coxae and profemur venter.

#### Queen description.

(5 measured) HL 0.72–0.85 (0.75), HW 0.65–0.76 (0.67), FLD 0.17–0.21 (0.17), PCW 0.03–0.05 (0.05), SL 0.53–0.66 (0.59), EL 0.17–0.20 (0.17), ACL 0.55–0.62 (0.57), ML 1.01–1.26 (1.06), PrW 0.55–0.73 (0.57), PSL 0.13–0.17 (0.15), SDL 0.08–0.12 (0.10), PL 0.37–0.48 (0.39), PH 0.20–0.25 (0.21), PW 0.17–0.22 (0.18), PPL 0.18–0.22 (0.19), PPH 0.19–0.26 (0.20), PPW 0.21–0.29 (0.22), MFL 0.60–0.80 (0.65), MTL 0.53–0.69 (0.56), CI 88–90 (90), SI 82–89 (88), REL 25–28 (25), FLI 26––28 (26), PSI 1.5–1.7 (1.5), MFI 95–107 (103), ACI1 64–68 (67), ACI2 93–106 (96).

Same as worker except for standard queen modifications and as follows: pronotum transversely rugose laterad, becoming smooth mesad; mesoscutum ranging from mostly smooth, with some faint foveolae, to mostly carinulate/rugose (strength of sculpture correlates with pronotum sculpture of worker); scutellum longitudinally rugose, often with a central patch of smooth cuticle; most of katepisternum and sometimes lower half of anepisternum smooth; propodeal spines slightly more developed; setae on mesoscutum subdecumbent to appressed, more dense; pilosity in general slightly more dense; wing venation in Figure 135D.

#### Male.

Unknown.

#### Biology.

*Stenamma ochrocnemis* is known mostly from extracts of sifted leaf litter collected from the forest floor, but workers also have been found at cookie baits and once underneath a rock. The species is a high-elevation specialist occurring from approximately 1600–2750 m, with most collections around 2000 m. It inhabits wet montane forests, such as cloud forest, oak forest, hardwood forest, and mixed oak cloud forest.

#### Comments.

The most distinctive feature of this species is its light colored legs, which always make it stand out from other *Stenamma* species when sorting specimens in ethanol. *Stenamma ochrocnemis* appears superficially similar to several other MAC species, but it should be separable by the characters listed in the diagnosis. Phylogenetic data show that *Stenamma ochrocnemis* belongs to a clade that includes *Stenamma hojarasca* and most likely *Stenamma cusuco* (Branstetter unpublished data).

Across its range there is significant variation in body size, surface sculpture, gastral pilosity and the configuration of the clypeus and basal margin of the mandible. I describe several variants here, but it should be noted that almost every population has some distinctive feature, making separation of this species into discrete forms somewhat arbitrary. In the type population (Figure 135), the body has a very dark red-brown color, the pronotum is strongly rugose, the basal margin of the mandible has a large deep depression, and the anterior clypeal margin forms several sharp teeth. This form is rather distinctive and seems to be restricted to localities in Honduras.

Variant 1 (Figure 135A–C) differs from the type population in that it has the pronotum mostly to completely smooth and shiny, it has lighter body color, and the depression in the basal margin of the mandible is not as deep. This form occurs mainly in Chiapas, Mexico, and Guatemala. Variant 2 (Figure 135D–F) is a slightly different version of variant 1 and it is known from the locality Huitepec in Chiapas, Mexico. Workers are noticeably larger, the propodeal spines are more developed, and the petiole appears elongate, with the node in profile more asymmetrical.

Variant 3 (Figure 135G–I) occurs mainly at the locality 5km SE Antigua in Guatemala, with similar forms at nearby sites. It has the pronotum mostly sculptured, it has lighter orange-brown body color, and it has the basal margin of the mandible only slightly sinuous, almost without a basal depression. Interestingly, this variant co-occurs with variant 1-like specimens (Figure 135J–L) at the Antigua site, suggesting that there may be reproductive isolation between the two forms. I have noticed some ecological differences, with the more sculptured form occurring at slightly lower elevation and in more disturbed habitat than the less sculptured form. Molecular data, however, suggest there is only one species. I have sampled one specimen of each form from the Antigua site, and these are inferred to be sister to each other and nested within the larger *Stenamma ochrocnemis* complex. This result shows that the specimens they are not grouping by sculpture type, but by locality. Additional evidence indicating the existence of only one species is that, specimens with intermediate phenotypes occur at other sites in Guatemala. Thus, I treat this variation as intraspecific.

Variant 4 (Figure 135M–O) is known only from a few specimens collected at Cusuco in Honduras. It is similar to variant 1, except that it has the frontal lobes somewhat dorsolaterally expanded. The form of the frontal lobes is very similar to *Stenamma cusuco* or *Stenamma hojarasca*, which both occur at Cusuco and have strongly expanded frontal lobes. This similarity is either an indication of a shared selection pressure, or perhaps some level of hybridization among species.

I include the specimens from Guerrero, Mexico, in *Stenamma ochrocnemis* with uncertainty. They represent the only collection of the species north of Chiapas, Mexico. Superficially they appear like variant 1, but there are some differences in sculpture and petiole shape, and it is difficult to interpret body and leg color because the specimens are old and faded. Until more material can be collected, I treat this record as dubious.

From the variation described above, there does seem to be a geographic pattern, in which specimens with a smooth pronotum occur in Chiapas, Mexico, both sculptured and unsculptured forms occur in Guatemala, and then mainly sculptured forms occur in Honduras. The degree of reproductive isolation of the different forms/populations is unclear and would be interesting to study. This pattern of morphological variation is similar to that seen in the *Stenamma maximon* complex (see comments section for *Stenamma maximon* above).

#### Material examined.

**GUATEMALA: *El Progresso***: Cerro Pinalón, Finca Las Nubes, 15.07724°N, 89.94785°W, 2350m, 21 Sep 2008 (R. S. Anderson);***Huehuetenango***: 3.3km WSW El Paraíso, 15.53697°N, 91.98158°W, 2350m, 16 Sep 2008 (M. G. Branstetter); San Miguel Chicharro, 4km NNW El Paraíso, 15.57958°N, 91.96916°W, 2100m, 16 Sep 2008 (M. G. Branstetter); ***Jalapa***: 4km E Mataquescuintla, 14.53320°N, 90.15298°W, 2400m, 1 Jun 2009 (LLAMA); Miramundo, Pino Dulce, 14.53388°N, 90.15236°W, 2300m, 18 Sep 2008 (R. S. Anderson);***Quetzaltenango***: 2.9km SSE Chichicastenango, 14.91861°N, 91.10449°W, 2000m, 17 Sep 2008 (M. G. Branstetter); ***Sacatepéquez***: 5km SE Antigua, 14.54155°N, 90.71138°W, 1715m, 12 Jun 2009 (LLAMA); 5km SE Antigua, 14.53666°N, 90.66491°W, 2150m, 10 Jun 2009 (LLAMA); 5km SE Antigua, 14.52799°N, 90.68945°W, 2350m, 10 Jun 2009 (LLAMA); Cerro Alux, 14.61053°N, 90.64191°W, 2190m, 9 Sep 2008 (R. S. Anderson); Cerro Alux, Guatemala City, [ca. 14.6167°N, 90.6333°W], 2260m, 9 Jun 1991 (R. S. Anderson); Cerro Carmona, Finca El Pilar, 14.54115°N, 90.70483°W, 1980m, 9 Sep 2008 (R. S. Anderson); ***Suchitepéquez***: 4km S Vol. Atitlán, 14.54867°N, 91.19089°W, 1625m, 15 Jun 2009 (LLAMA); 4km S Vol. Atitlán, 14.55279°N, 91.19317°W, 1750m, 15 Jun 2009 (LLAMA); **HONDURAS:** ***Comayagua***: 10km ENE Comayagua, 14.45982°N, 87.54564°W, 2000m, 15 May 2010 (LLAMA); 12km ENE Comayagua, 14.48032°N, 87.53240°W, 2140m, 15 May 2010 (LLAMA); 12km ENE Comayagua, 14.48418°N, 87.52934°W, 2270m, 16 May 2010 (LLAMA); ***Cortés***: PN Cusuco, 15.50739°N, 88.23373°W, 2030m, 3 Jun 2010 (LLAMA); ***Ocotepeque***: 13km E Nueva Ocotepeque, 14.45788°N, 89.06812°W, 2190m, 25 May 2010 (LLAMA) 13km E Nueva Ocotepeque, 14.41971°N, 89.06937°W, 2190m, 26 May 2010 (LLAMA); 13km E Nueva Ocotepeque, 14.4224°N, 89.0608°W, 2160m, 26 May 2010 (LLAMA); **MÉXICO: *Chiapas***: Cerro Huitepec (Pico), ca. 5km W San Cristobal, [ca. 16.7500°N, 92.6802°W], 2750m, 18 Sep 1991 (R. S. Anderson); 5km NE Coapilla, 17.17071°N,93.13677°W, 1970m, 27 May 2008 (R. S. Anderson); 2km SE Custepec, 15.72216°N, 92.94298°W, 1820m, 20 May 2008 (R. S. Anderson); 4km SE Custepec, 15.7119°N, 92.92840°W, 2140m, 20 May 2008 (LLAMA); 4km SE Custepec, 15.70777°N, 92.93110°W, 2125m, 20 May 2008 (LLAMA); Huitepec, S. Cristóbal, 16.75149°N, 92.68270°W, 2480m, 29 May 2008 (LLAMA); 7.4km SSW Motozintla de Mendoza, [ca. 15.367°N, 92.233°W], 2000m, 21 Sep 1992 (R. S. Anderson); 7km WSW San Cristóbal, 16.7205°N, 92.7012°W, 2400m, 9 Jul 2007 (M. G. Branstetter); 4km N Union Juarez, Volcan Tacana, lower slopes, [ca. 15.133°N, 92.100°W], 2000m, 19 Sep 1992 (R. S. Anderson); ***Guerrero***: 5.6km SW Filo de Caballo, [ca. 17.6212°N, 99.7469°W], 2310m, 13 Jul 1992 (R. S. Anderson). **Nicaragua:** ***Nueva Segovia***: Cerro Mogotón, 10km NNE Mozonte, 13.75599°N, 86.42062°W, 2010m, 24 Apr 2011 (M. G. Branstetter); 14km NNE Ocotal, 13.75409°N, 86.42094°W, 1900m, 24 Apr 2011 (R. S. Anderson).

### Stenamma pelophilum sp. n.

urn:lsid:zoobank.org:act:0148B1D6-E577-40C2-A94B-2714C8A131F1

http://species-id.net/wiki/Stenamma_pelophilum

**Figure 138.**
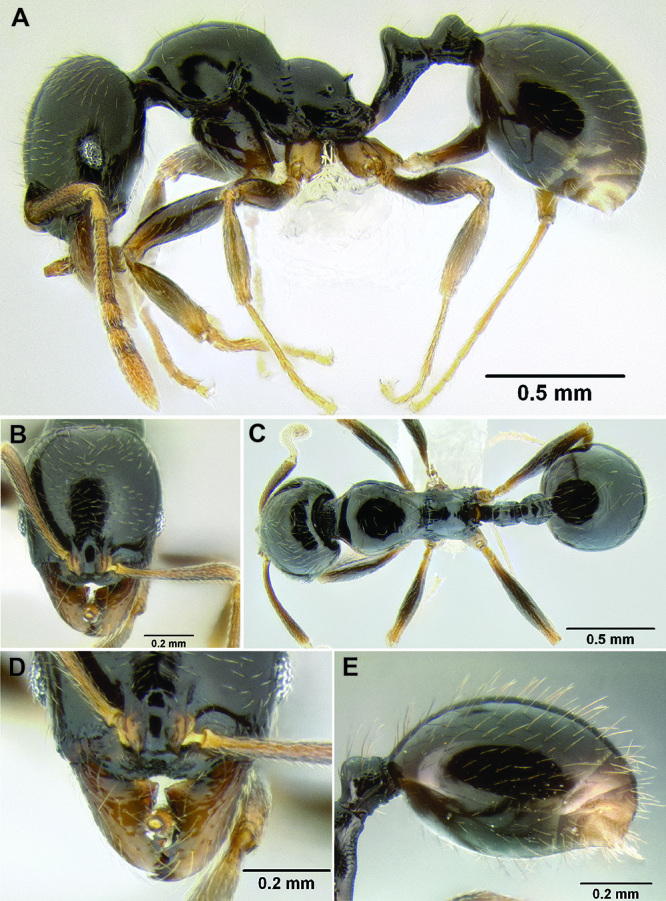
*Stenamma pelophilum* **A** Holotype worker (CASENT0622838), profile **B** Same, face **C** Same, dorsum **D** Same, anterior clypeal margin from anterodorsal view **E** Paratype worker (CASENT0622337), gaster.

**Figure 139.**
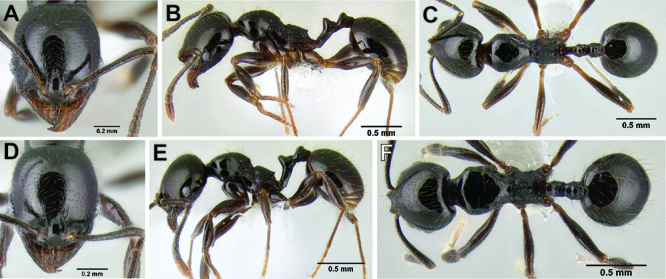
*Stenamma pelophilum* worker variants. Face, profile, dorsal views **A–C** Variant 1 (CASENT0622846) **D–F** Intermediate specimen between variant 1 and type form (CASENT0605516).

**Figure 140.**
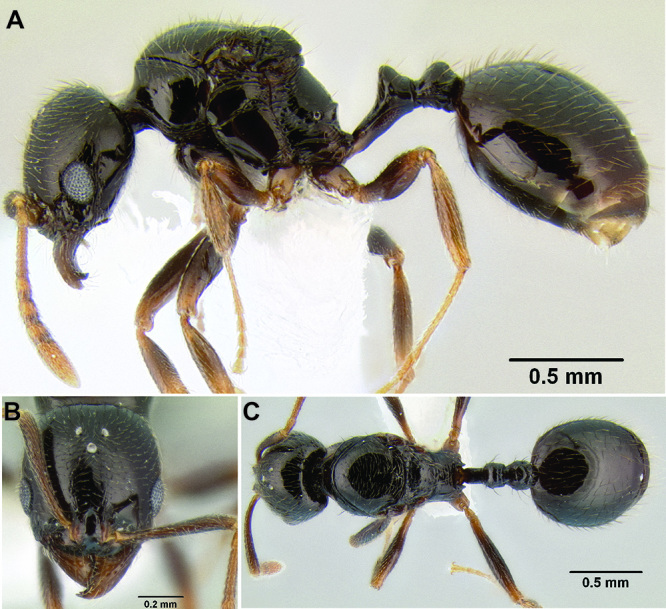
*Stenamma pelophilum* paratype queen (CASENT0622334) **A** Profile **B** Face **C** Dorsum.

**Figure 141.**
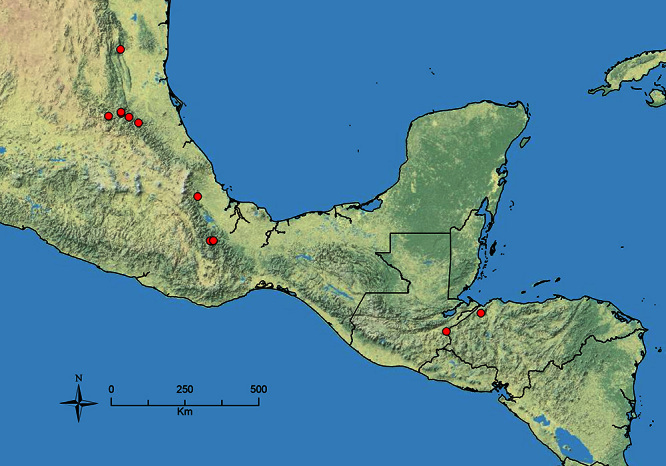
Distribution map of *Stenamma pelophilum*.

Worker: Figures 138 , 139 ; Queen: Figure 140 ; Map: Figure 141

Stenamma mgb27 Branstetter, 2012: phylogeny.

#### Type material.

*Holotype worker*. HONDURAS: Cortés, PN Cusuco, 15.48335°N, 88.22810°W ±50m, 1290m, 2 Jun 2010 (M. G. Branstetter, collection MGB1595) [USNM, specimen CASENT0622838]. *Paratypes*: same data as holotype [1w, CAS, CASENT0623452], [1w, EAPZ, CASENT0622839], [1w, ECOSCE, CASENT0622840], [1w, FMNH, CASENT0622841], [1w, ICN, CASENT0622842], [1w, INBio, CASENT0622843], [2w, JTLC, CASENT0622336], [2w, LACM, CASENT0622335], [2w, MGBPC, CASENT0622337], [1w, MCZ, CASENT0623449], [1w, MZSP, CASENT0623450], [1w, UCD, CASENT0623451], [1w, UNAM, CASENT0623453], [1dq, 2w, USNM, CASENT0622334, CASENT0623455 [1w, UVGC, CASENT0623454].

#### Worker diagnosis.

Integument mostly black to brown-black and shining; small to medium-sized species (see HL, ML, PrW below); anterior margin of clypeus with a shallow median emargination; basal margin of mandible straight to slightly curving, but without a distinct basal notch or depression; head and body almost entirely smooth and shiny, except for faint punctae on waist and sometimes on propodeum; pronotum in profile usually distinctly asymmetrical, with a long, steep posterior face, that forms a distinct angle with dorsal surface, and a short anterior face; eye of moderate size (EL 0.11–0.17, REL 19–23), oval-shaped, with 6–8 ommatidia at greatest diameter; propodeal spines reduced to small tubercles or short points (PSL 0.06–0.10, PSI 1.1–1.7); setae on gastral tergites sparse, moderately long, and mostly suberect; frontal lobes narrow (FLD 0.13–0.18, FLI 24–27), not obscuring torular lobe in full-face view. *Similar species*: *Stenamma llama*, *Stenamma tiburon*.

#### Geographic range.

Mexico (Atlantic slope) to Honduras.

#### Worker description.

(17 measured) HL 0.58–0.83 (0.66) HW 0.51–0.73 (0.58) FLD 0.13–0.18 (0.15), PCW 0.03–0.05 (0.04), SL 0.49–0.76 (0.54), EL 0.11–0.17 (0.13), ACL 0.47–0.65 (0.54), ML 0.74–1.06 (0.86), PrW 0.38–0.47 (0.44), PSL 0.06–0.10 (0.09), SDL 0.05–0.09 (0.07), PL 0.27–0.39 (0.32), PH 0.18–0.24 (0.22), PW 0.12–0.17 (0.15), PPL 0.14–0.21 (0.18), PPH 0.14–0.19 (0.17), PPW 0.14–0.20 (0.18), MFL 0.52–0.86 (0.62), MTL 0.41–0.67 (0.48), CI 86–92 (89), SI 89–107 (92), REL 19–23 (21), FLI 24–27 (26), PSI 1.1–1.7 (1.2), PI 51–58 (54), MFI 83–105 (94), ACI1 64–71 (66), ACI2 84–99 (99)

Small- to medium-sized species; general body color black to brown-black, with mandibles and appendages brown to yellow-brown, lighter toward extremities; setae translucent brown; mandible with 4–6 teeth, consisting of 3 distinct apical teeth, a basal tooth, and 2 inner teeth, which are very often effaced; masticatory margin of mandible sometimes gently curving inward (perhaps due to wear), causing basal tooth to appear more robust; basal margin of mandible straight to slightly curving, without a distinct notch or depression; some specimens with basal section of mandible, where mandible inserts under clypeus, distinctly expanded and thin; dorsal surface of mandible mostly smooth and shining, with scattered piligerous punctae and a few basal striae; median lobe of clypeus somewhat obliquely flattened, and sometimes angled more ventrally than average, creating distinct dorsal and anterior faces; median lobe with faint to absent longitudinal carinulae, middle of lobe often slightly concave, apex of lobe with a short transverse carinula, usually present near anterior margin, remainder of clypeus mostly smooth and shining; posterior extension of clypeus between frontal lobes of moderate width (PCW 0.03–0.05), with subparallel sides; frontal lobes very narrow (FLD 0.13–0.18, FLI 24–27), not obscuring torular lobes in full-face view; head somewhat longer than broad (CI 86–92), roughly oval-shaped, posterior margin flat, never strongly depressed medially, lateral corners gently curving; eyes of moderate size (EL 0.11–0.17, REL 19–23), oval-shaped, with 6–8 ommatidia at greatest diameter; face almost entirely smooth and shiny, except for a variable number of carinulae around frontal carinae and on genae; scape of moderate to somewhat long length (SI 89-107), just reaching or slightly surpassing posterior margin of head when laid back; dorsal surface of scape with scattered piligerous punctae and a few faint striae, otherwise smooth and shining; funiculus with distinct 4-segmented antennal club; mesosoma usually almost entirely smooth and shiny, except for some longitudinal carinulae along metanotal groove, and transverse carinulae on dorsal and declivitous faces of propodeum; in some populations propodeum with more developed rugulae and punctae; promesonotum in profile usually asymmetrically domed and somewhat bulging, with posterior slope distinctly longer and straighter than anterior slope, and forming a more well-defined angle with dorsal surface, apex of promesonotum offset toward posterior margin; lateral and posterior margins of promesonotum in dorsal view, usually well defined, with relatively sharp transitions; one population (from high-elevation) with promesonotum in profile low-domed and roughly symmetrical; propodeal spines forming tubercles, or small projecting points (PSL 0.06–0.10, PSI 1.1–1.7); petiole of moderate length (PL/HW 0.51–0.58), node of variable height (PH/PL 0.59–0.69) and volume, usually pointing slightly posteriad, anterior face longer than posterior face and rising from about midpoint of petiole, posterior face nearly vertical, dorsum in profile usually narrowly rounded, but sometimes forming a defined apex; postpetiole in profile slightly to distinctly smaller than petiolar node (PPH/PH 0.76–0.89), roughly symmetrical, with anterior face slightly longer and more sloping than posterior face; petiole mostly smooth and shining, except for light punctae on venter and lateral portions of peduncle; postpetiole with anterior face smooth, shiny and shield-like, venter and posterior half of postpetiole lightly punctate; gaster mostly smooth and shiny, with scattered piligerous punctae; most of body with a thin layer of moderately long standing setae; setae on gastral tergites sparse and uniformly suberect; facial setae mostly decumbent with a few erect hairs; setae on scape subdecumbent to appressed; setae on legs mostly decumbent to appressed, with coxae and ventral surfaces of the femora with some suberect setae.

#### Queen description.

(3 Measured) HL 0.63–0.69 (0.69), HW 0.58–0.63 (0.63), FLD 0.14–0.16 (0.16), PCW 0.03–0.06 (0.06), SL 0.54–0.56 (0.56), EL 0.17–0.19 (0.18), ACL 0.53–0.56 (0.56), ML 0.91–1.02 (1.02), PrW 0.52–0.56 (0.56), PSL 0.09–0.14 (0.10), SDL 0.08–0.09 (0.09), PL 0.33–0.39 (0.37), PH 0.20–0.24 (0.24), PW 0.17–0.20 (0.19), PPL 0.17–0.20 (0.20), PPH 0.19–0.20 (0.20), PPW 0.19–0.23 (0.22), MFL 0.60–0.67 (0.67), MTL 0.45–0.53 (0.53), CI 91–93 (91), SI 89–92 (89), REL 23–30 (29), FLI 24–26 (26), PSI 1.1–1.7 (1.2), MFI 94–105 (94), ACI1 65–67 (65), ACI2 94–101 (100).

Same as worker except for standard queen modifications and as follows: scutellum with irregular longitudinal rugulae/carinulae; metapleuron with longitudinal carinulae; dorsum of propodeum with transverse carinulae; petiolar node usually same as in workers, but one queen (Huejutla population) with node dorsum in posterior/anterior view forming two sharp points, separated by a steep concavity.

#### Male.

Unknown.

#### Biology.

This is a rare species that inhabits montane wet forests from 1000 to 1800 m elevation. Most collections are from Winkler or Berlese extracts of sifted leaf litter. However, at Cusuco National Park in Honduras, I found two nests in a mud bank bordering a stream. The surface of the bank was rocky with an overlying layer of dense mud. The first nest was located by haphazardly cutting into the bank in an area where I saw lots of worker activity. This nest had a single chamber with about 35 workers and some brood. The second nest was located by following a returning forager. This nest had a nearly undetectable entrance (2 mm diameter) consisting only of a small hole, without any surrounding structure or excavated substrate. Behind the entrance was a very short tunnel leading to a single, small chamber. This contained 46 workers, some brood and a single dealate queen. When the nest chamber was disturbed, the queen immediately absconded out onto the clay bank followed by a series of workers carrying brood. Before excavation, foragers outside the nest were observed to be slow moving and solitary, similar to behavior in most other *Stenamma* species.

#### Comments.

Molecular phylogenetic data show that *Stenamma pelophilum* is sister to the *lobinodus* species group, which includes *Stenamma llama*, *Stenamma lobinodus*, and *Stenamma tiburon* (Branstetter unpublished data). Although *Stenamma pelophilum* does not have the distinguishing features of this group, it does bear some similarity to *Stenamma llama* and *Stenamma tiburon*. It can be easily separated from these species by its distinctive promesonotum shape and average looking postpetiole, which lacks a distinctive dorsal lobe.

There is minimal morphological variation amongst most populations of *Stenamma pelophilum*, and I interpret this variation to be intraspecific. One population, however, is significantly divergent from the type population and I describe it here as variant 1 (Figure 139A–C). This variant is known from above 2000 m in Oaxaca, Mexico, on the wet, eastern slope of the Sierra Juarez. It is larger, has more sculpture on the mesopleuron and propodeum, and has the promesonotum in profile low-domed and roughly symmetrical. Also, it has a relatively longer scape and metafemur than other populations of standard *Stenamma pelophilum* (SI ≥ 100 vs. ≤ 96, MFI ≤ 86 vs. ≥ 93). It could be that this variant is a mountaintop endemic, derived from the *Stenamma pelophilum* populations found lower on the mountain. I choose, however, to include variant 1 within *Stenamma pelophilum*, because I have found specimens with intermediate phenotypes (Figure 139D–F). These have been collected from intermediate elevations, and from the same samples as variant 1. The morphological differences might be due to environmental effects on development, rather than reproductive isolation and divergence. It would be interesting to investigate the population dynamics between these two forms along the elevational gradient.

#### Material examined.

**GUATEMALA:** ***Zacapa***: 2km SE La Unión, 14.94681°N, 89.27583°W, 1550m, 12 May 2009 (LLAMA); **HONDURAS:** ***Cortés***: PN Cusuco, 15.48335°N, 88.22810°W, 1290m, 2 Jun 2010 (M. G. Branstetter); **MÉXICO: *Hidalgo***: 43km SW Huejutla, [ca. 20.988°N, 98.662°W], 14 Jun 1983 (S. & J. Peck); ***Oaxaca***: 20.6km SW Valle Nacional, 17.60404°N, 96.37786°W, 1730m, 13 Aug 2009 (M. G. Branstetter); 26km SW Valle Nacional, 17.58667°N, 96.44948°W, 2160m, 11 Aug 2009 (M. G. Branstetter); 27km SW Valle Nacional, 17.59582°N, 96.47572°W, 2290m (M. G. Branstetter); 27.4km SW Valle Nacional, 17.59635°N, 96.47449°W, 2280m, 12 Jun 2009 (M. G. Branstetter); 47.5km SW Valle Nacional, km100.5, [ca. 17.590°N, 96.410°W], 2125m, 26 Jul 1992 (R. S. Anderson); ***Querétaro***: 7km NE Pinal de Amoles, 21.17601°N, 99.57341°W, 1700m, 18 Aug 2009 (M. G. Branstetter); ***San Luis Potosí***: Taman, 20km SW Tamazunchale, [ca. 21.153°N, 98.947°W], 11 Jun 1983 (S. & J. Peck); 20km W Xilitla, [ca. 21.293°N, 99.194°W], 1600m, 12 Jun 1983 (S. & J. Peck); 40km W Xilitla, [ca. 21.259°N, 99.194°W], 1700m, 12 Jun 1983 (S. & J. Peck); ***Tamaulipas***: Sa. de Guatemala, Rancho del Cielo, [ca. 23.062°N, 99.209°W], 1070m, 4 Jul 1969 (S. & J. Peck); ***Veracruz***: Cordoba, Paraje Nuevo, Nacimiento, [ca. 18.88°N, 96.86°W], 7 Aug 1969 (S. & J. Peck).

### Stenamma picopicucha sp. n.

urn:lsid:zoobank.org:act:343076E5-D041-4CCF-9DF8-41CC4C3C275E

http://species-id.net/wiki/Stenamma_picopicucha

**Figure 142.**
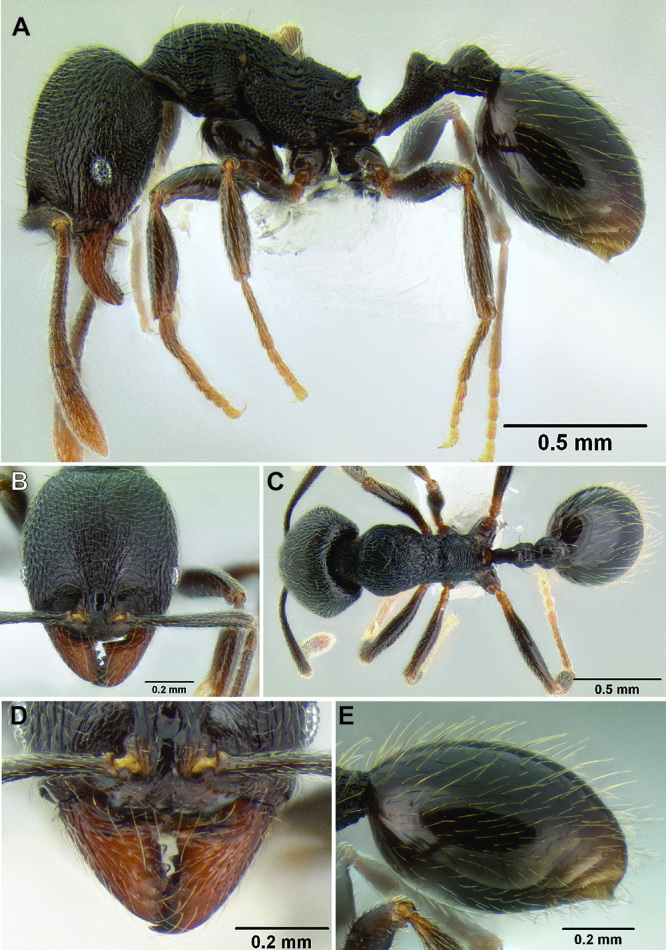
*Stenamma picopicucha* holotype worker (CASENT0621347) **A** Profile **B** Face **C** Dorsum **D** Anterior clypeal margin in anterodorsal view **E** Gaster.

**Figure 143.**
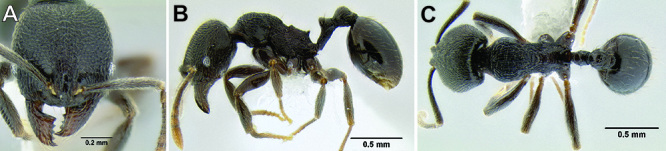
*Stenamma picopicucha* worker variant (CASENT0622155) **A** Face **B** Profile **C** Dorsum.

**Figure 144.**
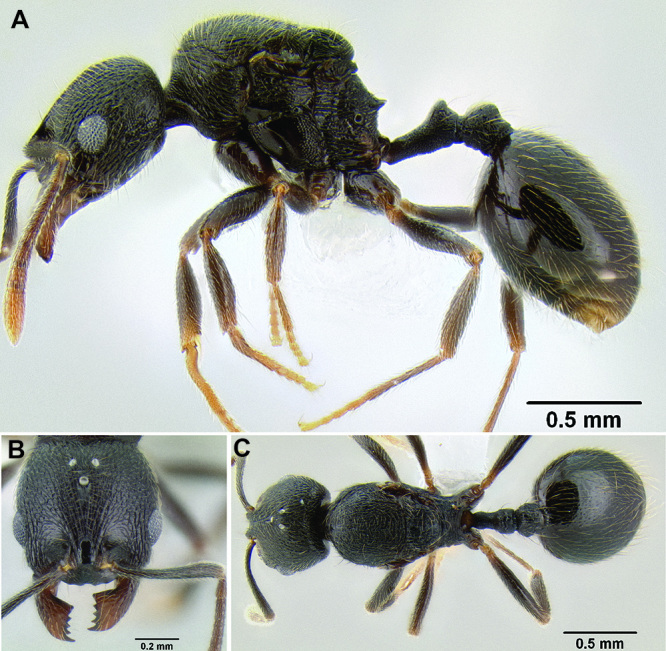
*Stenamma picopicucha* paratype queen (CASENT0621374) **A** Profile **B** Face **C** Dorsum.

**Figure 145.**
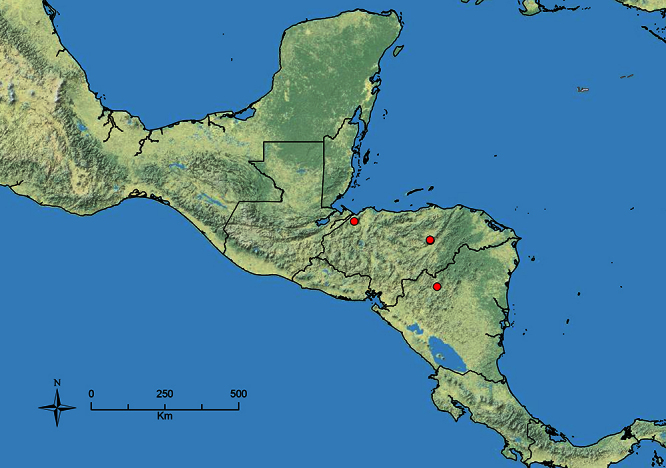
Distribution map of *Stenamma picopicucha*.

Worker: Figures 142 , 143 ; Queen: Figure 144 ; Map: Figure 145

#### Type material.

*Holotype worker*. HONDURAS,Olancho: 11km N Catacamas, 14.94839°N, 85.91477°W ±20m, 2020m, 8 May 2010, cloud forest, ex sifted leaf litter (LLAMA, collection Wa-C-02-1-03) [USNM, specimen CASENT0621356]. *Paratypes*: same data as holotype [1w, CAS, CASENT0621355], [1w, EAPZ, CASENT0621357], [1w, ECOSCE, CASENT0621358], [1w, FMNH, CASENT0623456], [1w, ICN, CASENT0623457], [1w, INBio, CASENT0623458], [1w, JTLC, CASENT0623459], [1w, LACM, CASENT0623461], [1w, MGBPC, CASENT0623462], [1w, MCZ, CASENT0623463], [1w, MZSP, CASENT0623464], [1w, UCD, CASENT0623465], [1w, UNAM, CASENT0623467], [1w, UVGC, CASENT0623468]; same data but 14.94879°N, 85.91486°W ±20m, 2020m, 8 May 2010 (LLAMA, Wa-C-02-1-12) [1dq, 2w, USNM, CASENT0621374, CASENT0621372, CASENT0621373],

#### Worker diagnosis.

Integument mostly dark brown to brown; small-sized species (see HL, ML, PrW below); basal margin of mandible straight, without a basal notch or depression; anterior clypeal margin forming two blunt teeth, which border the midline; face completely sculptured, mostly rugoreticulate; mesosoma completely sculptured, pronotum longitudinally rugose; pilosity on gastral dorsum bilayered, with a layer of long suberect setae, and a layer of very sparse decumbent setae; eye of moderate size (EL 0.10–0.12, REL 17–20), oval-shaped, with 5–6 ommatidia at greatest diameter; propodeal spines tuberculate to short (PSL 0.08–0.10, PSI 1.3–1.6). *Similar species*: *Stenamma crypticum*, *Stenamma huachucanum*, *Stenamma ignotum*.

#### Geographic range.

Honduras to Nicaragua.

#### Worker description.

(11 measured) HL 0.59–0.69 (0.69), HW 0.52–0.64 (0.64), FLD 0.14–0.15 (0.15), PCW 0.03–0.05 (0.05), SL 0.46–0.55 (0.55), EL 0.10–0.12 (0.12), ACL 0.45–0.53 (0.53), ML 0.71–0.84 (0.84), PrW 0.37–0.42 (0.42), PSL 0.08–0.10 (0.09), SDL 0.06–0.07 (0.07), PL 0.25–0.30 (0.30), PH 0.16–0.19 (0.19), PW 0.13–0.15 (0.15), PPL 0.14–0.18 (0.18), PPH 0.16–0.18 (0.18), PPW 0.16–0.19 (0.19), MFL 0.48–0.59 (0.59), MTL 0.41–0.51 (0.51), CI 89–94 (93), SI 85–89 (86), REL 17–20 (18), FLI 23–28 (23), PSI 1.3–1.6 (1.5), MFI 104–109 (108), ACI1 67–70 (69), ACI2 96–101 (96).

Small-sized species; general body color mostly dark brown to brown, with appendages brown to orange-brown, lighter at joints and toward extremities; setae golden brown; mandible with 6 teeth, inner two teeth near basal tooth, sometimes worn; basal margin of mandible straight, without a basal notch or depression; mandible surface mostly smooth, with scattered piligerous punctae and some faint striations on base and lateral surface; anterior clypeal margin forming 2 blunt teeth, which border the midline; median lobe of clypeus with a pair of faint longitudinal carinulae, which diverge anteriorly, apex of lobe with a short transverse carinula, area in between lobe and anterior clypeal margin forming a distinct cavity, remainder of clypeal surface smooth; posterior extension of clypeus between antennal insertions of moderate width (PCW 0.03–0.05), with sides subparallel; frontal lobes of moderate width (FLD 0.14–0.15, FLI 24–28), not greatly covering torular lobes in full-face view; head subrectangular (CI 89–94), posterior margin with a slight median depression; eye of moderate size (EL 0.10–0.12, REL 17–20), oval-shaped, with 5–7 ommatidia at greatest diameter; face mostly rugoreticulate, with some longitudinal carinulae along midline; scape somewhat short (SI 85–89), not quite reaching posterior margin of head when laid back; scape surface mostly smooth, with scattered piligerous punctae; flagellum with a somewhat distinct 4-segmented antennal club; mesosoma completely sculptured; pronotal dorsum longitudinally rugose, becoming rugoreticulate-punctae on mesonotal dorsum; side of pronotum rugose to rugose-punctate; katepisternum and side of propodeum mostly punctate, with a few rugulae on propodeum; propodeal dorsum mostly punctate, with a few transverse carinulae; propodeal declivity with transverse carinulae; promesonotum in profile slightly asymmetrical, with the anterior face long and steeper than posterior face; metanotal groove relatively distinct, but anterior slope transitioning smoothly into promesonotum; propodeal spines tuberculate (PSL 0.08–0.10, PSI 1.3–1.6); petiole in profile short (PL/HW 0.46–0.50); petiolar node usually small, somewhat dorsoventrally compressed (PH/PL 0.60–0.68), node asymmetrical in profile, anterior face longer and more sloping than nearly vertical posterior face, dorsum of node narrowly rounded, and pointing slightly posteriad; postpetiolar node in profile usually similar in size to petiolar node (type population), but sometimes bulging (PPH/PH 0.90–1.01); petiole and postpetiole usually mostly punctate, with only the anterior faces of the nodes smooth, but sometimes nodes mostly smooth; most of body dorsum with short to moderately long pilosity; pilosity on gastral dorsum bilayered, with a layer of long suberect setae, and a much sparser layer of short decumbent setae; setae on scape subdecumbent to appressed; setae on legs decumbent to appressed, with longer suberect setae on femoral venters and coxae.

#### Queen description.

(5 measured) HL 0.63–0.71 (0.64), HW 0.60–0.67 (0.61), FLD 0.16–0.17 (0.17), PCW 0.04–0.05 (0.03), SL 0.49–0.56 (0.47 (0.47), EL 0.17–0.20 (0.18), ACL 0.47–0.53), ML 0.91–1.05 (0.93), PrW 0.52–0.61 (0.54), PSL 0.12–0.14 (0.12), SDL 0.08–0.10 (0.09), PL 0.31–0.36 (0.32), PH 0.18–0.21 (0.19), PW 0.15–0.18 (0.16), PPL 0.17–0.21 (0.19), PPH 0.19–0.22 (0.19), PPW 0.20–0.24 (0.21), MFL 0.54–0.65 (0.55), MTL 0.46–0.56 (0.46), CI 94–96 (95), SI 81–84 (78), REL 29–30 (29), FLI 24–27 (28), PSI 1.4–1.6 (1.4), MFI 104–110 (109), ACI1 67–69 (68), ACI2 94–98 (101).

Same as worker except for standard queen modifications and as follows: pronotum with transverse carinulae; mesoscutum and scutellum carinulae; mesopleuron mostly smooth and shiny; propodeum transversely carinulate, with carinulae wrapping around surface; decumbent layer of setae on dorsum of gaster more dense.

#### Male.

Unknown.

#### Biology.

*Stenamma picopicucha* occurs from 1400–2300 m elevation, and is a specialist of montane wet forest habitats, such as cloud forest and dwarf forest. Most collections of this species are from samples of sifted leaf litter taken from the forest floor, but a few specimens are known from cookie bait cards. Nests have never been found, but dealate queens are common in the leaf litter, suggesting that nests might occur within this stratum.

#### Comments.

*Stenamma picopicucha* looks like a cross between *Stenamma crypticum* and *Stenamma ignotum*. The petiole and anterior clypeal margin are more like *Stenamma crypticum*, but the basal margin of the mandible and gastral pilosity are more similar to *Stenamma ignotum*. I originally intended to lump this species in with *Stenamma crypticum* until I noticed that the two species occur in sympatry near the peak at Cusuco in Honduras. It is interesting to note that this mountaintop population at Cusuco has the postpetiole bulging and smooth; as a consequence, I label this form variant 1 (Figure 143). This population is most likely the same as *Stenamma picopicucha* based on mandible and clypeus structure. More normal-looking specimens occur at lower elevation on the mountain.

#### Material examined.

**HONDURAS: *Cortés***: 25km N Cofradia, PN Cusuco, [ca. 15.497°N, 88.227°W], 1550m, 26 Aug 1994 (S. & J. Peck); PN Cusuco, 15.48965°N, 88.23383°W, 1300m, 31 May 2010 (LLAMA); PN Cusuco, 15.50739°N, 88.23373°W, 3 Jun 2010 (LLAMA);***Olancho***: 9km N Catacamas, 14.93806°N, 85.90685°W, 1430m, 10 May 2010 (R. S. Anderson); 10km N Catacamas, 14.94402°N, 85.91081°W, 1670m, 9 May 2010 (LLAMA); 11km N Catacamas, 14.94870°N, 85.91484°W, 2020m, 8 May 2010 (LLAMA); 12km N Catacamas, 14.95592°N, 85.91681°W, 2260m, 9 May 2010 (LLAMA); **NICARAGUA:** ***Jinotega***: RN Cerro Kilambé, 13.56933°N, 85.69745°W, 1500m, 23 May 2011 (LLAMA).

### Stenamma saenzae sp. n.

urn:lsid:zoobank.org:act:1FABD241-F9CB-4826-A078-5833D7F09F15

http://species-id.net/wiki/Stenamma_saenzae

**Figure 146.**
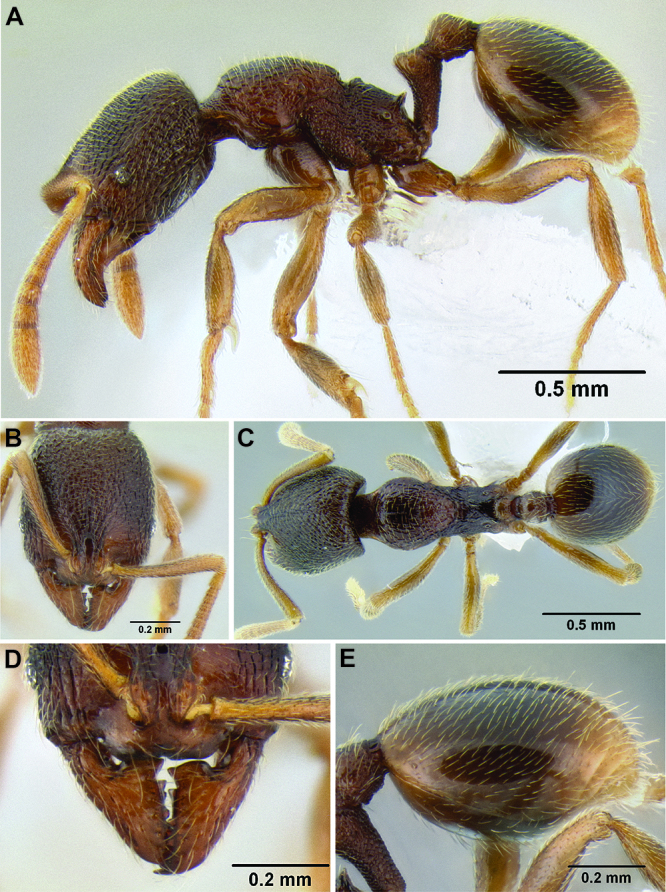
*Stenamma saenzae* holotype worker (CASENT0603860) **A** Profile **B** Face **C** Dorsum **D **Anterior clypeal margin in anterodorsal view **E** Gaster.

**Figure 147.**
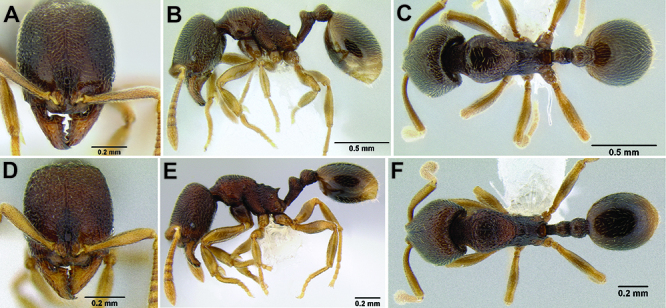
*Stenamma saenzae* worker variants. Face, profile, and dorsal views **A–C** Variant 1 (CASENT0604912) **D–F** Variant 2 (CASENT0621764).

**Figure 148.**
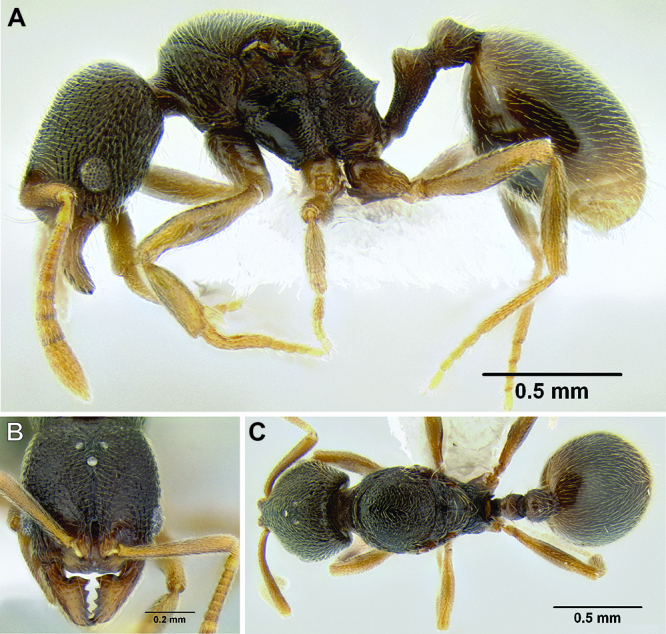
*Stenamma saenzae* paratype queen (CASENT0603866) **A** Profile **B** Face **C** Dorsum.

**Figure 149.**
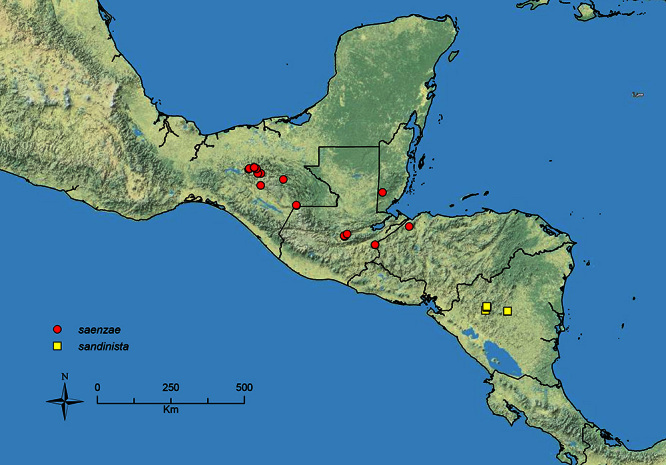
Distribution map of *Stenamma saenzae* (circles) and *Stenamma sandinista* (squares).

Worker: Figures 146 , 147 ; Queen:Figure 148 ; Map: Figure 149

#### Type material.

*Holotype worker*. MÉXICO, Chiapas: 5km NE Coapilla, 17.17550°N, 93.13212°W, 1990m, 25 May 2008, 2° mesophyll forest, ex sifted leaf litter (LLAMA, collection Wa-A-04-1-26) [USNM, specimen CASENT0603860]. *Paratypes*: same data as holotype but 17.18330°N, 93.15209°W ±50m, 1915m, 25 May 2008 (LLAMA, Wa-A-04-2-07) [1dq, 1w, CAS, CASENT0623473, CASENT0623474], [1w, EAPZ, CASENT0623475], [1w, ECOSCE, CASENT0623476], [1w, FMNH, CASENT0623477], [1w, ICN, CASENT0623478]; 17.17550°N, 93.13212°W ±50m, 1990m, 25 May 2008 (LLAMA, Wa-A-04-2-06) [1w, INBio, CASENT0623469], [1w, JTLC, CASENT0623470], [1w, LACM, CASENT0623471], [1w, MGBPC, CASENT0623472]; 17.18296°N, 93.15197°W ±50m, 1915m, 25 May 2008 (LLAMA, Wa-A-04-2-15) [1dq, 1w, MCZ, CASENT0623479, CASENT0623480], [1w, MZSP, CASENT0623481], [1w, UCD, CASENT0623482], [1w, UNAM, CASENT0623483], [1w, UVGC, CASENT0623484], [1w, USNM, CASENT0623485]; 17.18273°N, 93.15184°W ±50m, 1915m, 25 May 2008 (LLAMA, Wa-A-04-2-21) [1dq, USNM, CASENT0603866]

#### Worker diagnosis.

Integument mostly brown to orange-brown and mottled; small-sized species (see HL, ML, PrW below); basal margin of mandible sinuous, always with a basal notch containing a small tooth; anterior clypeal margin undulating, often forming 4 blunt teeth; face completely sculptured, usually mostly rugoreticulate; gastral pilosity with a dense layer of short decumbent to appressed setae, and a sparse layer of longer suberect setae; eye small (EL 0.05–0.09, REL 10–16), subcircular, with 3–5 ommatidia at greatest diameter; posterior extension of clypeus between antennal insertions narrow (PCW 0.01–0.02), with inner margins of frontal lobes almost touching anteriorly; scape short (SI 81–92), not reaching posterior margin of head when laid back; propodeal spines tuberculate to short (PSL 0.07–0.14, PSI 1.5–2.3). *Similar species*: *Stenamma crypticum*, *Stenamma excisum*, *Stenamma nanozoi*.

#### Geographic range.

Southern Mexico to Honduras.

#### Worker description.

(16 measured) HL 0.52–0.69 (0.65), HW 0.43–0.59 (0.54), FLD 0.11–0.14 (0.14), PCW 0.01–0.02 (0.02), SL 0.36–0.50 (0.50), EL 0.05–0.09 (0.07), ACL 0.37–0.49 (0.47), ML 0.60–0.82 (0.77), PrW 0.30–0.41 (0.38), PSL 0.07–0.14 (0.10), SDL 0.04–0.07 (0.06), PL 0.23–0.31 (0.29), PH 0.14–0.18 (0.17), PW 0.11–0.14 (0.14), PPL 0.12–0.19 (0.16), PPH 0.12–0.17 (0.17), PPW 0.14–0.19 (0.17), MFL 0.39–0.53 (0.51), MTL 0.32–0.44 (0.41), CI 84–89 (84), SI 81–92 (92), REL 10–16 (13), FLI 22–27 (26), PSI 1.5–2.3 (1.6), MFI 107–119 (107), ACI1 69–73 (70), ACI2 94–105 (94).

Small-sized species; general body color a mottled dark brown to light orange-brown, with mandibles and appendages lighter, usually orange-brown to yellow-brown; setae golden brown; mandible with 6–7 teeth (usually 6), consisting of 2–3 distinct apical teeth, a distinct, usually well-defined basal tooth, and 2–3 inner teeth, which are sometimes worn and indistinct; basal margin of mandible sinuous and always with a small basal notch containing a tooth; mandible mostly smooth and shining, with scattered piligerous punctae and basal striae; anterior clypeal margin viewed at an anterodorsal angle weakly to strongly undulating (appearing nearly flat in full-face view), often forming 4 blunt teeth, median undulation (emargination) sometimes narrow and notch-like; median lobe of clypeus lacking a distinct pair of longitudinal carinulae, either completely smooth, or with faint irregular striations (type population), apex of lobe with a transverse carina, remainder of clypeus mostly smooth and shiny; posterior extension of clypeus between antennal insertions very narrow (PCW 0.01–0.02), with sides subparallel and inner margins of frontal lobes almost touching anteriorly; frontal lobes of moderate width (FLD 0.11–0.14, FLI 22–27), not obscuring torular lobes in full-face view; head appearing subrectangular (CI 84–89), with posterior margin depressed medially; eyes small (EL 0.05–0.09, REL 10–16), subcircular to slightly oblong, with 3–5 ommatidia at greatest diameter; face densely sculptured, usually mostly rugoreticulate, with longitudinal rugae medially, but sometimes reticulae less distinct and interconnected, interstices lightly punctate; scape relatively short, not reashing posterior margin of head when laid back (SI 81–92), usually of average thickness (type population), but some populations with scape distinctly swollen distally; scape cuticle mostly smooth and somewhat shiny, with scattered piligerous punctae; flagellum with somewhat distinct 4-segmented antennal club, apical segment noticeably enlarged; sculpture on mesosoma variable among populations, lateral surface usually weakly to strongly punctate, with variable number of longitudinal rugulae, dorsal surface of promesonotum variably rugulose-punctate, with pronotum ranging from completely smooth to strongly sculptured, most populations intermediate (type population), pronotum sometimes with a distinct longitudinal carina; propodeal declivity smooth and shiny or with a few transverse carinulae; promesonotum in profile low-domed and roughly symmetrical (type population), or less often flattened and more asymmetrical, with anterior face distinctly longer than posterior; propodeal spines tuberculate or forming short, broad triangular spines (PSL 0.07–0.14, PSI 1.5–2.3); metanotal grove usually well demarcated, of moderate depth and width; petiole of moderate length (PL/HW 0.47–0.56), average-looking; node in profile somewhat small (PH/PL 0.57–0.64), subconical, with anterior face slightly longer and more sloping than posterior face, node dorsum in profile rounded, pointing vertical to slightly posteriad; postpetiole in profile subcircular, usually appearing similar in size to petiolar node (type population), but sometimes slightly larger and more bulging (PPH/PH 0.86–1.08); petiole and postpetiole lightly to somewhat strongly punctate, with nodes variably smooth and shiny; gaster mostly smooth and shiny, with scattered piligerous punctae; most of body dorsum with very short suberect to decumbent pilosity; scapes with a dense layer of decumbent to appressed setae; gastral pilosity consisting of a dense decumbent (type population) to appressed layer of setae, and a much sparser layer of suberect setae, which is sometimes difficult to see among decumbent setae; setae on legs mostly appressed, with a few suberect setae on coxae and femoral venters.

#### Queen description.

(5 measured) HL 0.56–0.67 (0.66), HW 0.50–0.59 (0.59), FLD 0.12–0.15 (0.15), PCW 0.02–0.03 (0.03), SL 0.41–0.51 (0.51), EL 0.13–0.15 (0.15), ACL 0.41–0.51 (0.51), ML 0.77–0.92 (0.91), PrW 0.43–0.52 (0.52), PSL 0.10–0.11 (0.10), SDL 0.06–0.08 (0.07), PL 0.27–0.32 (0.32), PH 0.16–0.19 (0.19), PW 0.14–0.16 (0.16), PPL 0.15–0.19 (0.19), PPH 0.16–0.19 (0.19), PPW 0.17–0.21 (0.21), MFL 0.44–0.55 (0.54), MTL 0.37–0.46 (0.46), CI 84–91 (89), SI 80–90 (86), REL 25–26 (26), FLI 24–27 (25), PSI 1.3–1.8 (1.5), MFI 103–115 (108), ACI1 69–72 (69), ACI2 95–100 (100).

Same as worker except for standard queen modifications and as follows: pronotum transversely rugose to rugoreticulate laterad, becoming punctate mesad; mesoscutum with narrow strip of smooth cuticle extending from anterior margin to about midpoint along midline; mesopleuron mostly smooth and shiny; propodeum mostly with transverse carinulae that wrap around surface, or less often mostly punctate; propodeal spines smaller (PSL 0.10–0.11, PSI 1.3–1.8).

#### Male.

Unknown.

#### Biology.

*Stenamma saenzae* is known almost exclusively from extracts of sifted leaf litter, with only a few queens collected from flight intercept traps in Belize. It is restricted to montane wet forest environments (e.g. cloud forest, mesophyll forest, pine cloud forest, oak-pine forest, liquidambar-oak-pine forest) and has been collected from 1000–2100 m elevation. Dealate queens as well as workers are commonly collected from leaf litter, suggesting that nests might be located within this stratum.

#### Comments.

Based on overall size and form, *Stenamma saenzae* might be confused with *Stenamma crypticum*, *Stenamma nanozoi*, or *Stenamma excisum*, but out of these species, *Stenamma nanozoi* is the only species to share the small tooth on the basal margin of the mandible. *Stenamma saenzae* can be easily separated from *Stenamma nanozoi* by comparing gastral pilosity, which in the latter species, is composed mainly of a sparse layer of thickened suberect setae. Tentative molecular phylogenetic results show *Stenamma saenzae* as sister to a clade that includes *Stenamma catracho*, *Stenamma crypticum*, and *Stenamma monstrosum* (Branstetter unpublished data).

*Stenamma saenzae* shows considerable morphological variability among populations. From this variation, I describe two variants that differ significantly from the type phenotype (indicated above). Variant 1 (Figure 147A–C) occurs mainly in central Guatemala (Biotopo Quetzal, Purulhá) and has the following distinguishing features: pronotal dorsum smooth and shiny; postpetiole more bulging, appearing larger than petiolar node; head somewhat enlarged. Although distinct from the type population, I find intermediate phenotypes at intervening localities, causing me to conclude that this variation is probably intraspecific, and likely due to environmental effects on size and sculpture. Supporting this view is the observation that the locality Biotopo Quetzal, where variant 1 is most distinct, is an especially cool, wet cloud forest site.

Variant 2 (Figure 147D–F) is known from the localities La Unión in eastern Guatemala and Cusuco in northwestern Honduras, and it has the following distinguishing character states: overall body size distinctly smaller; antennal scapes thickened distally; mesosoma and waist strongly punctate, with rugulae reduced; promesonotum in profile flatter, and more asymmetrical; propodeal spines larger and more broadly triangular; lower layer of gastral setae appressed (rather than decumbent), allowing the suberect layer to be clearly visible. Unlike variant 1, I do not find intermediate phenotypes between variant 2 and the other forms, suggesting that this variant is more isolated and perhaps represents a distinct species. Because this variant is not sympatric with the other forms, I choose to include it within *Stenamma saenzae* until more data become available. Preliminary molecular results show specimens from La Unión and Cusuco to be sister taxa, nested inside the larger *Stenamma saenzae* clade.

#### Material examined.

**BELIZE:** ***Cayo***: Chiquibul N.P., Doyle’s Delight, 16.49194°N, 89.04444°W, 1000m, 25–28 Aug 2007 (P. W. Kovarik); Chiquibul N.P., Doyle’s Delight, 16.49305°N, 89.04694°W, 1100m, 19–28 Aug 2007 (P. W. Kovarik); **GUATEMALA: *Baja Verapaz***: Biotopo Quetzal, 15.21307°N, 90.21512°W, 1750m, 7 May 2009 (LLAMA), Biotopo Quetzal, 15.2142°N, 90.2163°W, 1680m, 8 Jul 2007 (M. G. Branstetter); Purulhá, Biotopin, 15.21535°N, 90.21618°W, 1698m, 26–30 Mar 2008 (Méndez et al.); 7.3km E Purulhá, [ca. 15.2667°N, 90.132°W], 1700m, 19 May 1991 (R. S. Anderson); 8km S Purulhá, [ca. 15.1944°N, 90.2000°W], 1660m, 20 May 1991 (R. S. Anderson); Ranchito El Quetzal, 15.21508°N, 90.22003°W, 1700m, 20 Sep 2008 (R. S. Anderson); Ranchito El Quetzal, 15.21308°N, 90.22245°W, 1870m, 20 Sep 2008 (R. S. Anderson); Salamá, Hotel Posada del Quetzal 1, 15.19710°N, 90.21169°W, 1600m, 26–30 Mar 2008 (Méndez et al.); ***Suchitepéquez***: 4km S. Vol. Atitlán, 14.55112°N, 91.19848°W, 1750m, 15 Jun 2009 (LLAMA); ***Zacapa***: 2km SE La Unión, 14.95396°N, 89.27645°W, 1430m, 12 May 2009 (LLAMA); 2km SE La Unión, 14.94654°N, 89.27600°W, 1550m, 12 May 2009 (LLAMA); **HONDURAS: *Cortés***: PN Cusuco, 15.48839°N, 88.23592°W, 1260m, 31 May 2010 (LLAMA); PN Cusuco, 15.48940°N, 88.23746°W, 1290m, 20 May 2010 (LLAMA); PN Cusuco, 15.48965°N, 88.23383°W, 1300m, 31 May 2010 (LLAMA); **MÉXICO:** ***Chiapas***: Cerro de Tapalapa, 17.18786°N, 93.12308°W, 2260m, 27 May 2008 (R. S. Anderson); 4.5km NE Coapilla, 17.1653°N, 93.1389°W, 1800m, 12 Jul 2007 (R. S. Anderson); 5km NE Coapilla, 17.17598°N, 93.13269°W, 1990m, 25 May 2008 (LLAMA); 5km NNW Coapilla, 17.18273°N, 93.15184°W, 1915m, 25 May 2008 (LLAMA); 10km W El Bosque, [ca. 17.0440°N, 92.8612°W], 1475m, 15 Sep 1992 (R. S. Anderson); 10.6km W El Bosque, [ca. 17.043°N, 92.762°W], 1460m, 25–29 Aug 1973 (A. F. Newton); L. Pojoj, Lagos de Montebello, 16.10°N, 91.67°W, 1500m, 21 Dec 1991 (P. S. Ward); Lagos de Montebello, Cinco Lagos, 16.1012°N, 91.6740°W, 1600m, 21 Jul 2007 (R. S. Anderson); Lagunas de Montebello, Cinco Lagos, [ca. 16.1167°N, 91.6833°W], 2200m, 21 Sep 1991 (R. S. Anderson); 6km SW Ocosingo, [ca. 16.8672°N, 92.0787°W], 1400m, 22 Sep 1991 (R. S. Anderson); 2.1km NW Puebla Nuevo Solistahuacan, Yerbabuena Preserve, [ca. 17.183°N, 92.900°W], 2070m, 23 Sep 1992 (R. S. Anderson); 13km N Pueblo Nuevo Solistahuacán, [ca. 17.211°N, 92.964°W], 1860m, 26–27 Aug 1973 (A. F. Newton); 5km E Rayón, 17.217°N, 92.967°W, 1700m, 23 Dec 1991 (P. S. Ward); 8.9km E Rayon, 17.200000°N, 92.91633°W, 1500m, 19 Sep 1991 (R. S. Anderson); 16km WSW S. Cristóbal, 16.69496°N, 92.77234°W, 1763m, 7 Jul 2008 (R. S. Anderson).

### Stenamma sandinista sp. n.

urn:lsid:zoobank.org:act:EE5FF4F1-5693-4D13-9FB6-4B1C2C03B5A5

http://species-id.net/wiki/Stenamma_sandinista

**Figure 150.**
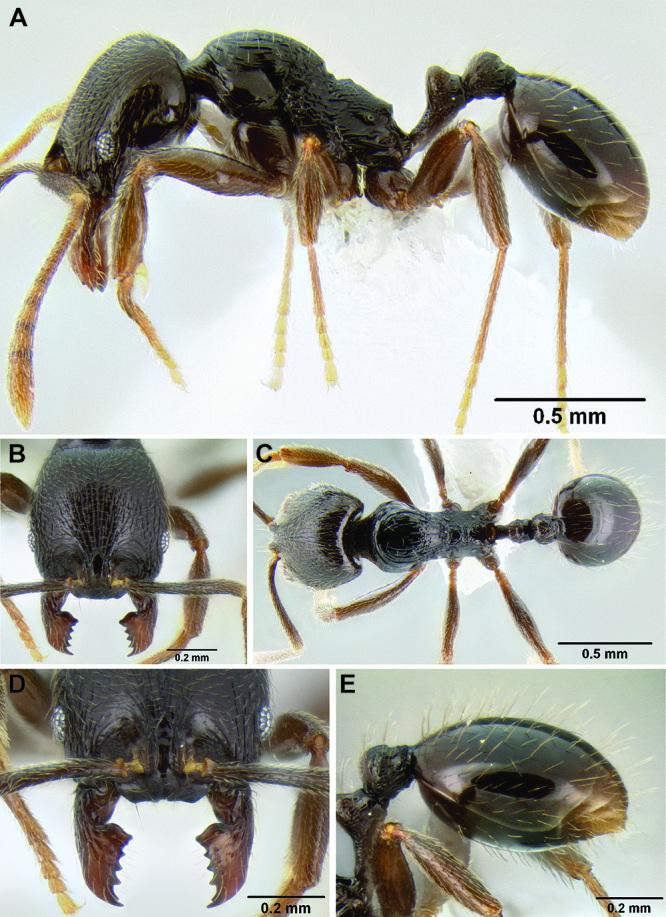
*Stenamma sandinista* holotype worker (CASENT0622578) **A** Profile **B** Face **C** Dorsum **D **Anterior clypeal margin in anterodorsal view **E** Gaster.

**Figure 151.**
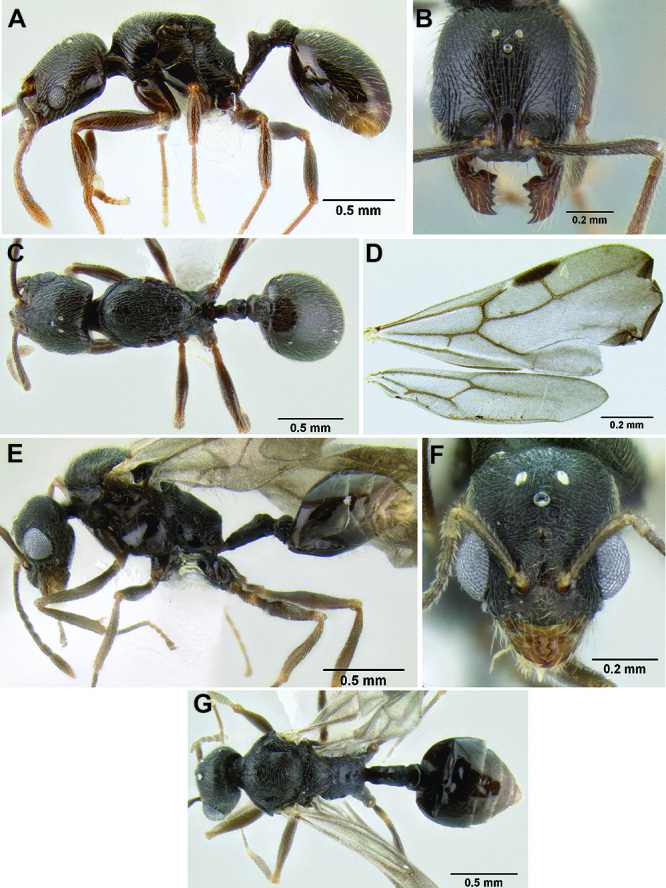
*Stenamma sandinista* **A** Paratype queen (CASENT0622583), profile **B** Same, face **C** Same, dorsum **D** Male (CASENT0622731), wings **E** Same, profile **F** Same, face **G** Same, dorsum.

Worker: Figure 150 ; Queen: Figure 151A–D ; Male: Figure 151E–G ; Map: Figure 149

#### Type material.

*Holotype worker*. NICARAGUA,Jinotega: Reserva Natural Datanlí El Diablo, 13.10852°N, 85.86780°W ±10m, 1440m, 18 May 2011, cloud forest, ex sifted leaf litter (LLAMA, collection Wa-D-04-1-01) [USNM, specimen CASENT0622578]. *Paratypes*: same data as holotype [1dq, 1w, CAS, CASENT0622575, CASENT0622576], [1w, EAPZ, CASENT0622577]; same data but 13.10859°N, 85.86724°W ±10m, 1440m, 18 May 2011 (LLAMA, Wa-D-04-1-03) [1w, ECOSCE, CASENT0623486], [1w, FMNH, CASENT0623487], [1w, ICN, CASENT0623488], [1w, INBio, CASENT0623489], [1w, JTLC, CASENT0623490], [1w, LACM, CASENT0623491], [1dq, 1w, MGBPC, CASENT0623498, CASENT0623492], [1dq, 1w, MCZ, CASENT0623493, CASENT0623494], [1w, MZSP, CASENT0623495], [1w, UCD, CASENT0623496], [1w, UNAM, CASENT0623497], [1dq, 1w, USNM, CASENT0622583, CASENT0623499], [1w, UVGC, CASENT0623500].

#### Worker diagnosis.

Integument mostly dark brown to brown; small-sized species (see HL, ML, PrW below); basal margin of mandible sinuous, with a distinct basal notch and accompanying small inner tooth; anterior clypeal margin forming 4 small teeth (inner teeth only visible from an anterodorsal angle and with mandibles open); gastral setae noticeably bilayered, with a layer of long suberect setae, and a sparse layer of short decumbent setae; face mostly covered with fine longitudinal carinulae and rugulae, posterior margin and posterolateral surfaces smooth and shiny; promesonotal dorsum with fine longitudinal and arcuate carinulae, that usually wrap around a central patch of smooth cuticle, remainder of mesosoma with light rugulae and punctae; eye of moderate size (EL 0.10–0.14, REL 15–20), with 4–5 ommatidia at greatest diameter; propodeal spines reduced to very small tubercles (PSL 0.07–0.09, PSI 1.1–1.3); frontal lobes of moderate width (FLD 0.13–0.16, FLI 20–24), not obscuring torular lobes in full-face view. *Similar species*: *Stenamma nanozoi*, *Stenamma nonotch*, *Stenamma saenzae*, *Stenamma schmidti*.

#### Geographic range.

Nicaragua.

#### Worker description.

(10 measured) HL 0.62–0.73 (0.62), HW 0.56–0.70 (0.56), FLD 0.13–0.16 (0.13), PCW 0.03–0.04 (0.03), SL 0.52–0.61 (0.52), EL 0.10–0.14 (0.11), ACL 0.51–0.58 (0.51), ML 0.77–0.89 (0.77), PrW 0.07–0.09 (0.08), PSL 0.07–0.09 (008), SDL 0.06–0.08 (0.07), PL 0.27–0.34 (0.28), PH 0.17–0.20 (0.17), PW 0.13–0.17 (0.13), PPL 0.15–0.19 (0.16), PPH 0.15–0.18 (0.15), PPW 0.17–0.21 (0.17), MFL 0.55–0.65 (0.55), MTL 0.43–0.52 (0.43), CI 90–96 (90), SI 87–94 (94), REL 15–20 (19), FLI 20–24 (24), PSI 1.1–1.3 (1.2), MFI 98–111 (102), ACI1 65–67 (67), ACI2 91–97 (96).

Small-sized species; general body color dark brown to brown, with appendages lighter, brown to yellow-brown toward extremities; setae golden brown; mandible usually with 6 distinct teeth, basal tooth well-defined; basal margin of mandible sinuous, with a distinct basal notch and accompanying small inner tooth; mandible mostly smooth, except for scattered piligerous punctae, and several striae around the base and lateral surface; anterior clypeal margin in anterodorsal view forming 4 small teeth (only outer teeth visible in full-face view); median clypeal lobe with faint longitudinal carinulae that diverge toward anterior margin, apex of lobe with a short and long transverse carinula, area in between transverse carinula and anterior clypeal margin forming a distinct cavity for mandibles, remainder of clypeal surface mostly smooth; posterior extension of clypeus between antennal insertions of moderate width (PCW 0.03–0.04), with sides subparallel; frontal lobes of moderate width (FLD 0.13–0.16, FLI 20–24), not covering torular lobes in full-face view; head subcircular to oval-shaped (CI 90–96), with posterior margin distinctly depressed; eye of moderate size (EL 0.10–0.14, REL 15–20), with 4–5 ommatidia at greatest diameter; face mostly covered with many short to long longitudinal carinulae and rugulae, posterior margin and posterolateral surfaces smooth and shiny; scape of moderate length (SI 87–94), not quite reaching posterior margin when laid back; scape surface mostly shiny, with some faint striations, and scattered piligerous punctae; flagellum with a distinct 4-segmented antennal club; promesonotal dorsum with longitudinal and arcuate carinulae, that usually wrap around a central patch of smooth cuticle, but sometimes entire dorsum sparsely carinulate, side of pronotum mostly smooth, mesopleuron and side of propodeum with variably developed punctae and rugulae, sometimes mesopleuron mostly smooth, propodeal dorsum and declivity with some transverse carinulae; promesonotum in profile low-domed, and roughly symmetrical; metanotal groove in profile of moderate width and depth, often with a small central welt (metanotum); propodeal spines reduced to small tubercles (PSL 0.07–0.09, PSI 1.1–1.3); dorsal edge of propodeal lobe in profile markedly elongate; petiole in profile appearing of moderate to long length (PL/HW 0.45–0.51); petiolar node of moderate height (PH/PL 0.58–0.65), slightly asymmetrical, with anterior face longer and more sloping than posterior face, dorsum of node gently rounded, always pointing distinctly posteriad; postpetiole in profile appearing similar in size to petiolar node (PPH/PH 0.83–0.94), subspherical, with anterior face only slightly longer than posterior face; petiole and postpetiole mostly lightly punctate, with some rugulae around nodes, anterior faces of nodes and dorsum of peduncle mostly smooth; gaster mostly smooth and shiny, with scattered piligerous punctae; face pilosity short, with a layer of suberect setae, and a denser layer of decumbent setae; setae on mesosoma and gaster mostly long and suberect, gaster with a sparse layer of decumbent setae; scape setae uniformly subdecumbent; setae on legs mostly subdecumbent, with a few suberect setae on femoral venters and coxae.

#### Queen description.

(5 measured) HL 0.67–0.70 (0.69), HW 0.62–0.65 (0.63), FLD 0.15–0.16 (0.15), PCW 0.03–0.05 (0.04), SL 0.58–0.59 (0.59), EL 0.17–0.18 (0.18), ACL 0.54–0.56 (0.54), ML 0.96–0.99 (0.99), PrW 0.55–0.56, PSL 0.10–0.12, SDL 0.07–0.09, PL 0.35–0.37 (0.36), PH 0.20 PW 0.16–0.18 (0.18), PPL 0.18–0.19 (0.19), PPH 0.19–0.21 (0.21), PPW 0.21–0.22 (0.22), MFL 0.62–0.65 (0.65), MTL 0.50–0.52 (0.51), CI 92–95 (92), SI 90–93 (93), REL 27–28 (28), FLI 24–25 (24), PSI 1.2–1.4 (1.3), MFI 98–104 (98), ACI1 63–68 (65), ACI2 92–97 (92).

Same as worker except for standard queen modifications and as follows: pronotum with transverse carinulae/rugulae; mesoscutum mostly with longitudinal carinulae/rugulae, sometimes humeral area mostly smooth, with only piligerous punctae; scutellum smooth in center, longitudinal rugulae elsewhere; propodeum with transverse carinulae that wrap around surface; mesopleuron mostly smooth; pilosity on gastral tergites clearly bilayered with a dense layer of short, subdecumbent pubescence, and a relatively sparse layer of longer suberect setae (not as long as in worker); wing venation as in Figure 151D.

#### Male.

See Figure 151E–G.

#### Biology.

*Stenamma sandinista* is a specialist of montane wet forest habitats (e.g. cloud forest, dwarf forest, mesic forest) and is known from approximately 900–1600 m elevation. Nearly all collections are from sifted leaf litter taken from the forest floor. Only once in dwarf cloud forest habitat have I found a nest fragment. It was in leaf litter and was located by randomly scraping back the top layer of leaves while collecting a Winkler sample. The nest had tens of workers and brood.

#### Comments.

*Stenamma sandinista* can be separated from similar forms by its eye size, mandibular structure, facial sculpture, and gastral pilosity. Geography is also useful, as it is present at only a few high-elevation sites within Nicaragua.

Given the complexity of the *Stenamma schmidti* complex (see below), it is possible that *Stenamma sandinista* is a high-elevation, allopatric variant of *Stenamma schmidti*. However, I describe it as a new species for the following reasons: it occurs at several sites within Nicaragua and maintains a consistent phenotype; it is sympatric at Cerro Musún with a somewhat distantly related *Stenamma schmidti* variant that occurs from Costa Rica to Nicaragua; and its sister species, *Stenamma nanozoi*, is quite divergent and restricted to the lowlands of Honduras.

#### Material examined.

**NICARAGUA: *Jinotega***: RN Datanlí El Diablo, 13.09452°N, 85.85942°W, 1300m, 20 May 2011 (LLAMA); RN Datanlí El Diablo, 13.10852°N, 85.86780°W, 1440m, 18 May 2011 (LLAMA); RN Datanlí El Diablo, 13.12233°N, 85.87024°W, 1530m, 19 May 2011 (LLAMA); ***Matagalpa***: RN El Musún, 4.8km NNW Río Blanco, 12.97389°N, 85.23380°W, 900m, 11 Oct 2008 (M. G. Branstetter); RN El Musún, 5.5km NNW Río Blanco, 12.98051°N, 85.2319°W, 1390m, 11 Oct 2008 (M. G. Branstetter); Selva Negra, 9.1km NNE Matagalpa, 13.00717°N, 85.91000°W, 1500m, 7 Oct 2008 (M. G. Branstetter).

### Stenamma schmidti

Menozzi

http://species-id.net/wiki/Stenamma_schmidti

**Figure 152.**
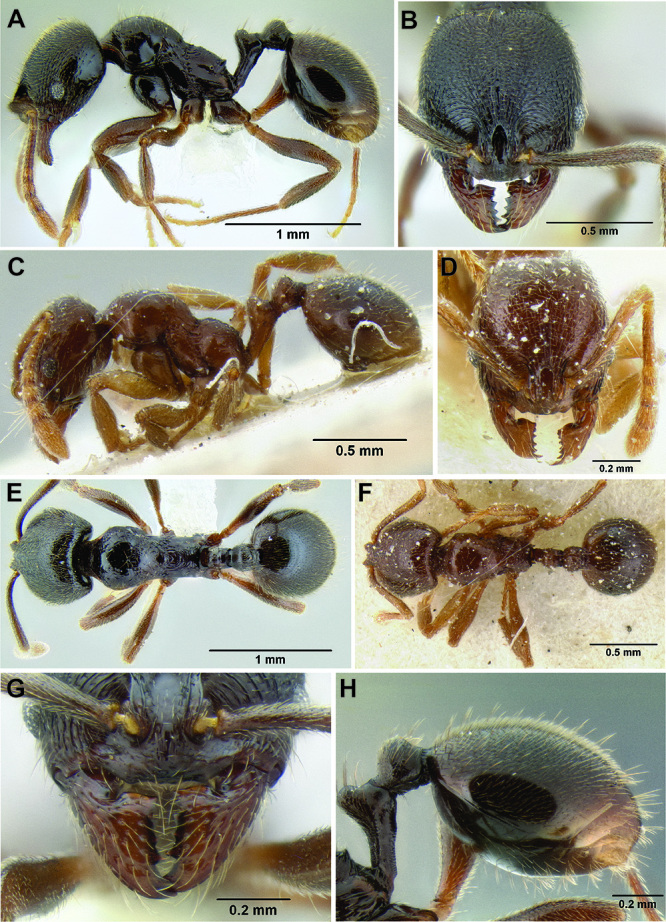
*Stenamma schmidti* **A, B, E, G, H** Worker (INB0003665417); **C, D, F** Holotype worker (CASENT0126701).

**Figure 153.**
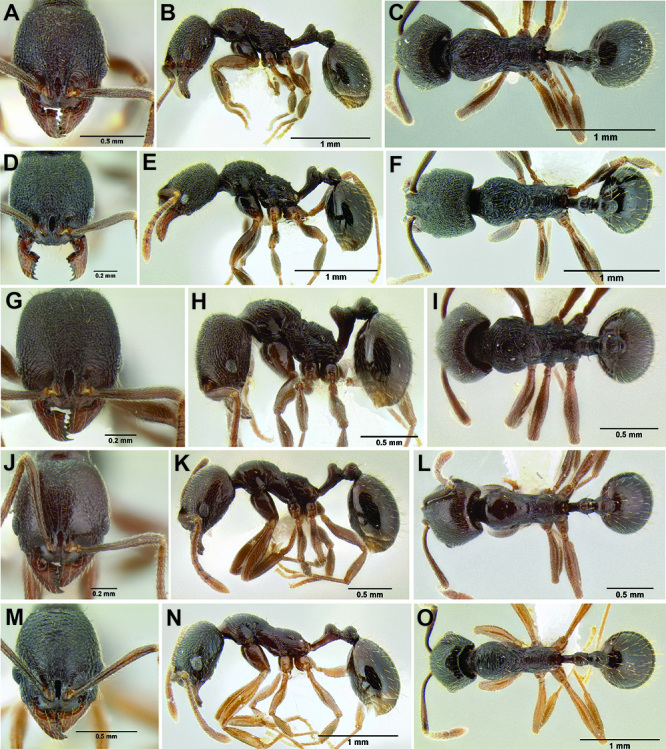
*Stenamma schmidti* worker variants 1. Face, profile, and dorsal views **A–C** Variant 1 (INB0003659427) **D–F** Variant 2 (CASENT0600256), **G–I** Variant 3 (INB0003662571) **J–L** Variant 4 (INBIOCRI001280989) **M–O** Variant 5 (INB0003210597).

**Figure 154.**
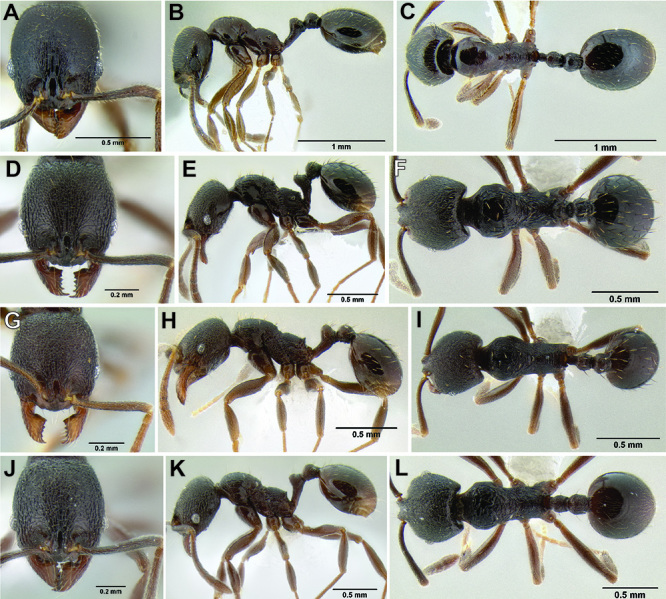
*Stenamma schmidti* worker variants 2. Face, profile, and dorsal views **A–C** Variant 6 (INB0002659320) **D–F** Variant 7 (CASENT0622465) **G–I** Variant 8 (CASENT0605211) **J–L** Variant 9 (CASENT0605896).

**Figure 155.**
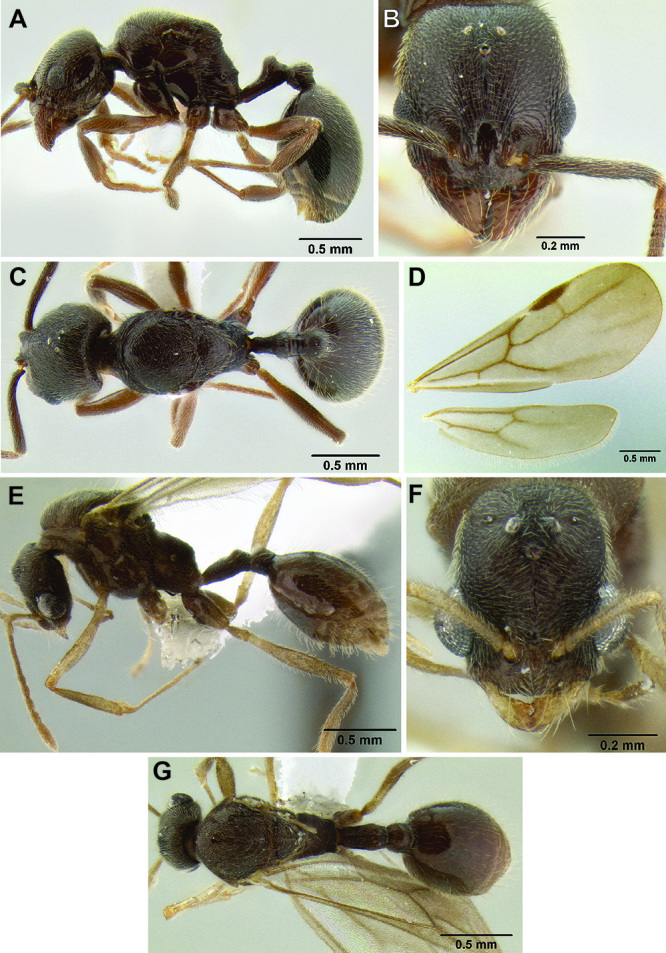
*Stenamma schmidti* **A** Queen (INBIOCRI002281278), profile **B** Same, face **C** Same, dorsum **D** Male (INBIOCRI002281270), wings **E** Same, profile **F** Same, face **G** Same, dorsum.

**Figure 156.**
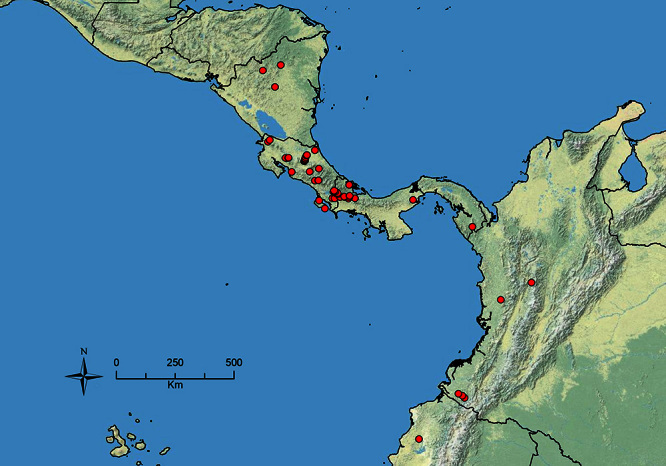
Distribution map of *Stenamma schmidti*.

Worker: Figures 152- 154 ; Queen: Figure 155A–D ; Male: Figure 155E–G ; Map: Figure 156

Stenamma schmidti Menozzi, 1931a: 198, fig 6. Holotypeworker: COSTA RICA, [Heredia]: Vara Blanca, [ca. 10.167°N, 84.150°W], 2000m (H. Schmidti) (NHMB, specimen CASENT0126701) [examined]. Menozzi, 1931b: 267, distribution, remarks on type locality. Borgmeier, 1937: 232: distribution, remarks on taxonomy of worker. Branstetter, 2012: phylogeny.

#### Worker diagnosis.

Note that this species is highly variable. See comments section below discussing morphological variants. Integument mostly dark brown, red-brown, or brown; small- to medium-sized species; petiolar node in profile usually broadly rounded and distinctly angled posteriad; postpetiole in profile subspherical; propodeal spines absent to tuberculate (PSL 0.06–0.13, PSI 0.8–1.5); basal margin of mandible usually with a distinct basal notch and small accompanying tooth, but sometimes with only a small notch, or with basal margin sinuous. If basal margin of mandible with notch and tooth then: anterior clypeal margin forming 2–4 sharp to blunt teeth, with outer teeth more projecting; eye of moderate to large size (EL 0.10–0.18, REL 19–29), with 6–10 ommatidia at greatest diameter. If basal margin with small notch, but no tooth then: face completely sculptured, densely rugoreticulate; mesosoma mostly sculptured, punctate-rugulose; pilosity on first gastral tergite sparse, mostly stout and suberect, with only a few decumbent setae. If basal margin of mandible sinuous (without notch and tooth) then: anterior clypeal margin with a simple median emargination; propodeal spines absent, reduced to blunt angles where propodeal dorsum and declivity meet; eye large (EL 0.15–0.18, REL 22–27), with 8–11 ommatidia at greatest diameter; face usually completely sculptured, but sculpture never very dense, mostly carinulate-punctate; carinulae usually longitudinal, but some specimens with transverse carinulae on anterior half of head; pronotum either completely carinulate-punctate, lightly punctate, or completely smooth; carinulae when present usually transverse in orientation; some specimens noticeably long and gracile, with scape, metafemur, and petiole relatively long (SI 107–121, 86–93; PL/HW 0.58–0.63); gastral pilosity mostly sparse and suberect, with only a few decumbent to appressed setae; erect setae often stout.

#### Geographic range.

Nicaragua to Ecuador.

#### Worker description.

(63 measured) HL 0.55–0.93 (0.66), HW 0.48–0.80 (0.57), FLD 0.11–0.25 (0.16), PCW 0.02–0.06 (0.04), SL 0.46–0.82 (0.55), EL 0.09–0.18 (0.12), ACL 0.47–0.75 (0.55), ML 0.68–1.21 (0.84), PrW 0.34–0.56 (0.40), PSL 0.06–0.13 (0.09), SDL 0.06–0.11 (0.09). PL 0.25–0.45 (0.31), PH 0.16–0.28 (0.19), PW 0.13–0.21 (0.15), PPL 0.13–0.27 (0.17), PPH 0.14–0.26 (0.18), PPW 0.16–0.28 (0.20), MFL 0.50–0.98 (0.59), MTL 0.41–0.76 (0.45), CI 79–94 (86), SI 85–121 (97), REL 18–29 (21), FLI 21–33 (29), PSI 0.8–1.5 (1.1), MFI 71–108 (96), ACI1 62–68 (66), ACI2 86–105 (100).

Small- to medium-sized species; general body color dark brown, to red-brown, to brown, with appendages brown to orange-brown, lighter at joints and toward extremities; setae golden; mandible with 5–7 teeth (usually 6), with basal tooth often appearing bidentate, inner teeth sometimes worn and indistinct; basal margin of mandible usually sinuous, with a distinct basal notch and accompanying small tooth (type population), but sometimes with a basal notch and no tooth, or only sinuous; mandible mostly smooth and shiny, with scattered piligerous punctae and a variable number of basal and lateral striae; anterior clypeal margin usually forming 2–4 sharp to blunt teeth (type population), but sometimes nearly flat, or with a simple median emargination; median clypeal lobe usually rather distinct, and produced slightly over anterior clypeal margin in full-face view (type population), but sometimes obliquely flattened and not produced; dorsal surface of lobe mostly smooth and shiny (type population), or with a variable number or irregular carinulae, apex of lobe with a short to long transverse carina; area in between carina and anterior clypeal margin, usually forming a distinct cavity where mandibles insert (type population); remainder of clypeal surface mostly smooth; posterior extension of clypeus between antennal insertions of narrow to moderate width (PCW 0.02–0.06; type population), with sides subparallel; frontal lobes moderate (type population) to slightly expanded (FLD 0.11–0.25; FLI 21–33), not greatly covering torular lobes in full-face view; head usually roughly oval-shaped (type population) to subcircular, with a distinct median depression in posterior margin, but head sometimes more elongate, with posterior margin flat (CI 79–94); eye of moderate to large size, sometimes relatively very large (EL 0.09–0.18, REL 18–29), oval-shaped, often bulging, with 5–11 ommatidia at greatest diameter; face sculpture highly variable, ranging from mostly smooth and shiny, to densely rugoreticulate (type population with fine longitudinal carinulae on middle of head and on gena, posterior 1/5 of head smooth); scape ranging from relatively short and somewhat thick to long and slender (SI 85–121), not surpassing to distinctly passing posterior margin when laid back (moderate length in type population, just reaching posterior margin); scape surface with numerous piligerous punctae, but mostly shiny; flagellum with a distinct (type population) to very distinct 4-segmented antennal club; mesosomal sculpture highly variable, ranging from mostly smooth, to densely rugose-rugoreticulate (type population with promesonotum mostly smooth and shiny, at most a few longitudinal carinulae dorsally); mesopleuron and side of propodeum mostly smooth, with some faint punctae and carinulae, propodeal dorsum and declivity with light transverse carinulae; promesonotum in profile usually low-domed and roughly symmetrical; metanotal groove variable, usually well-demarcated and of moderate width and depth (type population), but sometimes deeper and better defined, with metanotum forming a small welt, or sometimes shallow and indistinct, with propodeum connecting almost continuously to promesonotum; propodeal spines absent to tuberculate (PSL 0.06–0.13, PSI 0.8–1.5; forming a sharp angle in type population); petiole in profile appearing of moderate length (type population) to somewhat elongate (PL/ML 0.48–0.63); petiolar node usually broadly rounded and pointing distinctly posteriad (type population), but sometimes appearing subquadrate and asymmetrical, with an apex occurring at anterior margin of dorsum; petiolar node similar in size to petiolar node and subspherical; petiole and postpetiole usually mostly smooth and shiny, with only a few faint punctae (type population), but sometimes mostly punctate, rugulae sometimes present on posterior half of postpetiolar node; gaster mostly smooth, with scattered piligerous punctae; most of body dorsum with standing pilosity; gastral pilosity highly variable, sometimes distinctly bilayered, with a sparse layer of stout suberect setae, and a dense layer of short, decumbent pubescence (type population), sometimes pubescence absent, leaving only stout, suberect setae and a few decumbent setae, or sometimes all setae of moderate thickness and with variable density and layering (suberect layer usually always present); setae on scape usually relatively dense, and uniformly subdecumbent to appressed; setae on legs decumbent to appressed, with some longer suberect setae on femoral venters and coxae.

#### Queen description.

(16 measured) HL 0.59–0.89 (0.81), HW 0.53–0.76 (0.74), FLD 0.13–0.25 (0.22), PCW 0.03–0.07 (0.06), SL 0.48–0.81 (0.65), EL 0.16–0.25 (0.21), ACL 0.49–0.73 (0.65), ML 0.79–1.30 (1.17), PrW 0.45–0.68 (0.60), PSL 0.08–0.15 (0.12), SDL 0.08–0.13 (0.12), PL 0.30–0.47 (0.44), PH 0.18–0.26 (0.24), PW 0.16–0.23 (0.20), PPL 0.15–0.26 (0.24), PPH 0.16–0.24 (0.23), PPW 0.19–0.28 (0.26), MFL 0.53–0.93 (0.77), MTL 0.43–0.71 (0.60), CI 83–94 (91), SI 84–111 (88), REL 26–34 (29), FLI 24–34 (30), PSI 1.0–1.4 (1.0), MFI 78–103 (96), ACI1 63–67 (63), ACI2 90–106 (99).

Same as worker except for standard queen modifications and as follows (only type population queen considered): pronotum with some transverse rugulae laterad; mesoscutum lightly punctate to foveolate, with a central line of smooth cuticle; scutellum with longitudinal carinulae laterad, and a central smooth patch; propodeum with transverse carinulae that wrap around entire surface; mesopleuron mostly smooth; pilosity on mesoscutum bilayered similar to gaster, with a layer of longer erect to suberect setae, and a layer of short, dense pubescence; wing venation as in Figure 155D.

#### Male.

See Figure 155E–G.

#### Biology.

*Stenamma schmidti*, as defined here, is a rather variable species. It inhabits tropical wet forest environments from sea level to about 2400 m, becoming most abundant in cloud forest habitats above 800 m. At some cloud forest sites, *Stenamma schmidti* can be one of the most common ant species occupying the leaf litter. Despite this fact, the species is very cryptic and finding nests is an uncommon event. Most collections of *Stenamma schmidti* are from Winkler or Berlese samples of sifted leaf litter or epiphyte mats. Nest collections have been made, but these are very rare for the leaf-litter dwelling variants and only slightly more common for the arboreal forms. Nests are very small, with only tens of workers, and a single egg-laying queen. Workers, when encountered, are very slow moving and freeze upon disturbance. Additional natural history notes specific to particular morphological variants are described below.

#### Comments.

The *Stenamma schmidti* complex is as remarkable as it is maddening, comprising an amazing radiation of forms, which together occupy more morphospace than all of the Holarctic clade *Stenamma* species combined. Because many putative morphospecies appear to occur in sympatry, it is tempting to separate *Stenamma schmidti* into multiple species. But after careful review of many specimens and populations, accompanied by molecular phylogenetic data, I find it very difficult to divide up the complex in a satisfactory way. A major reason for this is the existence of specimens with intermediate phenotypes, forming what appears to be a morphological continuum. Consequently, I have decided to delimit a single polytypic species composed of many variants (described below). Several of these variants may constitute good species, but I believe more genetic and distribution data are needed (especially from Panama and South America) to adequately assess the distinctness of all forms in the complex.

The difficulty in resolving species boundaries in the complex probably stems from several factors, including phenotypic plasticity, morphological convergence, hybridization, and sampling bias. It could also be that if the group represents a recent radiation, there has been insufficient time for some species to reach monophyly. Based on my observations so far, I favor the idea that there are several weakly differentiated forms that occasionally come into contact and hybridize.

An interesting observation about diversification within the *schmidti* complex is that it seems particularly prone to convergence, with specimens from a particular elevation and microhabitat having similar morphological characteristics. For example, *Stenamma nanozoi* and variants 7 and 8 of *Stenamma schmidti* are all small, have densely sculptured faces, and have the gastral pilosity forming a single sparse layer of stout, suberect setae. All of these taxa occur at low elevation from sea level to approximately 1000 m. Counter to expectations, phylogenetic data show that they are not closely related to one another, with *Stenamma nanozoi* sister to *Stenamma sandinista*, and variants 6 and 7 of *Stenamma schmidti* both more closely related to other high-elevation variants (Branstetter unpublished data). This suggests that the characteristics one would probably use to unite these taxa as a single species are convergent, and thus misleading.

By combining many divergent forms into a single species, it has made characterizing *Stenamma schmidti* rather difficult due to its large phenotypic range. Most specimens that closely match one of the variants below should be identifiable as *Stenamma schmidti*, but specimens with intermediate phenotypes will probably not key out easily.

It will also be hard to adequately separate *Stenamma schmidti* from the closely related *Stenamma nanozoi* and *Stenamma sandinista*, as well as from the similar looking *Stenamma saenzae*, based only on morphology. The characters in the key and diagnosis will work for most specimens, but not all. Fortunately, geography should clarify matters, as most of the *schmidti* complex occurs farther south than the other species. Only *Stenamma sandinista* is found in sympatry with variant 6 of *Stenamma schmidti*. These two species both occur at Cerro Musún in Nicaragua, where they are separated by elevation, with *Stenamma schmidti* occurring below about 800 m and *Stenamma sandinista* occurring above 900 m.

Defining features of the holotype’s morphology (Figure 152C, D, F) are indicated in the species description above (see parenthetical comments), but main characteristics are as follows: basal margin of mandible with distinct notch and small accompanying tooth; anterior clypeal margin forming 2–4 teeth; median lobe of clypeus produced slightly over anterior clypeal margin in full-face view; head roughly oval-shaped to subcircular (CI 85–93); scape of moderate length (SI 85–97); eye large (EL 0.12–0.16, REL 19–23), with 6–9 ommatidia at greatest diameter; face sculpture mostly lightly carinulate, with posterior half or less of head usually smooth; mesosoma mostly smooth, with some faint carinulae/rugulae variably present; gastral pilosity distinctly bilayered, with a sparse layer of suberect setae, and a dense layer of decumbent pubescence. The holotype appears to be a small, less robust specimen of *Stenamma schmidti*, with much of the face and mesosoma smooth. A paratype specimen I observed from the same series as the holotype is slightly larger and has more developed sculpture, with the face carinulae extending close to the posterior margin of the head. What I consider to be the holotype form of *Stenamma schmidti* is a high-elevation morphospecies occurring in leaf litter from 1500 to over 2000 m. I have observed similar specimens from multiple sites in Costa Rica and in Panama. Specimens from the Cordillera de Talamanca in southern Costa Rica (e.g. Las Alturas, Altamira, Pittier) and from northern Panama (e.g. Cerro Punta, Hartman Finca) usually have more sculpture on the face and mesosoma. Molecular phylogenetic data show some clustering of specimens from Central Costa Rica, but specimens from southern Costa Rica and Panama are scattered throughout the tree. Nests of this form are almost unknown. Longino (pers. comm.) has reported finding a nest in the root disc of a fallen tree.

Variant 1 (Figure 153A–C) is a densely sculptured version of the holotype form. It varies as follows: face completely sculptured, with dense costulae in middle changing to rugoreticulae toward sides; pronotum with dense longitudinal costulae/rugulae, sometimes with a patch of smooth cuticle on side and middle of dorsum; mesonotum rugoreticulate; katepisternum mostly smooth; side of propodeum rugose; dorsum and declivity of propodeum transversely carinulate; petiole and postpetiole mostly punctate. Variant 1 occurs at multiple sites in Costa Rica and northern Panama and seems to occur only at mid-elevations from 800–1500 m. It is known only from samples of sifted leaf litter.

Variant 2 (Figure 153D–F) is a more robust version of variant 1. It differs as follows: body color darker; sculpture deeper, more developed; petiolar node thicker, more robust; propodeal lobes and spines in profile view noticeably broader; frontal lobes somewhat expanded, almost completely covering torular lobes in full-face view; layer of pubescent setae on gaster absent, with only a few short decumbent setae present under stout, suberect setae. This variant is known from sites in southern Costa Rica and northern Panama and occurs at mid-elevations between 1000–1500 m. It is known only from samples of sifted leaf litter.

Variant 3 (Figure 153G–I) is similar to variant 2, except as follows: sculpture on pronotal dorsum distinctly transverse and arcuate; sculpture on side of propodeum more dense and oriented so that it wraps up and over the dorsal surface; promesonotum in profile view slightly more robust, and more distinctly separated from metanotal groove. Variant 3 occurs at multiple sites in Costa Rica at mid-elevations between 1000–1500 m. Results from molecular phylogenetic data show that specimens of variant 2 and 3 from several sites in Costa Rica form a clade nested inside the *schmidti* complex. This might be evidence that these two variants represent a single good species. However, it is strange that the very similar looking variant 1 does not cluster with variant 2 and 3. A broader sampling of specimens from Costa Rica and especially Panama is needed to further investigate species boundaries. This variant is known only from samples of sifted leaf litter.

Variant 4 (Figure 153J–L) is similar to the holotype form, except as follows: face and mesosomal sculpture usually reduced, with posterior half of face mostly smooth; notch in basal margin of mandible very deep; anterior clypeal margin forming 4 distinct teeth, with outer teeth strongly projecting (presumably to fit in notch in basal margin of mandible); gastral pilosity mainly forming a single sparse layer of stout, suberect setae. This variant seems to be an arboreal version of the holotype form of *Stenamma schmidti*, with most specimens coming from canopy fogging samples or Winkler samples of epiphytic material. Some specimens are also known from litter samples taken from the forest floor. Variant 4 occurs mainly at mid elevations at Monteverde and Volcán Barva in Costa Rica. Specimens from the canopy at Monteverde are rather uniform in morphology, but specimens from the forest floor at Monteverde and on Volcán Barva are more variable and become difficult to assign to a particular variant with certainty. These difficult specimens have intermediate sculpture and gastral pilosity.

Variant 5 (Figure 153M–O) differs drastically from the holotype form and is potentially a good species nested within the *schmidti* complex. It has the following characters: basal margin of mandible sinuous, without a basal notch or tooth; anterior clypeal margin with a simple median emargination; head more elongate and narrow (CI 79–84), with posterior margin flat; eye large (EL 0.15–0.18, REL 22–27); scape long, surpassing posterior margin of head when laid back (SI 107–121); propodeal spines absent, at most forming blunt angles; mesosoma noticeably elongate and gracile; petiole noticeably elongate; sculpture on head and mesosoma carinulate-punctate; face sometimes with transverse carinulae on anterior half; pronotum with transverse carinulae; legs elongate (MFI 71–80); gastral pilosity forming a single sparse layer of stout, suberect setae. This variant is known only from Monteverde and Volcán Barva in Costa Rica from 1000–1500 m. It is apparently an arboreal form of *Stenamma schmidti* with specimens collected from fogging samples and from nests found under epiphytes in the canopy. Longino (pers. comm.) has reported that this variant prefers to nest under small epiphyte clumps on small branches. Molecular phylogenetic results show specimens from Monteverde and Volcán Barva clustering together, suggesting some reproductive isolation. However, I find specimens with intermediate morphology between this variant, the holotype form, and variant 4. Some specimens have the anterior clypeal margin forming sharp teeth, or the mesosoma stockier and less gracile. Counter to expectations, the intermediates do not cluster with variant 5 specimens in the phylogeny.

Variant 6 (Figure 154A–C) is similar to variant 5 in that it has the same mandible and clypeus structure, it lacks propodeal spines, and it has a relatively large eye. However, it has the following differences: smaller overall size; mesosoma less elongate and gracile; head roughly oval-shaped, less elongate (CI 85–87); scape and metafemur relatively smaller (SI 94–101, MFI 86–93); gastral pilosity not stout; sculpture reduced, usually faintly punctate or smooth. Variant 6 like variant 5 is known only from Monteverde and Volcán Barva in Costa Rica, and it is arboreal, with specimens collected from fogging samples and nests in epiphyte clumps. Molecular phylogenetic data show specimens from Monteverde and Volcán Barva clustering together. But, despite the similarities with variant 5, the two variants do not cluster together. I would not be surprised if variant 6 is a hybrid between variant 5 and the holotype form of *Stenamma schmidti* or variant 7. It may also be a distinct species that has convergently evolved some features of variant 5.

Variant 7 (Figure 154D–F) is a smaller, low-elevation version of the holotype form. It has the following differences: smaller overall size; face completely sculptured, with carinulae in middle changing to rugoreticulae toward lateral margin; dorsum of promesonotum usually with carinulae encircling margins (humeri), remainder of promesonotum smooth; eye sometimes relatively very large (EL 0.13–0.16, REL 23–29); gastral pilosity mainly a single sparse layer of stout, suberect setae, with a few decumbent setae underneath. This variant occurs from sea level to about 1000 m elevation and has been collected at multiple sites in Nicaragua and Costa Rica. It is very similar to variant 8, *Stenamma nanozoi*, and specimens from lowland Panama, Colombia, and Ecuador, but molecular phylogenetic data do not cluster all of these similar-looking forms together. Only specimens from lowland Nicaragua and northern Costa Rica form a clade. This variant is distinct and easy to identify at low elevations, but at mid elevations in Costa Rica, especially on Volcán Barva, it becomes very difficult to separate this variant from the holotype form or several of the other variants. Some of this difficulty might be because smaller specimens of the other variants look like variant 7, but I suspect it is also because of hybridization and perhaps adaptation of variant 7 to the higher elevation environment. Nearly all specimens of this variant are known from leaf litter, but one nest was found at the La Selva Biological Station in Costa Rica. It was in a small branch in the leaf litter (Longino, pers. comm.).

Variant 8 (Figure 154G–I) is a small version of *Stenamma schmidti* and is superficially similar to variant 7, but differs as follows: face mostly rugoreticulae; mesosoma more sculptured, mostly punctate to rugulose-punctate; eye usually smaller (EL 0.09–0.11, REL 19–20), with 5–6 ommatidia at greatest diameter; basal margin of mandible sometimes with only a small basal notch and no tooth; anterior clypeal margin sometimes appearing flat, without defined teeth. This variant mainly includes specimens from the Osa Peninsula in Costa Rica (13 km SSW Pt. Jimenez), but many lowland specimens from Panama to Ecuador are similar, with some variation in sculpture and mandible and clypeus structure. Despite similarities with variant 7, molecular phylogenetic data do not show these two variants clustering together, suggesting that their similar morphology is convergent. I find some similarities in mesosoma shape and sculpture between variant 8 and high-elevation specimens from southern Costa Rica that I assign to the holotype form of *Stenamma schmidti*. Perhaps overall size and pilosity are misleading characters in the *schmidti* complex.

Variant 9 (Figure 154J–L) is a mid- to high-elevation version of variant 8. It differs as follows: larger overall size (similar to holotype form); eye larger (EL 0.13–0.14, REL 20–21); dorsum of promesonotum with transverse arcuate carinulae; pilosity on gastral dorsum distinctly bilayered, with a sparse layer of somewhat stout suberect setae, and a layer of dense decumbent to appressed pubescence (compared to type form, suberect setae longer and less stout, and pubescence less dense). Variant 9 occurs between 1300–2000 m elevation and is known from a few sites in Colombia. I have not been able to include this variant in the phylogeny, but I suspect it is closely related to variant 8 and the lowland specimens from Panama and Ecuador.

#### Material examined.

**COLOMBIA: *Caldas***: Aguadas, Los Naranjos, Pte Albania, [ca. 5.60°N, 75.45°W], 2220m, 16 Nov 1994 (C. Sarmiento); ***Chocó***: Mpio. Novita, Vereda, Curundó, Río Ingará, [ca. 4.950°N, 76.617°W], 500m, 12 Jan 1983 (T. van der Hammen et al.);***Nariño***: El Diviso, [ca. 1.367°N, 78.233°W], 520m, Jul 1994 (F. Escobar); Ricuarte, R.N. La Planada, 1.206°N, 77.994°W, 1800m, Apr 1994 (F. Escobar); Río Nambí, [ca. 1.292°N, 78.084°W], 1350m, May 1995 (F. Escobar); **COSTA RICA: *Alajuela***: 10km E Monteverde, 10.30976°N, 84.71993°W, 880m, 1 Mar 2010 (J. Longino); Río Peñas Blancas, 10.3167°N, 84.7167°W, 800m, 26 Apr 1987 (J. Longino);***Cartago***: Ref. Nac. Fauna Silv. Tapanti, [ca. 9.792°N, 83.910°W], Nov 1991 (G. Mora); 4km E Turrialba, 9.90°N, 83.56°W, 550m, 13 May 1987 (J. Longino); ***Guanacaste***: Est. Cacao, Lado SO Vol. Cacao, [ca. 10.9167°N, 85.5000°W], 1200m, Jul 1991 (C. Chaves); Estacion Pitilla, 8km S Santa Cecilia, 10.9833°N, 85.4333°W, 650m, 24 Jan 1991 (J. Longino);***Heredia***: 11km SE La Virgen, 10.3333°N, 84.0667°W, 500m, 16 Apr 2003 (ALAS); 16km SSE La Virgen, 10.2667°N, 84.0833°W, 1100m, 14–17 Mar 2001 (ALAS); La Selva Biological Station, 10.4197°N, 84.0136°W, 2 Jul 1992 (J. Longino); PN Braulio Carrillo, 10.4043°N, 84.0385°W, 4 Mar 2005 (TEAM); 6km ENE Vara Blanca, 10.1833°N, 84.1167°W, 1900m, 16 Apr 2002 (ALAS); 9km NE Vara Blanca, 10.2333°N, 84.0833°W, 1500m, 14–20 Feb 2005 (R. S. Anderson); 10km NE Vara Blanca, 10.2333°N, 84.0833°W, 1500m, 12 Apr 2005 (ALAS); 6km N Vol. Barba, 10.1833°N, 84.1167°W, 1950m, 4 Jul 1986 (J. Longino); 8km N Vol. Barba, 10.20°N, 84.10°W, 1830m, 7 Jul 1986 (J. Longino); 9km N Vol. Barba, 10.2167°N, 84.1000°W, 1750m, 5 Jul 1986 (J. Longino); 17km N Vol. Barba, 10.2833°N, 84.0833°W, 800m, 14 Jul 1986 (J. Longino); ***Limón***: Sector Cerro Cocorí, Finca de E Rojas, 10.600°N, 83.717°W, 150m, Jun 1991 (E. Rojas); Valle del Silencio, Cerro Quemado, 9.0667°N, 82.9833°W, 2200m, 27 Feb 2005 (R. S. Anderson); ***Puntarenas***: Altamira Biological Station, 9.02922°N, 83.00813°W, 1400m, 1 Jun 2007 (M. G. Branstetter); 4km NE Altamira Biological Station, 9.06653°N, 82.98173°W, 1900m, 31 May 2007 (M. G. Branstetter); 5.1km NE Altamira Biological Station, 9.067°N, 82.983°W, 2100m, 31 May 2007 (M. G. Branstetter); Cerro Gemelo, 9.050°N, 82.933°W, 2400m, 3 Jul 1995 (J. Longino); Est. Biol. Los Llanos, 10.3049°N, 84.8373°W, 1150m, 28 Feb 2004 (J. Longino); Est. La Casona, Res Biol. Monteverde, 10.30°N, 84.80°W, 1520m, 9–11 Aug 1991 (Ugalde & Philips); Est. Pittier, 9.0333°N, 82.9667°W, 1670m, 28 Jun 1995 (J. Longino); Fila Cruces, nr San Vito, 8.783°N, 83.050°W, 1200m, 29 Jun 1995 (J. Longino); Las Alturas Biological Station, 8.94997°N, 82.83375°W, 1800m, 27 May 2007 (M. G. Branstetter); 4km NNW Las Alturas, 8.983°N, 82.850°W, 2090m, 21 Mar 1990 (P. S. Ward); 6km WNW Las Alturas, 8.967°N, 82.883°W, 1650m, 20 Mar 1990 (P. S. Ward); Las Cruces Biological Station, 8.78658°N, 82.95987°W, 1150m, 23 May 2007 (M. G. Branstetter); Monteverde, 10.30°N, 84.80°W, 1400m, 21 Dec 1986 (J. Longino); Monteverde, 10.30°N, 84.8°W, 1600m, 30 Apr 1989 (J. Longino); 13km SSW Pto. Jimenez, 8.40667°N, 83.32822°W, 130m, 10 Mar 2008 (J. Longino); Rancho Quemado, Osa Peninsula, 8.70°N, 83.55°W, 200m, 15 Dec 1990 (J. Longino); Res. Biol. Carara, 9.783°N, 84.600°W, 500m, 26 Jul 1985 (J. Longino); Valle del Silencio, Cerro Hoffman, 9.0781°N, 82.9767°W, 2300m, 27 Feb 2005 (R. S. Anderson); ***San José***: 1km N La Ese, 9.450°N, 83.717°W, 1400m, 5 Aug 1985 (P. S. Ward); 2km E San Gerardo, 9.467°N, 83.583°W, 1600m, 4 Aug 1985 (P. S. Ward); **ECUADOR:** ***Manabí***: 73km NE Chone, 85km W Sto. Domingo, [ca. 0.363°S, 79.739°W], 300m, 12 Jun 1976 (S. & J. Peck); 78km NE Chone, [ca. 0.363°S, 79.739°W], 450m, 9 Jun 1976 (S. & J. Peck); **NICARAGUA: *Jinotega***: RN Cerro Kilambé, 13.56754°N, 85.69690°W, 1420m, 23 May 2011 (LLAMA); RN Cerro Saslaya, 13.77174°N, 85.01295°W, 1110m, 12 May 2011 (LLAMA); ***Matagalpa***: RN Cerro Musún, 12.96067°N, 85.23326°W, 750m, 1May2011 (LLAMA); **PANAMA: *Bocas del Toro***: Almirante, [ca. 9.295°N, 82.397°W], 27 Mar 1959 (H. S. Dybas); 6km SE Buena Vista, 8.783°N, 82.183°W, 800m, 14–16 Jul 1987 (D. M. Olson); Cerro Pata de Macho, 8.833°N, 82.400°W, 2020m, 8 Aug 1987 (D. M. Olson); Fortuna-Chiriquí Grande Road, 8.7833°N, 82.1833°W, 800m, 16 Jul 1987 (D. M. Olson);***Chiriquí***: Boquete, Chiriquí Mts., [ca. 8.775°N, 82.432°W], 10 Mar 1923 (F. M. Gaige); Cerro Pata de Macho, 8.8833°N, 82.3833°W, 1500m, 23 Jul 1987 (D. M. Olson); 2km W Cerro Punta, [ca. 8.856°N, 82.590°W], 20 May 1977 (S. B. Peck); 24km W El Hato del Volcán, [ca. 8.833°N, 82.754°W], 1160m, 26–27 Jun 1976 (A. F. Newton); Finca Hartmann, 2km N Santa Clara, [ca. 8.833°N, 82.750°W], 1200m, 20 May 1977 (S. & J. Peck); N side Volcan Baru, 8.833°N, 82.567°W, 1950m, 28 Jul 1987 (P. S. Ward); ***Darien***: Cana, 7.717°N, 77.700°W, 1050m, 25 Aug 1987 (D. M. Olson); ***Panama***: Cerro Campana, [ca. 8.73°N, 79.97°W], 975m, 14–23 Feb 1976 (A. F. Newton).

### Stenamma stictosomum sp. n.

urn:lsid:zoobank.org:act:8E5ED2B1-FCD9-44A6-96D7-EBAA35F50A68

http://species-id.net/wiki/Stenamma_stictosomum

**Figure 157.**
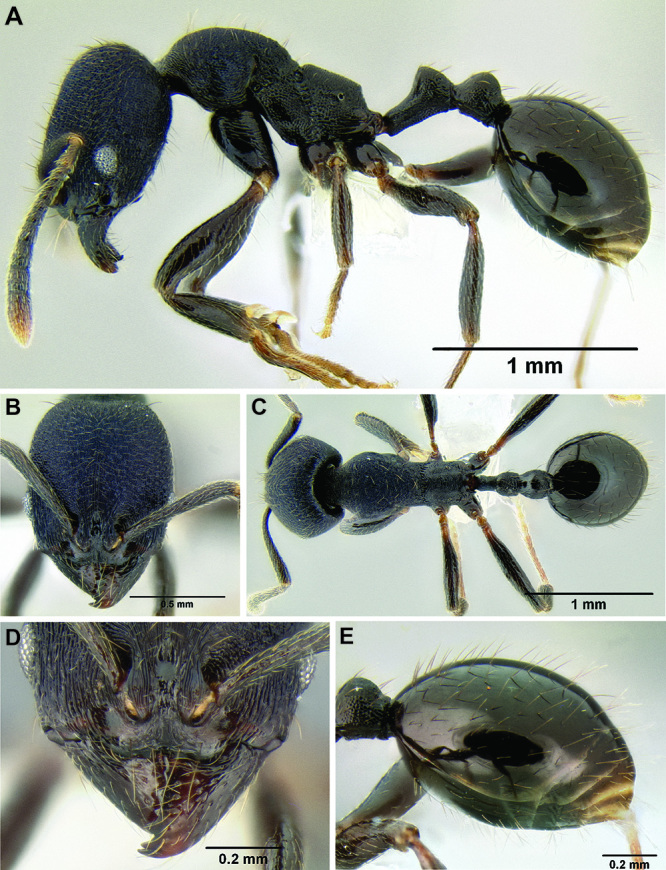
*Stenamma stictosomum* holotype worker (CASENT0605499) **A** Profile **B** Face **C** Dorsum **D** Anterior clypeal margin in anterodorsal view **E** Gaster.

**Figure 158.**
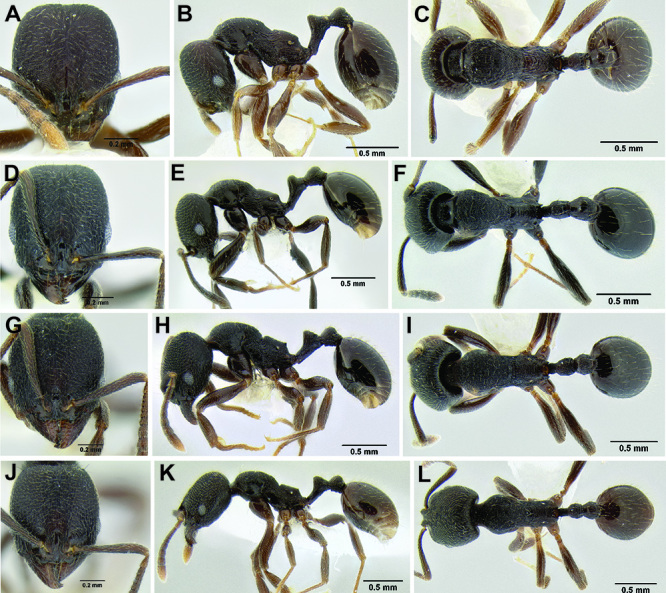
*Stenamma stictosomum* worker variants. Face, profile, dorsal views **A–C** Variant 1 (CASENT0126245) **D–F** Variant 2 (CASENT0606221) **G–I** Variant 3 (CASENT012624) **J–L** Variant 4 (CASENT0604636).

**Figure 159.**
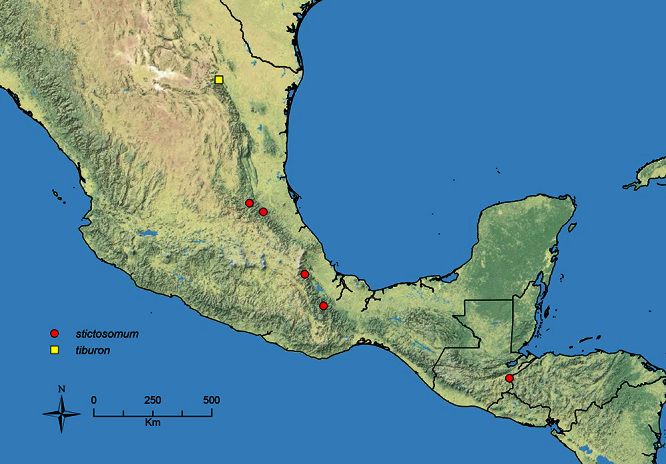
Distribution map of *Stenamma stictosomum* (circles) and *Stenamma tiburon* (squares).

Worker: Figures 157 , 158 ; Map: Figure 159

Stenamma mgb47 Branstetter, 2012: phylogeny.

#### Type material.

*Holotype worker*. MÉXICO, Oaxaca: 20.6km SW Valle Nacional, 17.60404°N, 96.37786°W ±60m, 1740m, 13 Aug 2009, cloud forest, ex sifted leaf litter (M. G. Branstetter, collection MGB1390) [USNM, specimen CASENT0605499]. *Paratypes*: same data as holotype [1w, UNAM, CASENT0605499], [1w, UCD, CASENT0605497].

#### Worker diagnosis.

Body color mostly black to dark brown, sometimes with a bluish glare in specimens with coarsely punctate sculpture; small- to medium-sized species (see HL, ML, PrW below); anterior clypeal margin broadly emarginate, with two blunt inner teeth (best viewed with mandibles open); basal margin of mandible sinuous, but without a basal notch or deep depression; setae on gastral tergites sparse, stout, and suberect, only sometimes with a few underlying short decumbent setae; eye relatively large (EL 0.15–0.18, REL 20–25), oval-shaped, with 8–9 ommatidia at greatest diameter; head and mesosomal sculpture densely punctate, densely carinulate, or carinulate-punctate, with carinulae merging into punctae; propodeal spines reduced to sharp angles or tubercles (PSL 0.09–0.11, PSI 0.9–1.2); frontal lobes well-developed, but not completely obscuring torular lobes in full-face view (FLD 0.15–0.22, FLI 23–28). *Similar species*: *Stenamma vexator*.

#### Geographic range.

Mexico (Atlantic slope) to Honduras.

#### Worker description.

(9 measured) HL 0.73–0.88 (0.88), HW 0.65–0.78 (0.78), FLD 0.15–0.22 (0.22), PCW 0.03–0.04 (0.03), SL 0.56–0.68 (0.68), EL 0.15–0.18 (0.18), ACL 0.51–0.61 (0.61), ML 0.93–1.11 (1.11), PrW 0.44–0.53 (0.53), PSL 0.09–0.11 (0.10), SDL 0.09–0.11 (0.11), PL 0.34–0.40 (0.39), PH 0.23–0.25 (0.25), PW 0.17–0.19 (0.19), PPL 0.20–0.24 (0.24), PPH 0.19–0.23 (0.23), PPW 0.20–0.23 (0.23), MFL 0.64–0.80 (0.77), MTL 0.51–0.65 (0.63), CI 86–94 (88), SI 79–92 (88), REL 20–25 (23), FLI 23–28 (28), PSI 0.9–1.2 (0.9), MFI 94–115 (101), ACI1 63–66 (63), ACI2 87–94 (89).

Small- to medium-sized species; general body color mostly black to dark brown, with appendages brown to orange-brown; setae dark golden brown; mandible with 6–7 teeth, consisting of 3 distinct apical teeth, a distinct basal tooth, and 2–3 teeth in between, which are often worn and indistinct; basal margin of mandible sinuous, but without a basal notch or deep depression; mandible mostly smooth and shining, with scattered piligerous punctae and striae; anterior clypeal margin broadly emarginate, with two blunt inner teeth (best viewed with mandibles open); surface of median clypeal lobe somewhat rough (irregular depressions, punctae), with a pair of faint longitudinal carinulae that diverge anteriorly, apex of lobe with a short transverse carinula, remainder of clypeus mostly smooth and shiny; posterior extension of clypeus between antennal insertions of moderate width (PCW 0.03–0.04), sides subparallel; frontal lobes moderately developed (FLD 0.15–0.22, FLI 23–28), but not completely covering torular lobes in full-face view; head subrectangular to slightly oval-shaped (CI 86–94), with posterior margin slightly to distinctly depressed medially; eye relatively large (EL 0.15–0.18, REL 20–25), oval-shaped, with 8–9 ommatidia at greatest diameter; head coarsely punctate (type population), or carinulate punctate, or mostly carinulate; in specimens with mainly carinulate sculpture, piligerous punctae are present in interstices, especially toward lateral margins; scape somewhat short (SI 79–92), not reaching posterior margin of head when laid back; scape surface mostly smooth and shiny (type population), or rougher, with dense piligerous punctae and carinulae; funiculus with a somewhat distinct 4-segmented antennal club; mesosoma sculpture variable, similar to head sculpture, either coarsely punctate (type population), carinulate punctate, or mostly carinulate, with carinulae mostly longitudinal in direction, but sometimes transverse on pronotal dorsum and propodeal dorsum, or arcuate on pronotal side; propodeal declivity always transversely carinulate; promesonotum in profile low-domed and roughly symmetrical; metanotal grove distinct to somewhat indistinct, of moderate to shallow width and depth; propodeal spines reduced to sharp angles or short tubercles (PSL 0.09–0.11, PSI 0.9–1.2); petiole and postpetiole robust, with nodes somewhat bulging; petiole of moderate length (PL/HW 0.45–0.58); petiolar node in profile usually subconical (type population), roughly symmetrical, with dorsum forming a well-defined apex that points vertically, but sometimes dorsum with a broadly rounded top that points slightly posteriad; postpetiole in profile subspherical, usually similar in same size to petiolar node (PPH/PH 0.79–0.93); petiole and postpetiole punctate, with anterior faces of nodes smooth and shiny; gaster mostly smooth and shiny, with scattered piligerous punctate; most of body dorsum with a layer of medium to short standing pilosity; scape with a uniform layer of subdecumbent to decumbent setae; gaster with a sparse layer of stout suberect setae, and sometimes a few underlying short decumbent setae; leg setae mostly appressed, with suberect setae on coxae and femoral venters.

#### Queen.

Unknown.

#### Male.

Unknown.

#### Biology.

A rare species, *Stenamma stictosomum* is a cloud forest specialist known from a few samples of sifted leaf litter, a beating sample, and from quarantine in Brownsville, TX. The latter specimen was found on orchids being shipped to the United States from either Oaxaca or San Luis Potosí, México. Collections range from 1450–1750 m elevation. The presence of *Stenamma stictosomum* on orchids and on vegetation (beating sample) suggests that it may nest or at least forage arboreally. This would help explain why the species appears to be so rare, despite being rather widespread.

#### Comments.

*Stenamma stictosomum* may form a complex of species, as evidenced by the significant amount of morphological variation among populations. However, an adequate assessment of species boundaries is impossible because so little material has been collected of the species (10 specimens from 7 localities). Consequently, I describe a single species here, but discuss some of the among population variation below.

The type population (20.6km SW Valle Nacional) is the most distinctive, with the head and mesosoma entirely coarsely punctate and without well-defined carinulae (except for the propodeal dorsum and declivity). In the right lighting, these specimens give off a bluish reflectance from the surface sculpturing.

Variant 1 (Figure 158A–C) is a specimen from quarantine, likely originating from Oaxaca or San Luis Potosí, Mexico. It is similar to the type population, but has reduced sculpture overall (less coarse, more polished) and carinulate punctate sculpture on the promesonotum.

Variant 2 (Figure 158D–F) is from Guatemala (a single specimen). It is similar to the type population, except that the punctae are less coarse (more polished, somewhat effaced) and there are smooth patches of cuticle on the side of the head and pronotum. Also, the petiolar node in profile has a rounded dorsum that points slightly posteriad.

Variant 3 (Figure 158G–I) is from Veracruz, Mexico (a single specimen) and is coarsely sculptured, but the sculpture is carinulate punctate, with the carinulae emerging out of the borders of the punctae. The carinulae on the promesonotal dorsum are indistinctly transverse in orientation and the carinulae on the pronotal side are arcuate.

Variant 4 (Figure 158J–L) is from Hidalgo and San Luís Potosí, Mexico. All specimens are similar in that the sculpture is mainly carinulate or rugulose (mostly longitudinal in orientation), rather than punctate. Punctae are visible in the interstices, but they do not form the dominant sculpture type. Also, the petiolar and postpetiolar nodes are less bulging, appearing more average in form, with the postpetiole clearly smaller than the petiolar node.

#### Material examined.

**GUATEMALA:** ***Zacapa***: 2km SE La Unión, 14.95284°N, 89.27655°W, 1450m, 14 May 2009 (LLAMA); **MÉXICO:** ***Hidalgo***: [Tlanchinol], 43km SW Huejutla, [ca. 20.988°N, 98.662°W], 1500m, 14 Jun 1983 (S. & J. Peck); ***Oaxaca***: Oaxaca or San Luis Potosí, 19 Apr 1961 (quarantine in Brownsville, TX); 20.6km SW Valle Nacional, 17.60404°N, 96.37786°W, 1740m, 13 Aug 2009 (M. G. Branstetter); ***San Luis Potosí***: 20km W Xilitla, [ca. 21.293°N, 99.194°W], 1600m, 12 Jun 1983 (S. & J. Peck); ***Veracruz***: 10km S Orizaba, 18.750°N, 97.083°W, 1500m, 19 Mar 1985 (P. S. Ward).

### Stenamma tiburon sp. n.

urn:lsid:zoobank.org:act:BBDA7D59-B962-4DE8-AA3D-13AEDAAD3D30

http://species-id.net/wiki/Stenamma_tiburon

**Figure 160.**
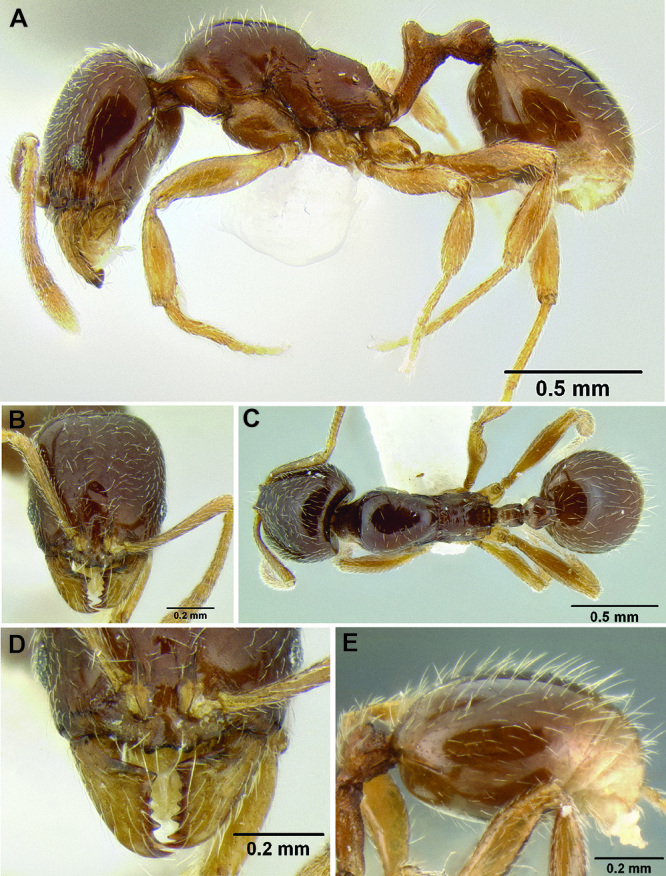
*Stenamma tiburon* holotype worker (CASENT0620965) **A** Profile **B** Face **C** Dorsum **D **Anterior clypeal margin in anterodorsal view **E** Gaster.

**Figure 161.**
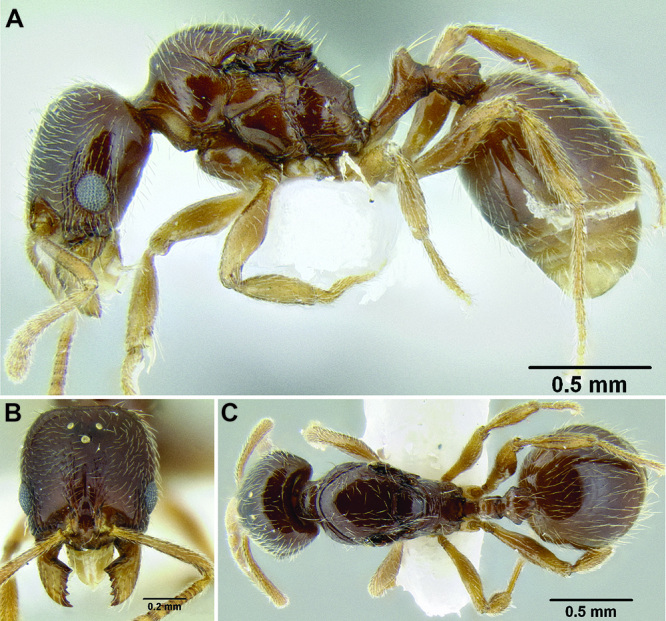
*Stenamma tiburon* paratype queen (CASENT0622423) **A** Profile **B** Face **C** Dorsum.

Worker: Figure 160 ; Queen: Figure 161 ; Map: Figure 159

#### Type material.

*Holotype worker*. MÉXICO: Nuevo León, near Monterrey, Mesa de Chipinque, [ca. 25.61°N, 100.36°W], 1650m, 22 Jun 1969 (S. & J. Peck, collection B164) [USNM, pin CASENT0620965, top specimen]. *Paratypes*: same data as holotype [2w, LACM, CASENT0193030], [1w, MCZ, CASENT0194031], [1dq, 1w, MGBPC, CASENT0622423], [2w, UNAM, CASENT0194031], [1w, USNM, CASENT0620965, bottom specimen].

#### Worker diagnosis.

Integument brown (probably darker in recently collected specimens); small to medium-sized species (see HL, ML, PrW below); head, mesosoma, and gaster mostly smooth and shining, with petiole and postpetiole punctate; promesonotum in profile distinctly asymmetrical, with anterior face gently rounded, dorsal surface flat or only slightly curving, and posterior face short, straight and forming a relatively sharp angle with dorsal surface; median lobe of clypeus bicarinate, projecting, and with a broad and rather deep median emargination at anterior margin; basal margin of mandible straight, without notch or substantial depression; petiolar node distinctly angled posteriad; postpetiole in dorsal view, with distinct mesolateral angles and a longitudinal lobe, giving anterior half of postpetiole a distinct pinched-in appearance; eye of moderate size (EL 0.10–0.12, REL 18–19), oval shaped, with 5–6 ommatidia at greatest diameter; setae on gastral tergites of moderate length and density, suberect to subdecumbent; propodeal spines absent, forming at most an obtuse angle in profile view (PSL 0.06–0.07, PSI 1.0-1.3); frontal lobes narrow (FLD 0.14–0.15, FLI 24–25), not obscuring torular lobes in full-face view (FLD 0.14-0.15, FLI 24-25). *Similar species*: *Stenamma pelophilum*.

#### Geographic range.

Northeastern Mexico.

#### Worker description.

(4 measured) HL 0.62–0.69 (0.67), HW 0.55–0.63 (0.59), FLD 0.14–0.15 (0.15), PCW 0.03–0.05 (0.05), SL 0.50–0.54 (0.52), EL 0.10–0.12 (0.11), ACL 0.48–0.51 (0.48), ML 0.79–0.86 (0.83), PrW 0.38–0.42 (0.40), PSL 0.06–0.07, SDL 0.05–0.07, PL 0.30–0.32 (0.30), PH 0.19–0.21 (020), PW 0.12–0.14 (0.13), PPL 0.16–0.18 (0.16), PPH 0.16–0.17 (0.16), PPW 0.20–0.21 (0.20), MFL 0.53–0.59 (0.55), MTL 0.44–0.48 (0.46), CI 88–91 (89), SI 86–92 (88), REL 18–19 (19), FLI 24–25 (25), PSI 1.0–1.3 (1.0), PI 51–57 (51), MFI 103–108 (107), ACI1 67–70 (70), ACI2 93–97 (93).

Small- to medium-sized species; general body color brown to light brown, with extremities becoming yellow-brown (note that all studied specimens are over 40 years old, the color of fresh specimens must be darker); setae golden; mandible with 6 teeth, consisting of 3 distinct apical teeth, a somewhat projecting basal tooth, and 2 inner teeth, which are often worn and indistinct; basal margin of mandible relatively straight, without notch or significant depression; dorsal surface of mandible mostly smooth and shining, with scattered piligerous punctae and a few short basal striae; median lobe of clypeus bicarinate, projecting, and with a broad and rather deep median emargination at anterior margin, apex of lobe with a transverse carina, area between carinae distinctly depressed; carinae on median lobe forming a distinctive triangular shape in anterodorsal view; remaining surface of clypeus mostly smooth and shiny; posterior extension of clypeus between frontal lobes somewhat narrow (PCW 0.03–0.05), with subparallel sides; frontal lobes narrow, not obscuring torular lobes in full-face view; head roughly oval-shaped (CI 88–91), posterior margin flat to slightly concave, never greatly depressed medially; eye of moderate size (EL 0.10–0.12, REL 18–19), oval-shaped, with 5–6 ommatidia at greatest diameter; face nearly completely smooth and shiny, with scattered piligerous punctae and a few longitudinal carinulae around frontal carinae and on genae; scape distinctly shorter than HW (SI 86–92), not quite reaching posterior margin of head when laid back; scape surface mostly smooth and shiny, except for scattered piligerous punctures and faint striae; flagellum with distinct 4-segmented antennal club; mesosoma mostly smooth and shining, except metanotal groove with several longitudinal carinae, metapleuron with faint punctae, and propodeal dorsum and declivity with faint transverse carinulae; promesonotum in profile distinctly asymmetrical, with anterior face gently rounded, dorsal surface flat or only slightly curving, and posterior face short, straight and forming a relatively sharp angle with dorsal surface; metanotal groove distinct, but not very deep; propodeal spines essentially absent (PSL 0.06–0.07, PSI 1.0–1.3), forming at most obtuse angles at transition between dorsal and declivitous faces of propodeum in profile; petiole of moderate length (PL/HW 0.51–0.57), node of moderate size (PH/PL 0.62–0.67) and distinctly angled posteriad, with anterior face slightly longer and more sloping than posterior face, node dorsum broadly rounded; postpetiole in profile asymmetrical, with long sloping anterior face and short nearly vertical posterior face; postpetiole in dorsal view with distinctive mesolateral angles and a longitudinal lobe, giving the anterior half of postpetiole a distinct pinched-in appearance; anterior faces of petiole and postpetiole smooth and shiny, remaining surfaces faintly punctate; most of body with a thin layer of short to medium length standing setae; pilosity on gastral tergites somewhat bilayered, with a layer of longer suberect setae, and a layer of equally dense subdecumbent setae; facial setae short and mostly decumbent; setae on scape subdecumbent to decumbent; setae on legs suberect to appressed, with longer suberect setae on femoral venters and coxae.

#### Queen description.

(1 measured) HL 0.69, HW 0.63, FLD 0.15, PCW 0.05, SL 0.53, EL 0.17, ACL 0.52, ML 0.93, PrW 0.50, PSL 0.09, SDL 0.08, PL 0.34, PH 0.22, PW 0.14, PPL 0.19, PPH 0.19, PPW 0.24, MFL 0.59, MTL 0.49, CI 92, SI 83, REL 27, FLI 24, PSI 1.2, MFI 107, ACI1 68, ACI2 98.

Same as worker except for standard queen modifications. See Figure 161.

#### Male.

Unknown.

#### Biology.

*Stenamma tiburon* is known only from a single Berlese sample collected from forest at 1650 m elevation.

#### Comments.

The combination of sculpture, clypeus structure, and postpetiole structure make *Stenamma tiburon* a very distinctive species that should not be confused with any other MAC species. I include *Stenamma pelophilum* as a similar species only because both species occur in the same couplet of the key, but these species are very different and can be easily separated by comparing clypeus structure.

It should be noted that this species is known from only nine specimens that were collected in 1969. Thus, the coloration described here is certainly different from what fresh specimens probably look like. Further more, this species has not been included in molecular analyses and it is somewhat uncertain to which species it is most closely related. However, because the postpetiole has a distinct dorsal lobe I include *Stenamma tiburon* in the *lobinodus* species group (diagnosis of group given under *Stenamma lobinodus* above) and I hypothesize based on distribution and morphology that is sister to *Stenamma lobinodus*.

#### Material examined.

Known only from the type locality.

### Stenamma tico sp. n.

urn:lsid:zoobank.org:act:65486F53-70F0-40A3-90EC-F4C24090422F

http://species-id.net/wiki/Stenamma_tico

**Figure 162.**
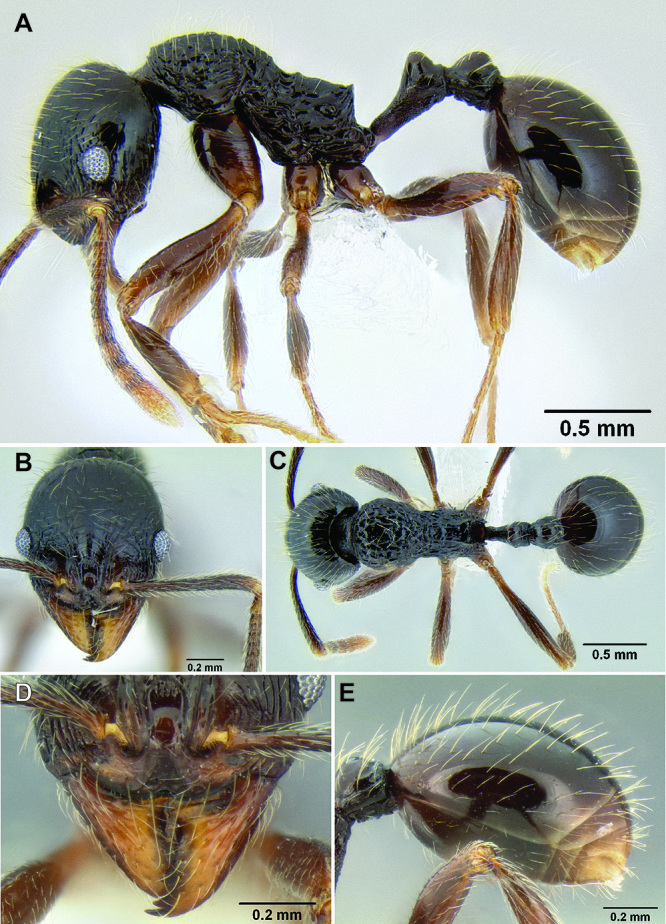
*Stenamma tico* holotype worker (CASENT0622416) **A** Profile **B** Face **C** Dorsum **D** Anterior clypeal margin in anterodorsal view **E** Gaster.

**Figure 163.**
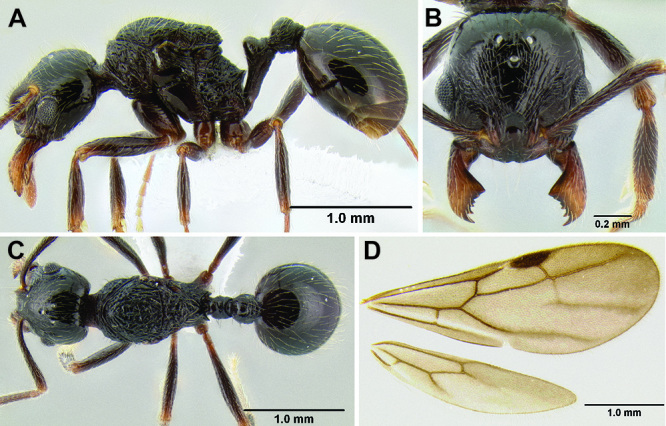
*Stenamma tico* **A** Queen (CASENT0600122), profile **B** Same, head **C** Same, dorsum **D **Queen (INB0003732299).

**Figure 164.**
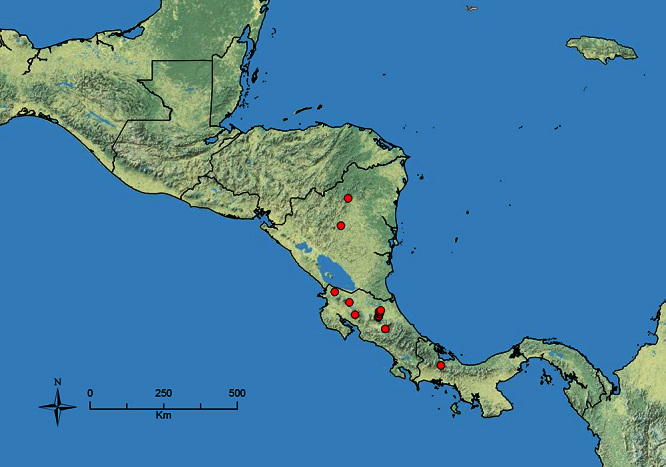
Distribution map of *Stenamma tico*.

Worker: Figure 162 ; Queen: Figure 163 ; Map: Figure 164

#### Type material.

*Holotype worker*. COSTA RICA, Heredia Prov.: 10km NE Vara Blanca, 10.233°N, 84.067°W, 1500m, 9 Mar 2005 (Project ALAS, collection 15/WF/02) [INBio,specimen CASENT0622416]. *Paratypes*:same data as holotype [1w, CAS, CASENT0622417], [1w, FMNH, CASENT0600104], [1w, ICN, CASENT0623501], [1w, INBio, CASENT0623502], [1w, JTLC, CASENT0623503], [1w, LACM, CASENT0623504], [1w, MGBPC, CASENT0623505], [1w, MCZ, CASENT0623506], [1w, MZSP, CASENT0623507], [1w, UCD, CASENT0622414], [1w, USNM, CASENT0622415].

#### Worker diagnosis.

Integument shining, largely black to red-black; medium-sized species (see HL, ML, PrW below); head mostly smooth and shiny; mesosoma reticulately costate to coarsely rugoreticulate; propodeal spines reduced to small upward projecting points, or sharp right angles at interface between dorsal and declivitous faces of propodeum (PSL 0.14–0.18, PSI 1.4-1.9); eyes large and somewhat bulging (EL 0.15–0.18, REL 23-24), with 7–10 ommatidia at greatest diameter; anterior margin of clypeus with shallow median emargination; basal margin of mandible straight, without notch or substantial depression; pilosity on gastral dorsum long, flexuous, and relatively sparse. *Similar species*: *Stenamma diversum*, *Stenamma lobinodus*.

#### Geographic range.

Nicaragua to Panama.

#### Worker description.

(11 measured) HL 0.71-0.85 (0.84), HW 0.63-0.76 (0.76), FLD 0.22-0.27 (0.26), PCW 0.06-0.07 (0.07), SL 0.60-0.77 (0.77), EL 0.15-0.18 (0.18), ACL 0.54-0.68 (0.68), ML 0.91-1.11 (1.11), PrW 0.48-0.58 (0.57), PSL 0.14–0.18 (0.14), SDL 0.08–0.10 (0.09), PL 0.37-0.46 (0.44), PH 0.21-0.26 (0.26), PW 0.16–0.22 (0.20), PPL 0.19–0.25 (0.23), PPH 0.19–0.25 (0.24), PPW 0.20–0.25 (0.25), MFL 0.72-0.91 (0.91), MTL 0.56-0.71 (0.71), CI 87-93 (91), SI 95-103 (101), REL 23-24 (24), FLI 33-36 (34), PSI 1.4-1.9 (1.4), MFI 82-88 (84), ACI1 63-66 (64) ACI2 84–91 (88).

Medium-sized species; general body color black to red-black, with brown patches on waist and gaster; mandibles, clypeus and appendages dark brown to yellow-brown; setae golden; mandible with 6–7 teeth, consisting of 3 distinct apical teeth, a basal tooth, and 2–3 inner teeth, which are often worn and indistinct; basal margin of mandible straight, without any notch or significant depression; dorsal surface of mandible mostly smooth and shiny, with scattered piligerous punctae and variable number of basal striae; median lobe of clypeus with a pair of vestigial longitudinal carinulae and/or several ill-defined rugulae, apex of lobe with a short transverse carinula, remainder of clypeus smooth and shiny; posterior extension of clypeus between frontal lobes rather broad (PCW 0.06-0.07), with subparallel sides; frontal lobes relatively well developed, but not markedly expanded dorsolaterally (as in *Stenamma diversum*), nor completely obscuring the underlying torular lobes in full-face view; head roughly oval-shaped, slightly, but distinctly longer than broad (CI 87-93), posterior margin gently convex, never depressed medially; eyes relatively large and somewhat bulging (EL 0.15–0.18, REL 23-24), oval-shaped, with 7–10 ommatidia at greatest diameter; face mostly smooth and shining, with variable amount of weak carinulae/rugulae and punctae confined to lower ¾ of head; most specimens with some carinulae around antennal sockets and on gena, others with more extensive carinulae fanning outward from frontal carinae, sometimes with reticulae between antennal carinae and eyes; scape relatively long (SI 95-103), reaching and slightly surpassing posterior margin of head when laid back; dorsal surface of scape striate; flagellum with distinct 4-segmented antennal club; mesosoma shiny, almost entirely reticulately costate to coarsely rugoreticulae; propodeal declivity smooth, sometimes with a few transverse carinulae; promesonotum in profile low-domed and roughly symmetrically, anterior slope a little steeper and longer than posterior slope; propodeal spines reduced to small upward projecting points, or only forming sharp right angles where dorsal and declivitous surfaces of propodeum meet (PSL 0.14–0.18, PSI 1.4-1.9); petiole moderately long (PL/HW 0.57–0.63) and robust, node roughly half length of petiole and asymmetrical, with anterior face long and gradually sloping, and posterior face nearly vertical, but slightly angled so that apex of petiole points posteriad; dorsum of petiolar node viewed from posterior side weakly rounded; postpetiole in profile similar in size to petiolar node (PPH/PH 0.91–0.97), roughly circular, although with anterior face slightly longer and more sloping than posterior face; petiole and postpetiole shiny, nodes mostly smooth, with some rugulae and faint punctae on sides; venter of petiole and postpetiole weakly punctate; gaster mostly smooth and shiny, with scattered piligerous punctae; most of body with single layer of long standing setae; setae on legs and scapes varying from predominately suberect to mostly decumbent; coxae and femoral venters always with a row of longer suberect setae.

#### Queen description.

(5 measured) HL 0.71–0.85 (0.79), HW 0.63–0.76 (0.73), FLD 0.22–0.27 (0.24), PCW 0.07–0.09 (0.07), SL 0.64–0.78 (0.69), EL 0.20–0.24 (0.21), ACL 0.53–0.66 (0.60), ML 1.05–1.29 (1.12), PrW 0.57–0.70 (0.60), PSL 0.18–0.21 (0.19), SDL 0.09–0.11 (0.10), PL 0.43–0.53 (0.48), PH 0.24–0.29 (0.25), PW 0.20–0.25 (0.22), PPL 0.20–0.26 (0.23), PPH 0.23–0.29 (0.25), PPW 0.25–0.31 (0.26), MFL 0.77–0.94 (0.81), MTL 0.61–0.73 (0.64), CI 91–93 (93), SI 93–95 (94), REL 28–30 (28), FLI 34–37 (24), PSI 2.0–2.2 (2.0), MFI 87–90 (90), ACI1 63–66 (64), ACI2 82–90 (87).

Same as worker except for standard queen modifications and the following: face usually more sculptured, with light fan of rugulae/carinulae extending from frontal lobes to ocelli, sculpture around antennal sockets more distinct; costae on mesoscutum with a decidedly longitudinal orientation, but often wavy, and usually with some reticulation anteriorly; costae on side of promesonotum longitudinal in orientation; mesopleuron mostly smooth and shiny; propodeal spines always present, short, projecting dorsoposteriad (PSL 0.18–0.21, PSI 2.0–2.2); wing venation as in Figure 163D.

#### Male.

Unknown.

#### Biology.

*Stenamma tico* is restricted to wet forest habitats from sea level to about 1500 m, being most abundant at mid elevations. Nests have never been collected of this species, with nearly all specimens coming from extracts of sifted leaf litter taken from the forest floor. Once at a site in Nicaragua I found a trail of workers migrating across a clay bank in montane wet forest. Although a nest site could not be found, the number of workers involved suggests that they were relocating the nest. The presence of *Stenamma tico* on clay banks indicates that it may have biological characteristics very similar to the closely related *Stenamma diversum*, which is known to nest in clay bank environments (see biology of *Stenamma diversum* above). *Stenamma tico* has large, bulging eyes and it may be that it is more active at night than *Stenamma diversum*.

#### Comments.

*Stenamma tico* and *Stenamma diversum* together form the *diversum* species group (diagnosis given under *Stenamma diversum* above).

Within its range *Stenamma tico* is unlikely to be confused with any other species. Only in northern Nicaragua does it occur in sympatry with its sister species *Stenamma diversum*. As mentioned above (see comments under *Stenamma diversum*), *Stenamma tico* can be separated from *Stenamma diversum* by its smaller propodeal spines and narrower frontal lobes. *Stenamma tico* also can be separated from *Stenamma diversum*, as well as from the similar looking *Stenamma lobinodus*, by its relatively long scape (SI > 95 vs. < 93) and metafemur (MFI < 89 vs. > 94).

*Stenamma tico* shows some elevational variation with specimens from higher localities becoming larger, darker and more sculptured (especially on the face). This variation appears to be continuous, and thus is unlikely due to the existence of cryptic species.

#### Material examined.

**COSTA RICA:** ***Cartago***: Navarro Farm, [ca. 9.884°N, 83.883°W], [ca. 1100m] (W. M. Mann); ***Guanacaste***: Est. Pitilla, 9km S Sta. Cecilia, P.N. Guanacaste, [ca. 10.989°N, 85.426°W], 700m (C. Moraga); P.N. Volcán Tenorio, Volcán Tenorio, 10.6764°N, 84.9775°W, 1650m (J. Azofeita); ***Heredia***: 9km NE Vara Blanca, 10.23°N, 84.10°W, 1450-1550m (ALAS); 11km ESE La Virgen, 10.35°N, 84.05°W, 250-350m (ALAS); 11km SE La Virgen, 10.33°N, 84.07°W, 550m (ALAS); 16km SSE La Virgen, 10.267°N, 84.083°W, 1050–1150m (ALAS); 12km N Vol. Barba, 10.250°N, 84.083°W, 1420m (J. Longino); Est. Biol. La Selva, 10.433°N, 84.017°W, 50-150m (J. Longino); P.N. Braulio Carrillo, 10.40°N, 84.03°W, 150-180m (TEAM); P.N. Braulio Carrillo, 10.40°N, 84.07°W, 300m (TEAM); ***Puntarenas***: Monteverde, 10.30°N, 84.80°W, 1500m (J. Longino); **NICARAGUA: *Jinotega***: P.N. Cerro Saslaya, 13.77172°N, 85.01277°W, 1110m (LLAMA); ***Matagalpa***: R.N. Cerro Musún, 12.96071°N, 85.23250°W, 750m (LLAMA); **PANAMA: *Bocas del Toro***: Fortuna-Chiriquí Grande Road, 8.783°N, 82.183°W, 500m (D. M. Olson).

### Stenamma vexator sp. n.

urn:lsid:zoobank.org:act:918C9F3B-9C8E-4B71-924A-060CA2CD8D3A

http://species-id.net/wiki/Stenamma_vexator

**Figure 165.**
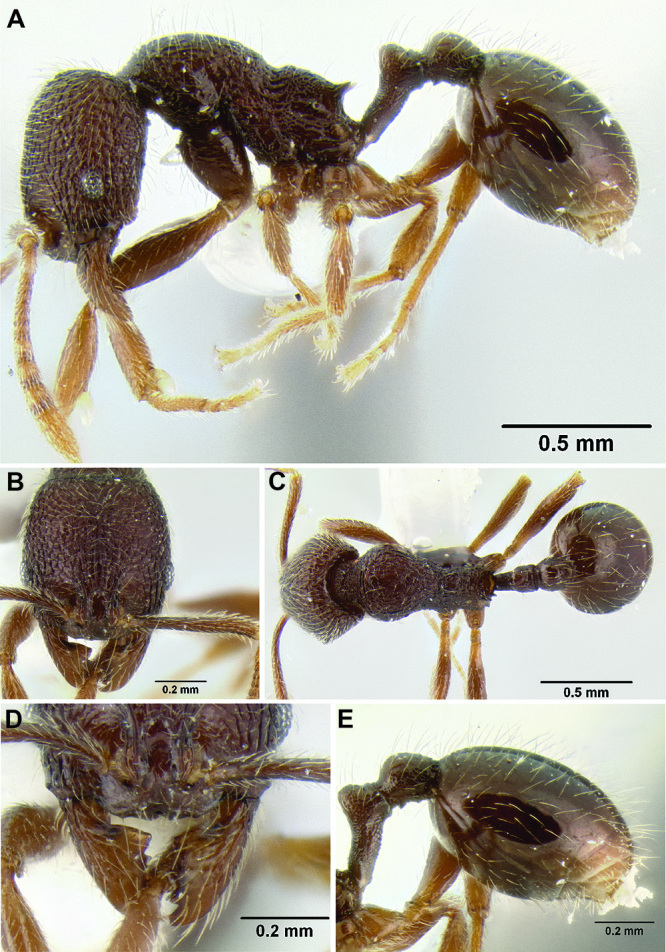
*Stenamma vexator* holotype worker (CASENT0126485) **A** Profile **B** Face **C** Dorsum **D **Anterior clypeal margin in anterodorsal view **E** Gaster.

**Figure 166.**
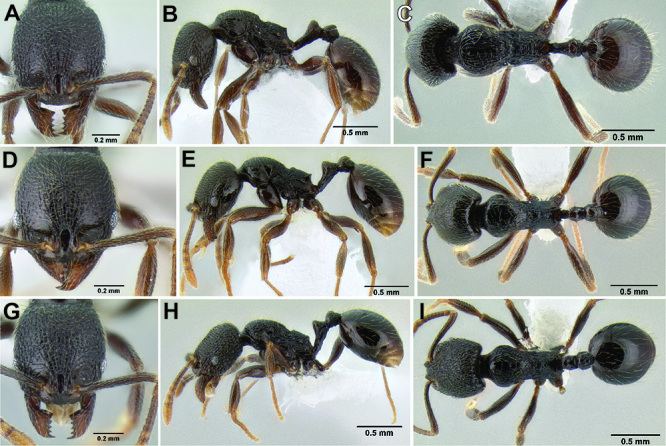
*Stenamma vexator* worker variants. Face, profile, and dorsal views **A–C** Variant 1 (CASENT0604610) **D–F** Variant 2 (CASENT0605506) **G–I** Variant 3 (CASENT060472).

**Figure 167.**
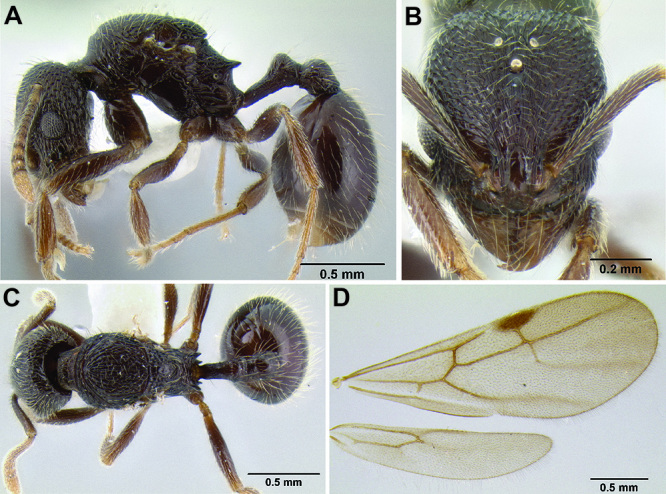
*Stenamma vexator* paratype queen (CASENT0126490) **A** Profile **B** Face **C** Dorsum **D** Wings.

**Figure 168.**
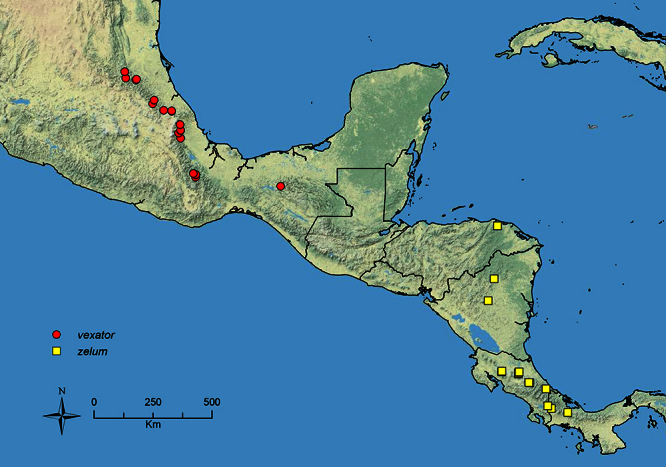
Distribution map of *Stenamma vexator* (circles) and *Stenamma zelum* (squares).

Worker: Figures 165 , 166 ; Queen: Figure 167 ; Map: Figure 168

Stenamma mgb39 [variant 2 below] Branstetter, 2012: phylogeny.

#### Type material.

*Holotype worker*. MÉXICO, Veracruz: 1.2mi [1.9km] S Huatusco, [ca. 19.193°N, 96.956°W], 1344m, 2-5 Aug 1969, cloud forest, [Berlese sample of sifted leaf litter] (S. & J. Peck, collection ANTC18239) [USNM, specimen CASENT0126485]. *Paratypes*: same data as holotype [1dq, 1w, CAS, CASENT0126500, CASENT0126495], [1dq, 1w, FMNH, CASENT126502, CASENT0126499], [1dq, 1w, INBio, CASENT0126503, CASENT0126506], [1w, JTLC, CASENT0126507], [1dq, 1w, LACM, CASENT0126487, CASENT0126486], [1dq, 1w, MGBPC, CASENT0126511, CASENT0126512], [1dq, 1w, MCZ, CASENT0126523, CASENT0126496], [1w, MZSP, CASENT0126517], [1dq, 1w, UCD, CASENT0126520, CASENT0126501], [1dq, 1w, UNAM, CASENT0126513, CASENT0126498], [1dq, 1w, USNM, CASENT0126490, CASENT0126492].

#### Worker diagnosis.

Integument mostly black or dark brown to red-brown; small- to medium-sized species (see HL, ML, PrW below); anterior clypeal margin undulating, forming 2–4 blunt teeth; basal margin of mandible usually sinuous, with a slight basal depression, but without a tooth; face completely sculptured, mostly rugoreticulate; pilosity on gastral dorsum predominately suberect and relatively sparse; eye of moderate size (EL 0.10–0.15, REL 18–22), oval-shaped, with 5–8 ommatidia at greatest diameter; propodeal spines tuberculate to short (PSL 0.08–0.16, PSI 1.1–1.8), often robust; petiole in profile often distinctive, appearing somewhat elongate, with node broadly rounded and pointed posteriad, venter under node with a small concavity. *Similar species*: *Stenamma crypticum*, *Stenamma huachucanum*, *Stenamma stictosomum*.

#### Geographic range.

Mexico (Atlantic slope).

#### Worker description.

(29 measured) HL 0.62–0.78 (0.65), HW 0.55–0.71 (0.58), FLD 0.15–0.20 (0.16), PCW 0.04–0.06 (0.04), SL 0.50–0.67 (0.55), EL 0.10–0.15 (0.12), ACL 0.48–0.60 (0.50), ML 0.77–1.02 (0.85), PrW 0.39–0.49 (0.43), PSL 0.08–0.16, SDL 0.07–0.10 (0.09), PL 0.31–0.40 (0.34), PH 0.17–0.23 (0.19), PW 0.14–0.19 (0.15), PPL 0.15–0.22 (0.20), PPH 0.16–0.21 (0.17), PPW 0.17–0.22 (0.19), MFL 0.53–0.76 (0.60), MTL 0.45–0.62 (0.48), CI 89–96 (89), SI 85–98 (94), REL 18–22 (21), FLI 25–29 (28), PSI 1.1–1.8 (1.6), PI 53–59 (59), MFI 93–107 (97), ACI1 65–71 (68), ACI2 87–98 (92)

Small- to medium-sized species; general body color black, or dark brown to red-brown (type population), with appendages brown to orange-brown or yellow-brown, lighter in joints and toward extremities; setae golden brown; mandible with 6 teeth, 2 teeth nearest basal tooth sometimes more worn and less distinct, basal tooth often well-defined, projecting; basal margin of mandible usually sinuous, with a slight basal depression (type population), but without a tooth, some populations with basal margin almost straight; mandible mostly smooth, with scattered piligerous punctae, and some striations near base and along lateral surface; median lobe of clypeus with a pair of faint longitudinal carinulae that diverge anteriorly, apex of lobe with a short transverse carinula, remainder of clypeus mostly smooth; area in between median lobe and anterior clypeal margin forming a distinct concavity where mandibles insert; posterior extension of clypeus between frontal lobes of moderate width (PCW 0.04–0.06), with sides subparallel; frontal lobes of moderate width (FLD 0.15–0.20, FLI 25–29), not greatly covering torular lobes in full-face view; head roughly oval-shaped (CI 89–96), with posterior margin flat to slightly depressed medially; eye of moderate size (EL 0.10–0.15, REL 18–22), oval-shaped, with 5–8 ommatidia at greatest diameter; face usually strongly sculptured, mostly rugoreticulate, with some longitudinal rugae along midline (type population), some populations with sculpture somewhat polished and reticulate less distinct; scape of moderate length (SI 85–98), just reaching posterior margin of head when laid back; surface of scape mostly smooth, with scattered piligerous punctae; flagellum with distinct 4-segmented antennal club; mesosoma usually completely sculptured (type population), but some populations shinier overall and with pronotum mostly smooth and shiny; pronotal dorsum longitudinally rugose, with rugae dense to somewhat sparse and irregular, rugae usually becoming punctae and reticulae on dorsum of mesonotum; side of pronotum mostly punctate or rugose, often with a small to large patch of smooth cuticle on ventral half; mesopleuron and side of propodeum rugulose-punctate; propodeal dorsum with rugoreticulae near anterior margin, changing to transverse carinulae near posterior margin; promesonotum in profile low-domed, slightly asymmetrical, with apex posterior of midpoint, anterior declivity of pronotum sometimes forming a sharp transition with dorsum, sculpture on declivity punctate, contrasting with rugose dorsum; metanotal groove usually well-demarcated, of moderate depth and width; propodeal spines tuberculate to short (PSL 0.08–0.16, PSI 1.1–1.8), often somewhat robust (type population); petiole in profile often distinctive, appearing somewhat elongate (PL/HW 0.53–0.56), with node broadly rounded and pointed posteriad, venter under node often with a small concavity; peduncle sometimes noticeably slender, thickening toward node; postpetiole in profile subspherical, similar in size to petiolar node (PPH/PH 0.85–0.91); anterior face of petiolar and postpetiolar nodes smooth, remainder of waist mostly punctate; gaster mostly smooth and shiny, with scattered piligerous punctae; pilosity on head, short and bilayered, with some longer suberect setae, and many shorter decumbent setae, remainder of body dorsum with longer suberect setae; pilosity on gastral dorsum predominately suberect and relatively sparse; setae on scape mostly subdecumbent; setae on legs mostly subdecumbent to appressed, with some longer suberect setae on femoral venters and coxae.

#### Queen description.

(11 measured) HL 0.66–0.77 (0.66), HW 0.60–0.70 (0.60), FLD 0.17–0.20 (0.18), PCW 0.05–0.07 (0.05), SL 0.53–0.64 (0.53), EL 0.18–0.20 (0.18), ACL 0.50–0.60 (0.51), ML 0.94–1.13 (0.94), PrW 0.53–0.64 (0.54), PSL 0.14–0.17 (0.15), SDL 0.09–0.12 (0.09), PL 0.37–0.44 (0.38), PH 0.19–0.23 (0.20), PW 0.16–0.19 (0.17), PPL 0.18–0.22 (0.20), PPH 0.18–0.23 (0.19), PPW 0.20–0.24 (0.20), MFL 0.59–0.75 (0.59), MTL 0.49–0.61 (0.51), CI 90–95 (92), SI 84–95 (87), REL 28–30 (30), FLI 26–30 (29), PSI 1.3–1.8 (1.6), MFI 90–104 (102), ACI1 65–68 (68), ACI2 88–98 (96).

Same as worker except for standard queen modifications and as follows: pronotum with transverse rugae and rugoreticulae on lateral surface, changing to carinulae and punctae mesad; mesoscutum densely longitudinally rugose; scutellum rugose to rugoreticulate, sometimes with a smooth patch mesad; side of propodeum rugulose, with faint punctae, dorsum with transverse carinulae; wing venation as in Figure 167D.

#### Male.

Unknown.

#### Biology.

This species is known only from Winkler and Berlese samples of sifted leaf litter. It occurs from approximately 1000–2000 m elevation and inhabits a variety of montane mesic forest environments (e.g. cloud forest, wet oak forest, oak-pine forest, tropical subevergreen forest, *Liquidambar* forest).

#### Comments.

*Stenamma vexator* is not a particularly distinctive species, but it should be separable from similar species by the diagnostic character states given above and in the key. It bears the most resemblance to *Stenamma huachucanum*, which occurs in sympatry with *Stenamma vexator* at several sites. *Stenamma vexator* is usually larger than *Stenamma huachucanum* and has a longer, more distinctive petiole. Molecular phylogenetic data tentatively show that *Stenamma vexator* is sister to the *diversum* species group, which includes *Stenamma diversum* and *Stenamma tico* (Branstetter 2012, unpublished data).

Several aberrant populations are worth separating out as variants of *Stenamma vexator*. Variant 1 (Figure 166A–C) occurs at several sites in Puebla, Mexico. It has the propodeal spines tuberculate, the basal margin of the mandible nearly straight, and the clypeal teeth reduced to blunt nubs. Variant 2 (Figure 166D–F) is known from a few collections taken at high elevation between Oaxaca and Valle Nacional, in Oaxaca, Mexico. It is larger, darker, and has reduced sculpturing, with the pronotum mostly smooth and shiny. It also has the propodeal spines tuberculate, and the petiolar node pointing more vertically. I have not seen intermediate specimens between this variant and the holotype form of *Stenamma vexator*, so it is possible that this high elevation variant represents a distinct species. Variant 3 (Figure 166G–I) is known from the locality Coapilla in Chiapas, Mexico. These specimens are black, have reduced sculpture, and have the propodeal spines almost absent. The petiolar node is also sharper and pointing more vertically. Placement of variant 3 within *Stenamma vexator* is uncertain and needs to be confirmed with molecular data.

#### Material examined.

**MÉXICO: *Chiapas***: 5km NE Coapilla, 17.17297°N, 93.14612°W, 1850m, 26 May 2008 (M. G. Branstetter); ***Hidalgo***: 18km NE Jacala, nr El Alamo, [ca. 21.058°N, 99.052°W], 1700m, 10 Jun 1988 (S. & J. Peck); Tlanchinol, 43km SW Huejutla, [ca. 20.988°N, 98.662°W], 1 Aug 1983 (S. & J. Peck); 43km SW Huejutla, [ca. 20.988°N, 98.662°W], 1500m, 14 Jun 1983 (S. & J. Peck); 4–6km N Tlanchinol, [ca. 21.026°N, 98.644°W], 1590m, 6–11 Jul 1973 (A. F. Newton);***Oaxaca***: 20.6km SW Valle Nacional, 17.60404°N, 96.37786°W, 1730m, 13 Aug 2009 (M. G. Branstetter); 22.4km SW Valle Nacional, 17.59112°N, 96.39193°W, 1990m, 13 Aug 2009 (M. G. Branstetter); 23km SW Valle Nacional, Km76, [ca. 17.6364°N, 96.4700°W], 1300m, 26 Jul 1992 (R. S. Anderson); 32km SW Valle Nacional, Km85, [ca. 17.5046°N, 96.3897°W], 1650m 26 Jul 1992 (R. S. Anderson); ***Puebla***: 2.7km S Apulco, nr Zacapoaxtla, [ca. 19.911°N, 97.606°W], 1400m, 22 Jul 1987 (R. S. Anderson); 7.6km SW La Cumbre, [ca. 20.1416°N, 98.0217°W], 1585m, 23 Jul 1987 (R. S. Anderson); Teziutlan, [ca. 19.881°N, 97.304°W], 1350m, 18 Apr 1946 (F. Bonnet); 2km NE Teziutlan, [ca. 19.892°N, 97.296°W], 1220m, 19 Jun 1983 (S. & J. Peck); 24km N Xicotepec de Juarez, [ca. 20.282°N, 97.963°W], 1070m, 17 Jun 1983 (R. S. Anderson); ***Querétaro***: 40km E Landa de Matamoros, [ca. 21.297°N, 99.090°W], 1520m, 14 Jul 1969 (S. & J. Peck); ***Veracruz***: Cordoba, [ca. 18.908°N, 96.958°W], 4 Aug 1969 (S. B. Peck); 5km NE Coscomatepec, [ca. 19.100°N, 97.033°W], 22 Jun 1983 (Peck & Anderson); 1.9km S Huatusco, [ca. 19.193°N, 96.956°W], 1344m, 2–8 Aug 1969 (S. B. Peck); km38, 3.2km S Huatusco on Fortín road, [ca. 19.185°N, 96.959°W], 1300m, 3 Aug 1965 (Cornell Univ. Mexico Field Party); 7km E Huatusco, [ca. 19.207°N, 96.914°W], 22 Jun 1983 (Peck & Anderson); 2.7km N Teocelo, [ca. 19.40°N, 96.98°W], 1130m, 22–24 Jul 1973 (A. F. Newton).

### Stenamma zelum sp. n.

urn:lsid:zoobank.org:act:43EBCA14-1F18-4D1C-A54E-64B85C9F7829

http://species-id.net/wiki/Stenamma_zelum

**Figure 169.**
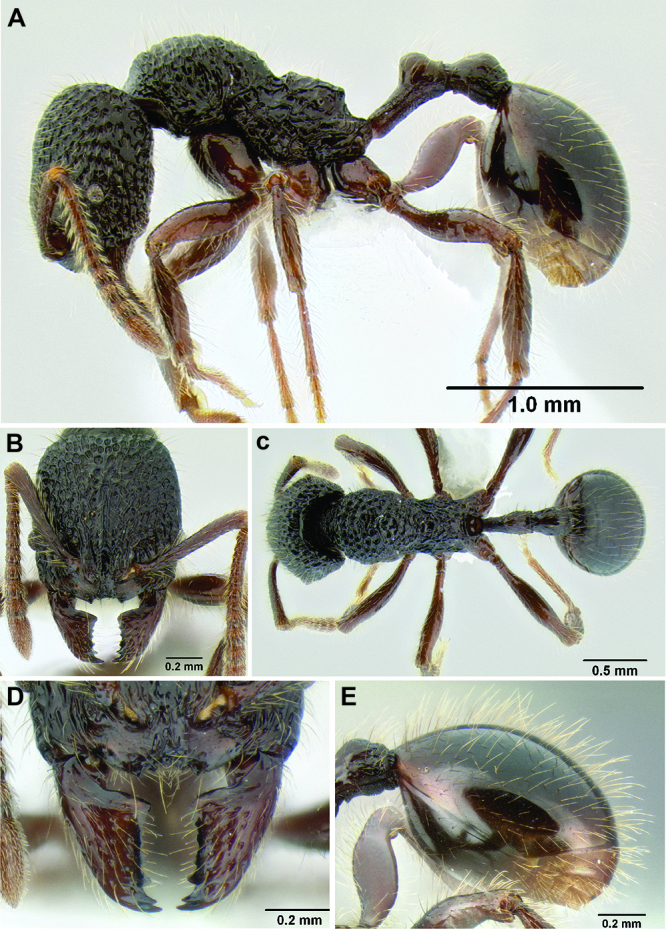
*Stenamma zelum* holotype worker (CASENT0622535) **A** Profile **B** Face **C** Dorsum **D **Anterior clypeal margin in anterodorsal view **E** Gaster.

**Figure 170.**
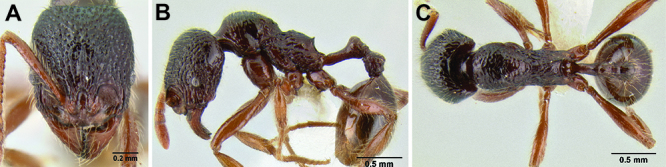
*Stenamma zelum* worker variant (INBIOCRI001280618) **A** Face **B** Profile **C** Dorsum.

**Figure 171.**
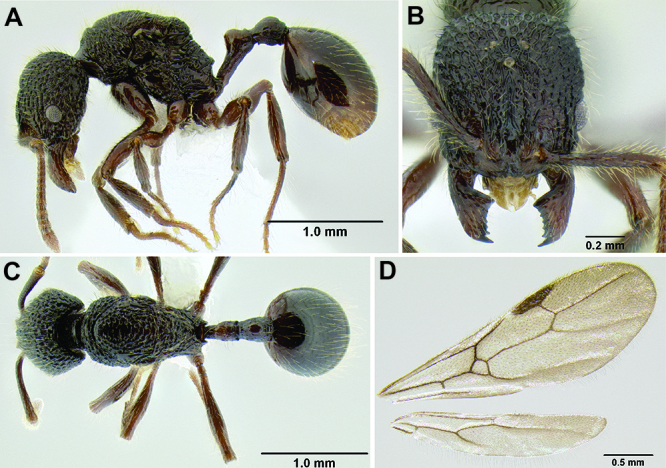
*Stenamma zelum* **A** Queen (CASENT0622537), profile **B** Same, face **C** Same, dorsum **D **Queen (CASENT0621980), wings.

Worker: Figures 169 , 170 ; Queen: Figure 171 ; Map: Figure 168

Stenamma mgb31 Branstetter, 2012: phylogeny.

#### Type material.

*Holotype worker*. NICARAGUA, Jinotega: Parque Nacional Cerro Saslaya, 13.77148°N, 85.01152°W ±10m, 1110m, 12 May 2011, ridgetop cloud forest, ex sifted leaf litter (LLAMA, collection Wa-D-03-1-35) [USNM, specimen CASENT0622535]. *Paratypes*: same data as holotype [1w, CAS, CASENT0622534], [1w, EAPZ, CASENT0622536]; same data but 13.77077°N, 85.00156°W ±20m, 850m, 8 May 2011 (LLAMA, Wm-D-02-2-02) [1w, ECOSCE, CASENT0623510], [1w, FMNH, CASENT0623511], [1w, ICN, CASENT0623512], [1dq, 1w, INBio, CASENT0623508, CASENT0623513], [1w, JTLC, CASENT0623514], [1w, LACM, CASENT0623515], [1dq, 1w, MGBPC, CASENT0623509, CASENT0623516]; 13.77000°N, 85.00326°W ±20m, 920m, 8 May 2011 (LLAMA, Wm-D-02-2-03) [1dq, 1w, MCZ, CASENT0623517, CASENT0623519], [1w, MZSP, CASENT0623520], [1w, UCD, CASENT0623521], [1w, UNAM, CASENT0623522], [1dq, 1w, USNM, CASENT0623518, CASENT0623523], [1w, UVGC, CASENT0623524].

#### Worker diagnosis.

Integument mostly dark red-brown to brown; medium to large-sized species (see HL, ML, PrW below); head and promesonotum mostly foveate to coarsely rugoreticulate; eye relatively small (EL 0.10–0.13, REL 12–16), somewhat bulging, subcircular to slightly oval-shaped, with 6–8 ommatidia at greatest diameter; gastral pilosity long, dense, and mostly suberect; propodeal spines reduced to sharp angles or small tubercles (PSL 0.12–0.16, PSI 1.0–1.3); anterior clypeal margin with a median excavation containing 2 sharp outer teeth and 2 smaller inner teeth (sharp to blunt); basal margin of mandible straight to slightly sinuous, without a basal notch or depression. *Similar species*: *Stenamma brujita*.

#### Geographic range.

Honduras to Panama.

#### Worker description.

(11 measured) HL 0.87–1.03 (0.99), HW 0.78–0.92 (0.85), FLD 0.26–0.31 (0.29), PCW 0.04–0.08 (0.07), SL 0.67–0.80 (0.74), EL 0.10–0.13 (0.11), ACL 0.58–0.69 (0.60), ML 1.15–1.38 (1.29), PrW 0.55–0.67 (0.63), PSL 0.12–0.16 (0.14), SDL 0.10–0.16 (0.13), PL 0.46–0.56 (0.53), PH 0.25–0.29 (0.29), PW 0.17–0.21 (0.20), PPL 0.27–0.32 (0.32), PPH 0.22–0.27 (0.25), PPW 0.21–0.26 (0.24), MFL 0.82–0.99 (0.93), MTL 0.65–0.80 (0.73), CI 86–92 (86), SI 83–89 (86), REL 12–16 (13), FLI 31–34 (34), PSI 1.0–1.3 (1.1), MFI 91–98 (92), ACI1 65–68 (65), ACI2 82–89 (82).

Medium to large-sized species; general body color mostly dark red-brown, with patches of brown and orange-brown on gaster, appendages lighter; setae golden brown; mandible with 6–9 teeth, consisting of 3 distinct apical teeth, a distinct basal tooth, and 2–5 inner teeth/denticles, which are usually worn and indistinct; basal margin of mandible straight to slightly sinuous, without a basal notch or depression; mandible mostly smooth and shiny, with scattered piligerous punctae and faint striae; anterior clypeal margin viewed from an anterodorsal angle with a median excavation containing 2 sharp outer teeth and 2 smaller inner teeth, which are sharp to blunt, and recessed behind median lobe of clypeus (not visible with mandibles closed); median lobe of clypeus usually with a pair of faint carinulae that diverge toward the anterior margin, apex of lobe with a short transverse carinula, remainder of clypeus mostly smooth and shiny, sometimes with a few additional rugulae on median lobe; posterior extension of clypeus between antennal insertions of moderate width (PCW 0.04–0.08), sides subparallel to slightly diverging posteriad; frontal lobes well-developed, but not completely covering torular lobes in full-face view (FLD 0.26–0.31, FLI 31–34); head appearing subrectangular (CI 86–92), with posterior margin depressed medially; eye relatively small (EL 0.10–0.13, REL 12–16), somewhat bulging, subcircular to slightly oval-shaped, with 6–8 ommatidia at greatest diameter; face usually strongly foveate to coarsely rugoreticulate, with a few longitudinal costae along midline, interstices with piligerous punctae, some high-elevation populations with face sculpture more polished and effaced, especially on side of head, which can be smooth and shiny; scape relatively short (SI 83–89), not reaching posterior margin of head when laid back; scape surface mostly smooth and shiny, with scattered piligerous punctae; flagellum with an indistinct 4-segmented antennal club; mesosoma usually densely sculptured, with promesonotum mostly foveate (especially on dorsum) and remaining surfaces foveate to rugoreticulate or rugose, in some high-elevation populations sculpture is reduced, with foveae on promesonotal dorsum appearing more like transverse furrows, and with side of promesonotum largely smooth and shiny; propodeal declivity mostly smooth and shiny, usually with a few transverse carinulae; promesonotum in profile low-domed, slightly asymmetrical, with the apex shifted anteriorly; anterior face of promesonotum longer and steeper than posterior face and mostly smooth; metanotal groove well-demarcated, of moderate width and depth; propodeal spines forming sharp angles or at most small tubercles (PSL 0.12–0.16, PSI 1.0–1.3); petiole relatively long (PL/HW 0.58–0.63), with node of average size (PH/PL 0.51–0.54); node in profile variable, usually forming a small roughly symmetrical dome with a rounded dorsum, but sometimes dome more asymmetrical with anterior face longer than posterior face, and sometimes with apex more sharp and positioned anterior of midpoint; postpetiole somewhat elongate (PPH/PPL 0.79–0.88) and globular, about the same height and volume as petiolar node (PPH/PH 0.88–0.98), postpetiolar node asymmetrical, with anterior face longer than posterior face; venter of postpetiole in profile usually distinctly sinuous, with anteroventral margin forming a small process; petiolar and postpetiolar nodes mostly smooth and shiny, remaining waist surfaces faintly punctate; gaster mostly smooth and shiny, with scattered piligerous punctae; most of body with a moderately dense layer of long suberect to subdecumbent pilosity; scape with layer of longer sparser suberect setae and layer of shorter subdecumbent setae; gastral setae not distinctly bilayered, forming a moderately dense layer of long suberect to subdecumbent setae; setae on legs suberect to decumbent, with longer suberect hairs on coxae and femoral venters.

#### Queen description.

(5 measured) HL 0.86–1.01 (0.92), HW 0.78–0.90 (0.84), FLD 0.27–0.32 (0.29), PCW 0.05–0.09 (0.06), SL 0.67–0.78 (0.71), EL 0.16–0.20 (0.17), ACL 0.59–0.67 (0.61), ML 1.25–1.48 (1.33), PrW 0.68–0.79 (0.71), PSL 0.15–0.19 (0.17), SDL 0.13–0.18 (0.14), PL 0.52–0.58 (0.54), PH 0.26–0.31 (0.28), PW 0.19–0.23 (0.21), PPL 0.31–0.33 (0.33), PPH P0.24–0.28 (0.25), PW 0.19–0.23 (0.21), MFL 0.83–0.98 (0.89), MTL 0.67–0.78 (0.69), CI 88–93 (92), SI 83–88 (84), REL 20–23 (20), FLI 33–35 (34), PSI 1.1–1.2 (1.1), MFI 92–94 (94), ACI1 65–67 (65), ACI2 83–89 (86).

Same as worker except for standard queen modifications and the following: mesoscutum foveate, except for a longitudinal strip of completely smooth cuticle, which crosses the entire surface; mesopleuron mostly smooth and shiny; propodeum carinate to rugose, with sculpture transversely wrapping around surface; wing venation as in Figure 171D.

#### Male.

Unknown.

#### Biology.

*Stenamma zelum* is known almost exclusively from Winkler and Berlese samples of sifted leaf litter. Only once has a stray individual (a dealate queen) been collected by general searching. This species is found from 350–1700 m elevation, but is most common at mid-elevations around 1000 m. It inhabits mature wet forest environments, such as montane rainforest and cloud forest.

#### Comments.

*Stenamma zelum* is a very distinctive species, unlikely to be confused with anything else. The only exception being *Stenamma brujita*, which is not closely related, but has many convergent traits (e.g. size and sculpture). Separation of these two species is discussed in detail in the comments section under *Stenamma brujita*, but I will restate here that these species have not been collected in sympatry, and should be easy to separate using locality data alone.

Molecular phylogenetic results indicate that *Stenamma zelum* is sister to the *expolitum* species group, which includes *Stenamma alas*, *Stenamma expolitico*, and *Stenamma expolitum* (Branstetter 2012). However, *Stenamma zelum* is distinct from these species and shares none of the diagnostic morphological character states of the species group.

Within my concept of *Stenamma zelum*, there are two morphotypes, the more common form, represented by the type series (Figure 169), and a high elevation form (variant 1; Figure 170), which is known only from a few sites above 1500 m in Costa Rica (e.g. 9km NE Vara Blanca, Est. Pittier, Las Alturas Biological Station). The variant has reduced sculpturing, in which the foveae have become less distinct, and large smooth patches are present on the side of the head and pronotum. Also, the clypeal teeth are reduced, with the inner teeth blunt and nearly absent.

Preliminary molecular phylogenetic results, which include several populations of each morphotype, show that the two forms separate into sister clades, suggesting that the high-elevation variant may represent a distinct species. However, I choose to recognize one species until variation within the high-elevation form is better understood.

#### Material examined.

**COSTA RICA:** ***Alajuela***: 10km E Monteverde, 10.3098°N, 84.7199°W, 880m, 1 Mar 2010 (J. Longino); 14km S Vol. Arenal, 10.333°N, 84.7167°W, 1000m, 29 Apr 1988 (J. Longino); ***Cartago***: Río Reventazon, 3–5km E Turrialba, [ca. 9.901°N, 83.685°W], 600m, 18–22 Jan 1973 (W. L. Brown); 4km E Turrialba, 9.90°N, 83.65°W, 550m, 13 May 1987 (J. Longino); ***Heredia***: P.N. Braulio Carrillo, 16km SSW Pt. Viejo, 10.3061°N, 84.0579°W, 500m, 16 Oct 2006 (TEAM); 9km NE Vara Blanca, 10.2333°N, 84.0833°W, 1500m, 12 Apr 2005 (ALAS); 10km NE Vara Blanca, 10.2333°N, 84.0833°W, 1500m, 12 Apr 2005 (ALAS); 16km SSE La Virgen, 10.2667°N, 84.0833°W, 1100m, 23 Feb 2001 (ALAS); 16km N Vol. Barba, 10.2667°N, 84.0833°W, 1020m, 9 Jul 1986 (J. Longino); ***Limon***: Res. Biol. Hitoy-Cerere, 9.667°N, 83.033°W, 500m, 30 Aug 1985 (J. Longino); ***Puntarenas***: Las Alturas Biol. St., 8.933°N, 82.833°W, 1500–2000m, 26 May 2007 (M. G. Branstetter); Est. Biol. Pittier, 9.0333°N, 82.9667°W, 1670m, 28 Jun 1995 (J. Longino); **HONDURAS:** ***Gracias a Dios***: Las Marias, 15.72235°N, 84.88480°W, 620m, 10 Jun 2010 (LLAMA); Las Marias, 15.72245°N, 84.88078°W, 510m, 10 Jun 2010 (LLAMA); Las Marias, 15.72200°N, 84.88325°W, 380m, 10 Jun 2010 (LLAMA); **NICARAGUA:** ***Matagalpa***: RN Cerro Musún, 12.95996°N, 85.23266°W, 750m, 1 May 2011 (LLAMA); ***Jinotega***: PN Cerro Saslaya, 13.77148°N, 85.01152°W, 1110m, 12 May 2011 (LLAMA); **PANAMA:** ***Bocas del Toro***: Fortuna-Chiriquí Gr. Rd., 8.783°N, 82.200°W, 1050m, 14 Jul 1987 (D. M. Olson).

**Figure 172.**
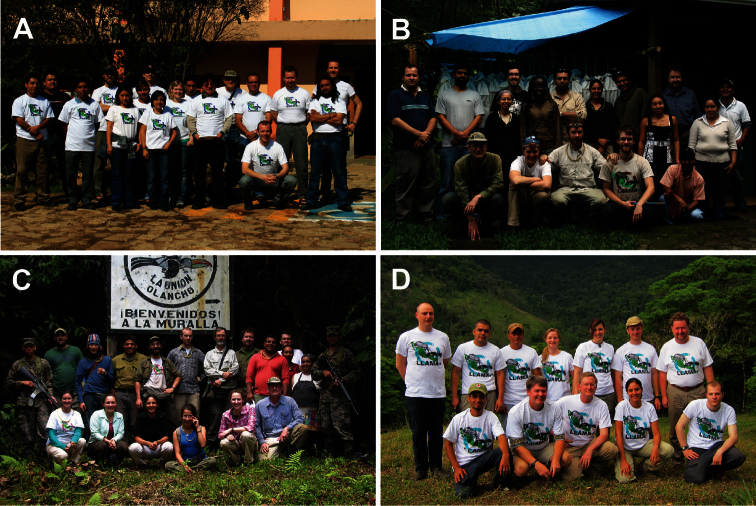
Expedition teams for each year of the Leaf Litter Arthropods of MesoAmerica (LLAMA) project **A** 2008 field team in Chiapas, Mexico **B** 2009 field team in Guatemala **C** 2010 field team in Honduras **D** 2011field team in Nicaragua.

## Supplementary Material

XML Treatment for Stenamma alas

XML Treatment for Stenamma andersoni

XML Treatment for Stenamma atribellum

XML Treatment for Stenamma brujita

XML Treatment for Stenamma callipygium

XML Treatment for Stenamma catracho

XML Treatment for Stenamma connectum

XML Treatment for Stenamma crypticum

XML Treatment for Stenamma cusuco

XML Treatment for Stenamma diversum

XML Treatment for Stenamma excisum

XML Treatment for Stenamma expolitico

XML Treatment for Stenamma expolitum

XML Treatment for Stenamma felixi

XML Treatment for Stenamma hojarasca

XML Treatment for Stenamma huachucanum

XML Treatment for Stenamma ignotum

XML Treatment for Stenamma lagunum

XML Treatment for Stenamma leptospinum

XML Treatment for Stenamma llama

XML Treatment for Stenamma lobinodus

XML Treatment for Stenamma longinoi

XML Treatment for Stenamma manni

XML Treatment for Stenamma maximon

XML Treatment for Stenamma megamanni

XML Treatment for Stenamma monstrosum

XML Treatment for Stenamma muralla

XML Treatment for Stenamma nanozoi

XML Treatment for Stenamma nonotch

XML Treatment for Stenamma ochrocnemis

XML Treatment for Stenamma pelophilum

XML Treatment for Stenamma picopicucha

XML Treatment for Stenamma saenzae

XML Treatment for Stenamma sandinista

XML Treatment for Stenamma schmidti

XML Treatment for Stenamma stictosomum

XML Treatment for Stenamma tiburon

XML Treatment for Stenamma tico

XML Treatment for Stenamma vexator

XML Treatment for Stenamma zelum

## Appendix

Key to *Stenamma* code names. (doi: 10.3897/zookeys.295.4905.app) File format: Mircrosoft Excell file (xlsx). **Explanation note:** This file contains a table that links the species names used in the present manuscript with the code names used in the phylogenetic study of Branstetter (2012).
